# Global, Regional, and National Burden of Cancer in Children Younger Than 5 Years, 1990–2019: Analysis of the Global Burden of Disease Study 2019

**DOI:** 10.3389/fpubh.2022.910641

**Published:** 2022-06-21

**Authors:** Hui-Ming Ren, Min-Qi Liao, Si-Xian Tan, Chen Cheng, Sui Zhu, Lu Zheng, Jun-Rong Ma, Ying-Jun Mu, Wan-Lin Li, Shi-Wen Zhang, Rui-Qing OuYang, Shu-Na Li, Yun-Feng Cui, Xing-Yao Ke, Ze-Yan Luo, Peng Xiong, Jun Liu, Li-Ping Li, Xiao-Feng Liang, Fang-Fang Zeng, Xue-Fen Su, Li-Yuan Han

**Affiliations:** ^1^Department of Rehabilitation Medicine, Hwa Mei Hospital, University of the Chinese Academy of Sciences, Ningbo, China; ^2^Department of Public Health and Preventive Medicine, School of Medicine, Jinan University, Guangzhou, China; ^3^Helmholtz Center Munich - German Research Center for Environmental Health, Neuherberg, Germany; ^4^Department of Preventive Medicine Laboratory, School of Public Health, Zunyi Medical University, Zunyi, China; ^5^Guangdong Provincial Key Laboratory for Breast Cancer Diagnosis and Treatment, Cancer Hospital of Shantou University Medical College, Shantou, China; ^6^Chinese Preventive Medicine Association, Beijing, China; ^7^Hwa Mei Hospital, University of Chinese Academy of Sciences, Ningbo, China; ^8^Department of Global Health, Ningbo Institute of Life and Health Industry, University of Chinese Academy of Sciences, Ningbo, China

**Keywords:** global burden of disease, childhood cancer, global health, secular trends, public health

## Abstract

**Objectives:**

To quantify the burden and variation trends of cancers in children under 5 years at the global, regional, and national levels from 1990 to 2019.

**Methods:**

Epidemiological data for children under 5 years who were diagnosed with any one childhood cancer were obtained from the Global Burden of Diseases, Injuries, and Risk Factors Study (GBD) from 1990 to 2019. The outcomes were the absolute numbers and rates of incidence, prevalence, mortality, and disability-adjusted life-years (DALYs) for different types of cancer.

**Results:**

In 2019, 8,774,979.1 incident cases (95% uncertainty interval [UI]: 6,243,599.2 to11,737,568.5) and 8,956,583.8 (6,446,323.9 to 12,364,520.8) prevalent cases of cancer in children under 5 years were identified worldwide; these cancers resulted in 44,451.6 (36,198.7 to 53,905.9) deaths and 3,918,014.8 (3,196,454.9 to 4,751,304.2) DALYs. From 1990 to 2019, although the numbers of incident and prevalent cases only decreased by −4.6% (−7.0 to −2.2) and −8.3% (−12.6 to −3.4), respectively, the numbers of deaths and DALYs clearly declined by −47.8% (−60.7 to −26.4) and −47.7% (−60.7 to −26.2), respectively. In 2019, the middle sociodemographic index (SDI) regions had the highest incidence and prevalence, whereas the low SDI regions had the most mortality and DALYs. Although all of the SDI regions displayed a steady drop in deaths and DALYs between 1990 and 2019, the low-middle and low SDI regions showed increasing trends of incidence and prevalence. Leukemia remained the most common cancer globally in 2019. From 1990 to 2019, the burdens of leukemia, liver cancer, and Hodgkin's lymphoma declined, whereas the incidence and prevalence of other cancers grew, particularly testicular cancer.

**Conclusions:**

The global childhood cancer burden in young children has been steadily decreasing over the past three decades. However, the burdens and other characteristics have varied across different regions and types of cancers. This highlights the need to reorient current treatment strategies and establish effective prevention methods to reduce the global burden of childhood cancer.

## Introduction

Children in the early phases of childhood are the main targets of public health policies. The under-five mortality rate (U5MR) is a valid indicator of the quality of national economics and healthcare systems (1). In 2000, the Millennium Development Goal 4 called for a two-thirds reduction in the U5MR, which dropped from 12.7 million in 1990 to around 6 million in 2015 (2). The Sustainable Development Goal 3 (SDG-3) also aimed to reduce U5MRs to at least 25 per 1,000 births by 2030 (3). Immense progress has been made in reducing worldwide U5MRs; this has been attributed to better control of the main causes of U5MR, such as lower respiratory tract infections, neonatal preterm birth complications, and neonatal encephalopathy arising from birth asphyxia and trauma (4). Data from the World Bank and GLOBOCAN have revealed an inverse correlation between U5MR and childhood cancer, with the burden of childhood cancer increasing following improvements in child healthcare systems and reduced competition from other causes of mortality (5).

Previous research has shown that, globally, approximately 175,000 new cases of cancer in children are diagnosed every year and that this number might be a significant under-representation of global childhood cancer incidence due to the under-diagnosis and under-registration of cancer (6). Approximately 80% of childhood cancer cases can be diagnosed and treated with advanced treatments and supportive care (7). The overall improvements in clinical treatments and supportive care have dramatically increased the cure rates of childhood cancers (5). However, not all children with cancer diagnoses have benefitted from these advances. Eighty percent of childhood cancer cases occur in low- and middle-income countries (LMICs) (8); this burden on LMICs has been projected to increase by about 30% by the end of the decade (5). With <5% of global resources dedicated to cancer treatment (5), children with cancer in these countries have much poorer prognoses and survival rates than those in other countries (8). This could cause an alarmingly significant difference in the survival rates and long-term quality of life of children with malignancies in high-income countries (HICs) and LMICs (9). Such differences can profoundly affect future healthcare needs and lead to the disproportionate loss of future human capital and productivity.

Cancer is a genetic disease. The genetic and epigenetic aberrations in human cells are primarily attributable to the interaction of genetic and exogenous cancer risk factors (10). Cancers in children are likely linked to prenatal and congenital factors (11), with at least 10% of pediatric cancer patients harboring a germline mutation in a cancer predisposition gene (12). For instance, children with certain constitutional genetic abnormalities in the *TP53* gene face an increased risk of acute lymphoblastic leukemia (ALL) (13); germline mutations in *SUFU* and *PTCH1* have also been revealed as risk factors for infant medulloblastoma (14). Genetic susceptibility, however, is not the only contributing factor to the development of cancers. Environmental factors such as air pollution due to fast industrialization; unhealthy lifestyles like nutritional deficiencies, excess body weight, and alcohol consumption; infectious diseases or limited access to safe water, sanitation, and hygiene can also contribute to the childhood cancer burden in resource-limited settings (15–18). Similarly, modern Western lifestyle risk factors (high energy-dense food consumption, nutrient-poor diet, and insufficient physical activity) have become increasingly common in developed Asian countries; these factors drive the simultaneous rise in obesity, which is more closely related to a high risk of cancer in HICs than in LMICs (19). Of note, the high burden of malignant skin melanoma in several developed countries can be partly attributed to chronic low-level arsenic exposure in the water supply in the United States of America (20) and exposure to high levels of ambient UV radiation in white populations in Australia and New Zealand (21); however, inherited predispositions account for a much higher proportion of cancers in children (22).

A recent study (23) based on the Global Burden of Diseases, Injuries, and Risk Factors Study 2017 (GBD 2017 study) estimated that 11.5 million disability-adjusted life-years (DALYs) globally could be attributed to childhood cancers in children aged 0 to 19 years. A more recent study based on GBD 2019 further indicated that cancers could result in 23.5 million DALYs in adolescents and young adults (aged 15–39 years) worldwide (24). However, these two studies (23, 24) focused on the global burden of childhood cancer in the broad age group of 0 to 19 and 15 to 39 years, not including the age group under-five years. The common types of childhood cancers varied across ages, with some cancers being more prevalent in specific age groups (25), and therefore, heterogeneity in the cancer burden across age groups should not be ignored (1). Assessing age-specific burdens by cause is thus an essential component of health surveillance. In addition, the GBD 2017 study (23) did not analyze trends in childhood cancers over the previous years. Valid trend analysis can not only offer valuable insights into the possible successes of past policies and programs, but it can also identify epidemiological transitions over time and predict the epidemic status of different regions or countries in terms of specific childhood cancers. However, neither the GBD 2017 (23) nor GBD 2019 study (24) analyzed trends of childhood cancers in the past three decades. An accurate estimate of the global burden of childhood cancer in children under 5 years is, therefore, a crucial step required to inform policymakers and guide policy development, resource investment and allocation, and future priorities for public health action. To date, studies focusing on the global burden of childhood cancer in children under 5 years have been rare.

Hence, we used data from the GBD from 1990 to 2019 in 204 countries and territories and focused on children aged under 5 years to (i) assess the burden (incidence, prevalence, deaths, and DALYs) of total and specific childhood cancer globally and in different areas (regional and national) in 2019 and (ii) explore secular changes in childhood cancer burden in specific regions and countries from 1990 to 2019.

## Materials and Methods

### Overview

The Global Burden of Diseases, Injuries, and Risk Factors Study 2019 (GBD 2019) provided a standardized approach for estimating mortality, incidence, and prevalence by cause and age on a global scale; it covered 204 countries and territories, grouped into 21 regions and seven super-regions (high-income; Central Europe, Eastern Europe, and Central Asia; Latin America and Caribbean; Southeast Asia, East Asia, and Oceania; South Asia; Sub-Saharan Africa; and North Africa and the Middle East). In brief, childhood cancer data were obtained from cancer registries, vital status registration systems, verbal autopsy data, and other sources that could provide partial or complete information on cancer incidence and mortality (24). The time period spanned 29 years, from 1990 to 2019. An integrative Bayesian meta-regression method was conducted using DisMod-MR2.1 to estimate a generalized negative binomial model for all epidemiological data, including the global burden of disease (26). Details of the methodology and main changes made to the GBD 2019 have been reported previously (26). Parameters such as incidence, prevalence, mortality, and DALYs have been reported with 95% uncertainty intervals (UIs), which is a range of values determined by the 2.5th and 97.5th percentiles of 1,000 estimated values in ascending order (26). Annual data on different indicators for several childhood cancers in children under 5 years by region and country were collected via the GBD Results Tool on the Global Health Data Exchange website: http://ghdx.healthdata.org/gbd-results-tool (27)].

The definitions and diagnostic criteria of childhood cancers listed in the GBD 2019 were derived from the International Classification of Diseases (ICD-9 and ICD-10). The coding of this study was concordant with the Guidelines for Accurate and Transparent Health Estimates Reporting recommendations (GATHER) (Supplementary Table 1 in Appendix 1) (28). Considering that some cancers are more prevalent in some age groups and the limitation of GBD cancer data across age groups, we used total and each of all accessible childhood cancer data in young children aged under 5 years (liver cancer, leukemia, brain and nervous system cancers, malignant skin melanoma, testicular cancer, kidney cancer, Hodgkin's lymphoma, non-Hodgkin's lymphoma, other malignant neoplasms, and other neoplasms) during the 1990 to 2019 period for 204 countries or territories [details of the definitions and ICD codes included under “other neoplasms” are described in Appendix 1 (29)].

### Incidence and Prevalence

Using updated data available in the literature, survey and surveillance data, health insurance claims, and other results, the estimates of incidence and prevalence were synthesized via the Bayesian meta-regression tool DisMod-MR2.1 (30). The estimates of incidence were obtained from the mortality estimates for each childhood cancer type divided by the corresponding mortality-to-incidence ratios (MIRs) (23). As prevalence data were not available for most of the countries, the prevalence was estimated from an incidence. The estimates of incidence and prevalence were adjusted for readmission, outpatient utilization, non-primary diagnosis, or a combination of the above (30). Details of the estimation methods used in the GBD 2019 have been described previously (26).

### Death, Mortality, and DALY Estimation

As morbidity and mortality data were not simultaneously available from some registries, MIRs, which were calculated using a linear-step mixed-effect model and spatial-temporal Gaussian regression, were adopted to estimate mortality by transferring the incidence data from cancer registries (23). The Cause of Death Ensemble model (CODEm), which uses all of the available covariates if the data quality varies and chooses the best model that reflects all of the input data, was used to generate the estimates for each location, year, age, and sex (31). The covariates in the CODEm were expected to have a plausible, but not necessarily causal, relationship with childhood cancer (31).

Using the cause of death database of the GBD 2019, cause-specific mortality and DALYs were estimated. DALYs measure the gap between current health status and an ideal health situation in which the entire population could be free of disease or disability and live to an advanced age. DALYs were computed by summing up years of life lost (YLLs) and years lived with disability (YLDs) by age, location, cause, and years (1990 and 2019).

### Sociodemographic Index

The sociodemographic index (SDI) is a measure that estimates a location's position on a development spectrum; it comprises per capita income, the average educational level of the population, and the total fertility rate under 25 years of age (32). The SDI value between 0 and 1 was defined by three components, including average educational attainment in the population older than 15 years, total fertility rate under 25 years of age, and income per capita (23, 30), setting the scales based on the observed minima and maxima for each component over the estimation period (32). The countries and territories were divided into five groups by SDI quintiles (high, high-middle, middle, low-middle, and low SDI regions) (33). The distributions of incidence, prevalence, deaths, mortality, and DALYs by SDI were analyzed to explore the effect of socioeconomic development on the under-5-year cancer burden.

### Percentage Change and Ranking

To assess secular trends over 30 years, the annual or overall percentage changes from 1990 to 2019 in incidence, prevalence, deaths, and DALYs were estimated by calculating the changes between pairs of 1,000 draws from the bootstrap distributions of annualized estimates; we then used the means and 25th and 975th ordered values of the combined distribution (34).

Based on the absolute numbers and rates (per 100,000 children) of incidence and DALYs, the ranks of the top 10 countries of specific childhood cancer burden in children younger than 5 years were explored.

## Results

### Global, Regional, National, and SDI Regional Burdens

**Figure 1:**
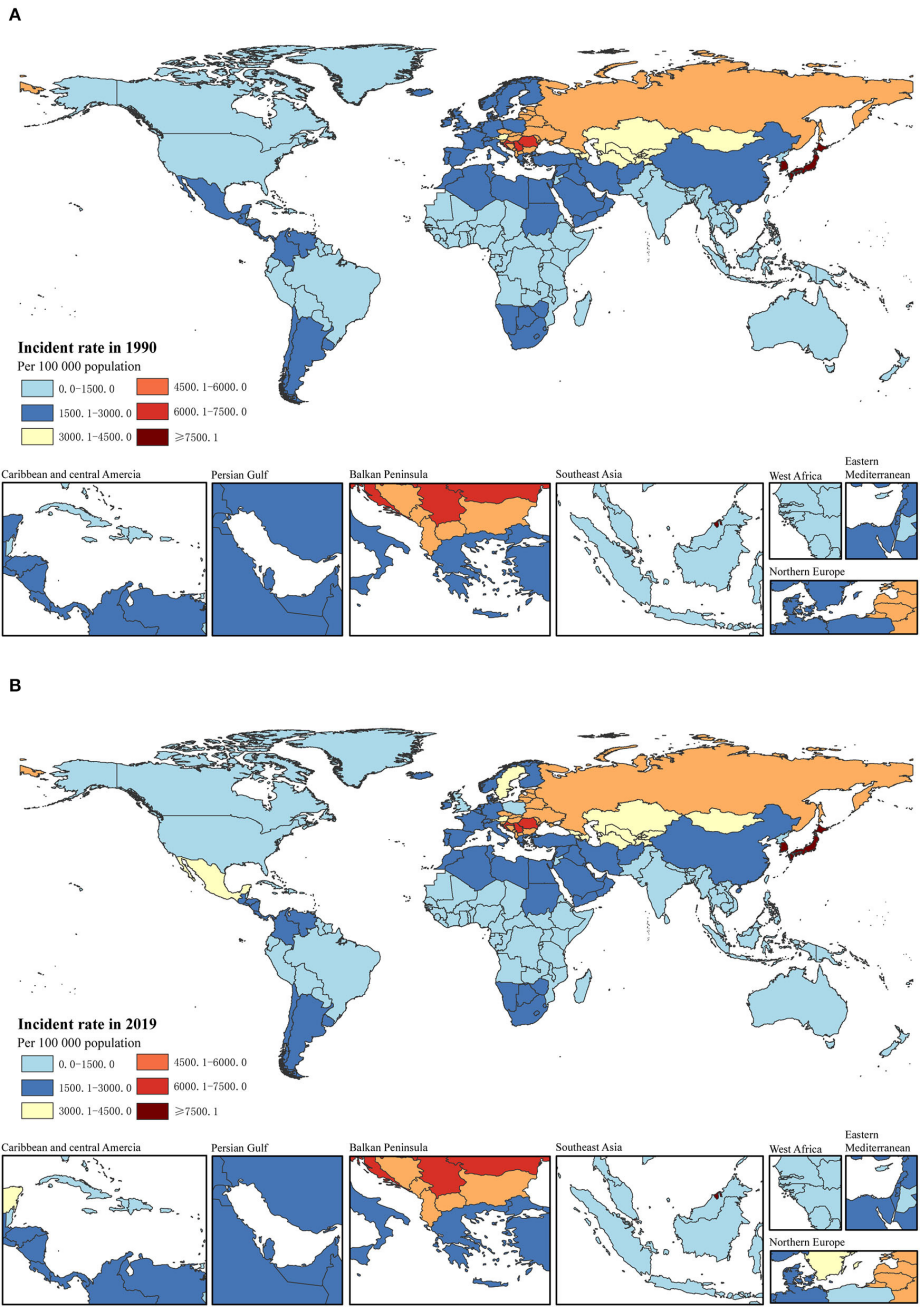
Incident rate per 100,000 of total childhood cancers in 1990 **(A)** and 2019 **(B)** in 204 countries.

**Figure 2:**
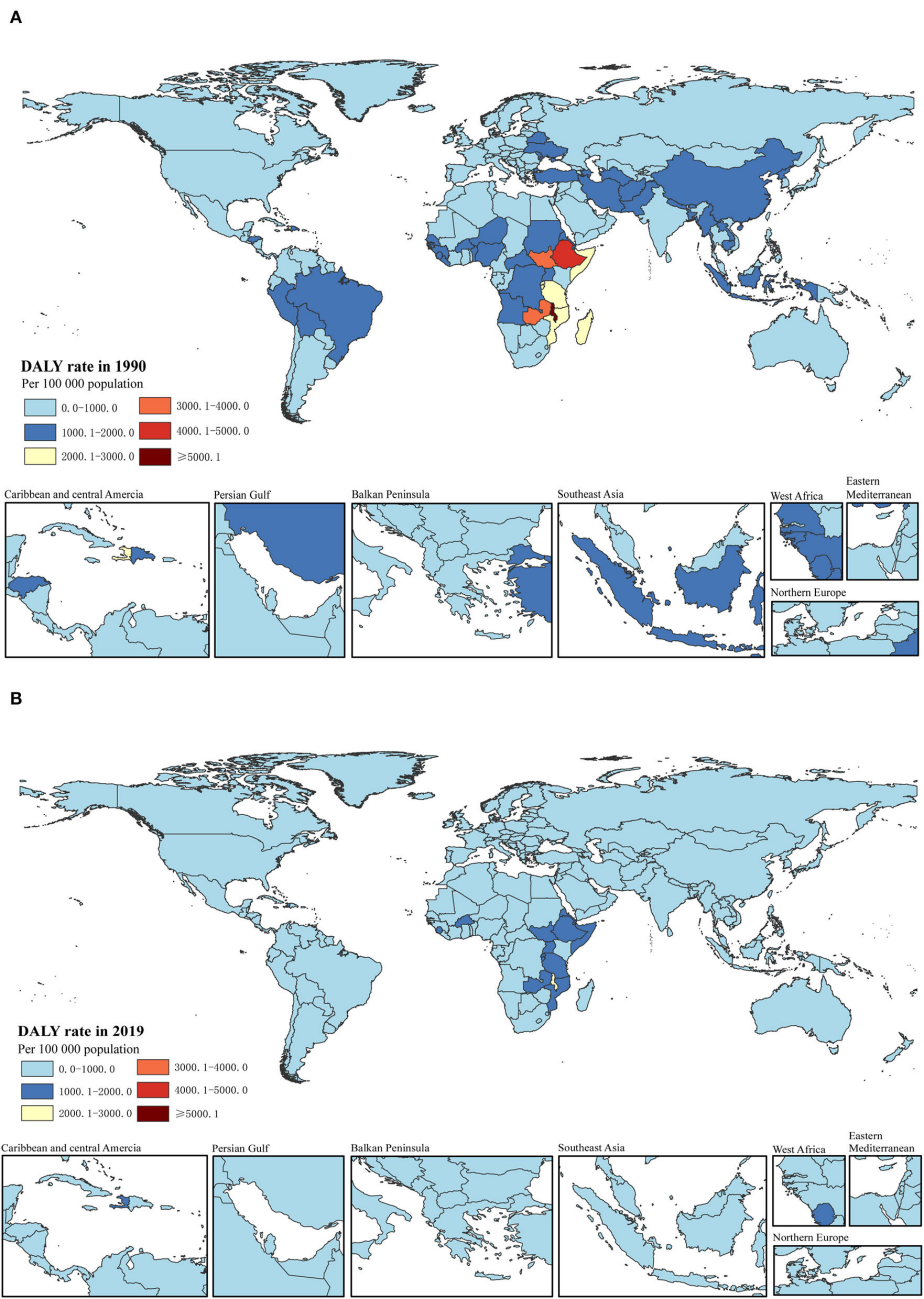
DALY rate per 100,000 of total childhood cancers in 1990 **(A)** and 2019 **(B)** in 204 countries. DALY, disability-adjusted life-years.

In 2019, the worldwide number of incident cases of cancer in children under 5 years was 8,774,979.1 (6,243,599.2 to 11,737,568.5) and of prevalent cases was 8,956,583.8 (6,446,323.9 to 12,364,520.8). The number of childhood cancer deaths was 44,451.6 (36,198.7 to 53,905.9), which corresponded to 3,918,014.8 (3,196,454.9 to 4,751,304.2) DALYs. The burden of childhood cancer declined steadily from 1990 to 2019, especially for the number of deaths and DALYs. The percentage changes were −4.6% (−7.0 to −2.2) for incident cases, −8.3% (−12.6 to −3.4) for prevalent cases, −47.8% (−60.7 to −26.4) for deaths, and −47.7% (−60.7 to −26.2) for DALYs, respectively. When stratified by gender, the incident and prevalent cases tended to be higher among girls whereas cancer death and DALYs were lower in two separated years, compared to their male counterparts, with greater declines in cancer burden between 1990 and 2019 [Table 1, Figures 1, 2, and Supplementary Figure 1 in Appendix 2 (35)].

**Table 1:** Incident cases, prevalence cases, deaths and DALYs of total neoplasms in children under 5-year-old globally and by GBD super-regions, SDI groups in 1990 and 2019.

	**Incidence cases**			**Prevalence cases**			**Deaths**			**DALYs**		
	**1990**	**2019**	**Percentage change 1990 and 2019**	**1990**	**2019**	**Percentage change 1990 and 2019**	**1990**	**2019**	**Percentage change 1990 and 2019**	**1990**	**2019**	**Percentage change 1990 and 2019**
**Global**												
**Overall**	9200909.6 (6495831.8 to 12413218.7)	8774979.1 (6243599.2 to 11737568.5)	−4.6% (−7.0 to −2.2)	9763754.5 (6934825.2 to 13322073.0)	8956583.8 (6446323.9 to 12364520.8)	−8.3% (−12.6 to −3.4)	85196.7 (60361.4 to 112686.7)	44451.6 (36198.7 to 53905.9)	−47.8% (−60.7 to −26.4)	7487603.1 (5311977.2 to 9900566.2)	3918014.8 (3196454.9 to 4751304.2)	−47.7% (−60.7 to −26.2)
Boys	4324889.9 (3037574.1 to 5866100.5)	4157747.9 (2967173.0 to 5596138.2)	−3.9% (−6.5 to −1.2)	4558377.2 (3261385.6 to 6288293.1)	4275033.2 (3089203.5 to 5918575.5)	−6.2% (−12.9 to −0.9)	47119.6 (28148.0 to 67456.7)	26606.7 (21127.9 to 32952.7)	−43.5% (−59.8 to −8.7)	4134312.7 (2465752.9 to 5924803.3)	2341849.6 (1861143.0 to 2899091.3)	−43.4% (−59.6 to −8.3)
Girls	4876019.7 (3467073.9 to 6536124.3)	4617231.2 (3293136.0 to 6190075.0)	−5.3% (−7.9 to −2.7)	5205377.3 (3700209.8 to 7038824.4)	4681550.6 (3368489.9 to 6427874.8)	−10.0% (−15.7 to −4.5)	38077.1 (25944.5 to 50332.4)	17844.9 (14817.7 to 21330.8)	−53.1% (−64.8 to −30.5)	3353290.4 (2285236.7 to 4402848.7)	1576165.2 (1312245.4 to 1880723.7)	−53.0% (−64.7 to −30.3)
**GBD super–regions**												
Central Europe, Eastern Europe, and Central Asia	1723140.8 (1227568.6 to 2333662.3)	1273706.4 (915288.2 to 1713841.9)	−26.1% (−26.8 to −25.3)	1693846.7 (1190894.9 to 2393629.4)	1244682.7 (886083.6 to 1752205.1)	−26.5% (−27.9 to −24.8)	3349.3 (3027.4 to 3836.0)	1284.7 (1084.5 to 1522.2)	−61.6% (−68.8 to −53.6)	293876.4 (266593.9 to 335513.6)	113749.4 (95422.0 to 135437.4)	−61.3% (−68.6 to −53.1)
High income	1788130.4 (1272635.3 to 2391961.5)	1484499.8 (1079750.4 to 1981046.5)	−17% (−20.2 to −13.5)	1885055.9 (1342628.4 to 2589934.2)	1546738.0 (1121944.8 to 2103483.6)	−17.9% (−22.4 to −12.7)	3284.9 (3068.4 to 3624.4)	1674.4 (1474.6 to 1857.7)	−49.0% (−56.4 to −42.4)	293315.8 (273152.9 to 320890.5)	153223.8 (132536.9 to 174974.7)	−47.8% (−55.9 to −40.6)
Latin America and Caribbean	882394.7 (608686.5 to 1214682.4)	840088.7 (590111.3 to 1139909.5)	−4.8% (−8.0 to −1.5)	903095.2 (629376.9 to 1269781.3)	852416.2 (605822.2 to 1174892.0)	−5.6% (−10.1 to −0.8)	5634.3 (4746.2 to 6653.0)	3145.5 (2461.2 to 3942.0)	−44.2% (−59.0 to −25.1)	493431.7 (415550.0 to 581775.9)	276590.9 (216149.8 to 347827.2)	−43.9% (−58.9 to −24.5)
North Africa and Middle East	951369.5 (665623.7 to 1279773.7)	1052383.8 (740809.4 to 1419417.8)	10.6% (9.3 to 12.0)	992683.5 (694849.1 to 1344612.7)	1089945.4 (757215.4 to 1496119.3)	9.8% (3.4 to 15.8)	5562.6 (3355.3 to 7964.7)	2977.9 (2142.2 to 3849.9)	−46.5% (−65.1 to −2.7)	490024 (297078.1 to 700637.2)	263794.8 (190532.7 to 341275.5)	−46.2% (−64.9 to −2.5)
South Asia	734033.9 (506005.8 to 1015418.0)	801216.1 (547095.2 to 1103573.7)	9.2% (2.8 to 16.5)	739521.6 (501795.7 to 1035151.0)	784800.7 (537236.0 to 1106327.2)	6.1% (−5.9 to 19.8)	16898.9 (8592.9 to 26571.7)	9298.5 (7051.1 to 11778.5)	−45.0% (−64.6 to −0.5)	1473870.2 (749682.0 to 2317724.7)	814344.1 (617468.4 to 1030524.1)	−44.7% (−64.5 to −0.1)
Southeast Asia, East Asia, and Oceania	2270907.3 (1570210.1 to 3092411.9)	1804384.6 (1291373.2 to 2449959.8)	−20.5% (−26.6 to– 13.6)	2674152.6 (1917718.8 to 3577190.8)	1923991.2 (1440693.2 to 2605709.1)	−28.1% (−36.4 to −17.1)	32318.7 (23977.3 to 42968.5)	8508.1 (7238.6 to 10215.0)	−73.7% (−80.5 to −61.8)	2856696.2 (2121839.2 to 3791790.1)	757543.4 (644936.0 to 908437.9)	−73.5% (−80.4 to −61.5)
Sub–Saharan Africa	850933.1 (590003.9 to 1167991.2)	1518699.6 (1049263.4 to 2107385.1)	78.5% (74.6 to 81.7)	875398.9 (604611.7 to 1194743.8)	1514009.7 (1046723.8 to 2115634.1)	73.0% (56.6 to 86.4)	18147.9 (10272.6 to 26687.6)	17562.5 (12667.5 to 23383.9)	−3.2% (−34.1 to 59.1)	1586388.8 (898930.5 to 2330960.4)	1538768.4 (1112756.2 to 2047771.8)	−3.0% (−34.0 to 59.7)
**SDI regions**												
High SDI	1734203.8 (1239925.2 to 2313504.7)	1407295.9 (1026861.1 to 1866085.6)	−18.9% (−22.0 to −15.4)	1825972.4 (1304452.8 to 2515609.9)	1467826.7 (1072578.0 to 1987130.2)	−19.6% (−23.8 to −14.7)	2908.1 (2730.6 to 3152.6)	1477.4 (1291.8 to 1631.2)	−49.2% (−57.1 to −43.1)	259775.9 (243713.6 to 279774.1)	135253.3 (115898.5 to 152491.8)	−47.9% (−56.7 to −41.1)
High–middle SDI	2500632.6 (1775358.4 to 3376793.8)	1910770.0 (1377109 to 2556753.5)	−23.6% (−25.8 to −21.0)	2626117.8 (1875233.8 to 3609760.4)	1972616.1 (1442115.5 to 2712615.1)	−24.9% (−28.2 to −20.8)	13088.2 (10883.8 to 15316.5)	4025.0 (3430.3 to 4680.1)	−69.2% (−76.2 to −60.0)	1155867.2 (962774.0 to 1350783.1)	360729.4 (307016.2 to 419682.0)	−68.8% (−75.9 to −59.1)
Middle SDI	2874093.4 (2015535.0 to 3887168.3)	2496805.3 (1769372.3 to 3331789.2)	−13.1% (−16.7 to −9.4)	3117817.7 (2213604.7 to 4223547.0)	2643172.1 (1898580.5 to 3646328.3)	−15.2% (−21.0 to −8.2)	27580.6 (21091.6 to 35504.6)	9288.8 (7869.5 to 10878.2)	−66.3% (−74.7 to −53.5)	2431797.3 (1863025.4 to 3130801.0)	822281.0 (696799.7 to 964410.1)	−66.2% (−74.6 to −53.1)
Low–middle SDI	1352240.3 (938250.9 to 1841305.1)	1449969.8 (1011053.3 to 1976767.7)	7.2% (4.5 to 10.4)	1418522.3 (993357.6 to 1945515.4)	1470777.4 (1026756.6 to 2046727.3)	3.7% (−5.9 to 12.5)	21651.8 (12691.2 to 31947.3)	10960.7 (8513.8 to 13598.3)	−49.4% (−65.0 to −17.1)	1895097.6 (1112499.9 to 2790072.0)	961294.7 (745102.3 to 1192937.8)	−49.3% (−64.9 to −17.1)
Low SDI	735500.4 (515170.7 to 1005412.4)	1327042.6 (919426.7 to 1828045.3)	80.4% (75.3 to 85.3)	770918.7 (529273.6 to 1052854.5)	1397138.2 (969633.3 to 1944557.8)	81.2% (59.6 to 100.4)	19919.8 (10650.8 to 30411.0)	18662.4 (13603.5 to 24697.8)	−6.3% (−36.6 to 61.2)	1740838.1 (930177.6 to 2658873.9)	1635176.5 (1193681.1 to 2164245.2)	−6.1% (−36.5 to 61.8)

*Data in parentheses are 95% uncertainty intervals (UIs) unless otherwise stated*.

*DALYs, disability-adjusted life-years; GBD, Global Burden of Disease; SDI, Sociodemographic index*.

When we considered the GBD super-regions, the burden of childhood cancer varied substantially. The absolute numbers of incidence and prevalence for total childhood neoplasms were the highest in Southeast Asia, East Asia, and Oceania in 1990 and 2019. The highest numbers of deaths and DALYs also occurred in Southeast Asia, East Asia, and Oceania in 1990, but the highest numbers overall were observed in Sub-Saharan Africa in 2019. Increasing trends in the incident and prevalent cases were observed in three regions: North Africa and the Middle East, South Asia, and Sub-Saharan Africa between 1990 and 2019; the most evident increases were in Sub-Saharan Africa, with a percentage change of 78.5% (74.6 to 81.7) for incident cases and 73.0% (56.6 to 86.4) for prevalent cases. However, decreasing trends were observed in the remaining regions, with the highest decreases observed in Central Europe, Eastern Europe, and Central Asia (−26.1% [−26.8 to −25.3] for incident cases and −26.5 [−27.9 to −24.8] for prevalent cases) and Southeast Asia, East Asia, and Oceania (−20.5% [−26.6 to−13.6] for incident cases and −28.1% [−36.4 to −17.1] for prevalent cases). In contrast, declines in the numbers of deaths and DALYs were observed in all seven super-regions, except for Sub-Saharan Africa. Remarkable decreases were observed in Southeast Asia, East Asia, and Oceania, with percentage changes of −73.7% (−80.5 to −61.8) for cancer deaths and −73.5% (−80.4 to −61.5) for DALYs, and in Central Europe, Eastern Europe, and Central Asia, with percentage changes of −61.6% (−68.8 to −53.6) for cancer deaths and −61.3% (−68.6 to −53.1) for DALYs [Table 1 and Supplementary Tables 1–3 in Appendix 2 (35)].

**Figure 3:**
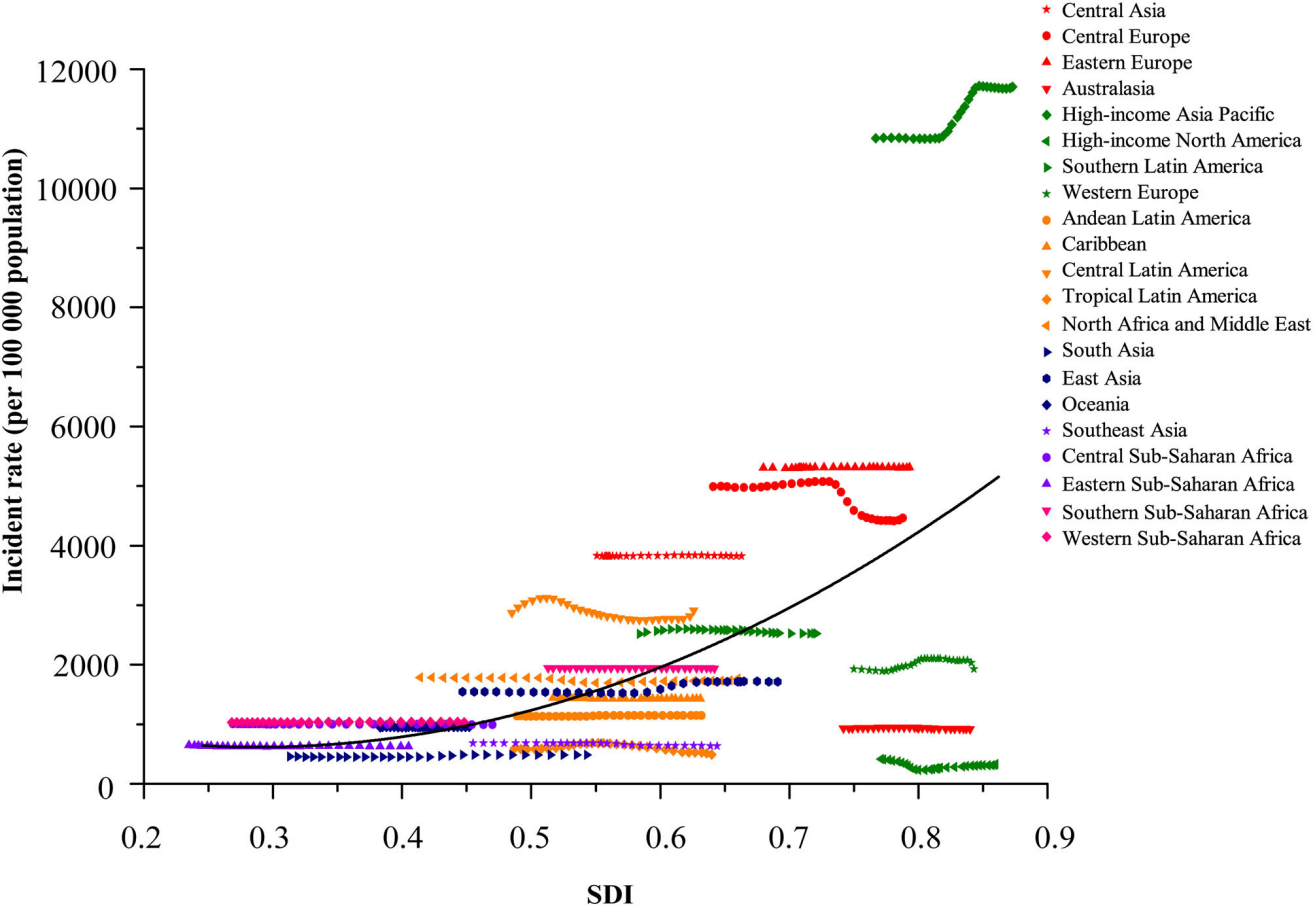
Coevolution of incident rate per 100,000 of total childhood cancers with SDI for 21 GBD regions, for both sexes combined, between 1990 and 2019. Each point in a line represents 1 year starting at 1990 and ending at 2019. Solid black line shows expected values across the spectrum of the SDI. SDI value between 0 and 1 was defined by three dimensions (lag-distributed income per capita, total fertility rate in those younger than 25 years, and average educational attainment among population older than 15 years) by setting the scales based on the observed minima and maxima for each component over the estimation period. GBD, Global Burden of Diseases, Injuries and Risk Factors Study. SDI, Sociodemographic Index.

**Figure 4:**
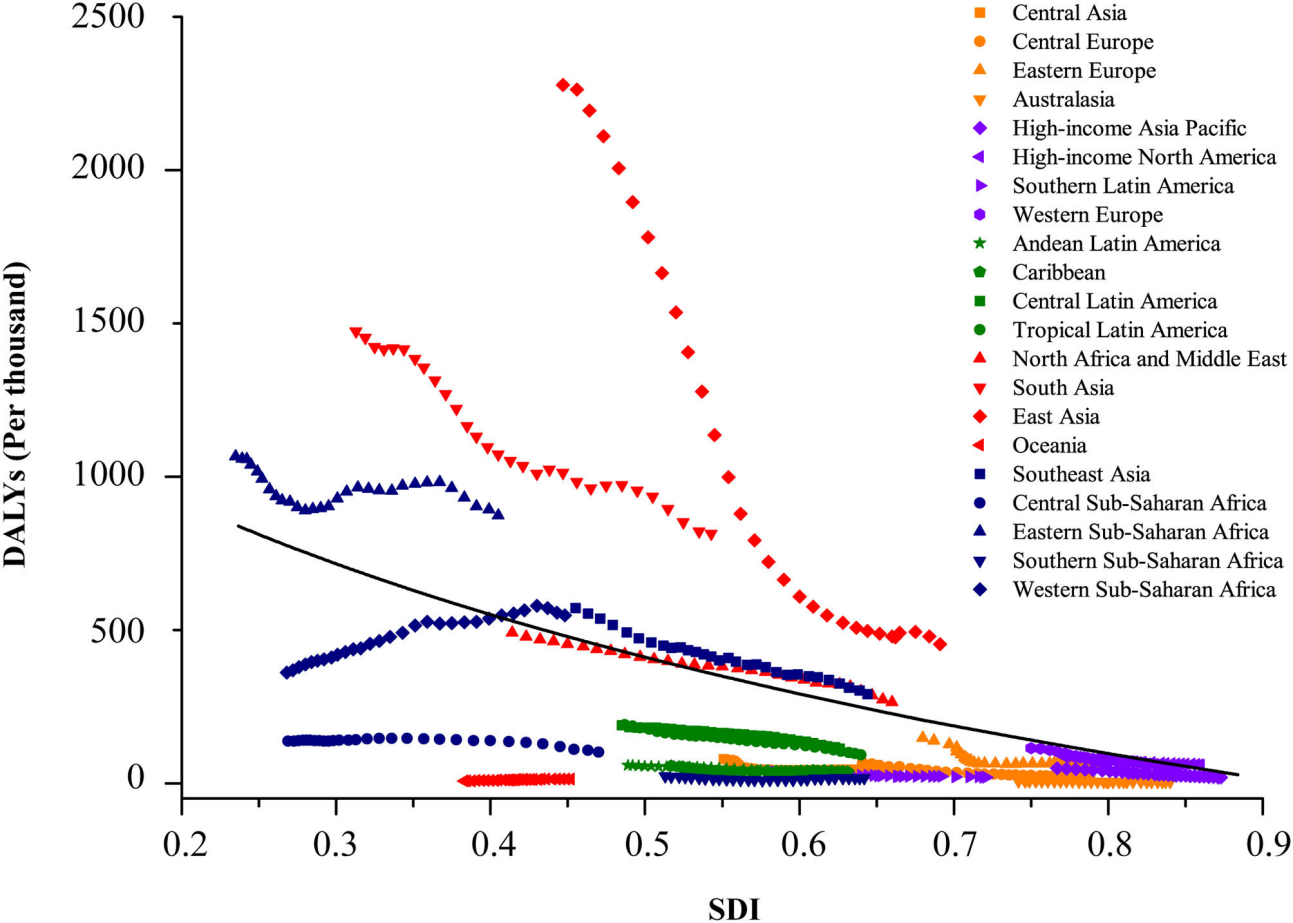
Coevolution of DALYs per thousand of total childhood cancers with SDI for GBD regions, for both sexes combined, between 1990 and 2019. Each point in a line represents 1 year, starting from 1990 and ending in 2019. Solid black line shows expected values across the spectrum of the SDI. SDI value between 0 and 1 was defined by three dimensions (lag-distributed income per capita, total fertility rate in those younger than 25 years, and average educational attainment among population older than 15 years) by setting the scales based on the observed minima and maxima for each component over the estimation period. DALY, disability-adjusted life-years. GBD, Global Burden of Diseases; Injuries and Risk Factors Study; SDI, Sociodemographic Index.

The burden of disease was also considerably different across the different SDI quintiles. In 2019, the numbers of incident and prevalent cases were highest in the middle SDI regions (incidence: 2,496,805.3 [1,769,372.3 to 3,331,789.2]; prevalence: 2,643,172.1 [1,898,580.5 to 3,646,328.3]), but the highest numbers of deaths and DALYs were both observed in the low SDI regions (deaths: 18,662.4 [13,603.5 to 24,697.8]; DALYs: 1,635,176.5 [1,193,681.1 to 2,164,245.2]). Between 1990 and 2019, rising trends in incidence and prevalence were observed in both low-middle and low SDI regions, particularly in the low SDI regions, with a substantial increase of 80.4% (75.3 to 85.3) in incident cases and 81.2% (59.6 to 100.4) in prevalent cases. The other three (high, high-middle, and middle) SDI regions experienced a decline in the numbers of childhood cancer incidence and prevalence. Except for a lack of significant change in the low SDI regions, the percentages of deaths and DALYs in the other four SDI regions during the same period reduced by about half (Table 1 and Figures 3, 4).

At the national or territorial level, China had the largest number of incident cases of total neoplasm, followed by Japan and the Russian Federation in 1990 [Supplementary Table 1 in Appendix 2 (35)]. Despite the numbers have declined by −21.5% (−28.8 to −12.9) between 1990 and 2019, they remained high in China in 2019, with Benin and India having the next-highest numbers. China and India consistently had the highest prevalence of childhood cancer during the 1990 to 2019 period. Ethiopia ranked third in 1990 and Pakistan in 2019. In terms of the absolute number of deaths, the top three countries and territories were China, India, and Ethiopia in 1990. The top two countries with the highest number of total neoplasm deaths remained the same in 2019, but the number of deaths increased by 15.8% (−27.1 to 117.6) from 1990 to 2019; Pakistan ranked third in this category in 2019. China, Japan, and the Russian Federation were the top three countries/territories with the highest number of DALYs in 1990; the rankings in 2019 were China, India, and Japan in the first, second, and third positions, respectively [Supplementary Table 3 in Appendix 2 (35)].

### Leading Causes of Burden

The global estimates of the incident and prevalent cases, deaths, and DALYs by childhood cancer for 1990 and 2019 and the corresponding percentage changes during those time periods are presented in Table 2. Excluding total neoplasms, other neoplasms, and their sub-types, with 59,483.4 (48,774.0 to 72,110.7) incident cases, leukemia accounted for the greatest childhood cancer burden in 2019, followed by brain and central nervous system cancer (18,244.1 [13,713.2 to 22,560.2]) and testicular cancer (17.343.6 [6,925.4 to 30,721.5]). Leukemia was also the most diagnosed incident cancer in 1990, although this number substantially declined (−55.7% [−70.1 to −26.7]) between 1990 and 2019. Of note, the incident cases of testicular cancer increased by 60.0% (5.0 to 287.8) during the same period.

**Table 2:** Global numbers of incidence, prevalence, deaths, and DALYs under 5-year-old by specific childhood neoplasms, 1990–2019.

	**Absolute numbers among children aged under five**		
	**1990**	**2019**	**Percentage change 1990–2019**
**Total neoplasms**			
Incidence	9200909.6 (6495831.8 to 12413218.7)	8774979.1 (6243599.2 to 11737568.5)	−4.6% (−7.0 to −2.2)
Prevalence	9763754.5 (6934825.2 to 13322073.0)	8956583.8 (6446323.9 to 12364520.8)	−8.3% (−12.6 to −3.4)
Deaths	85196.7 (60361.4 to 112686.7)	44451.6 (36198.7 to 53905.9)	−47.8% (−60.7 to −26.4)
DALYs	7487603.1 (5311977.2 to 9900566.2)	3918014.8 (3196454.9 to 4751304.2)	−47.7% (−60.7 to −26.2)
**Liver cancer**			
Incidence	2279.3 (1606.7 to 3255.3)	1251.9 (812.8 to 1796.1)	−45.1% (−65.1 to −20.8)
Prevalence	3988.4 (2821.0 to 5681.9)	2424.9 (1606.9 to 3456.2)	−39.2% (−61.2 to −13.4)
Deaths	3082.9 (2391.8 to 4164.2)	1731.2 (1280.0 to 2244.2)	−43.8% (−64.2 to −20.3)
DALYs	270183.1 (209819.7 to 364476.7)	151482.6 (111939.1 to 196599.2)	−43.9% (−64.3 to −20.3)
**Leukemia**			
Incidence	134237.7 (78876.4 to 194254.2)	59483.4 (48774.0 to 72110.7)	−55.7% (−70.1 to −26.7)
Prevalence	902530.5 (471203.1 to 1305523.6)	386762.4 (317829.6 to 477817.1)	−57.1% (−71.3 to −20.8)
Deaths	40677.3 (25239.6 to 59749.5)	16351.1 (13135.7 to 20114.5)	−59.8% (−72.3 to −35.2)
DALYs	3600795.8 (2238455.8 to 5275293.4)	1451022.0 (1165442.3 to 1788374.2)	−59.7% (−72.3 to −35.2)
**Brain and nervous system cancers**			
Incidence	21352.0 (11934.8 to 37856.4)	18244.1 (13713.2 to 22560.2)	−14.6% (−57.1 to 50.3)
Prevalence	69923.3 (42665.0 to 116839.3)	82226.8 (61465.4 to 99430.9)	17.6% (−44.4 to 96.1)
Deaths	13510.1 (7200.6 to 24508.6)	8530.4 (6413.9 to 10620.9)	−36.9% (−66.9 to 11.6)
DALYs	1179727.4 (628836.5 to 2135148.2)	747940.7 (563194 to 929664.2)	−36.6% (−66.8 to 12.1)
**Malignant skin melanoma**			
Incidence	854.3 (228.9 to 1858.7)	1038.7 (394.9 to 2565.7)	21.6% (−10.8 to106.8)
Prevalence	6434.0 (1597.2 to 15452.5)	8615.4 (3115.3 to 22395.0)	33.9% (0.2 to 132.5)
Deaths	185.7 (65.0 to 445.6)	141.4 (72.8 to 291.6)	−23.8% (−48.2 to 31.4)
DALYs	16447.0 (5733.3 to 38958.2)	12734.0 (6476.0 to 26102.3)	−22.6% (−47.7 to33.3)
**Testicular cancer**			
Incidence	10842.3 (2359.8 to 18674.1)	17343.6 (6925.4 to 30721.5)	60.0% (5.0 to 287.8)
Prevalence	68403.5 (8847.6 to 137471.5)	131933.0 (47057.9 to 244771.6)	92.9% (27.7 to 618.3)
Deaths	339.4 (179.8 to 1109.5)	322.8 (209.7 to 717.2)	−4.9% (−41.3 to 57.6)
DALYs	34186.6 (16635.2 to 101720.2)	36290.1 (23748.9 to 70719.9)	6.2% (−37.0 to 74.2)
**Kidney cancer**			
Incidence	7721.9 (5859.0 to 9910.1)	7890.4 (6453.0 to 9400.8)	2.2% (−22.3 to 34.8)
Prevalence	50541.9 (39437.6 to 64814.4)	55564.3 (46058.0 to 65914.2)	9.9% (−16.8 to 43.8)
Deaths	2280.5 (1587.4 to 2992.0)	1755.2 (1342.6 to 2227.1)	−23.0% (−40.3 to 2.9)
DALYs	201185.9 (139974.2 to 263728.5)	155753.9 (119112.4 to 197182.9)	−22.6% (−40.0 to 3.4)
**Hodgkin lymphoma**			
Incidence	818.1 (359.2 to 1172.7)	558.1 (417.8 to 708.5)	−31.8% (−50.1 to 34.9)
Prevalence	3832.9 (1970.9 to 5234.3)	3824.2 (3105.7 to 4602.4)	−0.2% (−28.2 to 83.8)
Deaths	411.5 (142.8 to 617.5)	150.0 (83.3 to 226.1)	−63.6% (−73.4 to −36.0)
DALYs	35717.4 (12444.5 to 53563.4)	13156.2 (7363.2 to 19717.5)	−63.2% (−73.1 to −34.8)
**Non–Hodgkin lymphoma**			
Incidence	0.0 (0.0 to 0.0)	0.0 (0.0 to 0.0)	0.0% (0.0 to 0.0)
Prevalence	0.0 (0.0 to 0.0)	0.0 (0.0 to 0.0)	0.0% (0.0 to 0.0)
Deaths	3546.6 (2030.4 to 4831.4)	1919.7 (1514.3 to 2369.2)	−45.9% (−61.2 to −9.0)
DALYs	305107.4 (174663.4 to 415641.0)	165134.3 (130258.6 to 203800.4)	−45.9% (−61.2 to −9.0)
**Other malignant neoplasms**			
Incidence	20494.1 (17059.3 to 24912.2)	19029.4 (16160.6 to 22410.6)	−7.1% (−28.0 to 18.6)
Prevalence	95763.3 (81912.2 to 112683.4)	109473.4 (92896.4 to 129244.9)	14.3% (−11.0 to 45.2)
Deaths	20808.7 (16067.1 to 26962.6)	13297.2 (10223.2 to 17044)	−36.1% (−48.6 to −18.6)
DALYs	1812053.2 (1401268.2 to 2344473.4)	1161199.8 (894574.6 to 1484588.5)	−35.9% (−48.5 to −18.4)
**Other neoplasms**			
Incidence	9002310.1 (6328428.5 to 12233793.2)	8650139.5 (6123797.2 to 11626471.1)	−3.9% (−6.5 to −1.4)
Prevalence	8765530.5 (5977365.2 to 12307007.5)	8359796.4 (5823844.9 to 11694827.4)	−4.6% (−7.6 to −1.4)
Deaths	354.0 (237.5 to 607.8)	252.6 (203.3 to 317.4)	−28.7% (−55.1 to 9.2)
DALYs	32199.3 (21887.4 to 54727.9)	23301.3 (18919.8 to 28932.3)	−27.6% (−53.7 to 8.4)
**Myelodysplastic, myeloproliferative, and other hematopoietic neoplasms**			
Incidence	17500.0 (11031.5 to 25759.4)	19253.9 (12260.8 to 28111.8)	10.0% (4.7 to 15.8)
Prevalence	28394.4 (17920.9 to 42311.8)	27191.5 (17457.8 to 40635.7)	−4.2% (−8.2 to 0.9)
Deaths	354.0 (237.5 to 607.8)	252.6 (203.3 to 317.4)	−28.7% (−55.1 to 9.2)
DALYs	32199.3 (21887.4 to 54727.9)	23301.3 (18919.8 to 28932.3)	−27.6% (−53.7 to 8.4)
**Benign and** ***in situ*** **intestinal neoplasms**			
Incidence	14225.0 (7327.7 to 22773.2)	14039.8 (7556.7 to 22498.7)	−1.3% (−5.5 to 4.4)
Prevalence	15684.6 (8308.3 to 26004.8)	15668.5 (8644.4 to 25833.2)	−0.1% (−4.3 to 6.3)
Deaths	NA	NA	NA
DALYs	0.0 (0.0 to 0.0)	0.0 (0.0 to 0.0)	NA
**Benign and** ***in situ*** **cervical and uterine neoplasms**			
Incidence	4806.1 (2249.0 to 8200.0)	4620.8 (2153.0 to 7918.5)	−3.9% (−9.8 to 0.6)
Prevalence	5402.7 (2427.7 to 9450.4)	5254.7 (2316.5 to 9264.5)	−2.7% (−8.8 to 1.9)
Deaths	NA	NA	NA
DALYs	0.0 (0.0 to 0.0)	0.0 (0.0 to 0.0)	NA
**Other benign and** ***in situ*** **neoplasms**			
Incidence	8965779.0 (6283414.0 to 12205020.5)	8612225.0 (6096794.4 to 11592099.3)	−3.9% (−6.5 to −1.4)
Prevalence	8753466.9 (5963635.7 to 12296861.6)	8351045.0 (5835295.3 to 11709053.4)	−4.6% (−7.6 to −1.6)
Deaths	NA	NA	NA
DALYs	0.0 (0.0 to 0.0)	0.0 (0.0 to 0.0)	NA

*Data in parentheses are 95% uncertainty intervals (UIs) unless otherwise stated*.

*DALYs, disability-adjusted life-years. NA, not available*.

Although the number of prevalent cases decreased by −57.1% (−71.3 to −20.8) between 1990 and 2019, leukemia was the leading childhood cancer with the most prevalent cases in 1990 (902,530.5 [471,203.1 to 1,305,523.6]) and 2019 (386,762.4 [317,829.6 to 477,817.1]). The number of prevalent cases of testicular cancer increased substantially by 92.9% (27.7 to 618.3) during the same period, ranking it second (131,933.0 [47,057.9 to 244,771.6]) in 2019. Brain and central nervous system cancer was the childhood cancer type with the third-largest number of prevalent cases (82,226.8 [61,465.4 to 99,430.9]) in 2019 [Table 2 and Supplementary Figure 2 in Appendix 2 (35)].

The three leading causes of cancer death among children under 5 years were leukemia (16,351.1 [13,135.7 to 20,114.5]), brain and central nervous system cancer (85,30.4 [6,413.9 to 10,620.9]), and non-Hodgkin's lymphoma (1,919.7 [1,514.3 to 2,369.2]) in 2019. A substantial decline was observed for both leukemia (−59.8% [−72.3to −35.2]) and non-Hodgkin's lymphoma (−45.9% [−61.2 to −9.0]) from 1990 to 2019 [Table 2 and Supplementary Figure 3 in Appendix 2 (35)].

In 2019, the heaviest burden of DALYs (1,451,022.0 [1,165,442.3 to 1,788,374.2]) was attributed to leukemia, followed by brain and central nervous system cancer (747,940.7 [563,194 to 929,664.2]) and non-Hodgkin's lymphoma (165,134.3 [130,258.6 to 203,800.4]). A declining trend in DALYs was observed for both leukemia (−59.7% [−72.3 to −35.2]) and non-Hodgkin's lymphoma (−45.9% [−61.2 to −9.0]) between 1990 and 2019 [Table 2 and Supplementary Figures 3–6 in Appendix 2 (35)].

### Top 10 Countries and Territories With the Highest Incidence and DALYs of Specific Cancer

In 2019, China had the highest number of incident cases and DALYs of total neoplasms, liver cancer, leukemia, and other neoplasms. The highest number of incident cases of brain and nervous system cancers, testicular cancer, kidney cancer, Hodgkin's lymphoma, and other malignant neoplasms were also observed in China. India had the highest DALYs for brain and nervous system cancers and testicular cancer, whereas Nigeria bore the heaviest burden of DALYs for kidney cancer and both Hodgkin's and non-Hodgkin's lymphoma. The United States of America ranked first for both incident cases and DALYs of malignant skin melanoma. The countries with the highest incidence rates were largely located in Europe and Asia, whereas those with the highest U5MR due to all cancers were mostly in Africa and the Caribbean [Supplementary Tables 4–6 in Appendix 2 (35)].

## Discussion

Using up-to-date data from the GBD 2019, we are the first to produce global estimates of cancer burden in children younger than 5 years and to explore trends of cancers in this age group across 204 countries and territories for the 1990 to 2019 period. As measured by changes in four indicators (incidence, prevalence, deaths, and DALYs), we found that the global burden of childhood cancers has remained considerably high over the past 30 years, particularly in low-middle SDI countries. Despite the global childhood cancer burden having declined during our study period, especially in terms of mortality and DALYs, the reduction in cancer burden varied widely across countries. In addition, the incidence and prevalence have increased in low-middle and low SDI countries since 1990. Our findings indicate that the progress in public regulatory policies and community programs on childhood cancer prevention are not sufficiently effective, emphasizing the need for country-level assessments and country-specific prevention plans.

Previous work based on the GBD 2017 and 2019 has estimated the global burden of cancer in children and adolescents (aged 0–19 years) (23) and adolescents and young adults (aged 15–39 years)(24), respectively. These studies have reported disproportionately high DALYs attributable to childhood cancers in resource-limited settings, with the greatest DALY burden for the majority of childhood cancers of their interest being the highest in low-middle SDI regions in 2017 (23) and middle SDI regions in 2019 (24). However, the present study indicated that the highest DALYs due to cancer in children younger than 5 years occurred in low SDI regions in 2019. The heavier burden in low SDI regions in our study can be explained by the gap between the unmet need for pediatric healthcare for cancer patients under 5 years due to their immature immune systems, and the scarce health infrastructures and inadequately skilled workforce for the care of critically ill children in LMICs (36). Of note, because different amounts of data were available for different cancers between age groups in the GBD datasets (37), the inevitable heterogeneity in the burden of childhood cancers in children and adolescents (aged 0–19 years) and adolescents and young adults (aged 15–39 years) could not be ignored in the older studies (23, 24). Furthermore, without exploring the secular trends of the burden over the past few years, their studies (23, 24) could not identify areas of progress in childhood cancer burden and areas in which cases current efforts are inadequate.

Notably, consistent with our study, the GBD 2017 (23) reported that the incidence of total childhood cancers showed an upward trend with the growth of SDIs; this epidemiology transition revealed that the GBD 2019 was further standardized and that the methods were revised to enhance the stability of data over different GBD cycles (26). The increased incidence rate of total neoplasms in HICs is attributable to the development of screening tests and early cancer detection in regions with effective healthcare systems (38), which could lead to over-diagnosis. Children with cancers in HICs benefit from the availability of well-developed and accessible medical and social infrastructure, whereas their counterparts in LMICs have limited access to diagnosis and treatments. In addition, the validity of diagnosis and laboratory investigations differs between developed and less-developed countries, potentially affecting data accuracy and the quality of the cancer registries and resulting in substantial heterogeneity in cancer incidence across countries or regions at various socioeconomic development levels (39, 40). Therefore, the development and implementation of effective policies to improve frail healthcare systems and address the inequality in access to healthcare are of paramount importance in LMICs.

As the average SDI across the world has steadily improved over the last five decades, less-developed countries have made faster progress in terms of SDI over the last 20 years (41). Substantial reductions in mortality and DALYs in different countries across the SDI spectrum suggest that improvements in healthcare have largely occurred due to overall socioeconomic improvements. The improvements in education level, gross domestic product per capita, and access to modern contraceptives are potential contributors to the accelerated progress in global health (41, 42); in addition, substantial improvements to primary healthcare systems have been made in LMICs (43). Consequently, the increases in childhood cancer in resource-limited settings may be related to the development and widespread application of new diagnostic capabilities or differential access to diagnostic investigations, rather than to a true change in the incidence of malignant disease (38, 41). Moreover, better prognoses for childhood cancer patients due to improved treatments have increased the prevalence of childhood cancers and the number of childhood cancer survivors (44).

China and India, as the largest and most populous LMICs, bore the heaviest burden of childhood cancers in 2019. The top 10 leading countries with a heavy burden of total childhood cancers were predominantly Sub-Saharan African and under-developed Asian countries. With about 85% of the global population living in LMICs, approximately 80% (240,000) of children in LMICs are newly diagnosed with cancers every year (39). The large proportion of younger populations in low-income countries might be associated with an increased risk of childhood cancers (39); this can be partly explained by the increased risk of susceptibility genes for cancer being inherited by larger families (45). A previous study showed that 29% of children were at risk for hereditary cancer due to their families' cancer histories (46). Moreover, the large household sizes in less-developed countries, reflecting the caregivers' limited time and heavy physical and emotional burdens of childcare, could delay the detection of early signs and symptoms of cancer and result in inadequate and low-quality care for sick children (47); this cumulatively leads to the poor survival of children with childhood cancers. As an ever-increasing proportion of the global birth cohort resides in Sub-Saharan Africa (48), accelerating the global pace of reducing the childhood cancer burden remains a challenging task.

Consistent with a previous study (49), leukemia was the most diagnosed cancer in children under 5 years in 2019; this considerable burden is attributable to genetic and environmental factors. Low-dose fetal exposure to ionizing radiation from antenatal X-rays can increase the absolute risk of cancer or leukemia (50). In addition, because of their immature immune systems, children under 5 years face a time window of increased susceptibility to air pollutants (51). A previous study reported a strong association between traffic-related pollution and leukemia in children below 5 years, particularly for ALL (51); a similar association between traffic-related benzene exposure and acute myeloblastic leukemia has also been reported (52), indicating the carcinogenic effects of traffic emissions. Although a growing body of literature has addressed the wide range of risk factors of leukemia, further high-quality studies are needed to clarify the etiology of childhood leukemia.

Our results point to a need to revisit childhood cancer control policies and health programs and emphasize a greater understanding of country-specific health priorities and program implementations to alleviate the burden of childhood cancer in resource-limited countries. As part of the SDG-3, the United Nations has featured U5MR control as a key component of sustainable development in the 2030 agenda (3). Nevertheless, numerous implementation challenges exist, particularly in less-developed countries. The cancer burden and its trends over the past three decades can not only be used by government and international agencies to guide policy agendas and program implementations to reduce global or national U5MRs due to cancer but also help identify regions or countries that require more medical attention to alleviate the global childhood cancer burden.

### Limitations

This study has several limitations. First, the accuracy and robustness of the GBD estimate largely rely on the availability and quality of the data. The completeness estimate for sample registration systems was affected by the sparsity of census data in different regions and the lag between censuses (41). Therefore, population coverage and quality of vital status registration data or cancer registries might vary substantially across countries along the sociodemographic spectrum. Similarly, the potential variability and inconsistency of vital status registration and cancer registry data across countries might influence geographic variations and temporal trends of childhood cancer. Therefore, it is crucial to develop standards and quality control for data collection by expanding or creating more systematic vital registration systems and cancer registries, thereby improving data quality via enhanced diagnostic facilities in low-resource countries. Second, the present anatomical site-based system for cancer reporting works well for adult cancers, but not for certain childhood cancers, which are labeled as “uncategorized cancers” (23). Several cancers that are common in the pediatric population but rare in adults have been categorized into the “other neoplasms” group. This might have increased the heterogeneity of cancers in this category, making it difficult to precisely estimate the burden of childhood cancer in this age group. Third, despite the comprehensive nature of the GBD dataset, data on certain childhood cancers from some regions or countries might be sparse or even absent. For example, the GBD 2019 did not report data for chronic lymphocytic leukemia in children younger than 5 years, as this sub-type of leukemia mostly affects adults (53); the study also lacked sufficient data for non-Hodgkin's lymphoma. For this reason, we were unable to accurately quantify the total number of children with leukemia or compare all incident cases between the cancers of interest in this study. Fourth, the GBD 2019 data were aggregated, with no differentiation in terms of tumor grades by histology. This limited further explorations of the severity of childhood cancers by location or over time. With the increased availability of diagnostic tools and continuous improvements in cancer registry data quality, annual updating of the GBD database will allow us to refine the methodology and more accurately estimate the childhood cancer burden.

## Conclusion

In conclusion, we found that the burden of childhood cancer in children under 5 years has been declining globally. However, the overall burden was remarkably high in 2019. Although the cancer burden reduced across different regions, LMICs bore the heaviest burden of childhood cancer; the incidence and prevalence of childhood cancers even increased over the past 30 years in these countries. Further work is required to understand childhood health needs in these situations. In this study, we highlight the urgent need for a concerted global initiative and effective policies and interventions to reduce the cancer burden in children in their first 5 years of life in these countries. It is of great importance for policymakers and healthcare providers to address the issues of inequitable healthcare access, further reduce the burden of childhood cancer, and decrease U5MRs to reach the SDG-3 target by 2030.

## Data Availability Statement

Publicly available datasets were analyzed in this study. This data can be found here: http://ghdx.healthdata.org/gbd-results-tool.

## Author Contributions

X-FS and L-YH developed the concept and designed the study. H-MR and M-QL drafted the original manuscript. S-XT, CC, SZ, LZ, J-RM, and Y-JM prepared the tables and figures. W-LL, S-WZ, R-QOY, S-NL, Y-FC, X-YK, and Z-YL contributed to data preparation and interpreted the results. JL, L-PL, X-FL, and F-FZ completed the scoping review. F-FZ, M-QL, and L-YH got the fundings. All other reviewed results, contributed equally to the paper, and approved the final version of the manuscript.

## Funding

This study was funded by the National Natural Science Foundation of China (Grant No. 81602853), the China Scholarship Council (File No. 202106780004), and the Ningbo Key Support Medical Discipline (Grant No. 2022-F22).

## Conflict of Interest

The authors declare that the research was conducted in the absence of any commercial or financial relationships that could be construed as a potential conflict of interest.

## Publisher's Note

All claims expressed in this article are solely those of the authors and do not necessarily represent those of their affiliated organizations, or those of the publisher, the editors and the reviewers. Any product that may be evaluated in this article, or claim that may be made by its manufacturer, is not guaranteed or endorsed by the publisher.
