# Supplementary material for: Global, Regional, and National Burden of Cancer in Children Younger Than 5 Years, 1990–2019: Analysis of the Global Burden of Disease Study 2019
Source: Front Public Health. 2022 Jun 21;10:910641. doi: 10.3389/fpubh.2022.910641 (PMC9255714; doi:10.3389/fpubh.2022.910641)
Supplement: Supplementary file 1 [file Data_Sheet_1.docx]

**Appendix 1**

**Global, regional, and national burden of cancer in children younger than 5 years, 1990–2019: Analysis of the Global Burden of Disease Study 2019**

**Table legends**

**Supplementary Table 1.** The Guidelines for Accurate and Transparent Health Estimates Reporting (GATHER) checklist of information that should be included in reports of global health estimates.

**Supplementary Table 2.** List of International Classification of Diseases (ICD) codes mapped to the Global Burden of Disease cause list.

**Supplementary Table 3.** Metric definitions.

**Supplementary Table 1.** The Guidelines for Accurate and Transparent Health Estimates Reporting (GATHER) checklist of information that should be included in reports of global health estimates.

| **Item number** | **Checklist item** |
| --- | --- |
| **Objectives and funding** | |
| 1 | Define the indicator(s), populations (including age, sex, and geographic entities), and time period(s) for which estimates were made. |
| 2 | List the funding sources for the work. |
| **Data inputs** | |
| For all data inputs from multiple sources that are synthesised as part of the study: | |
| 3 | Describe how the data were identified and how the data were accessed. |
| 4 | Specify the inclusion and exclusion criteria. Identify all ad-hoc exclusions. |
| 5 | Provide information about all included data sources and their main characteristics. For each data source used, report reference information or contact name/institution, population represented, data collection method, year(s) of data collection, sex and age range, diagnostic criteria or measurement method, and sample size, as relevant. |
| 6 | Identify and describe any categories of input data that have potentially important biases (eg, based on characteristics listed in item 5). |
| For data inputs that contribute to the analysis but were not synthesised as part of the study: | |
| 7 | Describe and give sources for any other data inputs. |
| For all data inputs: | |
| 8 | Provide all data inputs in a file format from which data can be efficiently extracted (eg, a spreadsheet rather than a PDF), including all relevant meta-data listed in item 5. For any data inputs that cannot be shared because of ethical or legal reasons, such as third-party ownership, provide a contact name or the name of the institution that retains the right to the data. |
| **Data analysis** | |
| 9 | Provide a conceptual overview of the data analysis method. A diagram may be helpful. |
| 10 | Provide a detailed description of all steps of the analysis, including mathematical formulae. This description should cover, as relevant, data cleaning, data pre-processing, data adjustments and weighting of data sources, and mathematical or statistical model(s). |
| 11 | Describe how candidate models were evaluated and how the final model(s) were selected. |
| 12 | Provide the results of an evaluation of model performance, if done, as well as the results of any relevant sensitivity analysis. |
| 13 | Describe methods of calculating uncertainty of the estimates. State which sources of uncertainty were, and were not, accounted for in the uncertainty analysis. |
| 14 | State how analytical or statistical source code used to generate estimates can be accessed. |
| **Results and discussion** | |
| 15 | Provide published estimates in a file format from which data can be efficiently extracted. |
| 16 | Report a quantitative measure of the uncertainty of the estimates (eg, uncertainty intervals). |
| 17 | Interpret results in light of existing evidence. If updating a previous set of estimates, describe the reasons for changes in estimates. |
| 18 | Discuss limitations of the estimates. Include a discussion of any modelling assumptions or data limitations that affect interpretation of the estimates. |

**NOTE:**

GATHER covers reporting of studies that disaggregate disease and injuries by underlying cause as defined by a classification system such as the International Classification of Disease (ICD) as well as those that attribute disease and injury to their determinants.

**Supplementary Table 2.** List of International Classification of Diseases (ICD) codes mapped to the Global Burden of Disease cause list

|  | **Cause** | **ICD10** | **ICD10 Used in Hospital/Claims Analyses** | **ICD9** | **ICD9 Used in Hospital/Claims Analyses** |
| --- | --- | --- | --- | --- | --- |
| 100 | Liver cancer | C22-C22.4, C22.7-C22.9, Z85.05 |  | 155-155.9, V10.07 |  |
| 110 | Malignant skin melanoma | C43-C43.9, Z85.82-Z85.828 |  | 172-172.9 |  |
| 119 | Testicular cancer | C62-C62.92, Z80.43, Z85.47-Z85.48 |  | 186-186.9, V10.47-V10.48, V16.43 |  |
| 120 | Kidney cancer | C64-C64.2, C64.9-C65.9, Z80.51, Z85.52-Z85.54 |  | 189-189.1, 189.5-189.6, 209.24 |  |
| 122 | Brain and central nervous system cancer | C70-C70.1, C70.9-C72.9, Z85.841-Z85.848, Z86.011 |  | 191-191.9 |  |
| 125 | Hodgkin lymphoma | C81-C81.49, C81.7-C81.79, C81.9-C81.99, Z85.71-Z85.72 |  | 201-201.98, V10.72 |  |
| 126 | Non-Hodgkin lymphoma | C82-C85.29, C85.7-C86.6, C96-C96.9 |  | 200-200.9, 202-202.98 |  |
| 128 | Leukemia | C91-C93.7, C93.9-C95.2, C95.7-C95.92, Z80.6, Z85.6 |  | 204-208.92, V10.59-V10.69, V16.6 |  |
| 129 | Acute lymphoid leukemia | C91.0-C91.02, C91.2-C91.32, C91.6-C91.62 |  | 204.0-204.02 |  |
| 130 | Chronic lymphoid leukemia |  |  |  |  |
| 131 | Acute myeloid leukemia | C92.0-C92.02, C92.3-C92.62, C93.0-C93.02, C94.0-C94.02, C94.2-C94.22, C94.4-C94.5 |  | 205.0-205.02, 205.2-205.32, 206.0-206.02, 207.0-207.02, 207.2-207.82 |  |
| 132 | Chronic myeloid leukemia | C92.1-C92.22 |  | 205.1-205.12, 207.1 |  |
| 133 | Other leukemia |  |  |  |  |
| 134 | Other malignant neoplasms |  |  |  |  |
| 135 | Other neoplasms | C75.90-C75.92, D00-D24.9, D26.0-D39.9, D4-D49.9, E34.0, K51.4-K51.419, K62.0-K62.3, K63.5, N60-N60.99, N84.0-N84.1, N87-N87.9 | C75.90-C75.92, D00-D24.9, D26.0-D39.9, D4-D49.9, E34.0, K51.4-K51.419, K62.0-K62.3, K63.5, N60-N60.99, N84.0-N84.1, N87-N87.9 | 209.4-209.57, 209.61, 209.63-209.67, 210.0-217.8, 219-237.6, 237.70-237.72, 237.9-239.9, 569.0, 610-610.9, 622.1-622.2, 622.7 | 209.4-217.8, 219-237.6, 237.70-237.72, 237.9-239.9, 569.0, 610-610.9, 622.1-622.2, 622.7 |

**Supplementary Table 3.** Metric definitions

| **Measure** | **Number** | **Percent** | **Rate** | **Years** | **Probability of death** |
| --- | --- | --- | --- | --- | --- |
| **Deaths** | Number of deaths in the population | Proportion of deaths for a particular cause relative to deaths from all causes | Deaths per 100,000 population | n/a | n/a |
| **Disability adjusted life years (DALYs)** | Number of DALYs in the population | Proportion of DALYs for a particular cause relative to DALYs for all causes | DALYs per 100,000 population | n/a | n/a |
| **Years of life lost (YLLs)** | Number of YLLs in the population | Proportion of YLLs for a particular cause relative to YLDs for all causes | YLLs per 100,000 population | n/a | n/a |
| **Years lived with disability (YLDs)** | Number of YLDs in the population | Proportion of YLDs for a particular cause relative to YLDs for all causes | YLDs per 100,000 population | n/a | n/a |
| **Prevalence** | Total number of cases in the population | Proportion of total cases of a particular cause relative to cases from all causes | Total cases per 100,000 population | n/a | n/a |
| **Incidence** | Number new of cases in the population | Proportion of news cases of a particular cause relative to cases from all causes | New cases per 100,000 population | n/a | n/a |

**NOTE:**  These apply only to measures in the GBD Results Tool and GBD Compare.
