# Supplementary material for: Global, Regional, and National Burden of Cancer in Children Younger Than 5 Years, 1990–2019: Analysis of the Global Burden of Disease Study 2019
Source: Front Public Health. 2022 Jun 21;10:910641. doi: 10.3389/fpubh.2022.910641 (PMC9255714; doi:10.3389/fpubh.2022.910641)
Supplement: Supplementary file 2 [file Data_Sheet_2.docx]

**Appendix 2**

**Global, regional, and national burden of cancer in children younger than 5 years, 1990–2019: Analysis of the Global Burden of Disease Study 2019**

**Table legends**

**Supplementary Table 1.** Global and GBD super-regional incidence cases and DALYs of specific childhood neoplasms among children under 5-year-old in 2019.

**Supplementary Table 2.** Global and GBD super-regional incidence cases and DALYs of the subtypes of leukemia and other neoplasms among children under 5-year-old in 2019.

**Supplementary Table 3.** Incident cases, deaths, and DALYs of total neoplasms and their chances in children under 5-year-old by GBD regions, countries and territories in 1990 and 2019.

**Supplementary Table 4.** Top ten countries and territories with specific cancers in children under 5-year-old in 2019.

**Supplementary Table 5.** Incidence cases and DALYs of specific cancers among children under 5-year-old globally, and by SDI regions, GBD super-regions, GBD regions, countries and territories in 2019.

**Supplementary Table 6.** Incidences and DALYs of total and subtypes of leukemia and other neoplasms among children under 5-year-old globally, and by SDI regions, GBD super-regions, GBD regions, countries and territories in 2019.

**Figure legends**

**Supplementary Figure 1.** Percentage changes in absolute numbers of incidence, prevalence, deaths and DALYs of total childhood cancers globally and for 7 GBD super regions between 1990 and 2019.

DALY=disability-adjusted life-years. GBD=Global Burden of Diseases, Injuries and Risk Factors Study.

**Supplementary Figure 2.** Leading 9 childhood cancers of global prevalence for 1990 and 2019, with changes of absolute number and rates for both sexes combined. Cancers are ranked by number of prevalence in 1990 and 2019.

The “Non-Hodgkin lymphoma” group is not included in these data because there is no data in this age group available in the result tool of GBD. GBD=Global Burden of Diseases, Injuries, and Risk Factors Study.

**Supplementary Figure 3.** Relative proportion of deaths (A) and DALYs (B) of each childhood cancers genotype. Size of pie charts is proportional to the number of deaths and DALY of total childhood cancers.

**Supplementary Figure 4.** Childhood cancers ranked by number of DALYs in both sexes, global and by 21 GBD regions in 2019.

Causes in the figure are ordered according to ranks for absolute number of DALYs. Ranks are also color shaded to indicate rank. DALY=disability-adjusted life-years. GBD=Global Burden of Diseases, Injuries, and Risk Factors Study.

**Supplementary Figure 5.** Global DALYs for 10 childhood cancers between 1990 and 2019. DALY=disability-adjusted life-years.

**Supplementary Figure 6.** Childhood cancers ranked globally and for both sexes by absolute DALYs, with changes of absolute number and rates for both sexes combined between 1990 and 2019. Cancers are ranked by number of DALYs in 1990 and 2019. DALY=disability-adjusted life-years.

**Supplementary Table 1.** Global and GBD super-regional incidence cases and DALYs of specific childhood neoplasms among children under 5-year-old in 2019.

|  | Total neoplasms | |  | Liver cancer | |  | Leukemia | |  | Brain and central nervous system cancer | |  | Malignant skin melanoma | |  | Testicular cancer | |
| --- | --- | --- | --- | --- | --- | --- | --- | --- | --- | --- | --- | --- | --- | --- | --- | --- | --- |
|  | Cases | DALYs |  | Cases | DALYs |  | Cases | DALYs |  | Cases | DALYs |  | Cases | DALYs |  | Cases | DALYs |
| **Global** |  |  |  |  |  |  |  |  |  |  |  |  |  |  |  |  |  |
| Absolute number | 8774979·1 (6243599·2 to 11737568·5) | 3918014·8 (3196454·9 to 4751304·2) |  | 1251·9 (812·8 to 1796·1) | 151482·6 (111939·1 to 196599·2) |  | 59483·4 (48774·0 to 72110·7) | 1451022·0 (1165442·3 to 1788374·2) |  | 18244·1 (13713·2 to 22560·2) | 747940·7 (563194·0 to 929664·2) |  | 1038·7 (394·9 to 2565·7) | 12734·0 (6476·0 to 26102·3) |  | 17343·6 (6925·4 to 30721·5) | 36290·1 (23748·9 to 70719·9) |
| Rates† | 1323·8 (941·9 to 1770·8) | 591·1 (482·2 to 716·8) |  | 0·2 (0·1 to 0·3) | 22·9 (16·9 to 29·7) |  | 9·0 (7·4 to 10·9) | 218·9 (175·8 to 269·8) |  | 2·8 (2·1 to 3·4) | 112·8 (85·0 to 140·3) |  | 0·2 (0·1 to 0·4) | 1·9 (1·0 to 3·9) |  | 2·6 (1·0 to 4·6) | 5·5 (3·6 to 10·7) |
| **Central Europe, Eastern Europe, and Central Asia** |  |  |  |  |  |  |  |  |  |  |  |  |  |  |  |  |  |
| Absolute number | 1273706·4 (915288·2 to 1713841·9) | 113749·4 (95422·0 to 135437·4) |  | 28·8 (17·2 to 46·2) | 3902·8 (2624·7 to 5941·1) |  | 1409·6 (1121·2 to 1735·8) | 34544·3 (28106·0 to 42072·8) |  | 604·1 (458·5 to 742·2) | 31184·0 (23662·7 to 38514·8) |  | 110·7 (24·0 to 291·6) | 1231·2 (304·7 to 3470·4) |  | 1828·2 (107·5 to 4261·5) | 1785·1 (619·8 to 3737·3) |
| Rates† | 4621·4 (3320·9 to 6218·3) | 412·7 (346·2 to 491·4) |  | 0·1 (0·1 to 0·2) | 14·2 (9·5 to 21·6) |  | 5·1 (4·1 to 6·3) | 125·3 (102·0 to 152·7) |  | 2·2 (1·7 to 2·7) | 113·1 (85·9 to 139·7) |  | 0·4 (0·1 to 1·1) | 4·5 (1·1 to 12·6) |  | 6·6 (0·4 to 15·5) | 6·5 (2·2 to 13·6) |
| **High-income** |  |  |  |  |  |  |  |  |  |  |  |  |  |  |  |  |  |
| Absolute number | 1484499·8 (1079750·4 to 1981046·5) | 153223·8 (132536·9 to 174974·7) |  | 85·2 (59·4 to 114·8) | 5265·4 (4102·3 to 6209·3) |  | 3767·1 (3034·5 to 4600·4) | 42066·2 (37097·4 to 47569·5) |  | 1920·2 (1355·2 to 2436·8) | 40015·1 (28725·9 to 46859·6) |  | 575·1 (117·6 to 1870·8) | 2705·4 (603·9 to 8963·1) |  | 5866·4 (231·4 to 14213·1) | 4300·6 (797·0 to 10181·5) |
| Rates† | 2607·0 (1896·2 to 3479·1) | 269·1 (232·8 to 307·3) |  | 0·1 (0·1 to 0·2) | 9·2 (7·2 to 10·9) |  | 6·6 (5·3 to 8·1) | 73·9 (65·1 to 83·5) |  | 3·4 (2·4 to 4·3) | 70·3 (50·4 to 82·3) |  | 1·0 (0·2 to 3·3) | 4·8 (1·1 to 15·7) |  | 10·3 (0·4 to 25·0) | 7·6 (1·4 to 17·9) |
| **Latin America and Caribbean** |  |  |  |  |  |  |  |  |  |  |  |  |  |  |  |  |  |
| Absolute number | 840088·7 (590111·3 to 1139909·5) | 276590·9 (216149·8 to 347827·2) |  | 74·1 (48·5 to 107·8) | 7702·4 (5490·3 to 10478·5) |  | 3450·3 (2649·0 to 4386·3) | 105269·8 (81740·2 to 132126·0) |  | 1261·7 (715·3 to 1725·4) | 53677·4 (31348·0 to 72346·0) |  | 82·0 (30·8 to 204·3) | 1273·5 (526·8 to 3261·2) |  | 1796·5 (243·4 to 4300·9) | 4485·7 (1936·8 to 19001·7) |
| Rates† | 1747·5 (1227·5 to 2371·2) | 575·3 (449·6 to 723·5) |  | 0·2 (0·1 to 0·2) | 16·0 (11·4 to 21·8) |  | 7·2 (5·5 to 9·1) | 219·0 (170·0 to 274·8) |  | 2·6 (1·5 to 3·6) | 111·7 (65·2 to 150·5) |  | 0·2 (0·1 to 0·4) | 2·6 (1·1 to 6·8) |  | 3·7 (0·5 to 8·9) | 9·3 (4·0 to 39·5) |
| **North Africa and Middle East** |  |  |  |  |  |  |  |  |  |  |  |  |  |  |  |  |  |
| Absolute number | 1052383·8 (740809·4 to 1419417·8) | 263794·8 (190532·7 to 341275·5) |  | 83·4 (39·6 to 161·8) | 8943·9 (5556·8 to 15764·5) |  | 4231·9 (2861·1 to 5788·5) | 101006·7 (65086·4 to 140446·7) |  | 1737·4 (1123·4 to 2322·2) | 57666·2 (37139·4 to 77963·3) |  | 32·8 (24·1 to 45·3) | 541·5 (398·7 to 809·6) |  | 1510·8 (829·3 to 2781·5) | 4206·2 (3057·0 to 5785·0) |
| Rates† | 1762·2 (1240·5 to 2376·8) | 441·7 (319·0 to 571·5) |  | 0·1 (0·1 to 0·3) | 15·0 (9·3 to 26·4) |  | 7·1 (4·8 to 9·7) | 169·1 (109·0 to 235·2) |  | 2·9 (1·9 to 3·9) | 96·6 (62·2 to 130·5) |  | 0·1 (0·0 to 0·1) | 0·9 (0·7 to 1·4) |  | 2·5 (1·4 to 4·7) | 7·0 (5·1 to 9·7) |
| **South Asia** |  |  |  |  |  |  |  |  |  |  |  |  |  |  |  |  |  |
| Absolute number | 801216·1 (547095·2 to 1103573·7) | 814344·1 (617468·4 to 1030524·1) |  | 185·6 (126·1 to 263·3) | 26123·4 (19937·7 to 33939·8) |  | 8027·1 (4674·7 to 11814·4) | 230564·4 (147862·6 to 326349·7) |  | 246919·9 (174764·4 to 324813·3) | 5326·1 (3713·9 to 7258·5) |  | 49·9 (34·8 to 78·9) | 1417·9 (1024·0 to 2279·7) |  | 1677·0 (1035·3 to 3091·2) | 7708·7 (5623·5 to 10463·6) |
| Rates† | 487·3 (332·8 to 6 71·3) | 495·3 (375·6 to 626·8) |  | 0·1 (0· 1to 0·2) | 15·9 (12·1 to 20·6) |  | 4·9 (2·8 to 7·2) | 140·2 (89·9 to 198·5) |  | 150·2 (106·3 to 197·6) | 3·2 (2·3 to 4·4) |  | 0·0 (0·0 to 0·0) | 0·9 (0·6 to 1·4) |  | 1·0 (0·6 to 1·9) | 4·7 (3·4 to 6·4) |
| **Southeast Asia, East Asia, and Oceania** |  |  |  |  |  |  |  |  |  |  |  |  |  |  |  |  |  |
| Absolute number | 1804384·6 (1291373·2 to 2449959·8) | 757543·4 (644936·0 to 908437·9) |  | 301·1 (200·9 to 427·4) | 38607·3 (27969·9 to 49438·9) |  | 20719·3 (15989·3 to 27206·4) | 339837·2 (277133·8 to 434783·4) |  | 4395·1 (3143·6 to 5899·7) | 143846·1 (109890·5 to 187521·3) |  | 97·0 (78·5 to 123·9) | 1377·0 (1136·6 to 1745·2) |  | 3468·3 (2368·0 to 4950·2) | 5080·9 (3818·9 to 6655·9) |
| Rates† | 1284·5 (919·3 to 1744·1) | 539·3 (459·1 to 646·7) |  | 0·2 (0·1 to 0·3) | 27·5 (19·9 to 35·2) |  | 14·7 (11·4 to 19·4) | 241·9 (197·3 to 309·5) |  | 3·1 (2·2 to 4·2) | 102·4 (78·2 to 133·5) |  | 0·1 (0·1 to 0·1) | 1·0 (0·8 to 1·2) |  | 2·5 (1·7 to 3·5) | 3·6 (2·7 to 4·7) |
| **Sub-Saharan Africa** |  |  |  |  |  |  |  |  |  |  |  |  |  |  |  |  |  |
| Absolute number | 1518699·6 (1049263·4 to 2107385·1) | 1538768·4 (1112756·2 to 2047771·8) |  | 493·7 (261·2 to 819·1) | 60937·4 (38395·1 to 88576·8) |  | 17878·2 (11500·3 to 24581·6) | 597733·3 (404034·2 to 812758·1) |  | 2999·4 (1939·9 to 4630·4) | 174632·0 (113018·8 to 267890·3) |  | 91·1 (37·6 to 268·8) | 4187·5 (1643·3 to 13398·8) |  | 1196·5 (510·7 to 3750·6) | 8722·9 (4344·4 to 23365·1) |
| Rates† | 916·7 (633·4 to 1272·1) | 928·8 (671·7 to 1236·1) |  | 0·3 (0·2 to 0·5) | 36·8 (23·2 to 53·5) |  | 10·8 (6·9 to 14·8) | 360·8 (243·9 to 490·6) |  | 1·8 (1·2 to 2·8) | 105·4 (68·2 to 161·7) |  | 0·1 (0·0 to 0·2) | 2·5 (1·0 to 8·1) |  | 0·7 (0·3 to 2·3) | 5·3 (2·6 to 14·1) |

Data in parentheses are 95% uncertainty intervals (UIs) unless otherwise stated; † All rates were calculated per 100,000 population.

Abbreviations: Cases = cases of incidence; DALYs = disability-adjusted life-years.

**Supplementary Table 1.** Global and GBD super-regional incidence cases and DALYs of specific childhood neoplasms among children under 5-year-old in 2019 (continue).

|  | **Kidney cancer** | |  | **Hodgkin lymphoma** | |  | **Non-Hodgkin lymphoma** | |  | **Other malignant neoplasms** | |  | **Other neoplasms** | |
| --- | --- | --- | --- | --- | --- | --- | --- | --- | --- | --- | --- | --- | --- | --- |
|  | **Cases** | **DALYs** |  | **Cases** | **DALYs** |  | **Cases** | **DALYs** |  | **Cases** | **DALYs** |  | **Cases** | **DALYs** |
| **Global** |  |  |  |  |  |  |  |  |  |  |  |  |  |  |
| Absolute number | 7890·4 (6453·0 to 9400·8) | 155753·9 (119112·4 to 197182·9) |  | 558·1 (417·8 to 708·5) | 13156·2 (7363·2 to 19717·5) |  | 0·0 (0·0 to 0·0) | 165134·3 (130258·6 to 203800·4) |  | 19029·4 (16160·6 to 22410·6) | 1161199·8 (894574·6 to 1484588·5) |  | 8650139·5 (6123797·2 to 11626471·1) | 23301·3 (18919·8 to 28932·3) |
| Rates† | 1·2 (1·0 to 1·4) | 23·5 (18·0 to 29·7) |  | 0·1 (0·1 to 0·1) | 2·0 (1·1 to 3·0) |  | 0·0 (0·0 to 0·0) | 24·9 (19·7 to 30·7) |  | 2·9 (2·4 to 3·4) | 175·2 (135·0 to 224·0) |  | 1305·0 (923·9 to 1754·0) | 3·5 (2·9 to 4·4) |
| **Central Europe, Eastern Europe, and Central Asia** |  |  |  |  |  |  |  |  |  |  |  |  |  |  |
| Absolute number | 420·8 (342·0 to 500·5) | 6400·8 (5155·7 to 7696·4) |  | 39·6 (25·5 to 50·7) | 455·5 (260·3 to 602·8) |  | 0·0 (0·0 to 0·0) | 4641·9 (3816·0 to 5719·4) |  | 834·3 (603·9 to 1124·0) | 29029·4 (24206·5 to 35130·4) |  | 1268430·3 (908408·5 to 1710560·4) | 574·4 (358·7 to 740·8) |
| Rates† | 1·5 (1·2 to 1·8) | 23·2 (18·7 to 27·9) |  | 0·1 (0·1 to 0·2) | 1·7 (0·9 to 2·2) |  | 0·0 (0·0 to 0·0) | 16·8 (13·8 to 20·8) |  | 3·0 (2·2 to 4·1) | 105·3 (87·8 to 127·5) |  | 4602·3 (3296·0 to 6206·4) | 2·1 (1·3 to 2·7) |
| **High-income** |  |  |  |  |  |  |  |  |  |  |  |  |  |  |
| Absolute number | 708·7 (570·4 to 882·4) | 6321·2 (5490·7 to 7199·7) |  | 113·9 (91·5 to 137·7) | 434·7 (379·2 to 489·1) |  | 0·0 (0·0 to 0·0) | 4404·9 (3856·0 to 5015·6) |  | 2180·4 (1667·7 to 2961·1) | 44608·3 (38978·1 to 51158·6) |  | 1469282·8 (1064903·4 to 1966645·6) | 3102·0 (2201·1 to 3724·3) |
| Rates† | 1·2 (1·0 to 1·5) | 11·1 (9·6 to 12·6) |  | 0·2 (0·2 to 0·2) | 0·8 (0·7 to 0·9) |  | 0·0 (0·0 to 0·0) | 7·7 (6·8 to 8·8) |  | 3·8 (2·9 to 5·2) | 78·3 (68·5 to 89·8) |  | 2580·3 (1870·2 to 3453·8) | 5·4 (3·9 to 6·5) |
| **Latin America and Caribbean** |  |  |  |  |  |  |  |  |  |  |  |  |  |  |
| Absolute number | 863·5 (679·3 to 1102·3) | 15335·8 (12123·6 to 19377·7) |  | 44·5 (32·7 to 61·2) | 793·1 (585·4 to 1058·8) |  | 0·0 (0·0 to 0·0) | 12993·4 (10263·8 to 16305·2) |  | 1532·2 (1095·7 to 2076·9) | 69010·8 (52900·4 to 89828·2) |  | 830984·0 (582041·4 to 1129962·4) | 6049·1 (4072·0 to 8180·7) |
| Rates† | 1·8 (1·4 to 2·3) | 31·9 (25·2 to 40·3) |  | 0·1 (0·1 to 0·1) | 1·6 (1·2 to 2·2) |  | 0·0 (0·0 to 0·0) | 27·0 (21·4 to 33·9) |  | 3·2 (2·3 to 4·3) | 143·6 (110·0 to 186·9) |  | 1728·5 (1210·7 to 2350·5) | 12·6 (8·5 to 17·0) |
| **North Africa and Middle East** |  |  |  |  |  |  |  |  |  |  |  |  |  |  |
| Absolute number | 1008·2 (718·3 to 1338·9) | 11283·4 (8133·3 to 15055·4) |  | 29·1 (13·3 to 47·3) | 542·6 (242·0 to 911·7) |  | 0·0 (0·0 to 0·0) | 9636·4 (6872·7 to 13006·0) |  | 2086·0 (1496·4 to 2716·8) | 68811·6 (54511·3 to 86253·9) |  | 1041664·1 (730141·5 to 1410119·1) | 1156·2 (846·0 to 1517·1) |
| Rates† | 1·7 (1·2 to 2·2) | 18·9 (13·6 to 25·2) |  | 0·0 (0·0 to 0·1) | 0·9 (0·4 to 1·5) |  | 0·0 (0·0 to 0·0) | 16·1 (11·5 to 21·8) |  | 3·5 (2·5 to 4·5) | 115·2 (91·3 to 144·4) |  | 1744·2 (1222·6 to 2361·2) | 1·9 (1·4 to 2·5) |
| **Southeast Asia, East Asia, and Oceania** |  |  |  |  |  |  |  |  |  |  |  |  |  |  |
| Absolute number | 2373·3 (1970·2 to 2843·1) | 35871·3 (29842·8 to 43262·4) |  | 151·6 (116·8 to 212·8) | 1834·3 (1402·3 to 2622·2) |  | 0·0 (0·0 to 0·0) | 33415·5 (26797·0 to 42096·4) |  | 5844·2 (4790·8 to 7118·3) | 151709·8 (128133·1 to 179258·9) |  | 1767034·7 (1255342·3 to 2414618·9) | 5964·1 (4736·0 to 8371·9) |
| Rates† | 1·7 (1·4 to 2·0) | 25·5 (21·2 to 30·8) |  | 0·1 (0·1 to 0·2) | 1·3 (1·0 to 1·9) |  | 0·0 (0·0 to 0·0) | 23·8 (19·1 to 30·0) |  | 4·2 (3·4 to 5·1) | 108·0 (91·2 to 127·6) |  | 1257·9 (893·6 to 1718·9) | 4·2 (3·4 to 6·0) |
| **South Asia** |  |  |  |  |  |  |  |  |  |  |  |  |  |  |
| Absolute number | 534·3 (352·2 to 730·8) | 12073·2 (8161·7 to 16098·1) |  | 59·8 (34·8 to 100·2) | 2017·6 (1187·6 to 3397·7) |  | 0·0 (0·0 to 0·0) | 29138·0 (22019·4 to 37569·9) |  | 2735·7 (1899·8 to 3537·4) | 255026·6 (186541·4 to 331620·9) |  | 782620·7 (528493·6 to 1088888·0) | 3354·4 (2171·4 to 5388·5) |
| Rates† | 0·3 (0·2 to 0·4) | 7·3 (5·0 to 9·8) |  | 0·0 (0·0 to 0·1) | 1·2 (0·7 to 2·1) |  | 0·0 (0·0 to 0·0) | 17·7 (13·4 to 22·9) |  | 1·7 (1·2 to 2·2) | 155·1 (113·5 to 201·7) |  | 476·0 (321·5 to 662·3) | 2·0 (1·3 to 3·3) |
| **Sub-Saharan Africa** |  |  |  |  |  |  |  |  |  |  |  |  |  |  |
| Absolute number | 1981·5 (1217·3 to 2878·1) | 68468·1 (40672·2 to 101514·6) |  | 119·6 (41·7 to 210·4) | 7078·4 (2339·7 to 12662·3) |  | 0·0 (0·0 to 0·0) | 70904·2 (43400·0 to 101875·7) |  | 3816·6 (2836·2 to 5203·3) | 543003·2 (368941·6 to 773276·1) |  | 1490122·9 (1022668·6 to 2080553·2) | 3101·2 (1994·5 to 5777·3) |
| Rates† | 1·2 (0·7 to 1·7) | 41·3 (24·6 to 61·3) |  | 0·1 (0·0 to 0·1) | 4·3 (1·4 to 7·6) |  | 0·0 (0·0 to 0·0) | 42·8 (26·2 to 61·5) |  | 2·3 (1·7 to 3·1) | 327·8 (222·7 to 466·8) |  | 899·5 (617·3 to 1255·9) | 1·9 (1·2 to 3·5) |

Data in parentheses are 95% uncertainty intervals (UIs) unless otherwise stated; † All rates were calculated per 100,000 population.

Abbreviations: Cases = cases of incidence; DALYs = disability-adjusted life-years.

**Supplementary Table 2.** Global and GBD super-regional incidence cases and DALYs of the subtypes of leukemia and other neoplasms among children under 5-year-old in 2019.

|  | **Acute lymphoid leukemia** | |  | **Acute myeloid leukemia** | |  | **Other leukemia** | |  | **Myelodysplastic, myeloproliferative, and other hematopoietic neoplasms** | |  | **Benign and in situ intestinal neoplasms** | |  | **Benign and in situ cervical and uterine neoplasms** | |  | **Other benign and in situ neoplasms** | |
| --- | --- | --- | --- | --- | --- | --- | --- | --- | --- | --- | --- | --- | --- | --- | --- | --- | --- | --- | --- | --- |
|  | **Cases** | **DALYs** |  | **Cases** | **DALYs** |  | **Cases** | **DALYs** |  | **Cases** | **DALYs** |  | **Cases** | **DALYs** |  | **Cases** | **DALYs** |  | **Cases** | **DALYs** |
| **Global** |  |  |  |  |  |  |  |  |  |  |  |  |  |  |  |  |  |  |  |  |
| Absolute number | 14839·6 (11460·4 to 18535·3) | 520731·5 (367311·6 to 678587·9) |  | 6617·9 (5069·7 to 8500·3) | 313264·2 (241310·9 to 398865·5) |  | 35823·9 (28358·0 to 45633·2) | 501951·0 (379444·0 to 658289·8) |  | 19253·9 (12260·8 to 28111·8) | 23301·3 (18919·8 to 28932·3) |  | 14039·8 (7556·7 to 22498·7) | 0·0 (0·0 to 0·0) |  | 4620·8 (2153·0 to 7918·5) | 0·0 (0·0 to 0·0) |  | 8612225·0 (6096794·4 to 11592099·3) | 0·0 (0·0 to 0·0) |
| Rates† | 2·2 (1·7 to 2·8) | 78·6 (55·4 to 102·4) |  | 1·0 (0·8 to 1·3) | 47·3 (36·4 to 60·2) |  | 5·4 (4·3 to 6·9) | 75·7 (57·2 to 99·3) |  | 2·9 (1·8 to 4·2) | 3·5 (2·9 to 4·4) |  | 2·1 (1·1 to 3·4) | 0·0 (0·0 to 0·0) |  | 0·7 (0·3 to 1·2) | 0·0 (0·0 to 0·0) |  | 1299·3 (919·8 to 1748·8) | 0·0 (0·0 to 0·0) |
| **Central Europe, Eastern Europe, and Central Asia** | |  |  |  |  |  |  |  |  |  |  |  |  |  |  |  |  |  |  |  |
| Absolute number | 581·5 (463·8 to 723·6) | 17089·7 (14006·4 to 20831·9) |  | 199·4 (157·9 to 244·8) | 9974·5 (7785·0 to 12408·5) |  | 612·3 (460·8 to 819·2) | 6788·6 (4963·4 to 9250·4) |  | 313·7 (183·2 to 484·3) | 574·4 (358·7 to 740·8) |  | 960·0 (536·1 to 1472·5) | 0·0 (0·0 to 0·0) |  | 177·5 (69·6 to 315·9) | 0·0 (0·0 to 0·0) |  | 1266979·1 (907307·3 to 1709222·9) | 0·0 (0·0 to 0·0) |
| Rates† | 2·1 (1·7 to 2·6) | 62·0 (50·8 to 75·6) |  | 0·7 (0·6 to 0·9) | 36·2 (28·2 to 45·0) |  | 2·2 (1·7 to 3·0) | 24·6 (18·0 to 33·6) |  | 1·1 (0·7 to 1·8) | 2·1 (1·3 to 2·7) |  | 3·5 (1·9 to 5·3) | 0·0 (0·0 to 0·0) |  | 0·6 (0·3 to 1·1) | 0·0 (0·0 to 0·0) |  | 4597·0 (3292·0 to 6201·6) | 0·0 (0·0 to 0·0) |
| **High-income** |  |  |  |  |  |  |  |  |  |  |  |  |  |  |  |  |  |  |  |  |
| Absolute number | 2198·8 (1731·5 to 2774·7) | 16648·4 (13956·4 to 19744·8) |  | 372·3 (285·6 to 469·2) | 16596·8 (13354·1 to 19084·6) |  | 1071·7 (846·1 to 1325·8) | 7907·1 (6599·5 to 9277·9) |  | 1435·7 (1029·9 to 1938) | 3102·0 (2201·1 to 3724·3) |  | 2733·4 (1708·0 to 4072·2) | 0·0 (0·0 to 0·0) |  | 242·9 (105·6 to 451·6) | 0·0 (0·0 to 0·0) |  | 1464870·8 (1061645·7 to 1962025·3) | 0·0 (0·0 to 0·0) |
| Rates† | 3·9 (3·0 to 4·9) | 29·2 (24·5 to 34·7) |  | 0·7 (0·5 to 0·8) | 29·1 (23·5 to 33·5) |  | 1·9 (1·5 to 2·3) | 13·9 (11·6 to 16·3) |  | 2·5 (1·8 to 3·4) | 5·4 (3·9 to 6·5) |  | 4·8 (3·0 to 7·2) | 0·0 (0·0 to 0·0) |  | 0·4 (0·2 to 0·8) | 0·0 (0·0 to 0·0) |  | 2572·6 (1864·4 to 3445·7) | 0·0 (0·0 to 0·0) |
| **Latin America and Caribbean** | |  |  |  |  |  |  |  |  |  |  |  |  |  |  |  |  |  |  |  |
| Absolute number | 1247·5 (932·0 to 1627·2) | 58216·0 (44143·8 to 75048·8) |  | 551·4 (403·1 to 716·2) | 24414·7 (18169·6 to 31431·5) |  | 1615·9 (1195·6 to 2114·3) | 20607·0 (14712·7 to 28036·7) |  | 1476·6 (994·6 to 2100·3) | 6049·1 (4072·0 to 8180·7) |  | 2972·2 (1825·2 to 4516·9) | 0·0 (0·0 to 0·0) |  | 1270·8 (750·0 to 1948·2) | 0·0 (0·0 to 0·0) |  | 825264·5 (577603·3 to 1124847·6) | 0·0 (0·0 to 0·0) |
| Rates† | 2·6 (1·9 to 3·4) | 121·1 (91·8 to 156·1) |  | 1·1 (0·8 to 1·5) | 50·8 (37·8 to 65·4) |  | 3·4 (2·5 to 4·4) | 42·9 (30·6 to 58·3) |  | 3·1 (2·1 to 4·4) | 12·6 (8·5 to 17·0) |  | 6·2 (3·8 to 9·4) | 0·0 (0·0 to 0·0) |  | 2·6 (1·6 to 4·1) | 0·0 (0·0 to 0·0) |  | 1716·7 (1201·5 to 2339·8) | 0·0 (0·0 to 0·0) |
| **North Africa and Middle East** | |  |  |  |  |  |  |  |  |  |  |  |  |  |  |  |  |  |  |  |
| Absolute number | 579·2 (316·2 to 829·4) | 27609·2 (14630·6 to 40140·5) |  | 524·5 (309·5 to 767·2) | 25443·6 (15012·2 to 37622·6) |  | 3024·8 (2097·4 to 4197·6) | 41899·3 (27429·9 to 61533·9) |  | 3945·2 (2614·1 to 5472·5) | 1156·2 (846·0 to 1517·1) |  | 1141·3 (529·0 to 1957·3) | 0·0 (0·0 to 0·0) |  | 293·3 (105·9 to 555·2) | 0·0 (0·0 to 0·0) |  | 1036284·3 (725628·1 to 1404771·2) | 0·0 (0·0 to 0·0) |
| Rates† | 1·0 (0·5 to 1·4) | 46·2 (24·5 to 67·2) |  | 0·9 (0·5 to 1·3) | 42·6 (25·1 to 63·0) |  | 5·1 (3·5 to 7·0) | 70·2 (45·9 to 103·0) |  | 6·6 (4·4 to 9·2) | 1·9 (1·4 to 2·5) |  | 1·9 (0·9 to 3·3) | 0·0 (0·0 to 0·0) |  | 0·5 (0·2 to 0·9) | 0·0 (0·0 to 0·0) |  | 1735·2 (1215·1 to 2352·3) | 0·0 (0·0 to 0·0) |
| **South Asia** |  |  |  |  |  |  |  |  |  |  |  |  |  |  |  |  |  |  |  |  |
| Absolute number | 1632·8 (1027·4 to 2714·2) | 80150·8 (52181·6 to 128619·3) |  | 1676·3 (1133·8 to 2458·3) | 66054·5 (46376·8 to 93639·5) |  | 4115·8 (2026·9 to 6661·9) | 55367·5 (28267·6 to 87804·3) |  | 4421·6 (2570·5 to 6706·4) | 3354·4 (2171·4 to 5388·5) |  | 3126·9 (1476·3 to 5279·3) | 0·0 (0·0 to 0·0) |  | 1087·4 (423·0 to 1945·3) | 0·0 (0·0 to 0·0) |  | 773984·8 (520591·9 to 1081220·4) | 0·0 (0·0 to 0·0) |
| Rates† | 1·0 (0·6 to 1·7) | 48·8 (31·7 to 78·2) |  | 1·0 (0·7 to 1·5) | 40·2 (28·2 to 57·0) |  | 2·5 (1·2 to 4·1) | 33·7 (17·2 to 53·4) |  | 2·7 (1·6 to 4·1) | 2·0 (1·3 to 3·3) |  | 1·9 (0·9 to 3·2) | 0·0 (0·0 to 0·0) |  | 0·7 (0·3 to 1·2) | 0·0 (0·0 to 0·0) |  | 470·8 (316·7 to 657·7) | 0·0 (0·0 to 0·0) |
| **Southeast Asia, East Asia, and Oceania** | |  |  |  |  |  |  |  |  |  |  |  |  |  |  |  |  |  |  |  |
| Absolute number | 5331·0 (3815·8 to 7388·5) | 125550·3 (96497·6 to 158257·9) |  | 902·8 (684·0 to 1384·0) | 49164·8 (37258·7 to 77211·6) |  | 14402·2 (10934·1 to 19885·4) | 162034·8 (127747·3 to 218314·1) |  | 2441·9 (1528·2 to 3665·0) | 5964·1 (4736·0 to 8371·9) |  | 1353·6 (481·8 to 2564·9) | 0·0 (0·0 to 0·0) |  | 710·0 (274·0 to 1311·5) | 0·0 (0·0 to 0·0) |  | 1762529·2 (1251944·6 to 2410470·9) | 0·0 (0·0 to 0·0) |
| Rates† | 3·8 (2·7 to 5·3) | 89·4 (68·7 to 112·7) |  | 0·6 (0·5 to 1·0) | 35·0 (26·5 to 55·0) |  | 10·3 (7·8 to 14·2) | 115·3 (90·9 to 155·4) |  | 1·7 (1·1 to 2·6) | 4·2 (3·4 to 6·0) |  | 1·0 (0·3 to 1·8) | 0·0 (0·0 to 0·0) |  | 0·5 (0·2 to 0·9) | 0·0 (0·0 to 0·0) |  | 1254·7 (891·2 to 1716·0) | 0·0 (0·0 to 0·0) |
| **Sub-Saharan Africa** |  |  |  |  |  |  |  |  |  |  |  |  |  |  |  |  |  |  |  |  |
| Absolute number | 3268·8 (1782·9 to 5041·9) | 195467·1 (104407·2 to 298175·7) |  | 2391·2 (1355·1 to 3508·0) | 121615·4 (68578·8 to 176187·7) |  | 10981·2 (5848·9 to 15896·3) | 207346·6 (114121·5 to 303926·7) |  | 5219·3 (3140·8 to 7788·1) | 3101·2 (1994·5 to 5777·3) |  | 1752·5 (606·0 to 3407·4) | 0·0 (0·0 to 0·0) |  | 838·8 (302·8 to 1578·3) | 0·0 (0·0 to 0·0) |  | 1482312·3 (1013526·0 to 2071111·2) | 0·0 (0·0 to 0·0) |
| Rates† | 2·0 (1·1 to 3·0) | 118·0 (63·0 to 180·0) |  | 1·4 (0·8 to 2·1) | 73·4 (41·4 to 106·4) |  | 6·6 (3·5 to 9·6) | 125·2 (68·9 to 183·5) |  | 3·2 (1·9 to 4·7) | 1·9 (1·2 to 3·5) |  | 1·1 (0·4 to 2·1) | 0·0 (0·0 to 0·0) |  | 0·5 (0·2 to 1·0) | 0·0 (0·0 to 0·0) |  | 894·8 (611·8 to 1250·2) | 0·0 (0·0 to 0·0) |

Data in parentheses are 95% uncertainty intervals (UIs) unless otherwise stated; † All rates were calculated per 100,000 population.

Abbreviations: DALYs = disability-adjusted life-years.

**Supplementary Table 3.** Incident cases, deaths, and DALYs of total neoplasms and their chances in children under 5-year-old by GBD regions, countries and territories in 1990 and 2019.

|  | **Incident cases (95% uncertainly interval)** | | |  | **Prevalent cases (95% uncertainly interval)** | | |  | **Deaths (95% uncertainly interval)** | | |  | **DALYs (95% uncertainly interval)** | | |
| --- | --- | --- | --- | --- | --- | --- | --- | --- | --- | --- | --- | --- | --- | --- | --- |
|  | **1990** | **2019** | **Percentage change 1990 and 2019** |  | **1990** | **2019** | **Percentage change 1990 and 2019** |  | **1990** | **2019** | **Percentage change 1990 and 2019** |  | **1990** | **2019** | **Percentage change 1990 and 2019** |
| **Central Europe, Eastern Europe, and Central Asia** | |  |  |  |  |  |  |  |  |  |  |  |  |  |  |
| **Central Asia** | 362750·6 (253453·2 to 498401·1) | 366026·0 (256621·9 to 502604·2) | 0·9% (0·3 to 1·6) |  | 354040·2 (247342·8 to 499350·8) | 353036·9 (245237·7 to 499209·0) | -0·3% (-3·4 to 2·7) |  | 887·0 (755·7 to 1120·2) | 465·1 (368·2 to 593·4) | -47·6% (-60·1 to -30·6) |  | 78013·2 (66472·2 to 97669·9) | 40859·6 (32331·9 to 52118·0) | -47·6% (-60·1 to -30·6) |
| Armenia | 14574·5 (10144·6 to 20021·3) | 7808·3 (5435·9 to 10731·4) | -46·4% (-46·7 to -45·9) |  | 14357·9 (10106·1 to 20147·1) | 7786·6 (5347·7 to 11062·2) | -45·8% (-49·7 to -40·6) |  | 38·5 (31·2 to 47·8) | 15·3 (11·7 to 20·1) | -60·2% (-70·8 to -44·1) |  | 3397·3 (2763·3 to 4217·7) | 1353·9 (1035·6 to 1773·9) | -60·1% (-70·9 to -44·0) |
| Azerbaijan | 34753·0 (24224·3 to 47710·4) | 28973·1 (20193·1 to 39824·6) | -16·6% (-17·1 to -16·1) |  | 34572·4 (24395·3 to 48479·0) | 28565·6 (19936·4 to 40509·7) | -17·4% (-23·3 to -10·9) |  | 107·5 (80·5 to 140·1) | 49·3 (28·2 to 83·2) | -54·1% (-77·1 to -10·6) |  | 9496·2 (7094·9 to 12353·8) | 4366·4 (2498·9 to 7358·5) | -54·0% (-76·9 to -10·4) |
| Georgia | 16403·6 (11152·9 to 22430·9) | 8794·9 (6873·3 to 11166·3) | -46·4% (-56·5 to -31·0) |  | 16218·9 (11115·9 to 22906·4) | 8214·4 (6431·7 to 10635·5) | -49·4% (-59·9 to -32·7) |  | 35·1 (27·4 to 45·0) | 14·2 (10·2 to 19·1) | -59·5% (-71·8 to -41·9) |  | 3103·5 (2426·2 to 3972·4) | 1257·9 (891 to 1696·9) | -59·5% (-71·8 to -42·2) |
| Kazakhstan | 70683·3 (49193·5 to 97083·1) | 70120·9 (48742·7 to 96345·4) | -0·8% (-1·1 to -0·4) |  | 69273·1 (48067·0 to 98061·1) | 68071·6 (46863 to 96376·6) | -1·7% (-8·5 to 5·3) |  | 176·8 (145·8 to 214·4) | 96·9 (70·2 to 127·8) | -45·2% (-61·1 to -23·7) |  | 15516·8 (12779·4 to 18831·8) | 8514·6 (6147·3 to 11270·8) | -45·1% (-61·2 to -23·9) |
| Kyrgyzstan | 25968·3 (18123·5 to 35934·0) | 30904·1 (21553·8 to 42748·4) | 19·0% (18·7 to 19·3) |  | 25381·3 (17270·8 to 36161·8) | 29956·7 (20259·8 to 43392·6) | 18·0% (10·3 to 25·9) |  | 42·6 (34·5 to 54·6) | 21·7 (17·6 to 26·4) | -49·2% (-62·0 to -33·2) |  | 3748·9 (3035·4 to 4781·4) | 1900·9 (1540·1 to 2313·5) | -49·3% (-62·1 to -33·4) |
| Mongolia | 12908·0 (8949·1 to 17770·0) | 14976·6 (10379·9 to 20629·0) | 16·0% (15·7 to 16·2) |  | 12267·1 (8449·7 to 17534·8) | 14214·5 (9750·3 to 20414·2) | 15·9% (8·8 to 23·2) |  | 24·2 (16·0 to 35·5) | 10·4 (7·1 to 16·2) | -57·2% (-76·9 to -20·4) |  | 2108·6 (1399·6 to 3103·7) | 905·5 (620·8 to 1418·5) | -57·1% (-76·9 to -20·5) |
| Tajikistan | 35874·1 (24879·4 to 49324·3) | 45866·0 (31792·8 to 63170·6) | 27·9% (27·2 to 28·3) |  | 34619·5 (23775·8 to 48808·8) | 43665·6 (29902·6 to 61658·7) | 26·1% (17·1 to 35·0) |  | 92·3 (61·4 to 185·8) | 53·1 (30·9 to 100·4) | -42·4% (-66·7 to 20·3) |  | 8099·6 (5398·6 to 16190·5) | 4650·5 (2696·9 to 8825·4) | -42·6% (-66·8 to 20·7) |
| Turkmenistan | 22540·1 (15680·0 to 30940·5) | 20992·5 (14576·6 to 28901·3) | -6·9% (-7·3 to -6·4) |  | 22094·0 (15522·5 to 30971·8) | 20252·9 (13892·4 to 28471·3) | -8·3% (-15·8 to -0·4) |  | 72·0 (55·5 to 106·8) | 33·8 (26·1 to 43·8) | -53·0% (-68·9 to -32·5) |  | 6339·7 (4921·4 to 9374·9) | 2976·7 (2284·6 to 3844·6) | -53·0% (-68·6 to -32·5) |
| Uzbekistan | 129045·8 (89605·0 to 177358·8) | 137589·6 (95638·5 to 189291·9) | 6·6% (6·3 to 6·9) |  | 125256·1 (87075·6 to 179230·8) | 132308·8 (90376·8 to 188406·1) | 5·6% (-1·0 to 12·4) |  | 298·0 (240·0 to 369·8) | 170·4 (131·9 to 219·1) | -42·8% (-59·4 to -19·3) |  | 26202·6 (21191·3 to 32343·0) | 14933·2 (11500·4 to 19213·0) | -43·0% (-59·6 to -19·6) |
| **Central Europe** | 446331·0 (316845·4 to 603205·2) | 252569·8 (183099·4 to 337200·2) | -43·4% (-45·2 to -41·3) |  | 430012·0 (303607·2 to 596200·7) | 244080·4 (175601·8 to 333863·8) | -43·2% (-45·9 to -39·4) |  | 775·6 (694·8 to 938·3) | 207·3 (169·2 to 252·5) | -73·3% (-79·4 to -65·9) |  | 68141·8 (61303·7 to 82265·3) | 18641·8 (14948·9 to 22949·7) | -72·6% (-79·3 to -65·0) |
| Albania | 21733·4 (15050·8 to 29601·8) | 8930·4 (6229·3 to 12165·7) | -58·9% (-59·1 to -58·7) |  | 20273·8 (14079·7 to 28148·0) | 8569·4 (6067·8 to 11798·4) | -57·7% (-60·1 to -54·8) |  | 44·0 (31·4 to 83·7) | 17·5 (12·7 to 25·9) | -60·3% (-72·4 to -41·9) |  | 3863·6 (2760·6 to 7314·9) | 1549·7 (1126·9 to 2295·9) | -59·9% (-72·0 to -41·0) |
| Bosnia and Herzegovina | 19382·9 (13415·5 to 26413·7) | 7945·8 (5507·8 to 10821·3) | -59·0% (-59·1 to -58·9) |  | 18193·9 (12684·6 to 25421·3) | 7537·8 (5267·0 to 10491·9) | -58·6% (-60·7 to -56·1) |  | 16·3 (10·9 to 22·0) | 5·1 (3·5 to 6·8) | -68·7% (-79·9 to -50·9) |  | 1427·5 (955·7 to 1918·2) | 452·2 (308·9 to 601·4) | -68·3% (-79·6 to -50·4) |
| Bulgaria | 29285·7 (20337·1 to 39817·8) | 17075·5 (11831·2 to 23236·1) | -41·7% (-41·8 to -41·5) |  | 28053·2 (19511·0 to 39072·5) | 16204·2 (11330·3 to 22553·4) | -42·2% (-44·9 to -39·6) |  | 49·2 (41·9 to 57·9) | 16·1 (12·4 to 20·6) | -67·2% (-75·5 to -55·9) |  | 4329·2 (3688·8 to 5081·7) | 1427·2 (1097·6 to 1823·8) | -67·0% (-75·4 to -55·8) |
| Croatia | 18540·9 (13148·9 to 24849·5) | 13513·8 (10165·5 to 17623·2) | -27·1% (-39·0 to -7·8) |  | 17986·8 (12638·1 to 25137·6) | 12987·0 (9693·4 to 17155·2) | -27·8% (-40·6 to -8·9) |  | 18·8 (15·4 to 22·6) | 6·7 (5·2 to 8·5) | -64·4% (-73·5 to -51·2) |  | 1668·4 (1373·0 to 1995·2) | 609·9 (460·2 to 785·3) | -63·4% (-73·0 to -49·8) |
| Czechia | 36456·0 (25550·0 to 49631·2) | 32044·4 (22445·8 to 43579·3) | -12·1% (-12·4 to -11·5) |  | 34641·7 (24170·2 to 48567·8) | 30736·2 (21777·3 to 43180·6) | -11·3% (-15·5 to -4·9) |  | 45·1 (38·3 to 53·3) | 15·3 (11·7 to 19·7) | -66·1% (-76·0 to -54·0) |  | 3974·7 (3394·4 to 4663·8) | 1398·0 (1043·4 to 1848·7) | -64·8% (-75·7 to -51·8) |
| Hungary | 33524·0 (23257·9 to 45653·1) | 23934·2 (16671·9 to 32547·3) | -28·6% (-28·9 to -28·1) |  | 31632·5 (21789·7 to 44420·3) | 22913·2 (15785·1 to 31947·4) | -27·6% (-32·0 to -21·7) |  | 46·3 (39·4 to 53·7) | 15·5 (11·3 to 20·1) | -66·5% (-74·9 to -55·6) |  | 4070·2 (3452·1 to 4711·4) | 1393·9 (995·9 to 1823·4) | -65·8% (-74·6 to -54·2) |
| Montenegro | 2868·7 (1992·0 to 3899·3) | 1883·6 (1308·6 to 2563·1) | -34·3% (-34·7 to -34·0) |  | 2758·1 (1926·0 to 3810·0) | 1796·1 (1252·0 to 2492·8) | -34·9% (-39·2 to -30·4) |  | 4·4 (3·2 to 5·8) | 1·1 (0·7 to 1·7) | -74·1% (-84·5 to -59·2) |  | 385·1 (280·2 to 512·0) | 101·7 (66·8 to 148·6) | -73·6% (-84·2 to -58·5) |
| North Macedonia | 9578·7 (6632·6 to 13043·3) | 6199·0 (4305·2 to 8439·6) | -35·3% (-35·5 to -35·0) |  | 9047·7 (6291·9 to 12567·0) | 5879·2 (4090·0 to 8146·4) | -71·2% (-82·5 to -55·5) |  | 15·5 (11·7 to 22·4) | 4·4 (3·2 to 5·9) | -71·6% (-82·9 to -55·6) |  | 1361·0 (1022·8 to 1964·0) | 392·4 (283·4 to 526·0) | -71·2% (-82·5 to -55·5) |
| Poland | 80364·6 (53419·8 to 113805·6) | 25110·7 (19131·8 to 32606·8) | -68·8% (-74·2 to -61·0) |  | 78447·8 (51283·3 to 114580·2) | 24971·2 (17686·2 to 34976·1) | -68·2% (-76·3 to -55·3) |  | 230·7 (197·6 to 276·4) | 58·6 (44·4 to 76·8) | -74·6% (-81·4 to -65·8) |  | 20282·4 (17453·4 to 24117·4) | 5301·8 (3958·3 to 7024·4) | -73·9% (-81·2 to -64·9) |
| Romania | 120717·2 (85541·7 to 160801·3) | 63740·4 (45054·3 to 85020·6) | -47·2% (-47·4 to -46·9) |  | 117959·7 (83893·3 to 165585·5) | 61982·2 (43137·4 to 86343·1) | -47·5% (-50·1 to -43·5) |  | 202·7 (172·3 to 256·7) | 43·8 (34·7 to 55·4) | -78·4% (-85·2 to -71·0) |  | 17738·8 (15125·2 to 22457·6) | 3910·5 (3064·8 to 4990·9) | -78·0% (-85·1 to -70·3) |
| Serbia | 43556·0 (30739·9 to 58931·0) | 30166·5 (21254·5 to 40711·6) | -30·7% (-31·5 to -30·1) |  | 42162·2 (29401·9 to 59219·3) | 29315·9 (20415·9 to 41093·6) | -30·5% (-34·4 to -26·5) |  | 73·3 (48·2 to 110·1) | 10·4 (6·4 to 15·6) | -85·8% (-93·1 to -70·8) |  | 6460·5 (4281·3 to 9671·2) | 941·0 (584·9 to 1391·9) | -85·4% (-93·0 to -70·7) |
| Slovakia | 23973·5 (16666·6 to 32754·0) | 16807·1 (11695·0 to 22984·4) | -29·9% (-30·1 to -29·6) |  | 22772·4 (15998·3 to 32333·8) | 16171·9 (11201·6 to 22802·5) | -29·0% (-32·8 to -24·6) |  | 23·6 (19·7 to 27·9) | 10·9 (8·2 to 14·1) | -54·0% (-66·8 to -36·4) |  | 2080·5 (1744·2 to 2454·7) | 982·7 (737·3 to 1274·5) | -52·8% (-65·4 to -34·1) |
| Slovenia | 6349·6 (4359·9 to 8677·6) | 5218·4 (3580·3 to 7126·8) | -17·8% (-18·2 to -17·3) |  | 6082·2 (4214·3 to 8470·1) | 5015·9 (3488·3 to 6964·1) | -17·5% (-23·6 to -9·7) |  | 5·6 (4·7 to 6·7) | 1·9 (1·3 to 2·5) | -65·9% (-76·9 to -52·9) |  | 499·8 (416·4 to 590·5) | 180·7 (119·9 to 243·8) | -63·9% (-76·3 to -49·3) |
| **Eastern Europe** | 914059·1 (648803·4 to 1238651·1) | 655110·6 (465644·3 to 888352·3) | -28·3% (-28·6 to -28·1) |  | 909794·5 (637193·3 to 1279137·6) | 647565·4 (454938·5 to 914128·7) | -28·8% (-30·2 to -27·3) |  | 1686·7 (1496·9 to 1895·6) | 612·3 (513·2 to 718·8) | -63·7% (-71·0 to -56·6) |  | 147721·5 (131724·9 to 165727·5) | 54248·0 (45279·1 to 63976·3) | -63·3% (-70·7 to -56·3) |
| Belarus | 41114·8 (29155·0 to 55929·5) | 28574·4 (20243·4 to 38987·4) | -30·5% (-30·8 to -30·1) |  | 39903·3 (27827·2 to 56499·2) | 27755·8 (19629·6 to 39383·4) | -30·4% (-34·4 to -25·0) |  | 101·1 (81·0 to 126·2) | 32·1 (23·2 to 44·4) | -68·3% (-78·8 to -53·9) |  | 8846·6 (7082·8 to 11044·9) | 2844·3 (2045·5 to 3931·6) | -67·8% (-78·6 to -53·1) |
| Estonia | 6130·1 (4353·0 to 8346·2) | 3532·9 (2501·1 to 4820·3 | -42·4% (-42·6 to -42·1) |  | 5959·4 (4220·8 to 8413·8) | 3402·1 (2383·3 to 4830·8) | -42·9% (-46·3 to -38·8) |  | 12·1 (10·2 to 14·2) | 2·6 (1·8 to 3·4) | -78·7% (-85·8 to -70·8) |  | 1062·1 (903·3 to 1243·2) | 230·2 (154·2 to 300·8) | -78·3% (-85·7 to -70·4) |
| Latvia | 9835·0 (6736·7 to 13561·0) | 5031·6 (3436·8 to 6937·3) | -48·8% (-49·0 to -48·7) |  | 9474·9 (6682·3 to 13409·9) | 4829·1 (3360 to 6870·7) | -49% (-52·2 to -45·7) |  | 21·4 (18·4 to 25·2) | 4·5 (2·9 to 5·9) | -78·9% (-86·7 to -70·8) |  | 1873·8 (1622·6 to 2199·5) | 399·0 (255·3 to 521·0) | -78·7% (-86·6 to -70·5) |
| Lithuania | 15269·2 (10647·7 to 20920·1) | 7599·9 (5290·4 to 10408·0) | -50·2% (-50·4 to -50·0) |  | 14656·0 (10296·6 to 20436·1) | 7289·5 (5032·7 to 10218·6) | -50·3% (-53·1 to -47·1) |  | 27·1 (23·5 to 32·0) | 5·8 (3·9 to 8·1) | -78·8% (-85·9 to -69·1) |  | 2382·0 (2065·4 to 2799·8) | 511·1 (341·6 to 723·7) | -78·5% (-86 to -68·7) |
| Republic of Moldova | 21647·2 (15399·2 to 29479·0) | 8786·8 (6220·0 to 11978·3) | -59·4% (-59·6 to -59·2) |  | 21208·1 (14958·3 to 29548·3) | 8485·9 (5988·2 to 11916·8) | -60·0% (-62·7 to -57·3) |  | 63·1 (49·3 to 78·9) | 11·9 (8·5 to 16·4) | -81·2% (-87·3 to -71·9) |  | 5534·7 (4338·5 to 6912·3) | 1044·7 (746·4 to 1443·2) | -81·1% (-87·2 to -71·9) |
| Russian Federation | 620682·9 (440636·8 to 839944·6) | 487296·1 (346366·6 to 660312·6) | -21·5% (-21·8 to -21·2) |  | 620296·1 (433726·6 to 874634·7) | 482972·5 (338397·1 to 681837·8) | -22·1% (-23·6 to -20·5) |  | 1015·9 (884·3 to 1176·9) | 395·5 (326·9 to 476·1) | -61·1% (-69·9 to -51·3) |  | 89016·8 (77925·8 to 102895·1) | 35167·8 (28860·2 to 42567·3) | -60·5% (-69·7 to -50·6) |
| Ukraine | 199379·8 (141432·5 to 269717·8) | 114289·0 (81181·3 to 154891·1) | -42·7% (-42·8 to -42·5) |  | 198296·9 (139426·3 to 280259·7) | 112830·5 (78424·1 to 159545·0) | -43·1% (-46·1 to -39·9) |  | 446·2 (347·4 to 563·5) | 160·0 (127·4 to 192·8) | -64·1% (-72·7 to -52·3) |  | 39005·3 (30505·3 to 49188·5) | 14050·8 (11203·0 to 16964·3) | -64·0% (-72·6 to -52·2) |
| **High-income** |  |  |  |  |  |  |  |  |  |  |  |  |  |  |  |
| **Australasia** | 14392·9 (9765·0 to 20159·1) | 16747·0 (11432·8 to 23054·3) | 16·4% (13·2 to 20·8) |  | 15116·6 (10194·7 to 21249·8) | 18635·1 (11994·1 to 26348·2) | 23·3% (5·4 to 51·7) |  | 76·9 (66·7 to 89·5) | 52·8 (42·9 to 61·7) | -31·4% (-46·6 to -16·8) |  | 6821·5 (5908·3 to 7883·9) | 4826·5 (3809·3 to 5845·0) | 29·2% (-46·2 to -12·5) |
| Australia | 11739·9 (7935·2 to 16471·6) | 14254·9 (9583·8 to 19864·5) | 21·4% (19·7 to 25·0) |  | 12302·3 (8145·3 to 17603·3) | 15887·6 (9947·9 to 22750·6) | 29·1% (7·7 to 62·1) |  | 61·7 (52·9 to 72·3) | 42·8 (35·0 to 50·5) | -30·6% (-46·3 to -15·3) |  | 5478·3 (4665·3 to 6417·8) | 3926·4 (3105·2 to 4752·9) | -28·3% (-45·4 to -11·4) |
| New Zealand | 2653·0 (1815·7 to 3711·8) | 2492·1 (1823·8 to 3309·7) | -6·1% (-18·5 to 10·5) |  | 2814·2 (1908·1 to 4006·7) | 2747·6 (1906·2 to 3819·2) | -2·4% (-21·4 to 26·1) |  | 15·1 (13·0 to 17·7) | 9·9 (7·8 to 12·1) | -34·3% (-51·3 to -15·2) |  | 1343·2 (1157·0 to 1573·4) | 900·1 (692·4 to 1110·6) | -33% (-50·9 to -13·9) |
| **High-income Asia Pacific** | 1111583·3 (801878·0 to 1463371·0) | 852690·8 (633048·1 to 1111964·0) | -23·3% (-27·4 to -18·9) |  | 1164324·5 (822482·0 to 1585694·9) | 865578·9 (630908·0 to 1154314·4) | -25·7% (-30·2 to -20·1) |  | 548·0 (488·8 to 640·6) | 200·9 (176·2 to 226·1) | -63·3% (-70·5 to -56·2) |  | 48942·6 (43708·8 to 56580·4) | 18404·2 (16011·2 to 20933·3) | -62·4% (-69·7 to -55·3) |
| Brunei Darussalam | 3670·5 (2649·5 to 4865·4) | 3331·9 (2404·4 to 4416·8) | -9·2% (-9·3 to -9·1) |  | 3804·2 (2645·6 to 5200·6) | 3455·6 (2401·1 to 4722·8) | -9·2% (-12·2 to -5·9) |  | 2·4 (1·9 to 2·9) | 2·0 (1·5 to 2·7) | -13·3% (-40·6 to 22·0) |  | 207·6 (167·4 to 258·9) | 181·1 (135·9 to 236·7) | -12·8% (-40·4 to 22·7) |
| Japan | 729581·8 (527411·4 to 971127·7) | 589327·2 (439099·5 to 770259·1) | -19·2% (-25·6 to -12·5) |  | 767850·7 (539425·6 to 1056165·6) | 588727·7 (436126·3 to 778882·2) | -23·3% (-30·0 to -14·7) |  | 296·2 (260·6 to 356·3) | 126·5 (111·9 to 138·6) | -57·3% (-66·3 to -49·8) |  | 26682·1 (23533·3 to 31710·8) | 11635·3 (10168·1 to 12978·8) | -56·4% (-65·7 to -48·5) |
| Republic of Korea | 356551·2 (257244·6 to 472984·9) | 229451·7 (165721·9 to 303737·1) | -35·6% (-35·8 to -35·5) |  | 370951·3 (257665·8 to 504079·8) | 241526·0 (168653·5 to 328786·5) | -34·9% (-37·0 to -32·5) |  | 233·8 (182·6 to 289·5) | 64·7 (47·1 to 86·2) | -72·3% (-80·9 to -56·1) |  | 20671·2 (16160·7 to 25635·7) | 5890·5 (4330·9 to 7829·2) | -72·3% (-80·9 to -56·1) |
| Singapore | 21779·9 (15668·8 to 29001·8) | 30579·9 (22076·9 to 40482·6) | 40·4% (39·5 to 41·9) |  | 21718·3 (15180·6 to 29610·1) | 31869·6 (22228·8 to 43552·4) | 46·7% (41·5 to 51·7) |  | 15·7 (13·3 to 18·6) | 7·6 (5·1 to 10·0) | -51·1% (-69·4 to -30·2) |  | 1381·7 (1177·2 to 1631·0) | 697·3 (468·9 to 907·9) | -49·5% (-68·6 to -27·8) |
| **High-income North America** | 89530·9 (62298·4 to 124609·9) | 68821·6 (50174·7 to 92212·9) | -23·1% (-31·9 to -13·6) |  | 118442·6 (79532·0 to 163993·3) | 97682·6 (65038·2 to 137112·6) | -17·5% (-30·2 to -1·6) |  | 967·9 (883·1 to 1064·5) | 626·9 (566·3 to 685·2) | -35·2% (-43·3 to -27·0) |  | 86685·8 (79194·0 to 94891·0) | 56901·6 (50580·1 to 63432·7) | -34·4% (-42·8 to -25·2) |
| Canada | 6331·2 (4474·3 to 8760·0) | 6457·2 (4592·5 to 8861·0) | 2·0% (-3·6 to 10·5) |  | 10258·3 (7008·8 to 14867·2) | 11581·1 (7316·6 to 17526·8) | 12·9% (-17·1 to 57·5) |  | 87·7 (75·8 to 101·4) | 60·4 (51·6 to 68·6) | -31·1% (-44·1 to -16·2) |  | 7949·3 (6839·2 to 9175·1) | 5664·1 (4739·7 to 6556·8) | -28·7% (-43·0 to -13·4) |
| Greenland | 16·8 (11·6 to 23·8) | 12·0 (8·2 to 17·1) | -28·3% (-29·7 to -27·0) |  | 16·4 (10·4 to 24·3) | 12·2 (7·7 to 18·1) | -25·6% (-38·0 to -7·7) |  | 0·3 (0·2 to 0·4) | 0·1 (0·1 to 0·2) | -68·3% (-82·7 to -42·2) |  | 26·1 (19·2 to 34·9) | 8·3 (5·0 to 13·4) | -68·0% (-82·4 to -42·0) |
| United States of America | 83180·9 (57760·0 to 115095·2) | 62351·3 (45703·4 to 83739·1) | -25·0% (-34·5 to -14·8) |  | 108165·3 (72017·6 to 151151·9) | 86087·7 (57352·9 to 120930·7) | -20·4% (-33·4 to -5·1) |  | 879·8 (796·4 to 971·6) | 566·4 (514·6 to 617·9) | -35·6% (-43·5 to -27·1) |  | 78708·5 (71094·6 to 86686·1) | 51228·3 (45716·6 to 57009·6) | -34·9% (-43·2 to -25·8) |
| **Southern Latin America** | 129931·7 (89768·8 to 178430·8) | 122446·5 (84364·1 to 168167·4) | -5·8% (-6·2 to -4·5) |  | 128154·7 (87709·1 to 177181·8) | 124056·8 (84919·1 to 174210·6) | -3·2% (-11·3 to 11·1) |  | 411·3 (374·6 to 463·5) | 207·5 (172·9 to 253·1) | -49·5% (-58·5 to -38·2) |  | 36118·8 (33054·1 to 40639·8) | 18589·5 (15271·1 to 22805·6) | -48·5% (-58·3 to -36·1) |
| Argentina | 86510·2 (59789·2 to 118766·8) | 87285·4 (60194·5 to 120005·1) | 0·9% (0·4 to 2·4) |  | 85390·4 (58118·0 to 118838·5) | 87122·9 (59134·2 to 122219·8) | 2·0% (-7·9 to 18·4) |  | 293·8 (260·7 to 335·9) | 152·6 (127·8 to 188·1) | -48·1% (-57·5 to -36·3) |  | 25778·1 (22890·2 to 29394·1) | 13586·0 (11310·0 to 16711·0) | -47·3% (-57·3 to -34·3) |
| Chile | 36555·9 (25237·0 to 50215·1) | 29350·3 (20192·0 to 40323·7) | -19·7% (-20·4 to -17·2) |  | 35977·2 (24288·4 to 50718·0) | 31071·7 (20683·0 to 45339·2) | -13·6% (-22·8 to 10·4) |  | 95·7 (85·5 to 110·7) | 45·0 (34·6 to 57·7) | -53·0% (-64·1 to -38·6) |  | 8432·2 (7547·7 to 9690·1) | 4123·0 (3057·3 to 5421·1) | -51·1% (-63·4 to -34·6) |
| Uruguay | 6860·4 (4739·0 to 9441·6) | 5804·6 (3999·9 to 7965·4) | -15·4% (-15·9 to -14·2) |  | 6781·9 (4690·1 to 9555·6) | 5855·9 (4000·8 to 8216·0) | -13·7% (-22·2 to -0·5) |  | 21·7 (18·8 to 25·0) | 9·9 (7·5 to 12·8) | -54·6% (-66·3 to -38·4) |  | 1907·0 (1649·8 to 2188·2) | 879·5 (662·0 to 1153·4) | -53·9% (-65·9 to -37·6) |
| **Western Europe** | 442691·6 (306779·3 to 610257·6) | 423793·9 (296156·6 to 582537·5) | -4·3% (-6·8 to -1·6) |  | 459017·6 (326999·7 to 641757·5) | 440784·7 (314951·9 to 620946·6) | -4·0% (-9·1 to 2·9) |  | 1280·9 (1142·2 to 1473·8) | 586·3 (490·8 to 662·6) | -54·2% (-63·8 to -45·9) |  | 114747·1 (101919·8 to 131126·6) | 54501·9 (44223·9 to 63522·5) | -52·5% (-63·1 to -43·0) |
| Andorra | 50·2 (35·1 to 70·0) | 49·6 (34·1 to 69·5) | -1·1% (-3·3 to 0·5) |  | 55·1 (39·2 to 75·0) | 53·4 (38·0 to 74·3) | -3·1% (-16·9 to 11·3) |  | 0·3 (0·2 to 0·4) | 0·1 (0·1 to 0·1) | -57·1% (-76·1 to -22·3) |  | 22·6 (14·1 to 34·0) | 10·0 (6·7 to 13·7) | -56% (-75·5 to -20·7) |
| Austria | 17154·0 (13641·4 to 21427·0) | 15517·5 (11311·1 to 20853·3) | -9·5% (-26·6 to 11·3) |  | 17052·0 (13794·4 to 20802·7) | 15766·4 (11519·9 to 21247·5) | 7·5% (-28·3 to 15·7) |  | 20·9 (17·4 to 25·1) | 9·8 (8·2 to 11·6) | -53·2% (-63·0 to -40·9) |  | 1883·6 (1558·1 to 2273·3) | 912·1 (741·8 to 1114·6) | -51·6% (-62·3 to -38·0) |
| Belgium | 11275·8 (7726·1 to 15717·0) | 11657·0 (8008·6 to 16188·6) | 3·4% (1·9 to 5·1) |  | 11464·9 (7839·4 to 16413·2) | 11990·5 (8310·7 to 16917·7) | 4·6% (-8·5 to 20·9) |  | 32·7 (27·9 to 38·1) | 15·3 (12·8 to 18·5) | -53·1% (-62·5 to -41·4) |  | 2927·3 (2503·1 to 3425·3) | 1427·1 (1167·9 to 1730·6) | -51·2% (-61·8 to -38·3) |
| Cyprus | 889·4 (601·8 to 1245·3) | 1031·1 (701·6 to 1441·3) | 15·9% (15·1 to 16·9) |  | 852·6 (589·4 to 1197·3) | 1027·0 (724·8 to 1444·0) | 20·5% (6·7 to 36·0) |  | 2·8 (2·1 to 3·6) | 1·7 (1·2 to 2·3) | -40·7% (-63·3 to -8·5) |  | 251·6 (187·4 to 320·6) | 154·1 (108·2 to 208·2) | -38·8% (-61·7 to -5·8) |
| Denmark | 5313·2 (3613·1 to 7361·8) | 5648·2 (3844·8 to 7819·2) | 6·3% (5·1 to 8·1) |  | 5461·1 (3927·1 to 7599·3) | 5986·0 (4090·3 to 8484·5) | 9·6% (-2·8 to 27·2) |  | 18·7 (15·0 to 23·6) | 8·5 (6·1 to 10·5) | -54·5% (-70·6 to -37·2) |  | 1676·7 (1349·0 to 2099·9) | 800·2 (554·5 to 1015·6) | -52·3% (-70·1 to -33·5) |
| Finland | 6174·0 (4152·5 to 8576·9) | 5155·6 (3493·0 to 7141·4) | -16·5% (-17·1 to -15·1) |  | 6127·0 (4266·0 to 8554·2) | 5326·0 (3642·6 to 7588·8) | -13·1% (-22·4 to 0·2) |  | 15·0 (12·2 to 18·5) | 6·7 (4·9 to 8·1) | -55·7% (-67·6 to -41·4) |  | 1341·4 (1091·7 to 1643·5) | 619·0 (449·5 to 772·3) | -53·9% (-66·7 to -37·9) |
| France | 71360·2 (48686·9 to 100124·3) | 67138·7 (45984·3 to 93992·1) | -5·9% (-7·0 to -4·7) |  | 73057·0 (50548·6 to 104160·3) | 70144·8 (48711·6 to 102100·6) | -4·0% (-14·9 to 9·8) |  | 200·6 (166·5 to 241·9) | 107·0 (85·7 to 125·1) | -46·7% (-59·4 to -31·6) |  | 17959·0 (14866·5 to 21744·0) | 9918·2 (7800·1 to 11905·4) | -44·8% (-58·3 to -29·5) |
| Germany | 94591·4 (64668·2 to 131993·0) | 82809·3 (56345·8 to 115059·5) | -12·5% (-13·3 to -11·2) |  | 96454·8 (67586·1 to 136014·0) | 86594·7 (59664·2 to 124429·9) | -10·2% (-20·1 to 3·0) |  | 236·4 (207·8 to 269·6) | 96·5 (80·0 to 110·3) | -59·2% (-67·2 to -51·6) |  | 21143·1 (18583·2 to 24063·8) | 9121·1 (7296·2 to 10842·4) | -56·9% (-66·1 to -46·7) |
| Greece | 10219·5 (7068·5 to 14309·3) | 8340·0 (5748·4 to 11650·3) | -18·4% (-20·2 to -16·7) |  | 10980·0 (7539·9 to 15758·5) | 8916·0 (6156·3 to 12685·9) | -18·8% (-32·5 to -2·4) |  | 27·9 (20·8 to 41·2) | 15·1 (11·9 to 19·4) | -46·0% (-60·2 to -26·5) |  | 2538·7 (1888·6 to 3670·5) | 1402·9 (1097·5 to 1789·1) | -44·7% (-59·5 to -25·1) |
| Iceland | 344·9 (234·5 to 478·3) | 342·9 (232·7 to 474·7) | -0·6% (-1·6 to 0·7) |  | 349·8 (241·6 to 496·2) | 349·2 (241·8 to 489·3) | -0·2% (-11·5 to 14·2) |  | 1·0 (0·8 to 1·3) | 0·5 (0·3 to 0·8) | -47·7% (-67·8 to -16·7) |  | 91·5 (73·5 to 115·2) | 49·0 (31·5 to 73·2) | -46·4% (-67·0 to -16·0) |
| Ireland | 5264·3 (3589·1 to 7425·1) | 5824·4 (3998·9 to 8168·2) | 10·6% (9·5 to 12·3) |  | 5323·6 (3637·0 to 7547·8) | 6016·7 (4125·6 to 8525·9) | 13·0% (-0·2 to 30·6) |  | 12·5 (9·9 to 16·0) | 6·6 (5·0 to 8·3) | -47·2% (-63·0 to -26·4) |  | 1116·1 (888·0 to 1407·5) | 621·2 (456·6 to 803·1) | -44·3% (-61·7 to -22·0) |
| Israel | 9408·7 (6458·9 to 13272·4) | 17317·7 (11826·6 to 24355·2) | 84·1% (82·9 to 86·1) |  | 9121·4 (6260·5 to 12926·4) | 17155·2 (11594·5 to 24431·2) | 88·1% (65·5 to 119·0) |  | 28·0 (21·9 to 38·8) | 23·1 (19·7 to 26·9) | -17·5% (-39·2 to 6·8) |  | 2468·6 (1932·8 to 3399·4) | 2104·4 (1771·8 to 2492·8) | -14·8% (-38·2 to 12·1) |
| Italy | 59913·1 (41106·8 to 82120·8) | 57761·0 (41602·2 to 77477·3) | -3·6% (-13·5 to 8·0) |  | 65839·7 (46740·8 to 91355·9) | 59952·6 (43836·7 to 82693·9) | -8·9% (-19·3 to 3·2) |  | 205·0 (170·7 to 250·8) | 65·1 (52·5 to 73·5) | -68·3% (-77·0 to -59·8) |  | 18444·1 (15352·5 to 22470·7) | 6079·0 (4769·8 to 7080·2) | -67·0% (-76·4 to -58·3) |
| Luxembourg | 430·5 (293·1 to 600·2) | 604·5 (408·3 to 848·4) | 40·4% (36·2 to 42·3) |  | 458·7 (310·3 to 678·8) | 620·4 (418·0 to 884·4) | 35·3% (9·1 to 55·1) |  | 1·3 (1·1 to 1·6) | 0·8 (0·5 to 1·3) | -36·0% (-59·6 to 2·5) |  | 119·9 (99·4 to 142·9) | 78·3 (50·0 to 118·1) | -34·7% (-58·5 to 3·5) |
| Malta | 454·0 (311·9 to 634·5) | 351·1 (241·3 to 490·3) | -22·7% (-23·5 to -21·1) |  | 449·4 (307·4 to 645·2) | 361·1 (247·1 to 516·1) | -19·7% (-29·1 to -4·7) |  | 1·4 (1·1 to 1·7) | 0·7 (0·5 to 1·0) | -48·7% (-65·7 to -24·7) |  | 120·8 (95·8 to 149·8) | 63·8 (43·4 to 90·3) | -47·2% (-64·8 to -23·1) |
| Monaco | 22·5 (15·7 to 31·3) | 30·1 (20·7 to 42·2) | 33·6% (29·1 to 36·8) |  | 33·3 (24·0 to 45·5) | 28·6 (20·6 to 38·5) | 16·2% (-6·4 to 38·6) |  | 0·1 (0·1 to 0·2) | 0·1 (0·0 to 0·1) | -51·1% (-67·4 to -24·7) |  | 11·6 (7·7 to 16·5) | 5·8 (4·1 to 7·8) | -50·2% (-66·8 to -23·8) |
| Netherlands | 17267·5 (11853·8 to 24253·2) | 16225·6 (11198·5 to 22678·1) | -6·0% (-7·2 to -4·5) |  | 17904·3 (12504·3 to 25425·9) | 17096·1 (11804·3 to 23930·5) | -4·5% (-16·5 to 10·2) |  | 49·8 (40·5 to 60·7) | 24·5 (18·6 to 29·2) | -50·8% (-64·1 to -35·7) |  | 4484·2 (3658·9 to 5414·6) | 2295·1 (1711·8 to 2813·4) | -48·8% (-63·1 to -32·2) |
| Norway | 7217·4 (4988·9 to 9838·6) | 7636·8 (5273·4 to 10388·7) | 5·8% (4·9 to 6·7) |  | 7663·5 (5373·4 to 10669·7) | 8122·2 (5662·7 to 11498·2) | 6·0% (-0·2 to 12·0) |  | 13·8 (11·6 to 16·8) | 6·2 (4·7 to 7·3) | -54·9% (-68·3 to -42·4) |  | 1257·9 (1062·1 to 1497·8) | 593·3 (430·6 to 714·0) | -52·8% (-67·7 to -39·5) |
| Portugal | 8828·0 (5941·7 to 12244·8) | 6569·0 (4536·6 to 8981·9) | -25·6% (-33·4 to -17·1) |  | 8926·3 (6259·9 to 12370·8) | 6575·4 (4551·2 to 9221·0) | -26·3% (-38·2 to -11·8) |  | 47·6 (37·6 to 61·1) | 11·1 (8·8 to 13·8) | -76·8% (-84·1 to -67·9) |  | 4198·2 (3329·2 to 5377·9) | 1006·9 (786·8 to 1276·0) | -76·0% (-83·7 to -66·4) |
| San Marino | 30·4 (21·0 to 42·5) | 26·3 (18·4 to 36·6) | 15·8% (13·1 to 17·9) |  | 35·1 (25·8 to 47·9) | 30·0 (21·6 to 40·7) | 17·0% (-0·6 to 36·8) |  | 0·1 (0·1 to 0·1) | 0·1 (0·1 to 0·2) | -29·6% (-55·4 to 11·9) |  | 13·0 (8·3 to 19·4) | 9·4 (6·8 to 12·6) | -27·9% (-53·9 to 15·0) |
| Spain | 38162·5 (26243·9 to 53680·9) | 36897·4 (25242·8 to 51884·1) | -3·3% (-4·0 to -2·1) |  | 38868·8 (27294·9 to 54268·1) | 38291·0 (26918·3 to 54534·2) | -1·5% (-11·3 to 11·3) |  | 125·4 (102·5 to 155·0) | 56·5 (45·5 to 65·6) | -54·9% (-67·5 to -41·3) |  | 11161·8 (9152·8 to 13782·8) | 5220·1 (4145·4 to 6142·7) | -53·2% (-66·4 to -38·5) |
| Sweden | 11735·4 (8106·4 to 16167·4) | 18818·7 (13031·0 to 26070·0) | 60·4% (43·9 to 81·5) |  | 12484·6 (8656·0 to 17495·8) | 20074·2 (13877·0 to 28119·7) | 60·8% (38·5 to 85·9) |  | 29·7 (25·8 to 34·1) | 16·5 (12·6 to 19·5) | -44·3% (-57·6 to -31·8) |  | 2695·2 (2316·5 to 3114·7) | 1555·7 (1136·3 to 1891·5) | -42·3% (-56·8 to -28·6) |
| Switzerland | 7317·3 (4946·3 to 10151·5) | 8203·1 (5509·6 to 11333·9) | 12·1% (10·8 to 14·0) |  | 7609·7 (5355·9 to 10647·2) | 8512·6 (5922·0 to 12082·8) | 11·9% (0·4 to 28·6) |  | 20·7 (16·8 to 26·0) | 11·1 (8·3 to 13·2) | -46·1% (-63·7 to -27·8) |  | 1870·2 (1532·1 to 2320·9) | 1043·3 (751·6 to 1276·2) | -44·2% (-63·2 to -24·4) |
| United Kingdom | 58903·9 (40151·3 to 81742·9) | 49464·9 (34881·5 to 67359·4) | -16·0% (-23·6 to -7·8) |  | 62073·5 (42106·9 to 88773·6) | 51400·6 (36583·6 to 71861·6) | -17·2% (-26·3 to -6·8) |  | 187·8 (154·2 to 228·8) | 102·1 (85·2 to 116·0) | -45·6% (-57·7 to -31·4) |  | 16854·6 (13990·1 to 20143·2) | 9364·4 (7657·3 to 10900·5) | -44·4% (-56·9 to -30·0) |
| **Latin America and Caribbean** |  |  |  |  |  |  |  |  |  |  |  |  |  |  |  |
| **Andean Latin America** | 62318·9 (44160·8 to 83310·6) | 72783·2 (52738·5 to 97092·6) | 16·8% (12·2 to 21·7) |  | 60947·6 (43584·6 to 81629·3) | 69716·4 (50124·8 to 94897·4) | 14·4% (3·3 to 26·5) |  | 653·3 (502·6 to 923·5) | 367·0 (250·6 to 525·3) | -43·8% (-70·3 to -6·1) |  | 57192·4 (44013·3 to 80824·5) | 32281·5 (22154·6 to 46212·0) | -43·6% (-70·2 to -5·7) |
| Bolivia (Plurinational State of) | 12551·5 (8655·0 to 17213·5) | 17845·0 (12204·1 to 24631·7) | 42·2% (38·9 to 44·9) |  | 12446·6 (8517·7 to 17345·6) | 16879·0 (11262·4 to 24170·2) | 35·6% (14·6 to 58·5) |  | 177·3 (101·4 to 271·0) | 134·7 (70·7 to 192·7) | -24·0% (-62·2 to 65·5) |  | 15488·7 (8855·0 to 23683·0) | 11776·5 (6205·4 to 16921·6) | -24·0% (-61·9 to 65·0) |
| Ecuador | 13918·1 (10380·1 to 18039·9) | 18210·8 (14448·1 to 22501·4) | 30·8% (12·4 to 51·0) |  | 12976·3 (9710·7 to 17090·1) | 16488·3 (13297·6 to 20380·8) | 27·1% (4·9 to 54·9) |  | 102·0 (81·9 to 130·8) | 85·5 (53·9 to 123·6) | -16·2% (-54·3 to 30·9) |  | 8926·1 (7192·4 to 11410·6) | 7499·3 (4720·6 to 10822·6) | -16·0% (-54·3 to 30·8) |
| Peru | 35849·3 (24593·2 to 49239·5) | 36727·5 (25114·7 to 50766·3) | 2·4% (-0·4 to 4·1) |  | 35524·7 (24501·2 to 48527·9) | 36349·1 (24567·8 to 51701·7) | 2·3% (-12·1 to 17·9) |  | 373·9 (276·9 to 561·5) | 146·8 (78·4 to 240·4) | -60·7% (-82·5 to -28·2) |  | 32777·6 (24300·2 to 49158·1) | 13005·7 (6961·3 to 21194·6) | -60·3% (-82·3 to -27·8) |
| **Caribbean** | 59518·7 (41249·9 to 82619·2) | 56385·4 (38966·6 to 78085·7) | -5·3% (-6·4 to -4·6) |  | 61177·3 (42518·2 to 85481·4) | 56342·4 (39085·7 to 79973·3) | -7·9% (-15·4 to -2·0) |  | 636·9 (351·9 to 951·1) | 424·5 (230·4 to 682·2) | -33·4% (-51·3 to -7·8) |  | 55717·6 (30823·6 to 83075·6) | 37132·9 (20168·7 to 59613·8) | -33·4% (-51·3 to -7·9) |
| Antigua and Barbuda | 92·8 (64·2 to 129·0) | 72·5 (50·1 to 100·3) | -21·9% (-22·3 to -21·1) |  | 91·4 (63·2 to 129·2) | 73·0 (49·7 to 104·0) | -20·1% (-28·2 to -10·5) |  | 0·3 (0·2 to 0·4) | 0·4 (0·2 to 0·6) | 18·5% (-32·1 to 98·1) |  | 26·7 (20·0 to 34·7) | 31·5 (19·5 to 48·2) | 18·0% (-32·4 to 95·9) |
| Bahamas | 357·5 (246·5 to 496·0) | 307·7 (212·5 to 426·6) | -13·9% (-14·3 to -13·5) |  | 346·4 (230·8 to 495·9) | 296·9 (200·4 to 429·7) | -14·3% (-22·7 to -5·3) |  | 1·7 (1·3 to 2·1) | 1·0 (0·7 to 1·4) | -42·1% (-63·6 to -11·9) |  | 144·9 (111·9 to 185·6) | 83·9 (57·8 to 118·4) | -42·1% (-63·4 to -12·1) |
| Barbados | 283·3 (195·7 to 393·7) | 206·6 (142·9 to 286·4) | -27·1% (-27·4 to -26·7) |  | 277·7 (190·3 to 394·6) | 202·8 (138·0 to 289·7) | -27·0% (-34·8 to -18·9) |  | 1·3 (1·1 to 1·7) | 0·7 (0·5 to 1·0) | -48·6% (-66·7 to -24·4) |  | 116·0 (92·3 to 144·5) | 59·9 (40·7 to 85·5) | -48·4% (-66·5 to -24·2) |
| Belize | 407·5 (281·7 to 564·8) | 531·5 (366·7 to 738·1) | 30·4% (29·4 to 31·2) |  | 418·0 (286·0 to 591·7) | 514·9 (348·4 to 744·1) | 23·2% (10·0 to 36·4) |  | 2·9 (2·3 to 3·6) | 1·6 (1·2 to 2·2) | -44·1% (-62·4 to -19·8) |  | 256·3 (202·6 to 313·1) | 143·3 (106·1 to 190·5) | -44·1% (-62·4 to -19·7) |
| Bermuda | 61·3 (42·3 to 85·1) | 37·4 (25·9 to 51·7) | -39·0% (-39·4 to -38·5) |  | 61·4 (42·8 to 86·9) | 38·8 (27·1 to 54·8) | -36·8% (-43·1 to -28·8) |  | 0·3 (0·3 to 0·4) | 0·1 (0·1 to 0·2) | -59·4% (-73·0 to -41·1) |  | 29·6 (22·3 to 38·8) | 12·2 (8·8 to 16·2) | -59·0% (-72·8 to -40·7) |
| Cuba | 12730·3 (8793·7 to 17662·5) | 7966·4 (5511·7 to 11023·2) | -37·4% (-37·8 to -36·9) |  | 12684·1 (8710·6 to 17947·4) | 7980·6 (5410·6 to 11416·9) | -37·1% (-43·7 to -29·7) |  | 71·9 (62·5 to 84·0) | 24·9 (17·5 to 31·7) | -65·5% (-77·6 to -53·6) |  | 6296·4 (5474·9 to 7333·4) | 2189·6 (1537·2 to 2798·0) | -65·2% (-77·5 to -52·8) |
| Dominica | 117·8 (81·2 to 163·5) | 60·3 (41·7 to 83·6) | -48·8% (-49·0 to -48·4) |  | 115·3 (78·2 to 167·1) | 59·8 (41·1 to 84·3) | -48·1% (-53·3 to -42·7) |  | 0·7 (0·6 to 0·9) | 0·5 (0·3 to 0·7) | -30·7% (-54·9 to 4·6) |  | 60·6 (48·5 to 74·4) | 42·0 (28·1 to 60·6) | -30·8% (-54·9 to 4·6) |
| Dominican Republic | 14944·4 (10346·6 to 20762·3) | 15555·8 (10722·9 to 21581·3) | 4·1% (2·9 to 5·0) |  | 15162·2 (10324·4 to 21355·5) | 15289·0 (10364·0 to 21774·7) | 0·8% (-10·8 to 13·2) |  | 124·2 (91·8 to 177·8) | 68·8 (46·1 to 100·5) | -44·6% (-66·2 to -12·6) |  | 10920·7 (8105·9 to 15578·8) | 6047·9 (4057·8 to 8835·8) | -44·6% (-66·2 to -13·1) |
| Grenada | 151·0 (104·5 to 209·1) | 99·4 (68·5 to 137·7) | -34·2% (-34·6 to -33·8) |  | 150·7 (104·7 to 213·2) | 96·7 (65·6 to 138·4) | -35·9% (-42·6 to -28·8) |  | 1·0 (0·7 to 1·3) | 0·4 (0·2 to 0·5) | -63·2% (-77·5 to -40·3) |  | 84·0 (62·5 to 111·4) | 31·0 (20·6 to 45·1) | -63·0% (-77·4 to -40·0) |
| Guyana | 1459·7 (1002·9 to 2027·8) | 1004·2 (691·4 to 1391·9) | -31·2% (-31·5 to -30·9) |  | 1406·0 (961·4 to 2028·5) | 958·2 (641·0 to 1376·5) | -31·8% (-38·6 to -24·7) |  | 6·4 (4·9 to 8·1) | 3·1 (2·1 to 4·6) | -50·8% (-69·9 to -19·8) |  | 558·2 (431·1 to 711·0) | 274·9 (185·6 to 403·5) | -50·8% (-69·8 to -19·7) |
| Haiti | 15301·2 (10696·6 to 21287·0) | 21948·7 (15190·3 to 30421·9) | 43·4% (37·6 to 46·6) |  | 16894·6 (10831·4 to 24553·3) | 22330·8 (15246·1 to 31613·3) | 32·2% (4·0 to 56·5) |  | 351·4 (96·9 to 654·3) | 288·5 (111·4 to 525·2) | -17·9% (-46·7 to 59·8) |  | 30668·0 (8471·2 to 57061·8) | 25182·3 (9734·7 to 45741·0) | -17·9% (-46·6 to 59·4) |
| Jamaica | 3939·2 (2721·5 to 5457·5) | 2609·0 (1795·0 to 3621·5) | -33·8% (-34·1 to -33·5) |  | 3934·0 (2751·6 to 5593·0) | 2520·2 (1691·6 to 3622·9) | -35·9% (-42·6 to -29·2) |  | 20·2 (16·2 to 26·0) | 6·9 (4·8 to 9·6) | -65·8% (-77 to -49·4) |  | 1765·5 (1416·7 to 2268·0) | 602·5 (420·9 to 843·4) | -65·9% (-77·0 to -49·6) |
| Puerto Rico | 4531·7 (3126·5 to 6295·9) | 1866·5 (1288·0 to 2586·3) | -58·8% (-59·1 to -58·3) |  | 4427·9 (2985·4 to 6344·0) | 1876·4 (1277·4 to 2707·9) | -57·6% (-62·0 to -51·7) |  | 15·3 (12·6 to 18·8) | 3·6 (2·7 to 4·6) | -76·5% (-83·9 to -66·7) |  | 1341·2 (1107·3 to 1645·8) | 321·1 (239·5 to 414·6) | -76·1% (-83·7 to -66·0) |
| Saint Kitts and Nevis | 66·0 (45·8 to 91·2) | 50·8 (35·2 to 70·3) | -23·0% (-23·8 to -22·2) |  | 67·9 (47·4 to 95·6) | 51·0 (34·2 to 72·8) | -24·8% (-33·0 to -14·3) |  | 0·4 (0·3 to 0·5) | 0·2 (0·1 to 0·3) | -59·4% (-85·9 to -33·2) |  | 36·8 (28·8 to 45·5) | 15·0 (5·4 to 23·5) | -59·2% (-85·5 to -33·0) |
| Saint Lucia | 262·6 (181·5 to 363·4) | 131·4 (90·9 to 181·6) | -50·0% (-50·3 to -49·5) |  | 141·4 (109·4 to 184·1) | 129·3 (87·3 to 186·7) | -49·8% (-55·5 to -44·0) |  | 1·6 (1·3 to 2·1) | 0·5 (0·4 to 0·8) | -66·5% (-79·0 to -47·8) |  | 141·4 (109·4 to 184·1) | 47·5 (31·5 to 70·6) | -66·4% (-79·0 to -47·7) |
| Saint Vincent and the Grenadines | 189·8 (131·3 to 263·3) | 109·7 (75·6 to 152·1) | -42·2% (-42·6 to -41·9) |  | 193·0 (133·7 to 273·5) | 106·3 (70·8 to 154·3) | -44·9% (-51·0 to -38·9) |  | 1·3 (1·0 to 1·7) | 0·4 (0·3 to 0·6) | -68·2% (-80·2 to -49·3) |  | 113·3 (87·0 to 145·8) | 36·0 (24·2 to 52·3) | -68·2% (-80·2 to -49·5) |
| Suriname | 621·5 (428·8 to 860·2) | 648·3 (447·6 to 897·8) | 4·3% (3·7 to 5·0) |  | 615·4 (414·3 to 870·4) | 630·4 (424·5 to 908·2) | 2·4% (-8·4 to 13·3) |  | 4·1 (2·7 to 5·3) | 3·0 (2·0 to 4·4) | -24·8% (-53·1 to 20·1) |  | 355·8 (240·0 to 461·5) | 267·4 (177·3 to 386·1) | -24·8% (-53·0 to 19·8) |
| Trinidad and Tobago | 1864·3 (1291·0 to 2581·1) | 1178·8 (814·8 to 1627·7) | -36·8% (-37·2 to -36·1) |  | 1887·9 (1283·2 to 2726·5) | 1191·8 (829·2 to 1701·4) | -36·9% (-43·2 to -29·2) |  | 10·3 (8·1 to 12·5) | 5·3 (3·7 to 7·5) | -48·1% (-64·3 to -24·5) |  | 903·5 (713·1 to 1096·2) | 469·9 (327·0 to 657·1) | -48·0% (-64·2 to -24·9) |
| United States Virgin Islands | 154·1 (106·0 to 214·0) | 90·4 (62·4 to 125·6) | -41·3% (-41·5 to -41·1) |  | 148·1 (101·0 to 213·7) | 86·9 (58·5 to 126·0) | -41·3% (-47·5 to -34·9) |  | 0·5 (0·3 to 0·7) | 0·2 (0·1 to 0·3) | -59·3% (-76·8 to -27·7) |  | 42·5 (27·1 to 60·6) | 17·3 (10·3 to 26·5) | -59·3% (-76·9 to -27·9) |
| **Central Latin America** | 657332·6 (451589·4 to 914653·7) | 631374·7 (446985·4 to 854666·3) | -3·9% (-8·0 to 0·1) |  | 670590·2 (460140·5 to 942197·7) | 644130·1 (454952·8 to 892958·4) | -3·9% (-9·0 to 1·6) |  | 2150·1 (1838·7 to 2493·3) | 1285·8 (970·2 to 1693·1) | -40·2% (-56·8 to -17·8) |  | 188655·7 (161750·6 to 218818·5) | 113344·6 (85413·6 to 149835·2) | -39·9% (-56·6 to -17·4) |
| Colombia | 117788·0 (80566·5 to 163944·4) | 106194·5 (72568·6 to 147392·7) | -9·8% (-10·1 to -9·3) |  | 118158·2 (80444·3 to 167155·5) | 109108·1 (74571·9 to 154488·2) | -7·7% (-14 to -0·1) |  | 344·7 (297·0 to 399·6) | 248·3 (179·8 to 337·1) | -28·0% (-49·6 to 2·8) |  | 30230·5 (26091·3 to 35053·7) | 21896·7 (15782·0 to 29722·2) | -27·6% (-49·2 to 4·0) |
| Costa Rica | 11213·7 (7682·0 to 15588·0) | 9397·3 (6421·3 to 13047·5) | -16·2% (-16·4 to -15·9) |  | 11420·0 (7832·1 to 16266·1) | 9594·9 (6615·4 to 13789·0) | -16·0% (-22·0 to -9·2) |  | 25·3 (21·2 to 31·3) | 12·2 (9·1 to 15·8) | -51·9% (-67·0 to -32·8) |  | 2219·9 (1865·7 to 2750·1) | 1076·7 (802·5 to 1406·7) | -51·5% (-66·8 to -32·2) |
| El Salvador | 20726·0 (14182·2 to 28778·3) | 15767·1 (10808·6 to 21938·9) | -23·9% (-24·4 to -23·5) |  | 21316·1 (14949·6 to 30051·0) | 16006·8 (10786·9 to 22979·0) | -24·9% (-30·7 to -19·3) |  | 67·2 (51·6 to 91·2) | 17·8 (7·8 to 29·5) | -73·5% (-88·9 to -50·4) |  | 5931·0 (4546·8 to 7989·3) | 1575·8 (693·5 to 2607·6) | -73·4% (-88·8 to -50·1) |
| Guatemala | 40251·6 (27499·1 to 56105·0) | 55180·5 (37716·3 to 76695·3) | 37·1% (36·6 to 37·8) |  | 40026·0 (26999·9 to 56955·5) | 55495·7 (37649·2 to 79136·2) | 38·6% (28·9 to 49·2) |  | 97·4 (77·7 to 140·8) | 94·1 (67·0 to 131·0) | -3·4% (-44·4 to 48·5) |  | 8546·8 (6841·4 to 12328·5) | 8269·1 (5877·9 to 11517·6) | -3·2% (-44·2 to 48·2) |
| Honduras | 22919·6 (15660·2 to 31779·5) | 31337·3 (21444·2 to 43481·8) | 36·7% (35·9 to 37·5) |  | 23573·9 (16128·5 to 33169·6) | 32030·6 (22081·2 to 45503·1) | 35·9% (25·0 to 46·1) |  | 130·8 (94·3 to 169·5) | 106·8 (56·4 to 165·2) | -18·3% (-53·3 to 25·4) |  | 11520·6 (8276·6 to 14875·3) | 9430·4 (4985·3 to 14581·5) | -18·1% (-53·1 to 25·6) |
| Mexico | 347157·7 (239357·9 to 484503·2) | 321106·6 (232641·9 to 429100·1) | -7·5% (-14·8 to 0·2) |  | 358562·6 (245265·7 to 504794·7) | 328652·9 (239649·3 to 449922·6) | -8·3% (-16·5 to 1·3) |  | 1195·9 (918·7 to 1510·5) | 633·1 (476·8 to 807·9) | -47·1% (-63·1 to -23·2) |  | 104986·4 (80919·7 to 132157·8) | 55887·5 (42095·0 to 71464·2) | -46·8% (-62·9 to -23·3) |
| Nicaragua | 18686·8 (12746·8 to 25953·4) | 18246·0 (12499·1 to 25365·7) | -2·4% (-2·8 to -2·0) |  | 18869·9 (12719·4 to 26973·5) | 18552·7 (12637·6 to 26789·0) | -1·7% (-8·3 to 6·2) |  | 65·4 (47·7 to 87·2) | 38·2 (27·0 to 52·7) | -41·6% (-64·5 to -9·0) |  | 5711·3 (4181·6 to 7609·6) | 3338·4 (2364·3 to 4596·6) | -41·5% (-64·3 to -8·9) |
| Panama | 7947·6 (5440·0 to 11052·7) | 10686·3 (7305·9 to 14842·9) | 34·5% (34·1 to 34·9) |  | 7969·5 (5419·0 to 11367·4) | 10854·0 (7325·0 to 15518·6) | 36·2% (27·2 to 46·8) |  | 20·2 (15·8 to 26·5) | 19·8 (14·7 to 26·8) | -1·7% (-31·8 to 40·8) |  | 1762·6 (1376·3 to 2312·8) | 1741·9 (1293·2 to 2357·3) | -1·2% (-31·4 to 42·2) |
| Venezuela (Bolivarian Republic of) | 70641·6 (48330·7 to 98409·5) | 63459·0 (43325·0 to 88186·0) | -10·2% (-10·4 to -9·8) |  | 70694·0 (48024·3 to 101661·7) | 63834·5 (43754·9 to 91463·6) | -9·7% (-16·4 to -2·4) |  | 203·1 (174·1 to 233·3) | 115·5 (83·5 to 155·8) | -43·1% (-60·4 to -20·5) |  | 17746·8 (15242·8 to 20351·7) | 10127·9 (7347·4 to 13740·5) | -42·9% (-60·4 to -20·0) |
| **Tropical Latin America** | 103224·5 (72716·2 to 141232·6) | 79545·3 (55435·0 to 107945·4) | -22·9% (-26·5 to -19·0) |  | 110380·2 (80601·6 to 148916·0) | 82227·2 (59498·5 to 112825·0) | -25·5% (-33·3 to -17·2) |  | 2194·0 (1758·0 to 2741·4) | 1068·2 (830·2 to 1327·8) | -51·3% (-66·8 to -33·1) |  | 191866·0 (154279·8 to 239141·0) | 93832·0 (72791·1 to 116499·1) | -51·1% (-66·7 to -33·1) |
| Brazil | 99597·9 (70266·9 to 136016·8) | 75850·4 (52919·1 to 102901·5) | -23·8% (-27·5 to -19·7) |  | 106763·2 (77879·6 to 143905·2) | 78740·4 (57084·4 to 107712·0) | -26·2% (-34·4 to -17·7) |  | 2153·2 (1723·1 to 2695·7) | 1050·5 (818·3 to 1307·9) | -51·2% (-66·9 to -33·0) |  | 188290·8 (151043·7 to 235476·3) | 92268·2 (71578·8 to 114635·2) | -51·0% (-66·7 to -32·8) |
| Paraguay | 3626·6 (2450·3 to 5040·3) | 3694·9 (2491·7 to 5149·6) | 1·9% (0·2 to 3·2) |  | 3617·0 (2505·6 to 5073·5) | 3486·8 (2353·6 to 5023·4) | -3·6% (-20·8 to 13·7) |  | 40·8 (30·5 to 54·3) | 17·7 (9·9 to 29·4) | -56·5% (-77·3 to -24·1) |  | 3575·3 (2684·5 to 4717·9) | 1563·8 (875·1 to 2585·5) | -56·3% (-77·2 to -24·1) |
| **North Africa and Middle East** | |  |  |  |  |  |  |  |  |  |  |  |  |  |  |
| **North Africa and Middle East** | 951369·5 (665623·7 to 1279773·7) | 1052383·8 (740809·4 to 1419417·8) | 10·6% (9·3 to 12·0) |  | 992683·5 (694849·1 to 1344612·7) | 1089945·4 (757215·4 to 1496119·3) | 9·8% (3·4 to 15·8) |  | 5562·6 (3355·3 to 7964·7) | 2977·9 (2142·2 to 3849·9) | -46·5% (-65·1 to -2·7) |  | 490024·0 (297078·1 to 700637·2) | 263794·8 (190532·7 to 341275·5) | -46·2% (-64·9 to -2·5) |
| Afghanistan | 35612·0 (24784·1 to 47827·8) | 116016·4 (80519·9 to 156824·2) | 225·8% (217·0 to 232·7) |  | 37743·5 (25422·0 to 51789·8) | 118245·8 (79943·6 to 164734·5) | 213·3% (165·0 to 260·6) |  | 430·6 (145·9 to 801·7) | 620·4 (354·9 to 1003·1) | 44·1% (-30·8 to 332·2) |  | 37787·7 (12831·7 to 70184·1) | 54558·1 (31156·4 to 87997·5) | 44·4% (-30·4 to 328·7) |
| Algeria | 65190·2 (45300·1 to 88430·1) | 74596·9 (51863·4 to 101125·1) | 14·4% (14·0 to 14·9) |  | 65463·5 (44587·7 to 91478·2) | 75503·1 (50423·3 to 103874·6) | 15·3% (5·2 to 26·3) |  | 182·2 (125·2 to 250·5) | 125·4 (90·2 to 172·0) | -31·2% (-56·4 to 9·0) |  | 16023·5 (11014·0 to 21927·0) | 11087·6 (8007·4 to 15249·4) | -30·8% (-56·0 to 9·3) |
| Bahrain | 1090·3 (759·0 to 1477·1) | 1235·6 (858·6 to 1676·7) | 13·3% (12·6 to 14·2) |  | 1074·5 (727·0 to 1497·0) | 1268·0 (857·5 to 1756·5) | 18·0% (7·7 to 29·4) |  | 2·3 (1·8 to 3·0) | 1·6 (1·2 to 2·1) | -33·5% (-52·7 to -5·1) |  | 206·4 (157·5 to 266·2) | 138·9 (106·7 to 184·8) | -32·7% (-52·2 to -4·0) |
| Egypt | 149059·8 (103929·5 to 201770·8) | 188831·3 (131033·2 to 255980·4) | 26·7% (25·5 to 27·7) |  | 150354·6 (102142·7 to 206888·3) | 192150·9 (130898·7 to 267915) | 27·8% (16·2 to 41·0) |  | 748·3 (540·1 to 1289·6) | 435·0 (285·7 to 645·8) | -41·9% (-68·2 to 4·0) |  | 65824·7 (47601·0 to 112832·9) | 38486·5 (25413·0 to 57292·4) | -41·5% (-68·0 to 4·4) |
| Iran (Islamic Republic of) | 159040·3 (110119·7 to 213144·8) | 121640·5 (84401·5 to 163426·6) | -23·5% (-24·5 to -22·8) |  | 168650·5 (120400·4 to 227062·5) | 126169·9 (89032·2 to 171653·4) | -25·2% (-30·6 to -20·0) |  | 1061·8 (660·6 to 1578·5) | 279·0 (149·7 to 398·5) | -73·7% (-86·1 to -51·3) |  | 93777·9 (58650·1 to 139329·4) | 25007·3 (13541·5 to 35506·7) | -73·3% (-85·8 to -51·0) |
| Iraq | 55233·4 (38692·3 to 74410·9) | 81482·6 (56806·8 to 110194·9) | 47·5% (46·0 to 49·0) |  | 57826·1 (39552·8 to 79276·5) | 84904·7 (59572·0 to 116890·4) | 46·8% (32·4 to 64·5) |  | 262·8 (161·2 to 388·1) | 199·8 (129·7 to 296·9) | -24·0% (-57·4 to 48·4) |  | 23284·0 (14387·6 to 34527·5) | 17741·4 (11590·6 to 26231·1) | -23·8% (-57·2 to 48·7) |
| Jordan | 8175·0 (5738·3 to 11080·2) | 17455·7 (12638·1 to 23298·3) | 113·5% (90·1 to 141·8) |  | 8031·5 (5517·1 to 11065·3) | 18069·4 (13275·3 to 24413·9) | 125·0% (92·4 to 168·2) |  | 24·3 (17·4 to 32·9) | 34·1 (24·2 to 48·7) | 40·7% (-6·2 to 119·5) |  | 2158·7 (1542·7 to 2931·4) | 3077·6 (2186·5 to 4386·2) | 42·6% (-5·3 to 121·4) |
| Kuwait | 3725·9 (2607·6 to 5033·8) | 5249·3 (3656·9 to 7089·9) | 40·9% (39·5 to 42·1) |  | 4163·1 (2949·7 to 5688·7) | 5692·2 (3932·8 to 7758·2) | 36·7% (22·8 to 51·6) |  | 16·4 (12·5 to 20·7) | 9·6 (7·5 to 12·1) | -41·5% (-58·0 to -18·8) |  | 1456·9 (1115·4 to 1846·7) | 871·6 (681·6 to 1099·5) | -40·2% (-57·0 to -16·5) |
| Lebanon | 8483·6 (5917·9 to 11474·3) | 8866·1 (6213·3 to 11995·4) | 4·5% (3·8 to 5·4) |  | 8533·8 (5738·0 to 11743·3) | 9344·2 (6429·8 to 12796·9) | 9·5% (-0·7 to 21·6) |  | 21·7 (12·2 to 33·6) | 15·9 (8·3 to 24·5) | -26·8% (-60·4 to 38·3) |  | 1924·0 (1077·2 to 2974·1) | 1433·5 (761·8 to 2223·8) | -25·5% (-59·6 to 39·4) |
| Libya | 12296·2 (8619·0 to 16604·7) | 7412·3 (5157·3 to 10052·7) | -39·7% (-40·2 to -39·2) |  | 12528·7 (8494·6 to 17365·1) | 7624·4 (5176·7 to 10593) | -39·1% (-44·7 to -33·1) |  | 49·8 (25·2 to 80·9) | 18·4 (12·0 to 27·0) | -63·1% (-77·9 to -27·4) |  | 4382·8 (2210·2 to 7100·3) | 1617·1 (1059·5 to 2376·7) | -63·1% (-77·9 to -27·1) |
| Morocco | 61897·3 (43160·3 to 83907·8) | 53061·5 (36787·9 to 72053·0) | -14·3% (-14·7 to -13·8) |  | 61311·4 (40802·9 to 86362·7) | 53314·2 (35260·4 to 73624·0) | -13·0% (-21·1 to -4·2) |  | 141·0 (80·2 to 228·6) | 65·4 (39·1 to 103·9) | -53·6% (-76·5 to -3·9) |  | 12385·0 (7075·9 to 19984·7) | 5776·5 (3448·7 to 9163·9) | -53·4% (-76·3 to -3·4) |
| Oman | 5477·1 (3814·2 to 7430·6) | 6860·6 (4785·7 to 9308·3) | 25·3% (24·6 to 26·0) |  | 5528·0 (3714·2 to 7747·6) | 7186·8 (4945·3 to 9899·9) | 30·0% (18·7 to 43·1) |  | 13·2 (8·5 to 18·4) | 12·1 (8·2 to 15·8) | -8·1% (-42·9 to 60·3) |  | 1161·6 (750·3 to 1620·9) | 1085·8 (733·2 to 1417·0) | -6·5% (-41·8 to 63·0) |
| Palestine | 7220·7 (5051·8 to 9733·6) | 10885·8 (7589·5 to 14747·4) | 50·8% (49·0 to 52·4) |  | 7617·8 (5364·2 to 10420·3) | 11263·4 (7697·2 to 15805·2) | 47·9% (32·1 to 63·5) |  | 33·0 (17·8 to 53·3) | 24·7 (17·0 to 35·4) | -25·3% (-56·6 to 45·5) |  | 2918·5 (1583·0 to 4697·7) | 2187·2 (1510·1 to 3136·1) | -25·1% (-56·5 to 46·3) |
| Qatar | 1155·7 (810·4 to 1552·6) | 3316·4 (2331·4 to 4468·5) | 186·9% (185·3 to 188·7) |  | 1221·6 (841·5 to 1711·8) | 3626·1 (2530·2 to 5048·9) | 196·8% (175·6 to 220·3) |  | 2·0 (1·2 to 2·9) | 3·3 (2·3 to 4·6) | 64·4% (-3·4 to 188·7) |  | 174·5 (109·9 to 258·4) | 293·6 (211·2 to 412·0) | 68·3% (-1·4 to 195·5) |
| Saudi Arabia | 43632·8 (30396·6 to 59226·5) | 39704·8 (27619·6 to 53803·8) | -9·0% (-9·6 to -8·4) |  | 43417·6 (28856·1 to 60577·6) | 40638·6 (27526·3 to 55714·8) | -6·4% (-14·8 to 4·5) |  | 88·0 (49·0 to 141·3) | 39·2 (25·3 to 57·2) | -55·4% (-78·6 to -8·3) |  | 7724·6 (4334·1 to 12378·1) | 3531·9 (2260·8 to 5084·8) | -54·3% (-78·0 to -6·5) |
| Sudan | 64945·8 (45124·0 to 86931·5) | 96608·8 (67366·3 to 130517·4) | 48·8% (43·4 to 52·8) |  | 69762·5 (46067·3 to 98964·3) | 100085·8 (68945·4 to 138279·0) | 43·5% (16·2 to 68·4) |  | 721·3 (223·1 to 1419·1) | 464·0 (244·6 to 744·9) | -35·7% (-68·9 to 122·6) |  | 63263·9 (19599·0 to 125033·3) | 40851·4 (21607·2 to 65550·6) | -35·4% (-68·7 to 122·9) |
| Syrian Arab Republic | 40342·6 (28170·7 to 54322·3) | 19884·5 (13905·4 to 26944·1) | -50·7% (-51·3 to -50·2) |  | 42788·5 (29367·8 to 58637·6) | 20812·5 (14312·6 to 28720·3) | -51·4% (-57·0 to -45·5) |  | 199·6 (100·7 to 336·0) | 52·3 (33·8 to 92·8) | -73·8% (-86·0 to -48·6) |  | 17729·0 (8886·4 to 29713·0) | 4640·5 (3003·2 to 8161·8) | -73·8% (-86·0 to -48·5) |
| Tunisia | 19846·1 (13820·9 to 26896·4) | 15134·7 (10524·7 to 20509·8) | -23·7% (-24·3 to -23·3) |  | 5837·2 (3581·6 to 8509·4) | 15490·1 (10378·8 to 21662·7) | -24·1% (-30·8 to -16·7) |  | 66·1 (40·8 to 96·4) | 18·5 (13·1 to 25·2) | -72·0% (-83·2 to -49·9) |  | 20406·8 (14053·2 to 28073·0) | 1653·8 (1184·9 to 2257·2) | -71·7% (-82·9 to -49·1) |
| Turkey | 154556·4 (108197·8 to 207667·0) | 99527·3 (68863·5 to 133806·9) | -35·6% (-36·8 to -34·5) |  | 170122·2 (114871·9 to 231361·6) | 112164·8 (79084·4 to 154973·6) | -34·1% (-42·7 to -24·4) |  | 1197·6 (645·8 to 1836·5) | 298·8 (210·2 to 391·9) | -75·1% (-85·1 to -48·3) |  | 105527·7 (56910·2 to 162868·0) | 26762·0 (18888·2 to 35137·1) | -74·6% (-85·0 to -47·5) |
| United Arab Emirates | 3982·4 (2778·3 to 5398·6) | 5838·8 (4061·0 to 7941·1) | 46·6% (45·4 to 47·9) |  | 4013·9 (2712·5 to 5625·6) | 6039·0 (4108·2 to 8442·7) | 50·4% (37·7 to 64·9) |  | 13·5 (9·2 to 18·3) | 9·5 (5·1 to 14·6) | -29·8% (-62·4 to 20·8) |  | 1184·2 (813·3 to 1599·2) | 838·1 (448·9 to 1292·2) | -29·2% (-62·2 to 22·3) |
| Yemen | 49766·0 (34801·6 to 67246·8) | 77704·8 (54042·8 to 105354·5) | 56·1% (53·8 to 58·2) |  | 51455·6 (35336·4 to 71286·4) | 79244·1 (53843·4 to 110333·5) | 54·0% (35·8 to 71·6) |  | 283·4 (123·6 to 496·3) | 248·1 (136·5 to 369·8) | -12·5% (-52·7 to 140) |  | 24961·8 (10929·1 to 43752·3) | 21886·5 (12082·1 to 32604·1) | -12·3% (-53·0 to 138·8) |
| **South Asia** |  |  |  |  |  |  |  |  |  |  |  |  |  |  |  |
| **South Asia** | 734033·9 (506005·8 to 1015418·0) | 801216·1 (547095·2 to 1103573·7) | 9·2% (2·8 to 16·5) |  | 739521·6 (501795·7 to 1035151·0) | 784800·7 (537236·0 to 1106327·2) | 6·1% (-5·9 to 19·8) |  | 16898·9 (8592·9 to 26571·7) | 9298·5 (7051·1 to 11778·5) | -45·0% (-64·6 to -0·5) |  | 1473870·2 (749682·0 to 2317724·7) | 814344·1 (617468·4 to 1030524·1) | -44·7% (-64·5 to -0·1) |
| Bangladesh | 70670·1 (48683·4 to 99283·5) | 51432·7 (34966·0 to 72731·5) | -27·2% (-31·1 to -24·5) |  | 73008·9 (45328·7 to 107149·6) | 48441·3 (31126·7 to 71043·6) | -33·7% (-50·4 to -12·8) |  | 2466·0 (1167·6 to 4066·7) | 799·4 (582·3 to 1066·0) | -67·6% (-82·9 to -27·3) |  | 215400·3 (102287·9 to 355841·4) | 69958·8 (50865·1 to 93486·9) | -67·5% (-82·8 to -27·3) |
| Bhutan | 365·5 (251·3 to 513·0) | 247·4 (168·9 to 349·1) | -32·3% (-36·8 to -29·1) |  | 372·3 (236·9 to 546·1) | 247·3 (165·0 to 354·2) | -33·6% (-50·7 to -13·8) |  | 11·8 (2·8 to 25·3) | 5·2 (3·1 to 7·9) | -56·0% (-82·2 to 80·2) |  | 1030·0 (242·6 to 2209·9) | 453·6 (272·3 to 687·1) | -56·0% (-82·2 to 79·4) |
| India | 567497·1 (391792·2 to 790633·8) | 609875·3 (414709·4 to 839949·0) | 7·5% (0·1 to 16·7) |  | 565770·0 (382322·4 to 791937·8) | 591480·3 (399111·7 to 836774·1) | 4·5% (-8·4 to 19·2) |  | 10970·9 (5577·6 to 17596·5) | 4884·5 (3764·6 to 6255·8) | -55·5% (-73·0 to -15·1) |  | 956310·6 (486583·9 to 1531552·1) | 428132·6 (330028·6 to 548223·0) | -55·2% (-72·8 to -14·7) |
| Nepal | 11476·5 (7881·8 to 16255·3) | 11773·0 (8166·9 to 16282·8) | 2·6% (-11·7 to 19·7) |  | 11485·1 (7376·1 to 16893·7) | 11073·9 (7282·3 to 16531·2) | -3·6% (-32·9 to 32·0) |  | 458·6 (192·8 to 792·2) | 153·8 (96·6 to 220·9) | -66·5% (-84·4 to -10·0) |  | 40032·4 (16909·2 to 68999·9) | 13489·6 (8487·7 to 19340·6) | -66·3% (-84·3 to -9·6) |
| Pakistan | 84024·6 (59108·5 to 115526·8) | 127887·8 (89121·1 to 175929·4) | 52·2% (46·4 to 57·1) |  | 88885·3 (59199·8 to 125589·9) | 133557·8 (90959·7 to 188055·7) | 50·3% (25·7 to 75·7) |  | 2991·6 (1362·9 to 5162·4) | 3455·7 (2176·5 to 4906·0) | 15·5% (-27·2 to 117·6) |  | 261096·9 (119231·6 to 449832·2) | 302309·5 (191274·8 to 429825·6) | 15·8% (-27·1 to 117·6) |
| **Southeast Asia, East Asia, and Oceania** | |  |  |  |  |  |  |  |  |  |  |  |  |  |  |
| **East Asia** | 1855001·6 (1274020·0 to 2566726·5) | 1440901·5 (1026038·1 to 1954947·4) | -22·3% (-29·5 to -14·0) |  | 2225694·3 (1610738·3 to 3011848·2) | 1555287·1 (1155324·8 to 2098276·1) | -30·1% (-38·8 to -18·3) |  | 25717·3 (19471·2 to 32243·3) | 5041·5 (4100·3 to 6315·9) | -80·4% (-86·2 to -69·0) |  | 2278191·9 (1722797·0 to 2852374·4) | 453423·6 (368042·5 to 569323·0) | -80·1% (-86·0 to -68·5) |
| China | 1798418·8 (1234130·6 to 2486305·4) | 1411849·2 (1004649·0 to 1915646·0) | -21·5% (-28·8 to -12·9) |  | 2157773·1 (1559072·9 to 2918225·5) | 1525106·1 (1135495·5 to 2055076·0) | -29·3% (-38·2 to -17·1) |  | 25048·4 (18974·2 to 31220·8) | 4908·8 (3986·7 to 6161·4) | -80·4% (-86·2 to -68·8) |  | 2218591·9 (1680143·0 to 2758047·8) | 441590·9 (357185·5 to 555561·7) | -80·1% (-86·0 to -68·4) |
| Democratic People's Republic of Korea | 37065·2 (25386·7 to 51405·4) | 229451·7 (165721·9 to 303737·1) | -35·6% (-35·8 to -35·5) |  | 47108·6 (32068·3 to 64546·0) | 241526·0 (168653·5 to 328786·5) | -34·9% (-37·0 to -32·5) |  | 563·3 (312·2 to 866·2) | 64·7 (47·1 to 86·2) | -72·3% (-80·9 to -56·1) |  | 50231·8 (27973·6 to 77087·3) | 5890·5 (4330·9 to 7829·2) | -71·5% (-80·2 to -55·1) |
| Taiwan (Province of China) | 19517·5 (13341·5 to 27596·6) | 8391·8 (6899·8 to 10264·7) | -57·0% (-67·8 to -43·9) |  | 20812·6 (14829·9 to 29348·1) | 9267·8 (7433·5 to 11516·4) | -55·5% (-67·2 to -38·9) |  | 105·6 (93·4 to 120·2) | 36·0 (27·8 to 44·6) | -65·9% (-74·3 to -56·1) |  | 9368·3 (8301·2 to 10667·6) | 3268·4 (2499·8 to 4117·9) | -65·1% (-73·8 to -54·8) |
| **Oceania** | 9358·9 (6385·4 to 13082·6) | 17628·8 (12003·0 to 24665·0) | 88·4% (86·4 to 90·2) |  | 9332·6 (6276·5 to 13174·9) | 17678·2 (11917·1 to 25286·1) | 89·4% (70·9 to 109·7) |  | 80·0 (47·2 to 122·8) | 148·9 (88·1 to 229·4) | 86·0% (25·7 to 183·7) |  | 7026·0 (4132·6 to 10750·4) | 13080·6 (7741·0 to 20148·5) | 86·2% (25·7 to 183·7) |
| American Samoa | 70·8 (48·0 to 99·3) | 48·8 (33·1 to 68·3) | -31·1% (-31·5 to -30·5) |  | 68·0 (44·9 to 97·4) | 47·5 (31·2 to 68·1) | -30·1% (-38·2 to -21·1) |  | 0·3 (0·2 to 0·5) | 0·2 (0·1 to 0·3) | -44·1% (-69·1 to 2·3) |  | 29·4 (20·7 to 40·3) | 16·4 (9·6 to 25·9) | -44·0% (-69·0 to 2·4) |
| Cook Islands | 22·4 (15·2 to 31·4) | 12·6 (8·6 to 17·7) | -43·6% (-44·1 to -43·2) |  | 22·3 (14·8 to 31·9) | 12·3 (8·0 to 18·0) | -44·7% (-53·0 to -35·1) |  | 0·1 (0·1 to 0·1) | 0·0 (0·0 to 0·0) | -77·8% (-92·2 to -58·1) |  | 7·5 (4·9 to 11·0) | 1·7 (0·6 to 2·8) | -77·7% (-92·1 to -57·6) |
| Fiji | 900·8 (613·4 to 1263·4) | 846·5 (576·2 to 1182·1) | -6·0% (-6·9 to -4·7) |  | 875·9 (573·1 to 1276·8) | 845·3 (560·0 to 1193·5) | -3·5% (-14·8 to 10·6) |  | 6·0 (3·9 to 8·8) | 7·2 (4·8 to 10·7) | 19·8% (-28·3 to 100·2) |  | 528·1 (344·0 to 771·8) | 631·8 (419·5 to 934·2) | 19·6% (-28·2 to 100·6) |
| Guam | 153·5 (103·9 to 215·3) | 150·6 (102·6 to 210·6) | -1·9% (-2·5 to -0·9) |  | 149·4 (102·3 to 214·9) | 152·6 (102·6 to 216·9) | 2·1% (-10·5 to 18·6) |  | 0·6 (0·5 to 0·8) | 0·7 (0·5 to 1·1) | 20·2% (-17·8 to 85·8) |  | 54·2 (41·5 to 69·3) | 65·4 (44·8 to 97·6) | 20·6% (-17·6 to 86·0) |
| Kiribati | 109·4 (74·7 to 152·3) | 138·9 (94·8 to 194·5) | 27·0% (24·8 to 29·1) |  | 109·2 (74·1 to 155·2) | 137·4 (92·5 to 197·9) | 25·9% (9·2 to 44·5) |  | 1·0 (0·7 to 1·4) | 1·0 (0·6 to 1·6) | -3·9% (-50·7 to 77·0) |  | 88·4 (62·9 to 120·9) | 85·0 (49·2 to 144·0) | -3·8% (-50·5 to 76·7) |
| Marshall Islands | 71·9 (48·8 to 100·5) | 57·5 (39·1 to 80·5) | -19·9% (-20·4 to -19·6) |  | 70·2 (45·8 to 101·1) | 55·5 (36·3 to 80·9) | -20·9% (-30·3 to -10·4) |  | 0·3 (0·2 to 0·5) | 0·3 (0·2 to 0·4) | -22·2% (-53·5 to 23·3) |  | 29·2 (20·0 to 41·2) | 22·7 (13·8 to 33·0) | -22·2% (-53·7 to 23·2) |
| Micronesia (Federated States of) | 158·1 (107·3 to 221·2) | 91·9 (62·5 to 128·6) | -41·9% (-42·3 to -41·4) |  | 154·3 (101·2 to 224·3) | 88·6 (58·4 to 127·5) | -42·6% (-49·4 to -34·5) |  | 0·9 (0·6 to 1·3) | 0·3 (0·1 to 0·5) | -62·3% (-85·9 to -30·1) |  | 80·0 (52·6 to 112·8) | 30·2 (11·0 to 47·2) | -62·3% (-85·7 to -30·0) |
| Nauru | 17·7 (12·2 to 24·8) | 13·4 (9·2 to 18·8) | -24·3% (-25·4 to -23·3) |  | 18·5 (12·5 to 26·1) | 13·9 (9·5 to 20·2) | -24·4% (-34·7 to -12·1) |  | 0·2 (0·1 to 0·3) | 0·1 (0·1 to 0·2) | -40·0% (-59·3 to -4·8) |  | 17·3 (10·0 to 25·8) | 10·4 (7·1 to 14·6) | -39·8% (-59·2 to -4·5) |
| Niue | 2·6 (1·8 to 3·7) | 1·2 (0·8 to 1·7) | -53·8% (-54·3 to -53·1) |  | 2·7 (1·8 to 3·8) | 1·3 (0·9 to 1·9) | -51·5% (-57·5 to -43·7) |  | 0·0 (0·0 to 0·0) | 0·0 (0·0 to 0·0) | -50·4% (-67·8 to -25·1) |  | 1·7 (1·1 to 2·4) | 0·9 (0·5 to 1·3) | -50·2% (-67·5 to -24·8) |
| Northern Mariana Islands | 47·6 (32·3 to 66·9) | 21·9 (14·9 to 30·6) | -54·1% (-54·5 to -53·5) |  | 46·1 (29·9 to 65·3) | 22·4 (15·0 to 31·9) | -51·4% (-57·9 to -43·5) |  | 0·3 (0·2 to 0·4) | 0·1 (0·1 to 0·2) | -46·6% (-66·6 to -15·6) |  | 22·6 (16·5 to 31·8) | 12·1 (7·3 to 18·5) | -46·5% (-66·3 to -15·2) |
| Palau | 15·9 (10·8 to 22·2) | 9·4 (6·4 to 13·2) | -40·7% (-42·1 to -39·5) |  | 16·5 (11·3 to 23·3) | 10·0 (6·8 to 14·1) | -39·6% (-49·1 to -29·6) |  | 0·1 (0·1 to 0·3) | 0·1 (0·0 to 0·1) | -61·2% (-79·5 to -26·1) |  | 12·1 (6·8 to 22·7) | 4·7 (3·2 to 6·8) | -61·0% (-79·3 to -25·3) |
| Papua New Guinea | 6113·2 (4172·3 to 8552·8) | 13815·9 (9400·4 to 19337·1) | 126·0% (122·6 to 128·6) |  | 6145·4 (4063·0 to 8727·0) | 13900·0 (9324·4 to 19866·5) | 126·2% (97·3 to 158·4) |  | 57·7 (27·7 to 96·8) | 122·6 (67·8 to 197·5) | 112·3% (34·1 to 262·6) |  | 5069·1 (2434·9 to 8469·2) | 10769·8 (5963·2 to 17341·3) | 112·5% (34·2 to 262·2) |
| Samoa | 191·9 (130·8 to 268·4) | 197·7 (134·2 to 276·9) | 3·0% (2·0 to 4·0) |  | 195·5 (132·8 to 281·1) | 195·9 (129·2 to 286·1) | -47·8% (-74·8 to 0·0) |  | 1·2 (0·8 to 1·8) | 0·6 (0·3 to 1·1) | -48·2% (-75·1 to -0·7) |  | 108·6 (67·2 to 160·3) | 56·7 (29·0 to 99·5) | -47·8% (-74·8 to 0·0) |
| Solomon Islands | 577·9 (391·6 to 809·0) | 906·2 (615·3 to 1268·7) | 56·8% (55·5 to 57·8) |  | 559·9 (368·8 to 805·9) | 875·8 (586·3 to 1264·1) | 56·4% (38·0 to 76·1) |  | 4·7 (2·8 to 7·2) | 6·4 (4·2 to 8·9) | 36·2% (-22·6 to 137·8) |  | 408·2 (246·4 to 626·6) | 555·4 (369·4 to 780·1) | 36·0% (-22·7 to 137·5) |
| Tokelau | 2·8 (1·9 to 4·0) | 1·7 (1·1 to 2·3) | -41·8% (-42·4 to -41·4) |  | 2·8 (1·9 to 4·0) | 1·6 (1·1 to 2·4) | -43·3% (-50·7 to -35·4) |  | 0·0 (0·0 to 0·0) | 0·0 (0·0 to 0·0) | -65·7% (-78·6 to -45·0) |  | 1·4 (0·9 to 2·0) | 0·5 (0·3 to 0·7) | -65·6% (-78·5 to -44·8) |
| Tonga | 127·4 (86·7 to 177·7) | 117·4 (80·6 to 163·2) | -7·8% (-10·5 to -3·5) |  | 131·1 (84·3 to 191·3) | 130·2 (83·8 to 194·2) | -0·7% (-24·9 to 43·7) |  | 0·9 (0·6 to 1·2) | 0·7 (0·5 to 1·1) | -17·9% (-49·6 to 29·4) |  | 77·0 (54·9 to 103·7) | 63·8 (40·5 to 96·3) | -17·2% (-49·0 to 30·5) |
| Tuvalu | 12·8 (8·8 to 17·9) | 10·1 (6·9 to 14·2) | -20·8% (-22·6 to -19·6) |  | 13·1 (9·0 to 18·7) | 9·7 (6·5 to 14·2) | -26·0% (-38·4 to -13·8) |  | 0·1 (0·1 to 0·2) | 0·0 (0·0 to 0·1) | -74·0% (-87·3 to -40·3) |  | 11·9 (6·6 to 19·2) | 3·1 (1·8 to 5·0) | -74·0% (-87·3 to -39·9) |
| Vanuatu | 244·2 (165·5 to 342·3) | 354·3 (240·4 to 496·1) | 45·0% (44·3 to 45·9) |  | 235·3 (156·9 to 339·2) | 343·2 (224·8 to 496·0) | 45·8% (28·3 to 65·8) |  | 1·0 (0·7 to 1·5) | 1·5 (0·9 to 2·1) | 46·7% (-10·8 to 137·4) |  | 90·4 (58·9 to 132·2) | 132·3 (82·6 to 187·9) | 46·5% (-11·1 to 137·5) |
| **Southeast Asia** | 244·2 (165·5 to 342·3) | 354·3 (240·4 to 496·1) | 45·0% (44·3 to 45·9) |  | 439125·7 (297133·5 to 598878·7) | 351025·8 (251903·3 to 475136·4) | -20·1% (-30·8 to -8·7) |  | 6521·3 (3364·9 to 11609·8) | 3317·7 (2570·5 to 4420·2) | -49·1% (-66·3 to -5·5) |  | 571478·2 (294997·5 to 1015700·0) | 291039·2 (225426·3 to 388310·5) | -49·1% (-66·2 to -5·2) |
| Cambodia | 406546·7 (282172·2 to 555882·9) | 345854·3 (243199·7 to 468954·4) | -14·9% (-18·0 to -11·3) |  | 13765·2 (8403·6 to 19596·1) | 11027·5 (7520·0 to 15393·1) | -19·9% (-41·4 to 6·5) |  | 378·9 (146·7 to 674·5) | 166·5 (107·0 to 248·9) | -56·1% (-77·9 to 19·4) |  | 33293·6 (12872·1 to 59284·9) | 14653·1 (9430·9 to 21938·0) | -56·0% (-77·9 to 19·6) |
| Indonesia | 12201·8 (8502·5 to 16510·9) | 11075·1 (7632·2 to 15136·5) | -9·2% (-14·3 to -5·3) |  | 193156·2 (128108·7 to 265473·7) | 135438·4 (97618·0 to 181787·7) | -29·9% (-41·1 to -16·3) |  | 3051·1 (1478·2 to 5192·2) | 1267·5 (927·6 to 1759·7) | -58·5% (-74·5 to -16·3) |  | 267315·5 (129511·5 to 454751·4) | 110974·3 (81290·8 to 154094·4) | -58·5% (-74·5 to -16·4) |
| Lao People's Democratic Republic | 172489·4 (118733·6 to 236483·5) | 132531·1 (94124·1 to 177917·6) | -23·2% (-28·8 to -16·2) |  | 5290·4 (3077·3 to 7893·0) | 4936·0 (3358·0 to 6934·3) | -6·7% (-34·8 to 31·0) |  | 121·1 (33·7 to 228·8) | 44·4 (25·4 to 69·9) | -63·3% (-80·8 to 19·1) |  | 10616·5 (2943·6 to 20143·9) | 3909·7 (2231·1 to 6171·4) | -63·2% (-80·8 to 19·0) |
| Malaysia | 4503·4 (3123·1 to 6142·7) | 4996·7 (3450·3 to 6882·2) | 11·0% (3·1 to 16·4) |  | 14365·6 (9640·1 to 19968·7) | 14800·4 (9849·5 to 20715·6) | 3·0% (-11·9 to 20·5) |  | 123·2 (76·0 to 182·4) | 58·6 (30·3 to 106·3) | -52·5% (-76·6 to -4·2) |  | 10808·6 (6664·1 to 15982·7) | 5183·1 (2675·5 to 9353·7) | -52% (-76·3 to -3·7) |
| Maldives | 14850·3 (10230·2 to 20375·3) | 15802·7 (10874·5 to 21771·4) | 6·4% (5·0 to 7·7) |  | 290·3 (191·4 to 413·2) | 267·1 (184·8 to 367·8) | -8·0% (-32·0 to 19·2) |  | 5·2 (2·2 to 9·4) | 2·2 (1·5 to 3·3) | -56·7% (-78·1 to 8·0) |  | 452·5 (195·5 to 819·3) | 197·5 (130·4 to 288·7) | -56·3% (-77·9 to 8·2) |
| Mauritius | 264·7 (184·4 to 359·6) | 258·6 (178·5 to 353·2) | -2·3% (-7·1 to 1·0) |  | 721·6 (505·9 to 987·9) | 447·9 (314·8 to 608·4) | -37·9% (-47·1 to -26·9) |  | 6·0 (4·9 to 7·3) | 3·4 (2·7 to 4·2) | -42·8% (-57·9 to -24·3) |  | 527·1 (435·6 to 638·2) | 301·8 (238·6 to 377·5) | -42·7% (-57·8 to -24·0) |
| Myanmar | 662·9 (462·7 to 905·1) | 407·8 (283·7 to 557·6) | -38·5% (-39·3 to -37·6) |  | 44931·0 (25876·3 to 70064·5) | 33776·6 (23920·6 to 45776·5) | -24·8% (-48·6 to 4·5) |  | 996·9 (338·6 to 2264·8) | 515·6 (284·0 to 873·3) | -48·3% (-76·0 to 46·5) |  | 87361·6 (29541·3 to 199967·3) | 45253·0 (24952·8 to 76538·0) | -48·2% (-75·9 to 46·2) |
| Philippines | 37565·9 (26012·2 to 51051·0) | 31815·0 (22179·7 to 43644·2) | -15·3% (-21·7 to -11·3) |  | 65424·6 (45160·4 to 89074·5) | 76208·6 (53684·8 to 104582·2) | 16·5% (0·1 to 34·8) |  | 927·6 (534·4 to 1722·2) | 812·7 (617·4 to 1107·4) | -12·4% (-44·4 to 51·8) |  | 81133·8 (46620·7 to 150744·9) | 71146·3 (54039·9 to 96828·2) | -12·3% (-44·2 to 51·8) |
| Seychelles | 61283·8 (42079·6 to 83952·9) | 75106·6 (51979·2 to 103278·4) | 22·6% (14·4 to 32·0) |  | 47·8 (31·9 to 66·7) | 45·2 (30·4 to 63·1) | -5·5% (-19·2 to 10·0) |  | 0·4 (0·3 to 0·5) | 0·3 (0·2 to 0·4) | -20·0% (-46·7 to 18·5) |  | 33·6 (24·1 to 44·9) | 27·0 (19·2 to 38·0) | -19·7% (-46·5 to 18·7) |
| Sri Lanka | 50·7 (34·9 to 69·7) | 46·3 (31·9 to 63·5) | -8·6% (-9·3 to -7·7) |  | 11197·8 (7564·8 to 15518·1) | 9770·7 (6760·4 to 13702·2) | -12·7% (-26·7 to 4·0) |  | 88·6 (61·9 to 117·0) | 47·5 (31·7 to 67·0) | -46·4% (-67·9 to -12·6) |  | 7794·2 (5449·4 to 10258·8) | 4212·6 (2798·3 to 5929·1) | -46·0% (-67·5 to -11·9) |
| Thailand | 11256·9 (7734·6 to 15488·9) | 9627·7 (6639·2 to 13214·0) | -14·5% (-15·6 to -13·3) |  | 35590·9 (24819·1 to 49052·6) | 21413·0 (14951·8 to 29344·3) | -39·8% (-50·2 to -26·8) |  | 298·4 (196·3 to 420·1) | 114·9 (56·9 to 158·0) | -61·5% (-81·9 to -31·5) |  | 26419·0 (17418·9 to 37201·7) | 10269·2 (5107·3 to 14140·1) | -61·1% (-81·7 to -31·3) |
| Timor-Leste | 33765·9 (23424·5 to 46209·7) | 19683·2 (13615·1 to 26994·5) | -41·7% (-43·0 to -40·5) |  | 1053·1 (607·0 to 1586·3) | 1086·3 (740·8 to 1502·9) | 3·2% (-27·8 to 44·5) |  | 21·0 (5·0 to 41·3) | 10·0 (2·8 to 16·0) | -52·5% (-86·9 to 83·7) |  | 1839·4 (434·2 to 3628·0) | 875·4 (251·5 to 1410·8) | -52·4% (-86·8 to 84·0) |
| Viet Nam | 915·0 (634·4 to 1244·0) | 1096·3 (756·5 to 1500·5) | 19·8% (12·1 to 25·8) |  | 52707·6 (34814·1 to 74314·9) | 41348·3 (27534·8 to 57661·2) | -21·6% (-34·6 to -4·3) |  | 494·4 (251·5 to 935·1) | 269·7 (174·3 to 410·6) | -45·5% (-67·8 to 11·8) |  | 43123·2 (21965·2 to 81403·0) | 23654·8 (15266·4 to 36037·9) | -45·1% (-67·6 to 12·5) |
| **Sub-Saharan Africa** |  |  |  |  |  |  |  |  |  |  |  |  |  |  |  |
| **Central Sub-Saharan Africa** | 850933·1 (590003·9 to 1167991·2) | 1518699·6 (1049263·4 to 2107385·1) | 78·5% (74·6 to 81·7) |  | 109946·0 (75243·2 to 154050·5) | 201279·8 (136108·1 to 281763·9) | 83·1% (56·1 to 108·6) |  | 1574·2 (632·0 to 2877·2) | 1170·0 (719·1 to 1786·3) | -25·7% (-53·1 to 56·5) |  | 137456·6 (55224·3 to 251794·8) | 102328·8 (63006·8 to 156026·7) | -25·6% (-53·0 to 56·9) |
| Angola | 108869·7 (74995·3 to 150287·9) | 206942·3 (141646·2 to 288292·3) | 90·1% (85·4 to 93·5) |  | 20571·5 (13409·8 to 29098·7) | 50562·3 (33356·8 to 71767·0) | 145·8% (98·6 to 193·1) |  | 334·8 (131·3 to 688·8) | 377·6 (219·1 to 595·3) | 12·8% (-39·2 to 125·6) |  | 29198·2 (11467·5 to 59904·4) | 32982·0 (19178·0 to 51852·0) | 13·0% (-39·3 to 126·3) |
| Central African Republic | 20077·4 (13805·9 to 27649·0) | 51662·4 (35636·2 to 71855·9) | 157·3% (147·8 to 163·0) |  | 5077·0 (3405·9 to 7183·2) | 8348·2 (5545·3 to 11723·0) | 64·4% (43·8 to 86·4) |  | 61·7 (27·9 to 122·4) | 78·9 (34·8 to 146·0) | 27·9% (-14·1 to 105·0) |  | 5389·6 (2445·6 to 10650·8) | 6887·8 (3037·4 to 12740·1) | 27·8% (-14·0 to 104·7) |
| Congo | 5094·8 (3484·8 to 7048·1) | 8456·3 (5792·1 to 11739·9) | 66·0% (63·3 to 67·8) |  | 4252·7 (2894·6 to 6002·1) | 6709·6 (4517·9 to 9554·0) | 57·8% (36·5 to 81·4) |  | 41·9 (22·3 to 83·9) | 28·2 (16·4 to 47·5) | -32·6% (-62·9 to 29·8) |  | 3654·8 (1952·8 to 7308·8) | 2466·0 (1437·2 to 4152·6) | -32·5% (-62·8 to 29·5) |
| Democratic Republic of the Congo | 4317·0 (2963·7 to 5990·4) | 6942·8 (4750·5 to 9656·1) | 60·8% (57·9 to 62·7) |  | 77504·0 (52633·5 to 108685·1) | 131955·6 (88038·5 to 185326·8) | 70·3% (41·4 to 100·4) |  | 1109·9 (414·2 to 2063·1) | 669·9 (367·3 to 1095·6) | -39·6% (-68·2 to 57·4) |  | 96952·4 (36377·0 to 180674·4) | 58638·7 (32086·7 to 95677·7) | -39·5% (-68·1 to 57·1) |
| Equatorial Guinea | 76793·0 (52993·3 to 106105·3) | 136022·1 (92987·8 to 189573·4) | 77·1% (72·6 to 80·6) |  | 882·3 (600·7 to 1234·4) | 1766·5 (1196·2 to 2493·8) | 100·2% (69·8 to 133·8) |  | 9·3 (4·1 to 18·3) | 6·3 (3·0 to 11·2) | -32·7% (-73·4 to 60·1) |  | 814·6 (361·3 to 1597·8) | 550·2 (259·7 to 980·7) | -32·5% (-73·3 to 61·2) |
| Gabon | 898·4 (614·3 to 1244·7) | 1839·5 (1260·4 to 2566·3) | 104·8% (99·8 to 108·3) |  | 1658·5 (1085·8 to 2303·0) | 1937·5 (1292·8 to 2738·0) | 16·8% (1·4 to 33·4) |  | 16·6 (7·5 to 29·1) | 9·2 (4·7 to 15·5) | -44·5% (-71·0 to 20·0) |  | 1446·9 (657·8 to 2537·6) | 804·0 (407·0 to 1352·9) | -44·4% (-70·9 to 20·0) |
| **Eastern Sub-Saharan Africa** | 1689·1 (1160·0 to 2348·7) | 2019·2 (1384·7 to 2821·8) | 19·5% (17·7 to 21·0) |  | 271085·8 (177382·7 to 375820·2) | 425539·4 (295536·4 to 589005·1) | 57·0% (24·1 to 91·1) |  | 12183·8 (6832·8 to 18741·7) | 9947·2 (7274·8 to 13169·3) | 57·0% (24·1 to 91·1) |  | 1065738·6 (598277·8 to 1639030·4) | 873050·7 (638941·3 to 1152792·5) | -18·1% (-50·0 to 51·3) |
| Burundi | 233076·3 (162684·5 to 318687·0) | 400974·6 (276930·6 to 557068·1) | 72·0% (61·6 to 80·4) |  | 7312·8 (4888·5 to 10450·1) | 12974·2 (8540·1 to 18713·7) | 77·4% (37·3 to 124·3) |  | 297·3 (167·3 to 471·3) | 239·9 (126·2 to 444·8) | -19·3% (-65·2 to 78·1) |  | 25939·2 (14651·5 to 40882·0) | 21078·9 (11145·3 to 39123·0) | -18·7% (-64·8 to 79·8) |
| Comoros | 6755·0 (4657·1 to 9301·1) | 12565·7 (8637·3 to 17445·9) | 86·0% (78·2 to 93·8) |  | 539·3 (371·7 to 742·9) | 486·4 (325·9 to 699·7) | -9·8% (-26·0 to 9·3) |  | 14·2 (6·9 to 21·3) | 7·9 (4·7 to 13·0) | -44·0% (-69·5 to 29·2) |  | 1246·4 (612·5 to 1864·3) | 699·6 (416·5 to 1147·8) | -43·9% (-69·5 to 29·0) |
| Djibouti | 502·3 (347·4 to 695·2) | 471·9 (323·8 to 657·2) | -6·1% (-8·7 to -2·8) |  | 603·9 (422·0 to 830·3) | 1057·3 (724·4 to 1481·3) | 75·1% (44·5 to 114·5) |  | 17·2 (11·8 to 25·0) | 26·0 (17·4 to 37·5) | 51·2% (-10·0 to 155·1) |  | 1507·3 (1039·5 to 2186·1) | 2280·1 (1527·6 to 3298·5) | 51·3% (-9·5 to 155·5) |
| Eritrea | 556·2 (385·2 to 766·1) | 974·6 (676·3 to 1350·7) | 75·2% (70·3 to 80·7) |  | 3521·1 (2397·8 to 5023·5) | 5798·4 (3748·1 to 8329·8) | 64·7% (32·6 to 104·6) |  | 113·0 (69·4 to 169·3) | 125·2 (80·2 to 195·9) | 10·8% (-40·1 to 111·2) |  | 9854·1 (6070·0 to 14780·6) | 10960·8 (7024·4 to 17253·5) | 11·2% (-39·9 to 110·8) |
| Ethiopia | 3382·5 (2326·6 to 4688·5) | 5629·4 (3874·4 to 7810·0) | 66·4% (61·4 to 72·4) |  | 91826·6 (51214·0 to 145338·6) | 116973·1 (79382·3 to 164364·5) | 27·4% (-15·3 to 77·0) |  | 4889·5 (1519·3 to 10036·2) | 2812·5 (1644·7 to 4331·9) | -42·5% (-67·9 to 56·7) |  | 429082·4 (133871·5 to 880678·6) | 247129·6 (144327·1 to 380698·9) | -42·4% (-67·8 to 56·9) |
| Kenya | 69933·5 (48573·3 to 95176·0) | 109387·9 (76924·3 to 150342·2) | 56·4% (35·6 to 70·6) |  | 29167·6 (19969·8 to 40795·1) | 40857·8 (27700·0 to 57778·5) | 40·1% (27·4 to 51·8) |  | 419·7 (239·0 to 609·2) | 349·6 (247·4 to 477·9) | -16·7% (-52·4 to 62·2) |  | 36990·4 (21167·9 to 53516·1) | 30841·7 (21844·5 to 42061·3) | -16·6% (-52·4 to 62·2) |
| Madagascar | 28026·7 (19451·5 to 38978·1) | 40901·3 (28191·4 to 56843·9) | 45·9% (43·2 to 48·4) |  | 14728·7 (9915·3 to 20182·8) | 23700·2 (15928·9 to 33751·2) | 60·9% (30·4 to 96·7) |  | 507·8 (343·3 to 729·8) | 331·3 (221·4 to 476·3) | -34·8% (-62·4 to 9·9) |  | 44438·6 (30208·5 to 63498·0) | 29155·9 (19480·5 to 41584·3) | -34·4% (-62·2 to 10·4) |
| Malawi | 13507·4 (9338·1 to 18732·7) | 23573·4 (16165·6 to 32777·2) | 74·5% (69·3 to 78·7) |  | 11965·1 (8043·0 to 16796·1) | 15501·8 (10404·0 to 22207·3) | 29·6% (3·3 to 58·1) |  | 1359·5 (748·5 to 2116·6) | 643·9 (419·3 to 962·3) | -52·6% (-73·1 to -9·5) |  | 117717·3 (65063·9 to 182932·9) | 55906·8 (36444·0 to 83404·2) | -52·5% (-73·0 to -9·2) |
| Mozambique | 11172·6 (7750·6 to 15601·1) | 15584·6 (10710·9 to 21601·2) | 39·5% (33·7 to 44·4) |  | 17602·6 (11636·5 to 24552·6) | 36558·7 (24698·8 to 51152·2) | 107·7% (48·8 to 185·0) |  | 752·7 (451·0 to 1110·5) | 952·1 (565·9 to 1589·9) | 26·5% (-32·2 to 155·4) |  | 65925·6 (39409·6 to 97016·8) | 84113·1 (49943·7 to 140509·4) | 27·6% (-31·4 to 158·0) |
| Rwanda | 14672·2 (10219·9 to 20156·8) | 31708·9 (21938·2 to 43840·3) | 116·1% (103·1 to 130·4) |  | 8778·7 (5900·6 to 12161·2) | 10546·4 (7041·3 to 14901·0·) | 20·1% (-4·5 to 51·5) |  | 340·3 (196·8 to 528·0) | 236·0 (144·0 to 352·1) | -30·7% (-65·1 to 43·8) |  | 29659·9 (17252·6 to 45828·1) | 20686·4 (12625·7 to 31046·2) | -30·3% (-64·8 to 45·3) |
| Somalia | 8299·1 (5721·4 to 11528·7) | 10047·0 (6885·3 to 13907·9) | 21·1% (16·3 to 26·4) |  | 8740·6 (5960·3 to 12123·5) | 23940·6 (16069·3 to 34436·9) | 173·9% (121·6 to 235·3) |  | 323·2 (182·5 to 526·9) | 527·7 (339·5 to 763·0) | 63·2% (-7·3 to 188·4) |  | 28237·7 (15960·9 to 45969·7) | 46349·5 (29960·1 to 66933·2) | 64·1% (-6·1 to 189·9) |
| South Sudan | 7946·0 (5453·5 to 11043·5) | 22635·3 (15546·3 to 31466·6) | 184·9% (174·4 to 194·8) |  | 7628·2 (5052·0 to 10668·0) | 10222·2 (6879·0 to 14303·0) | 34·0% (1·5 to 71·2) |  | 354·7 (202·2 to 543·0) | 291·7 (190·6 to 421·6) | -17·8% (-48·2 to 37·4) |  | 31028·9 (17708·2 to 47356·8) | 25606·3 (16859·6 to 36977·1) | -17·5% (-47·9 to 37·9) |
| Uganda | 6359·6 (4453·0 to 8771·8) | 9334·0 (6436·6 to 12901·5) | 46·8% (39·1 to 52·9) |  | 22372·7 (15039·5 to 31557·8) | 43732·9 (29238·9 to 62288·0) | 95·5% (61·8 to 136·4) |  | 765·6 (441·1 to 1239·6) | 983·5 (586·1 to 1462·3) | 28·5% (-27·2 to 130·4) |  | 66722·6 (38594·3 to 107751·5) | 85998·4 (51352·7 to 127598·5) | 28·9% (-26·9 to 131·1) |
| United Republic of Tanzania | 21857·9 (15007·3 to 30368·7) | 42814·1 (29382·4 to 59554·2) | 95·9% (91·0 to 101·0) |  | 35209·4 (23510·9 to 48161·4) | 64940·5 (44082·6 to 89805·7) | 84·4% (44·8 to 144·8) |  | 1468·5 (936·0 to 2149·4) | 2013·4 (1365·7 to 2882·3) | 37·1% (-17·3 to 136·6) |  | 128493·3 (82315·2 to 187809·9) | 176587·9 (119776·2 to 252534·4) | 37·4% (-16·9 to 137·3) |
| Zambia | 30421·0 (21188·4 to 42024·3) | 57856·0 (40098·9 to 79720·0) | 90·2% (81·8 to 100·4) |  | 10889·5 (7237·0 to 15075·4) | 17908·6 (12121·9 to 25296·6) | 64·5% (23·6 to 113·3) |  | 551·6 (327·9 to 828·9) | 398·6 (261·6 to 601·4) | -27·7% (-60·2 to 47·1) |  | 48112·9 (28632·8 to 72274·0) | 34957·6 (22958·9 to 52662·6) | -27·3% (-59·9 to 47·9) |
| **Southern Sub-Saharan Africa** | 9513·3 (6572·4 to 13070·4) | 17169·8 (11857 to 23802·9) | 80·5% (71·7 to 89·3) |  | 141277·6 (96292·8 to 196962·7) | 159523·2 (107914·0 to 221510·8) | 12·9% (9·6 to 16·6) |  | 259·7 (160·4 to 372·1) | 188·3 (139·3 to 247·8) | -27·5% (-51·5 to 14·8) |  | 22790·5 (14131·0 to 32564·2) | 16617·7 (12289·0 to 21860·2) | -27·1% (-51·2 to 15·3) |
| Botswana | 139386·8 (96946·1 to 189237·4) | 157209·4 (109266·5 to 213412·3) | 12·8% (12·5 to 13·1) |  | 3693·8 (2462·9 to 5277·0) | 4541·7 (3156·6 to 6382·2) | 23·0% (13·1 to 35·2) |  | 3·3 (2·0 to 4·9) | 8·0 (4·7 to 12·6) | 143·7% (48·3 to 303·7) |  | 290·3 (173·0 to 425·7) | 707·6 (412·3 to 1113·1) | 143·8% (48·2 to 303·3) |
| Eswatini | 3580·9 (2467·1 to 4912·5) | 4363·0 (3007·9 to 5976·7) | 21·8% (21·5 to 22·4) |  | 2729·0 (1821·2 to 3873·3) | 2654·5 (1761·3 to 3756·7) | -2·7% (-11·3 to 5·8) |  | 5·2 (2·8 to 9·0) | 4·7 (3·1 to 6·7) | -9·8% (-42·5 to 54·6) |  | 453·5 (243·4 to 785·5) | 410·1 (275·0 to 589·0) | -9·6% (-42·1 to 54·8) |
| Lesotho | 2662·1 (1834·5 to 3656·9) | 2586·2 (1787·0 to 3542·0) | -2·9% (-3·2 to -2·5) |  | 5053·2 (3384·9 to 7228·7) | 4123·9 (2773·5 to 5915·3) | -18·4% (-25·3 to -11·4) |  | 5·6 (3·7 to 7·8) | 6·4 (4·3 to 9·3) | 13·7% (-22·8 to 68·9) |  | 492·5 (327·5 to 682·8) | 558·9 (376·2 to 818·2) | 13·5% (-23·0 to 68·3) |
| Namibia | 4960·4 (3417·7 to 6808·7) | 4019·0 (2773·1 to 5511·6) | -19·0% (-19·2 to -18·7) |  | 4161·1 (2727·4 to 5944·2) | 5596·5 (3709·5 to 7976·9) | 34·5% (22·1 to 48·3) |  | 5·9 (2·9 to 9·9) | 8·4 (4·5 to 12·8) | 42·3% (-31·0 to 212·7) |  | 518·5 (253·0 to 866·0) | 740·5 (400·9 to 1126·1) | 42·8% (-30·7 to 211·7) |
| South Africa | 4056·8 (2794·2 to 5565·7) | 5459·4 (3764·7 to 7486·1) | 34·6% (34·3 to 34·9) |  | 92300·4 (62994·1 to 128529·2) | 102763·4 (69780·3 to 142074·0) | 11·3% (7·7 to 15·1) |  | 192·0 (111·3 to 283·9) | 103·7 (75·2 to 137·7) | -46·0% (-65·0 to -5·6) |  | 16826·0 (9790·7 to 24826·8) | 9167·4 (6662·5 to 12179·7) | -45·5% (-64·6 to -5·1) |
| Zimbabwe | 91700·2 (63882·0 to 123888·5) | 102014·3 (71177·2 to 137717·4) | 11·2% (11·0 to 11·5) |  | 33340·2 (22532·3 to 47680·4) | 39843·3 (26590·0 to 56567·0) | 19·5% (9·1 to 29·6) |  | 47·7 (29·0 to 73·6) | 57·2 (37·9 to 80·0) | 19·8% (-29·6 to 102·9) |  | 4209·6 (2562·0 to 6446·4) | 5033·1 (3348·7 to 7004·5) | 19·6% (-29·7 to 101·8) |
| **Western Sub-Saharan Africa** | 32426·4 (22359·4 to 44459·5) | 38767·6 (26734·7 to 53134·3) | 19·6% (19·3 to 19·8) |  | 353089·5 (243121·5 to 491073·0) | 727667·4 (499721·2 to 1014821·1) | 106·1% (99·4 to 111·9) |  | 4130·2 (2443·1 to 6458·5) | 6257·0 (3859·8 to 9157·0) | 51·5% (8·2 to 110·6) |  | 360403·1 (214134·7 to 563352·0) | 546771·3 (337876·6 to 800744·5) | 51·7% (8·3 to 110·6) |
| Benin | 369600·2 (254439·4 to 512421·4) | 753573·2 (521225·4 to 1045241·2) | 103·9% (101·9 to 105·3) |  | 9652·8 (6585·0 to 13533·7) | 21979·3 (14976·2 to 31060·4) | 127·7% (105·6 to 151·7) |  | 127·6 (74·8 to 190·8) | 240·3 (135·7 to 393·8) | 88·3% (18·9 to 185·5) |  | 11167·1 (6552·5 to 16677·9) | 21036·7 (11914·9 to 34377·7) | 88·4% (19·1 to 185·9) |
| Burkina Faso | 9933·6 (6821·0 to 13800·2) | 22407·5 (15383·7 to 31204·5) | 125·6% (123·1 to 128·0) |  | 18048·7 (11901·1 to 25479·4) | 40519·3 (26812·0 to 56133·8) | 124·5% (98·0 to 156·2) |  | 222·5 (123·1 to 358·2) | 581·7 (335·2 to 958·1) | 161·4% (65·6 to 315·2) |  | 19449·7 (10786·2 to 31258·5) | 50965·2 (29480·0 to 83871·7) | 162·0% (65·9 to 315·5) |
| Cabo Verde | 18715·9 (12805·9 to 26017·7) | 40895·0 (28113·1 to 56616·1) | 118·5% (116·0 to 122·0) |  | 574·9 (390·7 to 817·9) | 526·9 (357·0 to 729·9) | -8·3% (-19·5 to 4·3) |  | 5·4 (3·5 to 7·6) | 3·2 (1·6 to 5·0) | -40·0% (-69·8 to 10·6) |  | 478·0 (312·6 to 671·4) | 286·3 (143·7 to 437·8) | -40·1% (-69·8 to 10·4) |
| Cameroon | 577·9 (398·3 to 805·2) | 532·6 (368·1 to 742·0) | -7·8% (-8·8 to -7·0) |  | 18610·2 (12269·8 to 26180·3) | 40846·5 (27074·8 to 56902·6) | 119·5% (92·3 to 149·6) |  | 128·6 (73·3 to 224·7) | 278·8 (164·2 to 420·1) | 116·8% (29·0 to 255·6) |  | 11267·2 (6462·6 to 19600·5) | 24446·8 (14399·5 to 36817·7) | 117·0% (29·1 to 257·2) |
| Chad | 19603·5 (13428·2 to 27386·3) | 42077·3 (28857·1 to 58739·0) | 114·6% (113·0 to 116·4) |  | 11856·5 (8015·3 to 16563·5) | 32276·1 (21593·6 to 45125·1) | 172·2% (135·1 to 214·3) |  | 130·1 (69·4 to 200·9) | 289·3 (143·1 to 475·4) | 122·4% (50·9 to 234·8) |  | 11382·4 (6087·8 to 17542·3) | 25319·0 (12537·2 to 41486·1) | 122·4% (51·9 to 234·8) |
| Côte d'Ivoire | 12404·5 (8512·6 to 17249·7) | 33357·1 (22860·7 to 46437·8) | 168·9% (166·9 to 171·0) |  | 22366·0 (14942·0 to 31651·4) | 38708·1 (25653·6 to 54160·2) | 73·1% (55·2 to 93·4) |  | 227·3 (139·1 to 336·8) | 298·6 (164·2 to 481·7) | 31·4% (-19·1 to 111·9) |  | 19916·4 (12191·6 to 29445·2) | 26115·6 (14442·2 to 42080·2) | 31·1% (-19·1 to 111·8) |
| Gambia | 23344·7 (16033·3 to 32560·5) | 40107·0 (27421·5 to 55887·3) | 71·8% (70·3 to 73·3) |  | 1823·5 (1194·0 to 2595·3) | 3092·9 (2056·5 to 4351·1) | 69·6% (51·1 to 90·9) |  | 16·9 (10·7 to 25·1) | 21·2 (12·8 to 32·7) | 25·2% (-36·0 to 138·9) |  | 1478·5 (936·7 to 2195·4) | 1851·4 (1117·6 to 2863·0) | 25·2% (-35·9 to 138·1) |
| Ghana | 1942·6 (1334·0 to 2718·0) | 3282·0 (2248·1 to 4585·6) | 68·9% (67·6 to 70·6) |  | 25045·8 (16390·1 to 35575·2) | 37705·5 (24699·3 to 53136·5) | 50·5% (33·8 to 71·2) |  | 251·0 (158·7 to 362·4) | 247·4 (138·5 to 384·6) | -1·4% (-46·1 to 85·6) |  | 21858·7 (13814·1 to 31533·2) | 21635·2 (12097·2 to 33623·4) | -1·0% (-46·0 to 86·0) |
| Guinea | 26437·6 (18044·8 to 36854·7) | 39605·1 (27135·8 to 55219·4) | 49·8% (48·8 to 51·1) |  | 11488·1 (7676·1 to 16145·0) | 20334·7 (13649·7 to 28971·7) | 77·0% (55·8 to 99·7) |  | 165·1 (93·6 to 261·0) | 213·5 (136·0 to 310·7) | 29·3% (-18·8 to 124·5) |  | 14420·7 (8194·5 to 22742·8) | 18641·5 (11916·4 to 27120·9) | 29·3% (-18·8 to 124·4) |
| Guinea-Bissau | 12187·8 (8343·0 to 16963·4) | 21414·7 (14661·7 to 29887·5) | 75·7% (73·8 to 77·4) |  | 1820·6 (1239·7 to 2560·9) | 2745·8 (1827·5 to 3910·7) | 50·8% (31·4 to 72·0) |  | 24·3 (11·6 to 41·5) | 17·7 (10·0 to 28·5) | -27·3% (-59·2 to 34·4) |  | 2125·1 (1016·4 to 3615·9) | 1549·6 (873·9 to 2499·4) | -27·1% (-58·9 to 34·7) |
| Liberia | 1893·0 (1298·6 to 2632·6) | 2867·9 (1967·9 to 4001·8) | 51·5% (49·1 to 53·1) |  | 3297·1 (2245·0 to 4648·8) | 6043·9 (4024·8 to 8561·1) | 83·3% (54·8 to 108·2) |  | 59·2 (25·6 to 99·1) | 29·7 (16·8 to 47·7) | -49·8% (-73·4 to 28·7) |  | 5173·1 (2248·8 to 8659·2) | 2605·7 (1472·3 to 4173·3) | -49·6% (-73·3 to 29·0) |
| Mali | 3345·1 (2296·2 to 4620·0) | 6302·3 (4305·0 to 8804·8) | 88·4% (83·5 to 91·9) |  | 16320·3 (10977·8 to 23013·6) | 38962·1 (25935·9 to 55338·9) | 138·7% (111·0 to 169·5) |  | 181·4 (100·1 to 294·8) | 359·5 (228·3 to 535·6) | 98·2% (29·4 to 230·5) |  | 15858·0 (8789·6 to 25773·0) | 31419·6 (20024·3 to 46813·5) | 98·1% (29·4 to 230·0) |
| Mauritania | 17245·4 (11806·9 to 24037·4) | 40667·2 (27910·3 to 56768·1) | 135·8% (133·6 to 137·7) |  | 3598·2 (2362·0 to 5069·7) | 5064·3 (3412·4 to 7226·0) | 40·7% (23·9 to 58·5) |  | 24·2 (14·0 to 36·2) | 17·1 (9·8 to 27·2) | -29·2% (-65·6 to 42·6) |  | 2116·5 (1230·3 to 3164·3) | 1505·8 (862·2 to 2390·2) | -28·9% (-65·3 to 43·3) |
| Niger | 3787·2 (2604·8 to 5288·5) | 5281·5 (3613·0 to 7359·3) | 39·5% (38·4 to 40·7) |  | 16466·7 (11253·7 to 22799·2) | 47201·9 (31396·2 to 65648·2) | 186·7% (148·4 to 225·8) |  | 213·2 (93·4 to 373·6) | 408·2 (208·6 to 726·7) | 91·5% (8·6 to 242·1) |  | 18650·5 (8215·2 to 32774·9) | 35743·3 (18307·5 to 63581·7) | 91·6% (8·9 to 241·2) |
| Nigeria | 16915·8 (11635·6 to 23441·2) | 48605·2 (33375·1 to 67683·0) | 187·3% (181·8 to 190·6) |  | 164672·5 (112725·9 to 230420·5) | 347632·1 (237765·3 to 485230·1) | 111·1% (103·2 to 118·0) |  | 2006·2 (1035·2 to 3284·6) | 2894·7 (1638·9 to 4515·2) | 44·3% (-1·4 to 113·5) |  | 291657·9 (176937·5 to 483658·9) | 252426·2 (142601·9 to 393638·3) | 44·5% (-1·2 to 113·8) |
| Sao Tome and Principe | 212·9 (148·2 to 295·1) | 237·9 (163·4 to 330·9) | 11·8% (9·1 to 14·6) |  | 219·0 (153·1 to 298·2) | 245·4 (165·8 to 341·2) | 12·0% (-2·6 to 29·8) |  | 5·5 (3·5 to 7·9) | 4·0 (1·6 to 7·1) | -27·6% (-67·1 to 33·6) |  | 482·5 (311·6 to 699·9) | 350·8 (143·7 to 622·7) | -27·3% (-67·0 to 34·0) |
| Senegal | 14603·6 (10049·6 to 20330·7) | 21451·4 (14672·1 to 29885·2) | 46·9% (45·0 to 48·4) |  | 14077·4 (9407·3 to 19717·0) | 20667·4 (13759·0 to 29291·5) | 46·8% (29·4 to 65·6) |  | 168·2 (100·1 to 248·7) | 128·7 (76·8 to 196·6) | -23·5% (-59·8 to 48·2) |  | 14682·5 (8767·7 to 21679·0) | 11295·5 (6735·6 to 17286·0) | -23·1% (-59·6 to 48·5) |
| Sierra Leone | 6540·9 (4501·5 to 9060·1) | 12437·9 (8559·5 to 17257·1) | 90·2% (86·4 to 93·1) |  | 6439·6 (4358·2 to 8998·2) | 12419·1 (8471·6 to 17179·6) | 92·9% (64·3 to 123·0) |  | 114·0 (51·7 to 191·4) | 168·9 (88·4 to 291·6) | 48·2% (-15·2 to 168·1) |  | 9972·4 (4540·2 to 16756·1) | 14814·6 (7743·3 to 25573·3) | 48·6% (-14·9 to 168·9) |
| Togo | 6974·0 (4780·7 to 9730·0) | 11106·1 (7614·0 to 15487·1) | 59·2% (57·7 to 60·3) |  | 6699·8 (4477·9 to 9451·8) | 10685·9 (7061·7 to 15202·2) | 59·5% (40·7 to 80·3) |  | 59·5 (36·3 to 104·6) | 54·3 (32·1 to 93·3) | -8·7% (-46·2 to 52·5) |  | 5197·3 (3184·0 to 9124·5) | 4754·7 (2816·3 to 8150·5) | -8·5% (-45·9 to 52·4) |

Data in parentheses are 95% uncertainty intervals (UIs) unless otherwise stated.

Abbreviations: DALYs = disability-adjusted life-years; GBD = Global Burden of Disease.

**Supplementary Table 4.** Top ten countries and territories with specific cancers in children under 5-year-old in 2019.

|  | **Total neoplasms** | |  | **Liver cancer** | |  | **Leukemia** | |  | **Brain and nervous system cancers** | |  | **Malignant skin melanoma** | |  | **Testicular cancer** | |
| --- | --- | --- | --- | --- | --- | --- | --- | --- | --- | --- | --- | --- | --- | --- | --- | --- | --- |
|  | **Cases** | **DALYs** |  | **Cases** | **DALYs** |  | **Cases** | **DALYs** |  | **Cases** | **DALYs** |  | **Cases** | **DALYs** |  | **Cases** | **DALYs** |
| **Ranking by number of incidence cases**  **in 2019** | | | |  |  |  |  |  |  |  |  |  |  |  |  |  |  |
| 1 | China | China |  | China | China |  | China | China | - | China | India |  | United States of America | United States of America |  | China | India |
| 2 | India | India |  | India | India |  | Ethiopia | Ethiopia | - | India | China |  | China | Nigeria |  | United States of America | Pakistan |
| 3 | Japan | Pakistan |  | Pakistan | Nigeria |  | India | India | - | Pakistan | Pakistan |  | France | India |  | India | China |
| 4 | Russian Federation | Nigeria |  | Democratic Republic of the Congo | Pakistan |  | Pakistan | Pakistan | - | Brazil | Nigeria |  | United Kingdom | China |  | Mexico | Mexico |
| 5 | Nigeria | Ethiopia |  | Nigeria | Democratic Republic of the Congo |  | United Republic of Tanzania | United Republic of Tanzania | - | Nigeria | Brazil |  | Russian Federation | Brazil |  | Turkey | Nigeria |
| 6 | Mexico | United Republic of Tanzania |  | United Republic of Tanzania | Cambodia |  | Indonesia | Nigeria | - | United States of America | Bangladesh |  | Australia | Russian Federation |  | Russian Federation | Turkey |
| 7 | Republic of Korea | Indonesia |  | Ethiopia | United Republic of Tanzania |  | Nigeria | Indonesia | - | Bangladesh | United Republic of Tanzania |  | Brazil | United Republic of Tanzania |  | Germany | United States of America |
| 8 | Egypt | Brazil |  | Egypt | Indonesia |  | Mozambique | Mozambique | - | Turkey | Ethiopia |  | India | Pakistan |  | Pakistan | Indonesia |
| 9 | Uzbekistan | Uganda |  | Cambodia | Ethiopia |  | Philippines | Philippines | - | Ethiopia | Indonesia |  | Italy | Uganda |  | Argentina | Ethiopia |
| 10 | Democratic Republic of the Congo | Mozambique |  | United States of America | Egypt |  | Brazil | Brazil | - | Iran (Islamic Republic of) | United States of America |  | Germany | Mexico |  | France | Brazil |
| Total (proportion of global total number) † | 4472276.2（53.6） | 22215.7 (56.7) |  | 671.7 (53.7) | 84484.1 (55.8) |  | 36481.3 (61.3) | 861698.8 (59.4) |  | 11755.7 (65.4) | 471890.6 (63.1) |  | 616.5 (59.4) | 5814.4 (45.7) |  | 9386.4 (54.1) | 18005.0 (49.6) |
| **Ranking by the incidence rate in 2019**  **(per 100,000 population)** | | | |  |  |  |  |  |  |  |  |  |  |  |  |  |  |
| 1 | Japan | Malawi |  | Cambodia | Cambodia |  | San Marino | Ethiopia |  | San Marino | Haiti |  | Australia | New Zealand |  | Tonga | Tonga |
| 2 | Brunei  Darussalam | United Republic of Tanzania |  | Guinea | Gambia |  | Mozambique | Mozambique |  | Andorra | Pakistan |  | New Zealand | Australia |  | Chile | Kiribati |
| 3 | Singapore | South Sudan |  | Gambia | Guinea |  | Ethiopia | South Sudan |  | Denmark | Bhutan |  | Luxembourg | Luxembourg |  | Monaco | Turkey |
| 4 | Republic of Korea | Haiti |  | Malawi | Malawi |  | United Republic of Tanzania | United Republic of Tanzania |  | Norway | Albania |  | France | Guinea |  | Greece | Chile |
| 5 | Croatia | Mozambique |  | Burkina Faso | Burkina Faso |  | South Sudan | Haiti |  | Sweden | Mozambique |  | Sweden | Ukraine |  | Slovenia | Montenegro |
| 6 | Romania | Sao Tome and Principe |  | United Republic of Tanzania | Myanmar |  | Haiti | Djibouti |  | Norway | United Republic of Tanzania |  | Italy | Bermuda |  | Denmark | Monaco |
| 7 | Serbia | Ethiopia |  | Mozambique | United Republic of Tanzania |  | Djibouti | Somalia |  | Turkey | Northern Mariana Islands |  | United Kingdom | Poland |  | Sweden | Mexico |
| 8 | Slovakia | Djibouti |  | Mali | Mozambique |  | Albania | Zambia |  | Greece | San Marino |  | Austria | France |  | Netherlands | Slovakia |
| 9 | Czechia | Burkina Faso |  | Myanmar | Mali |  | China | Rwanda |  | Finland | Armenia |  | Norway | Sweden |  | Germany | Bolivia (Plurinational State of) |
| 10 | Albania | Rwanda |  | Uganda | Uganda |  | Somalia | Eritrea |  | Albania | Sierra Leone |  | Bermuda | Malawi |  | Poland | Ecuador |

† Percentages indicate proportions of the global total number that are accounted for by the top ten countries.

Abbreviations: Cases = Incidence cases; DALYs= disability-adjusted life-years.

**Supplementary Table 4.** Top ten countries and territories with specific cancers in children under 5-year-old in 2019 (continue).

|  | **Kidney cancer** | |  | **Hodgkin lymphoma** | |  | **Non-Hodgkin lymphoma** | |  | **Other malignant neoplasms** | |  | **Other neoplasms** | |
| --- | --- | --- | --- | --- | --- | --- | --- | --- | --- | --- | --- | --- | --- | --- |
|  | **Cases** | **DALYs** |  | **Cases** | **DALYs** |  | **Cases** | **DALYs** |  | **Cases** | **DALYs** |  | **Cases** | **DALYs** |
| **Ranking by number of incidence cases in 2019** | | | |  |  |  |  |  |  |  |  |  |  |  |
| 1 | China | Nigeria |  | China | Nigeria |  | - | Nigeria | - | China | Pakistan |  | China | China |
| 2 | Nigeria | China |  | Nigeria | Pakistan |  | - | India | - | Pakistan | Nigeria |  | India | India |
| 3 | Brazil | India |  | United Republic of Tanzania | China |  | - | China | - | India | India |  | Japan | Mexico |
| 4 | India | Indonesia |  | Pakistan | India |  | - | United Republic of Tanzania | - | Nigeria | China |  | Russian Federation | Brazil |
| 5 | Indonesia | Brazil |  | India | Ethiopia |  | - | Pakistan | - | United States of America | United Republic of Tanzania |  | Nigeria | United States of America |
| 6 | United States of America | Philippines |  | Mexico | Mexico |  | - | Myanmar | - | Russian Federation | Ethiopia |  | Mexico | Pakistan |
| 7 | Philippines | Pakistan |  | Japan | Indonesia |  | - | Indonesia | - | Indonesia | Uganda |  | Republic of Korea | Colombia |
| 8 | Pakistan | United Republic of Tanzania |  | Germany | Guinea |  | - | Burkina Faso | - | Iran (Islamic Republic of) | Malawi |  | Egypt | Nigeria |
| 9 | Mexico | Burkina Faso |  | Viet Nam | Uganda |  | - | Brazil | - | Bangladesh | Indonesia |  | Uzbekistan | Japan |
| 10 | Turkey | Mali |  | Brazil | Mali |  | - | Uganda | - | Mexico | Bangladesh |  | Democratic Republic of the Congo | Turkey |
| Total (Proportion of global total number) | 4041.3 (51.2) | 80641.6 (51.8) |  | 340.3 (61.0) | 8770.5 (66.7) |  | - | 92303.03 (55.9) |  | 10475.3 (55.0) | 654996.5 (54.6) |  | 4421683.3 (51.1) | 14672.0 (63.0) |
| **Ranking by the incidence rate in 2019**  **(per 100,000 population)** | | | |  |  |  |  |  |  |  |  |  |  |  |
| 1 | Kuwait | Benin |  | San Marino | Nigeria |  | - | Solomon Islands |  | Sao Tome and Principe | Malawi |  | Japan | Dominica |
| 2 | Republic of Moldova | Sierra Leone |  | Georgia | Guinea |  | - | Myanmar |  | Palau | Sao Tome and Principe |  | Brunei Darussalam | Colombia |
| 3 | Turkey | Burkina Faso |  | Panama | Georgia |  | - | Haiti |  | Canada | United Republic of Tanzania |  | Singapore | Bermuda |
| 4 | Niue | Mali |  | Japan | Panama |  | - | United Republic of Tanzania |  | Honduras | Uganda |  | Republic of Korea | Grenada |
| 5 | Benin | Sao Tome and Principe |  | Greece | Turkmenistan |  | - | Burkina Faso |  | Albania | South Sudan |  | Croatia | Saint Vincent and the Grenadines |
| 6 | Sierra Leone | Côte d'Ivoire |  | Germany | Mali |  | - | Dominica |  | San Marino | Djibouti |  | Romania | Mexico |
| 7 | Ukraine | Guinea |  | Ireland | Pakistan |  | - | South Sudan |  | Germany | Mozambique |  | Serbia | Ecuador |
| 8 | Burkina Faso | Haiti |  | Canada | Uzbekistan |  | - | Sierra Leone |  | Oman | Rwanda |  | Slovakia | Northern Mariana Islands |
| 9 | Sao Tome and Principe | Nigeria |  | Australia | Somalia |  | - | Ghana |  | Taiwan (Province of China) | Zambia |  | Czechia | Venezuela (Bolivarian Republic of) |
| 10 | Mali | Malawi |  | Netherlands | South Sudan |  | - | Benin |  | Peru | Eritrea |  | Albania | Dominican Republic |

**Supplementary Table 5.** Incidence cases and DALYs of specific cancers among children under 5-year-old globally, and by SDI regions, GBD super-regions, GBD regions, countries and territories in 2019.

|  | **Liver cancer** | |  | **Total Leukemia** | |  | **Brain and nervous system cancers** | |  | **Malignant skin melanoma** | |  | **Testicular cancer** | |
| --- | --- | --- | --- | --- | --- | --- | --- | --- | --- | --- | --- | --- | --- | --- |
|  | **Cases** | **DALYs** |  | **Cases** | **DALYs** |  | **Cases** | **DALYs** |  | **Cases** | **DALYs** |  | **Cases** | **DALYs** |
| **Global** | 1251.9 (812.8 to 1796.1) | 151482.6 (111939.1 to 196599.2) |  | 59483.4 (48774.0 to 72110.7) | 1451022.0 (1165442.3 to 1788374.2) |  | 18244.1 (13713.2 to 22560.2) | 747940.7 (563194.0 to 929664.2) |  | 1038.7 (394.9 to 2565.7) | 12734.0 (6476.0 to 26102.3) |  | 17343.6 (6925.4 to 30721.5) | 36290.1 (23748.9 to 70719.9) |
| High SDI | 78.1 (54.3 to 105.6) | 4842.6 (3742.9 to 5725.5) |  | 3379.6 (2733.3 to 4096.9) | 36819.2 (32413 to 41631.4) |  | 1779.3 (1233.6 to 2243.3) | 36056.2 (25378.1 to 42052.8) |  | 497.3 (103.4 to 1664.4) | 2268.6 (538.3 to 7612.4) |  | 4834.9 (248.5 to 11354.4) | 3434.4 (693.3 to 7780.8) |
| High-middle SDI | 99.9 (76.1 to 127.3) | 11221.8 (9227.5 to 13767.9) |  | 9172.4 (6978.0 to 11817.8) | 134509.1 (108954.0 to 165502.7) |  | 3001.8 (1950.7 to 3788.6) | 90749.6 (61148.9 to 110517.7) |  | 249.4 (89.0 to 561.5) | 2170.6 (815.7 to 5477.4) |  | 5173.9 (1583.3 to 10019.1) | 5810.7 (3332.0 to 10276.5) |
| Middle SDI | 276.1 (187.7 to 387.5) | 35236.9 (26313.3 to 45573.3) |  | 14847.7 (11622.5 to 18664.2) | 322373.7 (262143.4 to 398308.1) |  | 4507.1 (3145.1 to 5750.8) | 174015.6 (122969.3 to 220817.3) |  | 128.1 (87.0 to 211.9) | 2182.2 (1568.1 to 3541.3) |  | 4031.8 (2258.0 to 6580.5) | 9915.6 (6760.0 to 21727.6) |
| Low-middle SDI | 283.9 (173.1 to 432.2) | 39389.2 (28148.3 to 52356.9) |  | 11413.2 (8103.7 to 15500.2) | 317752.3 (236900.9 to 414697.1) |  | 4531.9 (3313.2 to 5863.6) | 221362.3 (162724.8 to 284983) |  | 71.2 (49.9 to 117.4) | 2146.8 (1466.8 to 3798.2) |  | 1696.6 (1008.4 to 2839.4) | 8062.9 (5568.5 to 13942.9) |
| Low SDI | 464.2 (263.7 to 753.2) | 60704.7 (40500.5 to 86713.5) |  | 18193.1 (11690.5 to 26064.4) | 638185.1 (438415.9 to 886960.0) |  | 3695.2 (2498.9 to 5305.1) | 225246.3 (150942.3 to 328789.2) |  | 79.7 (35.9 to 233.4) | 3957.4 (1627.6 to 12561.2) |  | 1079.0 (506.7 to 2979.0) | 9044.0 (5006.5 to 21385.1) |
| **Central Europe, Eastern Europe, and Central Asia** | 28.8 (17.2 to 46.2) | 3902.8 (2624.7 to 5941.1) |  | 1409.6 (1121.2 to 1735.8) | 34544.3 (28106 to 42072.8) |  | 604.1 (458.5 to 742.2) | 31184.0 (23662.7 to 38514.8) |  | 110.7 (24.0 to 291.6) | 1231.2 (304.7 to 3470.4) |  | 1828.2 (107.5 to 4261.5) | 1785.1 (619.8 to 3737.3) |
| **Central Asia** | 9.1 (4.6 to 16.3) | 1227.6 (768.8 to 2009.3) |  | 547.6 (386.6 to 768.9) | 13965.6 (10210.5 to 18974.7) |  | 198.0 (144.1 to 258.8) | 10512.0 (7753.0 to 13849.5) |  | 16.1 (5.1 to 38.5) | 294.6 (104.1 to 775.3) |  | 273.3 (19.8 to 835.4) | 415.7 (165.3 to 1396.2) |
| Armenia | 0.2 (0.1 to 0.5) | 30.1 (12.5 to 60.2) |  | 20.5 (14.3 to 28.3) | 443.7 (314.6 to 616.6) |  | 8.1 (5.5 to 11.4) | 391.6 (263.3 to 544.9) |  | 0.4 (0.1 to 1.0) | 5.1 (2.1 to 15.0) |  | 22.3 (0.4 to 88.6) | 24.0 (4.7 to 78.5) |
| Azerbaijan | 0.7 (0.3 to 1.5) | 94.8 (48.4 to 200.8) |  | 118.0 (53.0 to 241.8) | 2114.6 (979.8 to 4154.0) |  | 17.3 (9.0 to 29.7) | 931.4 (485.1 to 1599.7) |  | 0.4 (0.2 to 0.7) | 7.7 (4.0 to 15.0) |  | 15.0 (4.3 to 41.7) | 45.2 (23.9 to 78.0) |
| Georgia | 0.1 (0.1 to 0.3) | 21.8 (8.3 to 42.3) |  | 16.1 (10.3 to 24.7) | 391.6 (270.7 to 569.2) |  | 6.6 (3.1 to 9.8) | 347.7 (156.8 to 514.7) |  | 0.7 (0.2 to 2.6) | 12.0 (3.2 to 48.1) |  | 25.7 (0.4 to 103.5) | 32.6 (9.3 to 106.0) |
| Kazakhstan | 1.5 (0.6 to 3.1) | 207.4 (96.8 to 385.9) |  | 93.1 (63.9 to 134.9) | 2646.3 (1836.6 to 3635.6) |  | 39.5 (12.4 to 63.0) | 1963.7 (611.4 to 3112.3) |  | 6.9 (1.2 to 21.1) | 95.8 (19.0 to 350.4) |  | 75.2 (1.0 to 365.6) | 78.4 (16.6 to 335.3) |
| Kyrgyzstan | 0.5 (0.2 to 0.9) | 65.5 (38.1 to 109.8) |  | 20.3 (14.7 to 27.2) | 632.1 (457.6 to 839.9) |  | 6.9 (4.8 to 9.3) | 372.5 (260.1 to 500.7) |  | 1.0 (0.4 to 2.8) | 21.9 (8.6 to 64.6) |  | 16.4 (0.4 to 61.0) | 24.5 (6.1 to 103.7) |
| Mongolia | 0.2 (0.1 to 0.3) | 23.6 (13.2 to 39.8) |  | 6.6 (3.0 to 12.9) | 253.8 (116.4 to 533.0) |  | 4.3 (2.3 to 6.9) | 243.1 (128.3 to 384.6) |  | 0.1 (0.1 to 0.2) | 3.4 (2.4 to 4.6) |  | 5.0 (0.9 to 13.2) | 22.3 (7.5 to 37.9) |
| Tajikistan | 1.1 (0.5 to 2.7) | 128.7 (72.3 to 323.9) |  | 70.7 (33.8 to 157.8) | 1949.5 (953.3 to 3963.0) |  | 20.0 (7.9 to 51.8) | 1146.2 (452.5 to 2961.7) |  | 1.2 (0.4 to 3.0) | 41.6 (13.0 to 103.7) |  | 0.4 (0.2 to 0.8) | 7.3 (5.3 to 10.0) |
| Turkmenistan | 1.3 (0.7 to 2.0) | 176.9 (128.6 to 241.0) |  | 35.6 (24.8 to 50.1) | 937.0 (675.2 to 1278.6) |  | 10.0 (6.4 to 14.7) | 542.6 (348.5 to 780.8) |  | 0.9 (0.2 to 2.3) | 17.8 (5.5 to 54.2) |  | 26.7 (0.8 to 109.5) | 51.6 (10.9 to 290.7) |
| Uzbekistan | 3.6 (1.6 to 6.5) | 478.7 (268.3 to 781.1) |  | 166.8 (116.5 to 244.0) | 4597.0 (3320.2 to 6370.5) |  | 85.4 (56.7 to 118.1) | 4573.2 (3057.5 to 6309.9) |  | 4.5 (1.5 to 11.3) | 89.4 (31.8 to 243.6) |  | 86.6 (2.0 to 369.2) | 129.7 (33.1 to 537.7) |
| **Central Europe** | 2.4 (1.5 to 3.5) | 331.0 (252.0 to 420.0) |  | 256.9 (196.4 to 334.4) | 4684.3 (3780.6 to 5824.0) |  | 143.6 (106.3 to 197.2) | 6029.6 (4465.1 to 8040.2) |  | 34.2 (6.8 to 84.0) | 276.5 (60.8 to 824.7) |  | 747.5 (55.5 to 1918.6) | 662.0 (200.4 to 1413.0) |
| Albania | 0.2 (0.1 to 0.4) | 24.8 (13.4 to 39.1) |  | 33.3 (16.7 to 64.6) | 526.8 (312.6 to 854.4) |  | 8.4 (5.1 to 16.3) | 362.2 (223.0 to 715.9) |  | 0.5 (0.3 to 1.0) | 5.2 (2.5 to 8.6) |  | 16.6 (4.1 to 51.3) | 23.8 (11.2 to 47.4) |
| Bosnia and Herzegovina | 0.1 (0.0 to 0.2) | 11.0 (6.7 to 18.8) |  | 5.2 (2.8 to 8.0) | 126.5 (68.2 to 184.3) |  | 3.2 (1.9 to 5.1) | 146.3 (89.5 to 237.1) |  | 0.3 (0.1 to 0.5) | 2.9 (1.1 to 5.4) |  | 9.1 (1.5 to 27.1) | 16.8 (4.0 to 31.2) |
| Bulgaria | 0.1 (0.1 to 0.2) | 20.3 (15.1 to 27.5) |  | 23.6 (16.4 to 33.1) | 406.2 (303.3 to 548.4) |  | 10.8 (6.4 to 15.7) | 520.3 (313.4 to 735.8) |  | 1.1 (0.2 to 3.4) | 10.5 (2.7 to 37.6) |  | 19.3 (0.2 to 81.7) | 22.2 (6.3 to 67.6) |
| Croatia | 0.1 (0.0 to 0.1) | 9.5 (6.4 to 13.1) |  | 11.9 (6.6 to 19.2) | 140.5 (99.1 to 188.6) |  | 6.9 (4.4 to 10.7) | 208.3 (141.4 to 303.8) |  | 0.8 (0.1 to 3.2) | 7.1 (1.3 to 31.3) |  | 27.5 (0.3 to 104.6) | 20.4 (3.1 to 65.0) |
| Czechia | 0.2 (0.1 to 0.4) | 31.4 (22.5 to 42.3) |  | 23.1 (13.3 to 37.6) | 308.7 (214.7 to 423.5) |  | 12.6 (7.0 to 18.3) | 491.2 (272.8 to 683.5) |  | 4.8 (0.8 to 17.6) | 23.2 (4.0 to 94.0) |  | 80.3 (0.8 to 324.2) | 58.0 (8.9 to 204.1) |
| Hungary | 0.2 (0.1 to 0.3) | 22.9 (15.9 to 31.0) |  | 16.4 (9.7 to 26.4) | 318.1 (203.4 to 453.2) |  | 10.7 (6.6 to 15.3) | 436.4 (256.8 to 602.1) |  | 3.0 (0.4 to 9.2) | 21.1 (3.2 to 75.0) |  | 60.9 (0.6 to 247.9) | 46.4 (6.9 to 155.4) |
| Montenegro | 0.0 (0.0 to 0.0) | 1.9 (1.1 to 3.0) |  | 1.1 (0.5 to 1.9) | 23.2 (13.0 to 38.0) |  | 1.0 (0.6 to 1.9) | 42.7 (23.0 to 77.6) |  | 0.1 (0.0 to 0.2) | 0.9 (0.3 to 1.8) |  | 4.7 (0.5 to 13.9) | 6.7 (1.2 to 13.7) |
| North Macedonia | 0.1 (0.0 to 0.1) | 7.4 (4.9 to 10.9) |  | 5.2 (3.1 to 8.2) | 96.6 (62.9 to 143.7) |  | 3.0 (1.8 to 4.7) | 136.8 (86.5 to 215.8) |  | 0.4 (0.2 to 1.1) | 4.5 (1.8 to 12.0) |  | 10.1 (2.2 to 29.4) | 16.9 (5.3 to 31.3) |
| Poland | 0.7 (0.4 to 1.0) | 98.2 (66.8 to 143.3) |  | 60.9 (35.5 to 101.6) | 1190.7 (859.9 to 1560.9) |  | 43.5 (26.2 to 73.2) | 1833.3 (1161.5 to 2824.3) |  | 14.7 (1.8 to 37.8) | 127.6 (19.0 to 402.1) |  | 290.3 (2.9 to 993.3) | 223.3 (45.5 to 630.0) |
| Romania | 0.5 (0.3 to 0.7) | 68.9 (48.1 to 92.2) |  | 42.5 (29.4 to 61.6) | 1039.3 (774.5 to 1380.0) |  | 23.9 (16.8 to 33.2) | 1069.2 (750.9 to 1457.0) |  | 5.9 (0.9 to 16.2) | 54.4 (9.3 to 181.8) |  | 123.5 (1.8 to 503.8) | 112.6 (22.1 to 354.0) |
| Serbia | 0.1 (0.1 to 0.2) | 13.0 (7.6 to 20.6) |  | 13.0 (6.6 to 21.6) | 211.6 (113.5 to 342.0) |  | 10.2 (5.5 to 17.7) | 398.5 (223.0 to 678.7) |  | 0.8 (0.3 to 2.1) | 8.3 (2.8 to 22.3) |  | 46.3 (6.1 to 124.4) | 55.2 (9.2 to 112.7) |
| Slovakia | 0.1 (0.1 to 0.3) | 17.8 (11.6 to 26.7) |  | 15.5 (8.7 to 26.2) | 253.7 (174.0 to 359.1) |  | 7.6 (4.3 to 11.4) | 327.6 (189.5 to 481.5) |  | 0.9 (0.3 to 2.2) | 6.6 (2.6 to 15.5) |  | 42.6 (6.9 to 108.6) | 48.8 (11.5 to 93.7) |
| Slovenia | 0.0 (0.0 to 0.0) | 3.8 (2.4 to 5.4) |  | 5.2 (2.5 to 8.4) | 42.4 (25.8 to 60.6) |  | 1.8 (0.9 to 2.7) | 56.8 (28.8 to 82.7) |  | 0.9 (0.1 to 3.6) | 4.3 (0.6 to 18.3) |  | 16.3 (0.2 to 53.5) | 10.9 (1.2 to 32.7) |
| **Eastern Europe** | 17.3 (10.7 to 27.1) | 17.3 (10.7 to 27.1) |  | 605.1 (488.2 to 731.8) | 15894.4 (12916.2 to 18925.8) |  | 262.5 (190.9 to 324.1) | 14642.4 (10527.4 to 18185.2) |  | 60.4 (10.8 to 161.3) | 660.1 (122.7 to 2025.9) |  | 807.4 (16.3 to 1967.3) | 707.4 (201.6 to 1519.0) |
| Belarus | 0.9 (0.5 to 1.4) | 114.4 (78.3 to 160.3) |  | 40.2 (24.5 to 66.9) | 875.6 (611.3 to 1252.8) |  | 17.9 (9.6 to 30.5) | 743.6 (402.7 to 1213.4) |  | 3.1 (0.5 to 10.5) | 21.3 (4.1 to 82.0) |  | 44.9 (0.4 to 194.6) | 34.9 (6.5 to 115.1) |
| Estonia | 0.0 (0.0 to 0.1) | 5.3 (3.3 to 8) |  | 4.6 (2.6 to 7.6) | 73.4 (45.2 to 104.4) |  | 1.8 (0.9 to 2.8) | 69.6 (34.3 to 100.9) |  | 0.6 (0.1 to 2.1) | 3.0 (0.5 to 11.2) |  | 4.9 (0.0 to 19.9) | 3.6 (0.6 to 11.6) |
| Latvia | 0.1 (0.0 to 0.1) | 8.1 (5.0 to 12.8) |  | 5.7 (3.2 to 9.3) | 128.7 (74.0 to 185.5) |  | 2.7 (1.5 to 4.0) | 111.3 (60.9 to 158.7) |  | 0.5 (0.1 to 1.6) | 4.4 (0.7 to 13.8) |  | 4.9 (0.0 to 21.7) | 4.4 (1.0 to 14.3) |
| Lithuania | 0.1 (0.0 to 0.1) | 9.2 (5.8 to 14.5) |  | 5.7(3.5 to 8.8) | 158.2 (99.9 to 238.5) |  | 2.5 (1.5 to 4.1) | 127.9 (79.1 to 204.2) |  | 0.7 (0.1 to 2.3) | 5.3 (1.0 to 20.6) |  | 9.7 (0.1 to 42.4) | 8.0 (1.5 to 25.7) |
| Republic of Moldova | 0.1 (0.0 to 0.1) | 12.1 (7.7 to 18.7) |  | 12.2 (8.0 to 18.1) | 268.2 (184.1 to 385.0) |  | 4.7 (3.0 to 7.1) | 241.3 (154.4 to 357.0) |  | 0.5 (0.1 to 1.7) | 7.2 (2.0 to 26.2) |  | 11.7 (0.3 to 47.7) | 14.4 (3.4 to 53.7) |
| Russian Federation | 12.6 (7.6 to 20.0) | 1727.2 (1096.8 to 2770.9) |  | 375.8 (299.3 to 463.9) | 9802.0 (7927.2 to 11968.2) |  | 164.1 (116.0 to 208.1) | 9886.4(6826.2 to 12643.7) |  | 42.9 (7.8 to 121.7) | 440.5 (82.9 to 1462.9) |  | 650.1 (13.1 to 1597.7) | 556.3 (150.2 to 1230.2) |
| Ukraine | 3.5 (2.1 to 5.8) | 467.9 (293.2 to 751.6) |  | 160.9 (117.7 to 215.0) | 4588.2 (3537.3 to 5725.6) |  | 68.7 (50.8 to 90.1) | 3462.2 (2578.3 to 4446.9) |  | 12.2 (1.4 to 30.0) | 178.2 (24.8 to 489.7) |  | 81.0 (0.9 to 301.9) | 85.8 (26.4 to 205.9) |
| **High-income** | 85.2 (59.4 to 114.8) | 5265.4 (4102.3 to 6209.3) |  | 3767.1 (3034.5 to 4600.4) | 42066.2 (37097.4 to 47569.5) |  | 1920.2 (1355.2 to 2436.8) | 40015.1 (28725.9 to 46859.6) |  | 575.1 (117.6 to 1870.8) | 2705.4 (603.9 to 8963.1) |  | 5866.4 (231.4 to 14213.1) | 4300.6 (797.0 to 10181.5) |
| **Australasia** | 1.6 (1.0 to 2.4) | 147.5 (111.5 to 187.5) |  | 87.8 (56.2 to 136.1) | 1092.1 (881.4 to 1359.3) |  | 47.2 (30.0 to 68.8) | 1377.3 (912.2 to 1783.3) |  | 48.4 (5.0 to 185.3) | 211.6 (23.4 to 809.7) |  | 245.9 (4.6 to 737.1) | 168.7 (21.6 to 476.0) |
| Australia | 1.3 (0.7 to 2.1) | 126.0 (93.4 to 163.4) |  | 65.8 (38.4 to 110.9) | 870.8 (686.4 to 1107.6) |  | 41.5 (26.3 to 61.1) | 1114.7 (735.7 to 1468.6) |  | 41.6 (4.3 to 160.2) | 177.2 (19.2 to 679.5) |  | 218.1 (3.7 to 682.8) | 146.6 (16.5 to 432.7) |
| New Zealand | 0.3 (0.2 to 0.4) | 21.5 (16.7 to 27.5) |  | 21.9 (12.5 to 34.7) | 221.3 (170.8 to 288.5) |  | 5.7 (3.5 to 7.8) | 262.6 (165.3 to 347.0) |  | 6.9 (0.7 to 24.5) | 34.4 (3.7 to 134.6) |  | 27.8 (0.5 to 97.2) | 22.1 (4.0 to 61.8) |
| **High-income Asia Pacific** | 13.0 (8.8 to 18.3) | 727.5 (562.1 to 886.4) |  | 640.3 (496.1 to 828.1) | 5740.4 (4817 to 6807.2) |  | 284.5 (184.0 to 381.9) | 4099.3 (2773.6 to 4962.1) |  | 20.8 (12.9 to 38.4) | 97.2 (64.6 to 175.5) |  | 397.1 (29.2 to 956.7) | 286.2 (61.2 to 665.5) |
| Brunei Darussalam | 0.0 (0.0 to 0.0) | 3.1 (2.0 to 4.7) |  | 2.6 (1.8 to 3.7) | 64.2 (44.9 to 91.3) |  | 1.1 (0.8 to 1.6) | 39.1 (26.9 to 56.3) |  | 0.0 (0.0 to 0.0) | 0.3 (0.2 to 0.6) |  | 0.6 (0.2 to 2.0) | 1.2 (0.6 to 2.1) |
| Japan | 9.8 (6.6 to 14.0) | 511.3 (388.0 to 596.2) |  | 404.6 (287.6 to 567.4) | 3481.0 (3038.4 to 3940.1) |  | 177.4 (112.0 to 255.0) | 2457.0 (1664.0 to 2842.7) |  | 16.2 (8.6 to 33.7) | 74.7 (44.6 to 149.2) |  | 315.1 (8.3 to 851.3) | 205.9 (31.0 to 553.7) |
| Republic of Korea | 2.8 (1.4 to 5.0) | 191.1 (118.6 to 284.1) |  | 211.4 (128.0 to 325.3) | 1984.4 (1234.3 to 2853.6) |  | 97.3 (56.3 to 151.4) | 1468.0 (952.7 to 2144.6) |  | 4.0 (3.0 to 5.4) | 19.2 (14.2 to 25.7) |  | 67.3 (13.7 to 149.7) | 69.6 (16.2 to 137.5) |
| Singapore | 0.3 (0.1 to 0.5) | 22.0 (14.6 to 31.3) |  | 21.7 (11.6 to 35.0) | 210.7 (144.0 to 287.3) |  | 8.7 (3.6 to 13.7) | 135.2 (57.4 to 187.9) |  | 0.6 (0.4 to 1.3) | 2.9 (1.8 to 6.7) |  | 14.1 (0.3 to 45.5) | 9.4 (1.2 to 28.8) |
| **High-income North America** | 39.9 (27.1 to 55.5) | 2460.6 (1893.2 to 2929.1) |  | 861.5 (661.4 to 1105.0) | 14872.6 (13483.2 to 16335.0) |  | 713.9 (487.9 to 993.7) | 14691.3 (11366.8 to 17017.5) |  | 237.9 (41.4 to 911.5) | 1086.4 (219.0 to 4020.1) |  | 1860.5 (62.5 to 4754.8) | 1310.9 (201.4 to 3112.7) |
| Canada | 3.4 (1.8 to 5.4) | 215.9 (168.6 to 274.5) |  | 172.2 (114.7 to 244.1) | 1366.3 (1112.0 to 1690.9) |  | 84.5 (50.8 to 128.7) | 1523.9 (963.7 to 1906.1) |  | 21.0 (3.5 to 74.7) | 102.8 (19.1 to 380.6) |  | 256.3 (4.5 to 759.8) | 172.4 (19.7 to 474.2) |
| Greenland | 0.0 (0.0 to 0.0) | 0.3 (0.2 to 0.6) |  | 0.0 (0.0 to 0.1) | 1.7 (0.9 to 2.9) |  | 0.0 (0.0 to 0.1) | 2.3 (1.2 to 4.1) |  | 0.0 (0.0 to 0.0) | 0.1 (0.0 to 0.1) |  | 0.1 (0.0 to 0.2) | 0.2 (0.0 to 0.4) |
| United States of America | 36.5 (24.6 to 51.3) | 2244.4 (1717.2 to 2701.6) |  | 689.2 (518.6 to 906.5) | 13504.4 (12234.1 to 14928.4) |  | 629.3 (427.3 to 890.1) | 13165.0 (10354.1 to 15415.4) |  | 216.9 (37.4 to 826.0) | 983.6 (199.6 to 3652.0) |  | 1604.1 (55.5 to 4069.9) | 1138.2 (176.9 to 2700.3) |
| **Southern Latin America** | 3.8 (2.4 to 5.5) | 399.1 (316.1 to 498.5) |  | 216.6 (162.2 to 282.5) | 6451.4 (5212.0 to 7886.8) |  | 99.2 (70.3 to 136.0) | 4060.7 (3116.8 to 5164.2) |  | 17.8 (4.0 to 55.9) | 210.3 (53.1 to 803.1) |  | 759.0 (14.5 to 2623.2) | 787.7 (176.3 to 2457.7) |
| Argentina | 2.4 (1.4 to 3.8) | 254.7 (181.0 to 357.8) |  | 147.0 (107.7 to 196.4) | 4806.5 (3867.5 to 5879.0) |  | 70.1 (47.4 to 100.9) | 3041.6 (2307.2 to 3982.7) |  | 11.9 (2.5 to 39.9) | 160.8 (39.2 to 643.7) |  | 432.8 (6.2 to 1921.3) | 492.4 (113.0 to 1646.0) |
| Chile | 1.3 (0.7 to 2.2) | 131.1 (73.3 to 201.6) |  | 60.4 (40.4 to 86.3) | 1410.9 (1045.6 to 1872.4) |  | 24.0 (14.9 to 36.2) | 816.3 (512.1 to 1147.0) |  | 4.8 (1.1 to 15.0) | 38.2 (10.5 to 134.1) |  | 297.0 (4.6 to 1257.0) | 265.9 (45.0 to 870.1) |
| Uruguay | 0.1 (0.1 to 0.2) | 13.3 (9.4 to 18.4) |  | 9.2 (6.0 to 13.1) | 233.7 (164.6 to 315.6) |  | 5.1 (3.3 to 7.6) | 202.7 (143.1 to 279.6) |  | 1.1 (0.2 to 3.1) | 11.3 (2.2 to 37.8) |  | 29.1 (0.4 to 122.6) | 29.4 (6.0 to 93.8) |
| **Western Europe** | 26.9 (17.7 to 38.0) | 1530.7 (1162.4 to 1858.2) |  | 1961.0 (1527.0 to 2425.4) | 13909.6 (11664 to 16076.4) |  | 775.4 (499.3 to 1003.1) | 15786.4 (10181.4 to 18567.1) |  | 250.2 (47.1 to 725.6) | 1099.9 (220.4 to 3288.0) |  | 2603.9 (83.8 to 6338.3) | 1747.1 (248.1 to 4305.9) |
| Andorra | 0.0 (0.0 to 0.0) | 0.3 (0.2 to 0.5) |  | 0.3 (0.2 to 0.6) | 2.4 (1.4 to 3.7) |  | 0.2 (0.1 to 0.4) | 3.7 (2.2 to 5.8) |  | 0.0 (0.0 to 0.0) | 0.1 (0.0 to 0.1) |  | 0.3 (0.0 to 0.6) | 0.3 (0.0 to 0.5) |
| Austria | 0.5 (0.3 to 0.8) | 26.9 (19.6 to 35.2) |  | 26.1 (16.7 to 37.8) | 210.6 (165.5 to 261.5) |  | 12.7 (7.4 to 19.3) | 281.8 (172.0 to 365.4) |  | 5.5 (0.9 to 19.7) | 24.2 (4.1 to 88.6) |  | 59.2 (0.8 to 184.2) | 39.8 (4.6 to 116.1) |
| Belgium | 0.6 (0.4 to 1.0) | 39.4 (29.9 to 50.7) |  | 43.3 (27.1 to 64.7) | 363.6 (286.4 to 460.2) |  | 23.3 (14.7 to 36.6) | 448.8 (322.6 to 624.0) |  | 4.9 (1.0 to 18.5) | 22.5 (5.3 to 86.5) |  | 83.1 (1.5 to 263.4) | 56.3 (6.6 to 168.0) |
| Cyprus | 0.0 (0.0 to 0.1) | 3.2 (2.0 to 4.8) |  | 6.2 (4.0 to 9.3) | 46.0 (30.5 to 66.8) |  | 2.1 (1.3 to 3.3) | 43.8 (26.9 to 65.0) |  | 0.3 (0.1 to 0.6) | 1.4 (0.6 to 2.8) |  | 3.7 (0.5 to 8.0) | 3.6 (0.6 to 7.0) |
| Denmark | 0.3 (0.2 to 0.5) | 19.5 (13.6 to 25.6) |  | 27.6 (16.7 to 39.9) | 201.1 (141.8 to 262.8) |  | 22.9 (10.6 to 34.6) | 272.9 (130.1 to 373.8) |  | 3.1 (0.5 to 13.0) | 13.5 (2.4 to 56.0) |  | 49.4 (1.3 to 140.8) | 33.2 (3.4 to 88.9) |
| Finland | 0.3 (0.1 to 0.4) | 13.5 (9.1 to 19.8) |  | 20.2 (11.6 to 30.7) | 148.0 (103.7 to 195.0) |  | 15.3 (8.6 to 22.8) | 196.2 (108.6 to 262.3) |  | 2.1 (0.4 to 7.5) | 9.4 (1.8 to 33.3) |  | 29.2 (0.3 to 99.9) | 19.8 (2.4 to 65.1) |
| France | 4.6 (2.6 to 7.0) | 274.3 (196.4 to 357.7) |  | 366.6 (239.9 to 502.6) | 2696.3 (2129.1 to 3314.5) |  | 163.1 (76.9 to 257) | 3078.1 (1517.8 to 4057.3) |  | 52.5 (7.7 to 154.9) | 234.8 (36.6 to 730.4) |  | 425.0 (7.4 to 1305.3) | 281.4 (31.0 to 810.4) |
| Germany | 4.7 (2.7 to 7.2) | 254.6 (188.0 to 325.8) |  | 342.2 (238.6 to 475.6) | 2139.7 (1709 to 2641.7) |  | 114.2 (66.6 to 165.6) | 2912.7 (1740.4 to 3627.5) |  | 29.7 (7.3 to 113.3) | 124.8 (33.5 to 455.8) |  | 598.6 (12.1 to 1742.9) | 396.9 (45.4 to 1071.5) |
| Greece | 0.4 (0.2 to 0.6) | 26.6 (20.5 to 34.5) |  | 48.0 (28.2 to 74.6) | 390.3 (293.2 to 498.1) |  | 26.5 (16.3 to 41.2) | 551.9 (402.6 to 781.7) |  | 3.7 (0.8 to 10.0) | 17.0 (4.2 to 49.6) |  | 75.8 (1.4 to 243.8) | 52.2 (6.6 to 150.1) |
| Iceland | 0.0 (0.0 to 0.0) | 1.1 (0.7 to 1.8) |  | 1.1 (0.7 to 1.5) | 9.2 (5.5 to 14.1) |  | 1.0 (0.6 to 1.5) | 18.7 (10.5 to 30.4) |  | 0.2 (0.0 to 0.6) | 0.8 (0.1 to 3.0) |  | 2.1 (0.0 to 6.2) | 1.4 (0.1 to 3.8) |
| Ireland | 0.3 (0.2 to 0.4) | 15.6 (11.4 to 20.5) |  | 18.9 (11.5 to 27.7) | 127.7 (89.6 to 176.6) |  | 12.1 (6.4 to 19.5) | 208.3 (119.2 to 290.2) |  | 3.2 (0.5 to 11.7) | 13.9 (2.5 to 51.8) |  | 41.3 (0.8 to 122.8) | 27.7 (2.9 to 81.4) |
| Israel | 0.8 (0.5 to 1.2) | 54.6 (40.9 to 69.8) |  | 36.8 (22.5 to 59.4) | 433.1 (327.6 to 579.8) |  | 30.2 (19.6 to 46.0) | 712.5 (518.6 to 972.4) |  | 10.7 (1.7 to 37.8) | 53.6 (9.7 to 215.8) |  | 87.7 (1.4 to 286.9) | 60.9 (8.1 to 184.0) |
| Italy | 3.4 (2.3 to 4.7) | 178.5 (146.8 to 212.8) |  | 252.4 (180.6 to 331.9) | 1639.2 (1316.6 to 1928.4) |  | 57.6 (37.1 to 84.7) | 1364.2 (934.6 to 1668.1) |  | 31.5 (5.1 to 89.2) | 134.1 (23.1 to 382.0) |  | 276.3 (6.8 to 722.2) | 184.3 (26.0 to 471.0) |
| Luxembourg | 0.0 (0.0 to 0.0) | 2.0 (1.2 to 3.1) |  | 2.3 (1.5 to 3.1) | 19.5 (12.0 to 29.7) |  | 1.3 (0.7 to 2.1) | 26.8 (14.2 to 42.3) |  | 0.6 (0.1 to 1.4) | 2.7 (0.3 to 7.4) |  | 3.6 (0.1 to 11.2) | 2.4 (0.2 to 7.2) |
| Malta | 0.0 (0.0 to 0.0) | 1.3 (0.9 to 2.0) |  | 1.5 (0.8 to 2.3) | 14.4 (9.6 to 20.8) |  | 0.8 (0.5 to 1.3) | 18.2 (11.0 to 27.3) |  | 0.1 (0.0 to 0.4) | 0.6 (0.2 to 2.2) |  | 2.9 (0.1 to 9.2) | 2.1 (0.2 to 6.4) |
| Monaco | 0.0 (0.0 to 0.0) | 0.1 (0.1 to 0.2) |  | 0.3 (0.2 to 0.5) | 2.1 (1.3 to 3.2) |  | 0.1 (0.0 to 0.1) | 1.4 (0.8 to 2.2) |  | 0.0 (0.0 to 0.0) | 0.1 (0.0 to 0.1) |  | 0.3 (0.0 to 0.6) | 0.3 (0.1 to 0.5) |
| Netherlands | 1.3 (0.7 to 2.0) | 70.9 (49.7 to 93.3) |  | 86.5 (59.5 to 119.9) | 574.9 (426.0 to 731.0) |  | 36.5 (19.1 to 58.0) | 613.3 (333.7 to 822.1) |  | 9.1 (1.7 to 31.3) | 39.2 (7.7 to 137.8) |  | 137.6 (2.8 to 397.2) | 92.3 (10.5 to 258.0) |
| Norway | 0.2 (0.1 to 0.3) | 11.9 (8.5 to 14.3) |  | 15.2 (9.6 to 20.6) | 120.4 (90.0 to 144.9) |  | 18.4 (11.8 to 25.3) | 215.2 (140.7 to 261.3) |  | 3.5 (0.5 to 12.2) | 16.1 (2.4 to 58.9) |  | 41.3 (0.8 to 103.0) | 27.4 (3.3 to 69.9) |
| Portugal | 0.4 (0.2 to 0.7) | 30.7 (21.8 to 42.5) |  | 27.7 (16.0 to 43.5) | 255.7 (191.3 to 340.9) |  | 10.7 (6.4 to 16.4) | 255.7 (162.8 to 358.0) |  | 2.8 (0.6 to 7.0) | 14.9 (3.6 to 42.5) |  | 33.9 (0.5 to 123.7) | 23.9 (3.3 to 78.8) |
| San Marino | 0.0 (0.0 to 0.0) | 0.2 (0.1 to 0.3) |  | 0.5 (0.3 to 0.8) | 2.8 (1.8 to 4.0) |  | 0.2 (0.1 to 0.3) | - 1. (2.0 to 4.7) |  | 0.0 (0.0 to 0.0) | 0.1 (0.0 to 0.2) |  | 0.1 (0.0 to 0.2) | 0.1 (0.0 to 0.2) |
| Spain | 2.2 (1.3 to 3.5) | 139.3 (100.3 to 184.2) |  | 237.7 (160.9 to 330.9) | 1558.8 (1218.5 to 1945.5) |  | 65.1 (39.4 to 98.6) | 1231.4 (802.9 to 1542.0) |  | 22.3 (4.4 to 58.0) | 97.5 (19.5 to 262.2) |  | 162.6 (3.5 to 557.7) | 112.5 (14.6 to 335.6) |
| Sweden | 0.5 (0.3 to 0.7) | 35.2 (26.4 to 44.4) |  | 58.6 (38.1 to 79.2) | 416.5 (292.2 to 526.6) |  | 36.7 (24.1 to 51.4) | 467.6 (311.3 to 598.1) |  | 8.3 (1.1 to 28.8) | 38.2 (5.5 to 138.6) |  | 94.3 (1.9 to 252.0) | 61.9 (6.4 to 163.6) |
| Switzerland | 0.5 (0.3 to 0.7) | 25.5 (19.8 to 31.9) |  | 37.4 (24.9 to 52.9) | 253 (188.9 to 327.3) |  | 19.7 (8.3 to 32.6) | 349.3 (156.2 to 484.0) |  | 4.3 (0.8 to 14.8) | 19.4 (3.8 to 65.3) |  | 51.5 (0.7 to 182.0) | 36.8 (4.7 to 110.6) |
| United Kingdom | 6.0 (3.7 to 8.7) | 304.0 (210.2 to 383.3) |  | 302.1 (220.9 to 399.8) | 2272.3 (1879.7 to 2620.3) |  | 104.1 (74.0 to 145.6) | 2496.9 (1982.7 to 3134.6) |  | 51.7 (8.2 to 145.5) | 220.2 (37.6 to 647.8) |  | 341.9 (11.6 to 936.9) | 228.1 (28.3 to 598.3) |
| **Latin America and Caribbean** | 74.1 (48.5 to 107.8) | 7702.4 (5490.3 to 10478.5) |  | 3450.3 (2649.0 to 4386.3) | 105269.8 (81740.2 to 132126.0) |  | 1261.7 (715.3 to 1725.4) | 53677.4 (31348.0 to 72346.0) |  | 82.0 (30.8 to 204.3) | 1273.5 (526.8 to 3261.2) |  | 1796.5 (243.4 to 4300.9) | 4485.7 (1936.8 to 19001.7) |
| **Andean Latin America** | 10.9 (6.1 to 17.2) | 1345.4 (879.8 to 1957.9) |  | 447.1 (243.2 to 733.4) | 13759.6 (7922.5 to 21507.6) |  | 114.8 (60.7 to 174.5) | 5627.9 (3052.5 to 8518.9) |  | 5.9 (3.0 to 11.2) | 136.5 (70.7 to 253.1) |  | 204.9 (91.1 to 432.2) | 922.8 (560.3 to 1403.7) |
| Bolivia (Plurinational State of) | 3.4 (1.7 to 5.5) | 410.1 (229.4 to 601.0) |  | 162.6 (77.8 to 263.2) | 5403.3 (2696.6 to 8827.9) |  | 32.9 (15.7 to 54.7) | 1803.9 (879.5 to 2988.6) |  | 2.1 (0.8 to 4.3) | 66.5 (24.4 to 135.0) |  | 36.1 (11.8 to 80.2) | 235.6 (121.5 to 356.3) |
| Ecuador | 2.2 (1.1 to 3.7) | 280.5 (155.9 to 432.8) |  | 91.0 (50.7 to 144.9) | 3065.0 (1729.6 to 4750.2) |  | 25.1 (8.7 to 41.9) | 1197.3 (424.1 to 2015.4) |  | 1.5 (0.7 to 3.6) | 29.4 (14.1 to 68.5) |  | 50.5 (17.8 to 127.0) | 262.6 (132.2 to 445.3) |
| Peru | 5.3 (2.6 to 9.3) | 654.8 (339.0 to 1092.7) |  | 193.5 (80.3 to 383.4) | 5291.2 (2321.3 to 10019.7) |  | 56.9 (26.9 to 96.0) | 2626.7 (1266.4 to 4449.1) |  | 2.3 (1.0 to 5.0) | 40.6 (17.5 to 86.4) |  | 118.3 (33.6 to 329.4) | 424.6 (188.7 to 775.1) |
| **Caribbean** | 5.7 (2.9 to 9.8) | 618.7 (380.0 to 968.4) |  | 491.1 (212.4 to 920.3) | 14890.7 (6870.9 to 28115.2) |  | 98.2 (41.8 to 209.3) | 5361.1 (2147.9 to 11873.2) |  | 4.1 (1.9 to 7.7) | 106.8 (46.2 to 235.6) |  | 50.6 (9.9 to 119.4) | 179.7 (97.4 to 377.0) |
| Antigua and Barbuda | 0.0 (0.0 to 0.0) | 0.3 (0.2 to 0.5) |  | 0.4 (0.2 to 0.6) | 9.4 (5.5 to 15.2) |  | 0.1 (0.0 to 0.2) | 5.4 (1.9 to 10.2) |  | 0.0 (0.0 to 0.0) | 0.2 (0.0 to 0.6) |  | 0.1 (0.0 to 0.6) | 0.2 (0.0 to 0.6) |
| Bahamas | 0.0 (0.0 to 0.0) | 1.4 (0.9 to 1.9) |  | 0.6 (0.4 to 0.9) | 21.7 (14.6 to 31.7) |  | 0.3 (0.1 to 0.4) | 14.0 (6.2 to 22.2) |  | 0.0 (0.0 to0.2) | 0.9 (0.2 to 3.6) |  | 0.1 (0.0 to 0.4) | 0.2 (0.1 to 0.4) |
| Barbados | 0.0 (0.0 to 0.0) | 3.3 (2.2 to 4.8) |  | 0.6 (0.4 to 0.9) | 16.4 (10.8 to 24.4) |  | 0.2 (0.1 to 0.3) | 8.9 (3.0 to 14.9) |  | 0.0 (0.0 to 0.1) | 0.3 (0.1 to 0.9) |  | 0.2 (0.0 to 0.8) | 21.3 (14.4 to 30.9) |
| Belize | 0.0 (0.0 to 0.0) | 2.4 (1.7 to 3.1) |  | 1.7 (1.2 to 2.4) | 44.0 (31.9 to 58.5) |  | 0.5 (0.2 to 0.8) | 27.6 (11.3 to 43.5) |  | 0.0 (0.0 to 0.1) | 0.8 (0.3 to 2.3) |  | 0.6 (0.0 to 2.0) | 1.1 (0.3 to 4.6) |
| Bermuda | 0.0 (0.0 to 0.0) | 0.2 (0.1 to 0.3) |  | 0.2 (0.1 to 0.3) | 3.3 (2.3 to 4.5) |  | 0.1 (0.0 to 0.2) | 2.8 (1.1 to 4.4) |  | 0.0 (0.0 to 0.1) | 0.2 (0.0 to 0.6) |  | 0.1 (0.0 to 0.2) | 0.0 (0.0 to 0.1) |
| Cuba | 0.3 (0.2 to 0.4) | 28.7 (20.9 to 37.0) |  | 22.4 (15.4 to 31.3) | 649.5 (471.6 to 866.3) |  | 13.7 (4.0 to 21.8) | 523.5 (147.8 to 784.4) |  | 1.2 (0.4 to 3.0) | 11.7 (4.8 to 32.0) |  | 16.4 (0.4 to 67.0) | 17.6 (4.2 to 50.7) |
| Dominica | 0.0 (0.0 to 0.0) | 0.3 (0.2 to 0.5) |  | 0.4 (0.2 to 0.6) | 12.7 (8.1 to 19.4) |  | 0.1 (0.0 to 0.1) | 2.8 (1.1 to 4.9) |  | 0.0 (0.0 to 0.0) | 0.1 (0.0 to 0.1) |  | 0.0 (0.0 to 0.1) | 0.2 (0.1 to 0.3) |
| Dominican Republic | 2.1 (0.9 to 4.1) | 237.3 (126.6 to 416.1) |  | 73.4 (36.0 to 135.0) | 1963.0 (1028.5 to 3318.7) |  | 9.3 (4.2 to 16.8) | 480.2 (220.6 to 861.1) |  | 0.5 (0.3 to 0.7) | 11.1 (6.2 to 17.6) |  | 5.9 (1.7 to 17.5) | 29.1 (13.8 to 52.3) |
| Grenada | 0.0 (0.0 to 0.0) | 0.5 (0.3 to 0.8) |  | 0.4 (0.2 to 0.5) | 8.6 (5.6 to 12.9) |  | 0.1 (0.0 to 0.2) | 5.3 (2.4 to 8.9) |  | 0.0 (0.0 to 0.0) | 0.2 (0.1 to 0.6) |  | 0.0 (0.0 to 0.2) | 0.1 (0.0 to 0.5) |
| Guyana | 0.0 (0.0 to 0.1) | 5.3 (3.2 to 8.0) |  | 2.3 (1.5 to 3.7) | 88.3 (56.4 to 137.6) |  | 0.6 (0.3 to 1.3) | 36.3 (15.5 to 71.6) |  | 0.1 (0.0 to 0.1) | 1.6 (0.6 to 4.4) |  | 0.9 (0.0 to 3.1) | 2.4 (0.7 to 12.8) |
| Haiti | 2.7 (1.1 to 6.2) | 287.1 (134.9 to 607.3) |  | 351.5 (83.7 to 774.6) | 10966.8 (3038.9 to 23967.6) |  | 62.4 (14.8 to 167.9) | 3723.3 (882.5 to 10098.2) |  | 1.4 (0.4 to 4.0) | 66.9 (18.3 to 191.0) |  | 11.2 (3.3 to 36.0) | 100.6 (45.4 to 226.7) |
| Jamaica | 0.1 (0.1 to 0.2) | 14.1 (8.9 to 21.8) |  | 6.2 (4.0 to 9.1) | 226.2 (151.6 to 320.4) |  | 2.0 (0.8 to 3.3) | 102.8 (43.8 to 168.0) |  | 0.2 (0.1 to 0.5) | 3.9 (1.8 to 10.0) |  | 2.0 (0.1 to 7.4) | 4.0 (0.9 to 23.6) |
| Puerto Rico | 0.1 (0.0 to 0.1) | 6.3 (4.6 to 8.3) |  | 6.0 (3.9 to 8.8) | 103.1 (75.6 to 138.2) |  | 2.2 (0.8 to 3.3) | 72.9 (28.9 to 105.0) |  | 0.3 (0.1 to 0.8) | 2.5 (1.1 to 7.3) |  | 6.3 (0.1 to 27.5) | 6.1 (1.3 to 18.9) |
| Saint Kitts and Nevis | 0.0 (0.0 to 0.0) | 0.2 (0.1 to 0.3) |  | 0.2 (0.1 to 0.3) | 4.2 (1.5 to 6.6) |  | 0.0 (0.0 to 0.1) | 2.0 (0.7 to 3.6) |  | 0.0 (0.0 to 0.0) | 0.1 (0.0 to 0.1) |  | 0.1 (0.0 to 0.5) | 0.2 (0.0 to 0.8) |
| Saint Lucia | 0.0 (0.0 to 0.0) | 0.6 (0.4 to 0.9) |  | 0.5 (0.3 to 0.7) | 12.9 (8.2 to 19.3) |  | 0.1 (0.1 to 0.2) | 6.1 (2.4 to 10.9) |  | 0.0 (0.0 to 0.0) | 0.3 (0.1 to 0.9) |  | 0.3 (0.0 to 1.3) | 0.5 (0.1 to 2.1) |
| Saint Vincent and the Grenadines | 0.0 (0.0 to 0.0) | 0.5 (0.3 to 0.7) |  | 0.4 (0.2 to 0.6) | 10.7 (6.9 to 16.0) |  | 0.1 (0.0 to 0.2) | 4.8 (2.2 to 7.9) |  | 0.0 (0.0 to 0.0) | 0.3 (0.1 to 0.7) |  | 0.1 (0.0 to 0.3) | 0.2 (0.0 to 0.8) |
| Suriname | 0.0 (0.0 to 0.0) | 3.2 (2.0 to 5.0) |  | 2.5 (1.6 to 4.1) | 68.2 (43.8 to 103.9) |  | 1.4 (0.4 to 2.6) | 75.6 (24.3 to 136.4) |  | 0.0 (0.0 to 0.0) | 0.5 (0.3 to 0.8) |  | 0.7 (0.3 to 1.5) | 4.0 (2.3 to 6.7) |
| Trinidad and Tobago | 0.0 (0.0 to 0.1) | 5.6 (3.8 to 8.0) |  | 4.6 (3.0 to 6.8) | 172.5 (114.9 to 246.4) |  | 1.6 (0.4 to 2.7) | 81.3 (20.3 to 139.8) |  | 0.1 (0.0 to 0.2) | 1.5 (0.7 to 3.5) |  | 3.7 (0.0 to 14.9) | 6.8 (2.1 to 25.5) |
| United States Virgin Islands | 0.0 (0.0 to 0.0) | 0.2 (0.1 to 0.4) |  | 0.2 (0.1 to 0.3) | 4.7 (2.4 to 8.7) |  | 0.1 (0.0 to 0.1) | 3.8 (1.9 to 6.6) |  | 0.0 (0.0 to 0.0) | 0.2 (0.1 to 0.5) |  | 0.0 (0.0 to 0.1) | 0.1 (0.0 to 0.1) |
| **Central Latin America** | 37.1 (21.4 to 57.7) | 3814.2 (2427.5 to 5604.7) |  | 1476.0 (1079.3 to 1985.9) | 47231.5 (35516.2 to 62477.6) |  | 375.7 (189.5 to 545.6) | 16058.9 (8026.5 to 23037.0) |  | 37.1 (13.4 to 99.9) | 536.4 (210.1 to 1496.7) |  | 1112.7 (66.0 to 2968.2) | 2582.5 (789.9 to 14081.7) |
| Colombia | 6.8 (3.8 to 11.0) | 748.5 (483.0 to 1110.0) |  | 284.0 (189.5 to 418.7) | 8732.8 (5934.7 to 12299.2) |  | 80.8 (41.0 to 127.1) | 3383.1 (1755.1 to 5091.7) |  | 12.5 (3.1 to 40.2) | 148.4 (41.5 to 534.2) |  | 162.9 (3.5 to 682.1) | 281.3 (73.9 to 1302.8) |
| Costa Rica | 0.3 (0.2 to 0.5) | 34.4 (22.5 to 50.9) |  | 12.1 (8.2 to 18.0) | 381.3 (269.8 to 525.3) |  | 6.0 (2.0 to 9.4) | 230.2 (77.8 to 346.1) |  | 1.0 (0.3 to 3.7) | 9.9 (3.1 to 40.6) |  | 12.3 (0.3 to 49.5) | 20.8 (4.8 to 101.7) |
| El Salvador | 0.2 (0.1 to 0.4) | 25.4 (11.1 to 42.8) |  | 29.4 (11.6 to 62.5) | 621.9 (242.9 to 1238.5) |  | 7.3 (2.5 to 12.7) | 341.7 (115.4 to 586.3) |  | 0.3 (0.1 to 0.5) | 4.8 (1.9 to 7.8) |  | 10.1 (1.9 to 30.0) | 47.5 (11.2 to 97.2) |
| Guatemala | 3.0 (1.7 to 4.4) | 328.0 (224.5 to 465.7) |  | 121.5 (82.4 to 172.5) | 3435.5 (2360.5 to 4862.0) |  | 20.2 (11.4 to 30.7) | 1085.3 (603.1 to 1641.9) |  | 1.8 (0.6 to 5.4) | 49.7 (17.6 to 158.5) |  | 52.8 (1.4 to 188.2) | 168.3 (42.6 to 921.1) |
| Honduras | 2.0 (0.8 to 4.3) | 218.0 (102.6 to 439.4) |  | 106.8 (44.4 to 225.2) | 3522.0 (1460.7 to 7098.2) |  | 28.0 (7.8 to 60.4) | 1519.8 (419.0 to 3270.2) |  | 0.3 (0.2 to 0.5) | 9.2 (4.4 to 14.9) |  | 6.0 (1.8 to 17.2) | 52.1 (21.3 to 98.1) |
| Mexico | 22.1 (11.7 to 36.0) | 2140.8 (1201.2 to 3304.2) |  | 761.1 (545.4 to 1023.3) | 24342.9 (18704 to 30758.3) |  | 187.4 (99.4 to 275.1) | 7388.9 (3809.2 to 10300.2) |  | 17.2 (6.3 to 47.2) | 252.3 (99.8 to 698.7) |  | 781.6 (28.4 to 2067.8) | 1832.5 (436.4 to 11185.7) |
| Nicaragua | 0.6 (0.3 to 0.9) | 61.1 (40.8 to 86.0) |  | 37.1 (23.2 to 56.0) | 1349.5 (867.3 to 2016.4) |  | 12.4 (5.6 to 20.8) | 563.0 (263.6 to 917.6) |  | 0.5 (0.3 to 0.7) | 7.9 (5.4 to 12.2) |  | 13.5 (4.6 to 33.8) | 65.0 (41.7 to 99.5) |
| Panama | 0.4 (0.2 to 0.6) | 41.2 (28.5 to 57.2) |  | 18.3 (12.5 to 26.2) | 677.3 (478.0 to 938.6) |  | 7.6 (4.6 to 11.1) | 325.4 (201.0 to 468.8) |  | 0.6 (0.2 to 1.8) | 7.4 (3.4 to 24.1) |  | 8.9 (0.3 to 33.7) | 18.8 (4.2 to 106.8) |
| Venezuela (Bolivarian Republic of) | 1.8 (1.0 to 3.0) | 216.7 (138.9 to 320.3) |  | 105.8 (71.7 to 152.6) | 4168.3 (2831.1 to 5909.6) |  | 26.1 (9.5 to 43.3) | 1221.7 (444.0 to 1949.6) |  | 2.9 (1.1 to 8.6) | 46.7 (19.1 to 148.7) |  | 64.5 (0.9 to 227.3) | 96.1 (26.8 to 349.4) |
| **Tropical Latin America** | 20.5 (14.6 to 27.4) | 1924.1 (1396.0 to 2519.4) |  | 1036.1 (801.0 to 1300.4) | 29388.1 (23015.9 to 36632.3) |  | 673.0 (362.7 to 910.1) | 26629.5 (14473.1 to 35684.6) |  | 34.9 (9.3 to 94.2) | 493.8 (139.6 to 1506.6) |  | 428.3 (25.3 to 1155.7) | 800.7 (251.1 to 3685.5) |
| Brazil | 20.1 (14.1 to 26.7) | 1879.0 (1361.3 to 2461.8) |  | 1017.2 (788.6 to 1274.1) | 28835.3 (22708.3 to 35733.6) |  | 667.2 (360.1 to 903.9) | 26340.2 (14375.9 to 35269.7) |  | 34.6 (9.0 to 93.9) | 486.8 (134.6 to 1498.7) |  | 414.3 (18.2 to 1138.5) | 741.8 (206.9 to 3650.6) |
| Paraguay | 0.4 (0.2 to 0.7) | 45.1 (23.0 to 77.7) |  | 18.8 (9.4 to 37.4) | 552.8 (269.4 to 1177.0) |  | 5.7 (1.8 to 10.6) | 289.3 (95.6 to 520.1) |  | 0.3 (0.2 to 0.6) | 7.0 (3.6 to 12.9) |  | 14.0 (3.4 to 40.1) | 58.8 (19.7 to 116.3) |
| **North Africa and Middle East** | 83.4 (39.6 to 161.8) | 8943.9 (5556.8 to 15764.5) |  | 4231.9 (2861.1 to 5788.5) | 101006.7 (65086.4 to 140446.7) |  | 1737.4 (1123.4 to 2322.2) | 57666.2 (37139.4 to 77963.3) |  | 32.8 (24.1 to 45.3) | 541.5 (398.7 to 809.6) |  | 1510.8 (829.3 to 2781.5) | 4206.2 (3057.0 to 5785.0) |
| **North Africa and Middle East** | 83.4 (39.6 to 161.8) | 8943.9 (5556.8 to 15764.5) |  | 4231.9 (2861.1 to 5788.5) | 101006.7 (65086.4 to 140446.7) |  | 1737.4 (1123.4 to 2322.2) | 57666.2 (37139.4 to 77963.3) |  | 32.8 (24.1 to 45.3) | 541.5 (398.7 to 809.6) |  | 1510.8 (829.3 to 2781.5) | 4206.2 (3057.0 to 5785.0) |
| Afghanistan | 3.4 (1.5 to 6.5) | 383.4 (196.6 to 664.5) |  | 855.1 (380.0 to 1655.5) | 25120.9 (11449.2 to 47338.5) |  | 189.9 (60.4 to 372.6) | 10534.6 (3359.6 to 20635.2) |  | 1.5 (0.8 to 3.0) | 68.7 (38.7 to 144.3) |  | 16.5 (5.2 to 37.3) | 269.9 (133.0 to 480.4) |
| Algeria | 4.4 (1.9 to 8.2) | 414.8 (237.7 to 681.8) |  | 114.6 (67.8 to 186.0) | 3118.7 (1955.2 to 5107.6) |  | 79.5 (36.5 to 138.1) | 2209.0 (1041.6 to 3741.1) |  | 2.6 (1.9 to 3.6) | 37.0 (27.5 to 48.8) |  | 79.0 (23.9 to 206.8) | 232.1 (140.8 to 346.6) |
| Bahrain | 0.0 (0.0 to 0.1) | 3.0 (2.0 to 4.7) |  | 1.7 (1.1 to 2.7) | 47.2 (31.1 to 70.2) |  | 1.4 (0.8 to 2.2) | 29.8 (18.7 to 43.2) |  | 0.1 (0.0 to 0.1) | 0.6 (0.5 to 0.8) |  | 0.9 (0.2 to 2.2) | 2.0 (0.9 to 3.2) |
| Egypt | 46.7 (15.7 to 117.5) | 5214.6 (2296.0 to 11637.9) |  | 381.7 (156.4 to 786.6) | 9879.8 (4385.9 to 20728.6) |  | 185.4 (79.0 to 322.2) | 6819.4 (2833.9 to 11537.3) |  | 3.1 (1.8 to 4.8) | 66.1 (39.3 to 98.8) |  | 132.4 (32.9 to 421.0) | 600.1 (299.7 to 1042.6) |
| Iran (Islamic Republic of) | 5.5 (3.2 to 8.4) | 527.0 (333.3 to 773.7) |  | 365.9 (164.4 to 604.1) | 8135.1 (4104.8 to 12909.8) |  | 280.3 (119.0 to 448.9) | 6260.2 (2860.0 to 9710.2) |  | 6.2 (4.1 to 9.0) | 70.7 (46.7 to 108.3) |  | 223.7 (62.3 to 434.1) | 496.9 (200.6 to 804.5) |
| Iraq | 3.8 (1.8 to 6.9) | 409.6 (242.0 to 658.6) |  | 389.3 (222.7 to 619.2) | 6427.2 (3903.4 to 9919.3) |  | 172.6 (94.3 to 298.1) | 5605.1 (3192.7 to 9567.4) |  | 2.0 (1.2 to 3.2) | 35.6 (22.7 to 53.6) |  | 72.5 (16.9 to 233.5) | 253.8 (121.5 to 449.8) |
| Jordan | 0.5 (0.3 to 0.9) | 54.6 (36.1 to 81.0) |  | 107.9 (69.0 to 170.2) | 1215.0 (795.5 to 1859.0) |  | 28.3 (17.3 to 44.2) | 688.5 (447.0 to 997.1) |  | 0.8 (0.5 to 1.2) | 9.5 (6.8 to 13.1) |  | 27.5 (6.7 to 73.0) | 68.7 (40.7 to 110.1) |
| Kuwait | 0.2 (0.1 to 0.3) | 16.9 (12.4 to 22.6) |  | 20.9 (14.6 to 29.7) | 267.8 (197.6 to 351.2) |  | 12.8 (7.8 to 19.4) | 198.1 (136.4 to 273.2) |  | 0.5 (0.3 to 1.0) | 3.2 (2.3 to 6.6) |  | 14.1 (0.6 to 44.3) | 10.6 (1.6 to 30.4) |
| Lebanon | 0.5 (0.2 to 1.1) | 40.0 (19.4 to 76.7) |  | 32.5 (15.9 to 55.5) | 536.7 (271.3 to 878.4) |  | 19.2 (7.9 to 36.4) | 309.1 (138.8 to 547.8) |  | 0.6 (0.2 to 1.3) | 4.4 (1.7 to 10.4) |  | 23.2 (5.4 to 54.2) | 35.3 (14.6 to 67.0) |
| Libya | 0.5 (0.2 to 1.0) | 51.7 (27.1 to 93.1) |  | 25.7 (13.5 to 44.7) | 587.1 (343.1 to 964.7) |  | 13.4 (7.2 to 23.1) | 413.4 (233.6 to 721.4) |  | 0.2 (0.1 to 0.3) | 3.2 (2.3 to 4.5) |  | 5.0 (1.2 to 14.7) | 16.7 (9.4 to 29.7) |
| Morocco | 1.1 (0.4 to 2.3) | 120.4 (57.2 to 226.2) |  | 47.8 (21.9 to 90.4) | 1347.2 (683.3 to 2425.6) |  | 30.9 (13.9 to 62.9) | 1189.7 (562.4 to 2309.2) |  | 1.1 (0.5 to 1.9) | 24.2 (10.9 to 41.3) |  | 24.9 (5.8 to 72.0) | 126.6 (53.7 to 248.9) |
| Oman | 0.6 (0.3 to 0.9) | 52.8 (36.2 to 71.6) |  | 16.0 (8.7 to 26.5) | 325.6 (180.0 to 501.9) |  | 9.7 (4.2 to 16.2) | 197.1 (90.7 to 308.9) |  | 0.4 (0.3 to 0.5) | 3.8 (3.0 to 4.7) |  | 9.5 (2.2 to 21.3) | 19.1 (9.2 to 30.5) |
| Palestine | 0.4 (0.2 to 0.7) | 40.1 (26.1 to 61.2) |  | 42.6 (25.2 to 71.1) | 679.6 (426.1 to 1022.1) |  | 27.3 (15.8 to 44.2) | 844.5 (518.8 to 1329.2) |  | 0.3 (0.2 to 0.5) | 5.5 (3.9 to 7.5) |  | 2.6 (0.7 to 7.1) | 12.0 (8.0 to 18.1) |
| Qatar | 0.1 (0.1 to 0.3) | 14.6 (8.5 to 24.5) |  | 4.7 (2.5 to 7.9) | 90.2 (53.8 to 145.9) |  | 4.3 (2.2 to 7.8) | 70.0 (39.5 to 113.2) |  | 0.2 (0.1 to 0.2) | 1.2 (0.9 to 1.7) |  | 5.0 (0.8 to 11.6) | 8.7 (3.5 to 14.8) |
| Saudi Arabia | 1.1 (0.4 to 2.2) | 97.7 (51.2 to 162.9) |  | 34.8 (14.8 to 67.0) | 1024.5 (458.6 to 1871.6) |  | 33.9 (16.4 to 69.8) | 804.8 (411.8 to 1601.7) |  | 1.7 (1.0 to 2.5) | 19.5 (11.8 to 30.1) |  | 107.6 (12.8 to 296.0) | 126.1 (42.0 to 260.6) |
| Sudan | 5.2 (2.2 to 10.8) | 567.9 (286.4 to 1101.1) |  | 762.7 (323.1 to 1456.9) | 19026.0 (8616.8 to 34914.4) |  | 197.8 (66.8 to 366.2) | 8845.1 (3064.2 to 16320.6) |  | 1.9 (1.0 to 3.9) | 56.8 (30.7 to 118.8) |  | 40.5 (8.7 to 131.2) | 277.2 (117.4 to 531.9) |
| Syrian Arab Republic | 0.9 (0.5 to 1.5) | 94.6 (61.6 to 138.3) |  | 141.0 (76.8 to 287.9) | 2653.3 (1553.7 to 5275.5) |  | 29.2 (15.4to 56.6) | 875.0 (488.2 to 1641.1) |  | 0.8 (0.5 to 1.1) | 12.0 (8.5 to 16.9) |  | 23.1 (5.1 to 68.6) | 69.3 (40.7 to 110.6) |
| Tunisia | 0.6 (0.2 to 1.1) | 47.6 (23.6 to 90.7) |  | 24.0 (13.6 to 40.6) | 605.1 (354.6 to 974.7) |  | 12.5 (6.1 to 23.2) | 277.5 (146.3 to 474.5) |  | 0.7 (0.4 to 1.1) | 7.6 (4.9 to 11.4) |  | 22.9 (6.4 to 58.1) | 50.5 (31.0 to 82.4) |
| Turkey | 4.3 (2.3 to 6.9) | 434.7 (288.6 to 631.0) |  | 432.8 (268.9 to 635.2) | 9284.3 (6264.1 to 13179.4) |  | 304.4 (140.4 to 503.0) | 6648.9 (3263.3 to 10195.4) |  | 7.2 (3.9 to 13.6) | 77.2 (41.9 to 142.0) |  | 659.1 (160.6 to 1855.5) | 1379.7 (765.5 to 2667.4) |
| United Arab Emirates | 0.1 (0.0 to 0.2) | 15.4 (5.7 to 27.9) |  | 3.5 (1.6 to 7.2) | 115.3 (55.1 to 220.6) |  | 8.4 (2.6 to 17.2) | 280.9 (87.4 to 543.4) |  | 0.1 (0.1 to 0.2) | 2.6 (1.7 to 4.0) |  | 3.8 (0.8 to 11.4) | 15.2 (5.4 to 29.0) |
| Yemen | 3.4 (1.6 to 6.2) | 333.2 (178.4 to 565.5) |  | 422.4 (191.2 to 763.1) | 10417.4 (4721.2 to 18249.6) |  | 94.4 (32.9 to 167.7) | 4506.7 (1591.9 to 7954.6) |  | 0.9 (0.5 to 1.7) | 31.3 (18.1 to 59.7) |  | 15.4 (3.7 to 45.1) | 131.6 (66.3 to 245.4) |
| **South Asia** | 185.6 (126.1 to 263.3) | 26123.4 (19937.7 to 33939.8) |  | 8027.1 (4674.7 to 11814.4) | 230564.4 (147862.6 to 326349.7) |  | 5326.1 (3713.9 to 7258.5) | 246919.9 (174764.4 to 324813.3) |  | 49.9 (34.8 to 78.9) | 1417.9 (1024.0 to 2279.7) |  | 1677.0 (1035.3 to 3091.2) | 7708.7 (5623.5 to 10463.6) |
| **South Asia** | 185.6 (126.1 to 263.3) | 26123.4 (19937.7 to 33939.8) |  | 8027.1 (4674.7 to 11814.4) | 230564.4 (147862.6 to 326349.7) |  | 5326.1 (3713.9 to 7258.5) | 246919.9 (174764.4 to 324813.3) |  | 49.9 (34.8 to 78.9) | 1417.9 (1024.0 to 2279.7) |  | 1677.0 (1035.3 to 3091.2) | 7708.7 (5623.5 to 10463.6) |
| Bangladesh | 21.1 (8.9 to 42.4) | 3116.5 (1752.4 to 5407.1) |  | 427.3 (277.8 to 621.0) | 15507.6 (9963.5 to 22614.3) |  | 368.2 (194.7 to 618.0) | 20420.8 (10946.4 to 33894.7) |  | 4.6 (3.1 to 6.6) | 140.6 (96.9 to 201.8) |  | 115.9 (44.1 to 263.3) | 556.4 (324.3 to 893.0) |
| Bhutan | 0.1 (0.0 to 0.2) | 15.5 (8.2 to 26.7) |  | 4.0 (1.8 to 7.2) | 139.6 (67.3 to 239.0) |  | 2.8 (1.4 to 4.9) | 150.0 (74.2 to 262.1) |  | 0.0 (0.0 to 0.1) | 1.0 (0.6 to 1.5) |  | 1.0 (0.3 to 2.6) | 4.0 (2.2 to 6.6) |
| India | 96.6 (62.7 to 140.8) | 14198.6 (10500.0 to 19058.2) |  | 4134.7 (2449.2 to 6353.9) | 128060.8 (84334.4 to 181876.8) |  | 3194.3 (2102.0 to 4554.9) | 152525.1 (106999.6 to 209989.5) |  | 31.8 (22.8 to 44.8) | 877.4 (660.9 to 1170.1) |  | 973.8 (640.1 to 1485.5) | 4268.3 (3194.2 to 5735.2) |
| Nepal | 6.8 (2.1 to 18.0) | 1013.1 (445.7 to 2343.2) |  | 67.5 (39.6 to 104.0) | 2500.6 (1524.6 to 3749.6) |  | 59.1 (29.1 to 100.7) | 3375.2 (1710.6 to 5701.6) |  | 0.6 (0.4 to 0.9) | 21.2 (14.2 to 30.0) |  | 15.6 (5.6 to 36.5) | 85.2 (46.3 to 137.4) |
| Pakistan | 61.0 (36.2 to 98.6) | 7779.6 (4883.3 to 12604.6) |  | 3393.5 (1594.6 to 5701.0) | 84355.7 (42752.7 to 139567.0) |  | 1701.7 (988.1 to 2559.0) | 70448.7 (40359.5 to 109317.1) |  | 12.9 (7.3 to 32.5) | 377.7 (207.7 to 1003.3) |  | 570.7 (215.0 to 1606.2) | 2794.8 (1712.5 to 4957.0) |
| **Southeast Asia, East Asia, and Oceania** | 301.1 (200.9 to 427.4) | 38607.3 (27969.9 to 49438.9) |  | 20719.3 (15989.3 to 27206.4) | 339837.2 (277133.8 to 434783.4) |  | 4395.1 (3143.6 to 5899.7) | 143846.1 (109890.5 to 187521.3) |  | 97.0 (78.5 to 123.9) | 1377.0 (1136.6 to 1745.2) |  | 3468.3 (2368.0 to 4950.2) | 5080.9 (3818.9 to 6655.9) |
| **East Asia** | 181.5 (126.5 to 231.6) | 19328.5 (13879.4 to 23856.7) |  | 15761.2 (11437.2 to 22082.8) | 207234.9 (152706.8 to 286797.0) |  | 3694.5 (2371.0 to 5148.8) | 104213.9 (70581.3 to 143438.7) |  | 84.3 (67.4 to 106.9) | 856.3 (703.9 to 1061.4) |  | 2761.5 (1617.5 to 4143.9) | 2576.1 (1411.9 to 3884.7) |
| China | 178.0 (123.7 to 227.2) | 18963.4 (13558.5 to 23387.9) |  | 15441.1 (11159.9 to 21725.7) | 202038.0 (149061.7 to 280692.5) |  | 3622.8 (2313.5 to 5059.9) | 101584.8 (68678.5 to 140556.2) |  | 83.3 (66.5 to 106.0) | 828.3 (678.2 to 1028.5) |  | 2690.6 (1600.0 to 4089.4) | 2509.1 (1356.4 to 3832.5) |
| Democratic People's Republic of Korea | 2.6 (1.1 to 4.9) | 290.1 (142.1 to 497.6) |  | 218.8 (123.4 to 377.2) | 4060.2 (2427.8 to 6606.5) |  | 33.9 (20.4 to 54.1) | 1701.9 (1045.6 to 2625.5) |  | 0.3 (0.2 to 0.5) | 14.7 (9.1 to 22.4) |  | 16.1 (4.8 to 42.8) | 28.1 (12.3 to 51.6) |
| Taiwan (Province of China) | 0.9 (0.5 to 1.4) | 74.9 (53.5 to 102.9) |  | 101.3 (68.4 to 146.3) | 1136.7 (837.3 to 1510.4) |  | 37.9 (22.1 to 57.3) | 927.1 (545.3 to 1246.2) |  | 0.7 (0.4 to 2.1) | 13.3 (7.7 to 40.8) |  | 54.8 (1.1 to 188.4) | 38.9 (5.3 to 120.7) |
| **Oceania** | 1.3 (0.7 to 2.2) | 188.0 (127.0 to 275.2) |  | 216.9 (85.8 to 420.3) | 5664.8 (2591.4 to 10636.4) |  | 51.3 (21.1 to 119.0) | 2984.5 (1214.9 to 6808.5) |  | 0.5 (0.3 to 1.4) | 27.6 (13.4 to 71.4) |  | 17.4 (9.1 to 33.3) | 100.4 (66.2 to 146.4) |
| American Samoa | 0.0 (0.0 to 0.0) | 0.3 (0.2 to 0.5) |  | 0.1 (0.1 to 0.2) | 3.6 (1.9 to 6.3) |  | 0.1 (0.0 to 0.2) | 4.6 (2.1 to 8.9) |  | 0.0 (0.0 to 0.0) | 0.0 (0.0 to 0.1) |  | 0.0 (0.0 to 0.0) | 0.0 (0.0 to 0.1) |
| Cook Islands | 0.0 (0.0 to 0.0) | 0.1 (0.0 to 0.1) |  | 0.0 (0.0 to 0.0) | 0.3 (0.1 to 0.7) |  | 0.0 (0.0 to 0.0) | 0.4 (0.1 to 0.8) |  | 0.0 (0.0 to 0.0) | 0.0 (0.0 to 0.0) |  | 0.0 (0.0 to 0.1) | 0.0 (0.0 to 0.1) |
| Fiji | 0.1 (0.0 to 0.1) | 8.5 (4.9 to 14.0) |  | 7.3 (4.5 to 11.5) | 262.9 (159.8 to 417.4) |  | 2.1 (1.0 to 4.6) | 115.9 (57.1 to 246.3) |  | 0.0 (0.0 to 0.0) | 0.9 (0.6 to 1.6) |  | 3.0 (1.0 to 7.7) | 13.5 (8.5 to 21.0) |
| Guam | 0.0 (0.0 to 0.0) | 1.2 (0.7 to 1.9) |  | 0.9 (0.6 to 1.4) | 18.4 (12.0 to 27.3) |  | 0.5 (0.2 to 1.1) | 21.9 (8.6 to 50.0) |  | 0.0 (0.0 to 0.0) | 0.1 (0.1 to 0.2) |  | 0.1 (0.0 to 0.3) | 0.3 (0.1 to 0.5) |
| Kiribati | 0.0 (0.0 to 0.0) | 1.9 (0.9 to 3.5) |  | 0.8 (0.4 to 1.5) | 19.7 (10.2 to 37.2) |  | 0.1 (0.1 to 0.3) | 7.8 (3.6 to 15.2) |  | 0.0 (0.0 to 0.0) | 0.1 (0.1 to 0.2) |  | 0.8 (0.3 to 1.9) | 7.3 (3.1 to 12.8) |
| Marshall Islands | 0.0 (0.0 to 0.0) | 0.4 (0.2 to 0.7) |  | 0.3 (0.1 to 0.4) | 7.3 (3.8 to 12.2) |  | 0.1 (0.0 to 0.2) | 5.4 (2.8 to 10.3) |  | 0.0 (0.0 to 0.0) | 0.1 (0.0 to 0.1) |  | 0.0 (0.0 to 0.1) | 0.2 (0.1 to 0.3) |
| Micronesia (Federated States of) | 0.0 (0.0 to 0.0) | 0.6 (0.2 to 1.0) |  | 0.3 (0.1 to 0.6) | 9.2 (2.4 to 17.4) |  | 0.1 (0.1 to 0.3) | 7.4 (3.3 to 13.9) |  | 0.0 (0.0 to 0.0) | 0.1 (0.0 to 0.1) |  | 0.1 (0.0 to 0.1) | 0.3 (0.1 to 0.5) |
| Nauru | 0.0 (0.0 to 0.0) | 0.2 (0.1 to 0.3) |  | 0.2 (0.1 to 0.3) | 3.7 (2.0 to 6.1) |  | 0.0 (0.0 to 0.1) | 2.2 (1.1 to 3.9) |  | 0.0 (0.0 to 0.0) | 0.0 (0.0 to 0.0) |  | 0.0 (0.0 to 0.1) | 0.1 (0.1 to 0.1) |
| Niue | 0.0 (0.0 to 0.0) | 0.0 (0.0 to 0.0) |  | 0.0 (0.0 to 0.0) | 0.3 (0.1 to 0.5) |  | 0.0 (0.0 to 0.0) | 0.2 (0.1 to 0.4) |  | 0.0 (0.0 to 0.0) | 0.0 (0.0 to 0.0) |  | 0.0 (0.0 to 0.0) | 0.0 (0.0 to 0.0) |
| Northern Mariana Islands | 0.0 (0.0 to 0.0) | 0.2 (0.1 to 0.3) |  | 0.1 (0.1 to 0.2) | 3.0 (1.7 to 4.8) |  | 0.1 (0.0 to 0.3) | 4.6 (2.0 to 9.4) |  | 0.0 (0.0 to 0.0) | 0.0 (0.0 to 0.0) |  | 0.0 (0.0 to 0.0) | 0.0 (0.0 to 0.0) |
| Palau | 0.0 (0.0 to 0.0) | 0.1 (0.0 to 0.1) |  | 0.0 (0.0 to 0.0) | 0.5 (0.3 to 0.8) |  | 0.0 (0.0 to 0.0) | 0.6 (0.3 to 1.1) |  | 0.0 (0.0 to 0.0) | 0.0 (0.0 to 0.0) |  | 0.0 (0.0 to 0.0) | 0.0 (0.0 to 0.0) |
| Papua New Guinea | 0.8 (0.4 to 1.3) | 108.9 (66.4 to 178.2) |  | 189.0 (65.6 to 380.8) | 4864.3 (1958.6 to 9569.7) |  | 43.0 (15.6 to 107.5) | 2530.5 (915.9 to 6200.6) |  | 0.4 (0.2 to 1.2) | 23.3 (10.4 to 62.7) |  | 8.2 (3.0 to 21.7) | 54.7 (29.8 to 94.9) |
| Samoa | 0.0 (0.0 to 0.0) | 1.7 (0.6 to 3.6) |  | 0.6 (0.2 to 1.3) | 12.9 (5.5 to 28.8) |  | 0.3 (0.1 to 0.7) | 14.5 (5 to 35.7) |  | 0.0 (0.0 to 0.0) | 0.3 (0.1 to 0.8) |  | 0.4 (0.1 to 1.2) | 1.3 (0.4 to 2.4) |
| Solomon Islands | 0.4 (0.2 to 0.7) | 50.9 (26.1 to 87.9) |  | 5.1 (2.5 to 8.8) | 133.2 (72.9 to 219.7) |  | 1.5 (0.8 to 3.2) | 86.0 (42.9 to 175.0) |  | 0.0 (0.0 to 0.0) | 0.8 (0.5 to 1.5) |  | 0.5 (0.2 to 1.2) | 2.7 (1.3 to 4.5) |
| Tokelau | 0.0 (0.0 to 0.0) | 0.0 (0.0 to 0.0) |  | 0.0 (0.0 to 0.0) | 0.1 (0.1 to 0.2) |  | 0.0 (0.0 to 0.0) | 0.1 (0.1 to 0.2) |  | 0.0 (0.0 to 0.0) | 0.0 (0.0 to 0.0) |  | 0.0 (0.0 to 0.0) | 0.0 (0.0 to 0.0) |
| Tonga | 0.0 (0.0 to 0.0) | 1.8 (0.8 to 3.4) |  | 0.4 (0.2 to 0.7) | 12.3 (6.6 to 21.5) |  | 0.1 (0.1 to 0.2) | 8.0 (4.2 to 14.1) |  | 0.0 (0.0 to 0.0) | 0.1 (0.1 to 0.2) |  | 3.3 (1.1 to 8.7) | 14.2 (8.7 to 22.9) |
| Tuvalu | 0.0 (0.0 to 0.0) | 0.1 (0.0 to 0.1) |  | 0.0 (0.0 to 0.1) | 0.9 (0.5 to 1.7) |  | 0.0 (0.0 to 0.0) | 0.8 (0.4 to 1.6) |  | 0.0 (0.0 to 0.0) | 0.0 (0.0 to 0.0) |  | 0.0 (0.0 to 0.0) | 0.0 (0.0 to 0.1) |
| Vanuatu | 0.0 (0.0 to 0.0) | 2.3 (1.2 to 3.9) |  | 1.5 (0.8 to 2.5) | 44.7 (23.3 to 71.8) |  | 0.6 (0.3 to 1.1) | 32.6 (16.9 to 63.6) |  | 0.0 (0.0 to 0.0) | 0.3 (0.2 to 0.6) |  | 0.1 (0.0 to 0.3) | 0.9 (0.5 to 1.4) |
| **Southeast Asia** | 118.3 (61.0 to 207.0) | 19090.8 (12787.1 to 27833.1) |  | 4741.1 (3415.4 to 6890.2) | 126937.4 (94127.5 to 185503.4) |  | 649.3 (465.8 to 880.5) | 36647.7 (26217.6 to 51027.4) |  | 12.1 (9.2 to 16.9) | 493.2 (373.8 to 714.9) |  | 689.3 (411.9 to 1161.4) | 2404.5 (1808.3 to 3130.4) |
| Cambodia | 43.4 (12.5 to 105.8) | 6492.4 (2697.7 to 13243.5) |  | 184.1 (101.3 to 294.5) | 4229.1 (2441.1 to 6722.6) |  | 22.2 (12.0 to 38.7) | 1211.9 (658.1 to 2105.7) |  | 0.3 (0.2 to 0.5) | 14.2 (10.0 to 23.3) |  | 11.1 (4.1 to 28.6) | 51.8 (33.6 to 76.7) |
| Indonesia | 33.6 (22.2 to 47.8) | 5764.5 (4206.0 to 7727.1) |  | 1667.5 (1109.1 to 2584.0) | 48317.1 (32987.5 to 75382.6) |  | 220.3 (149.0 to 319.6) | 13950.3 (9217.2 to 20416.3) |  | 3.4 (2.5 to 4.9) | 171.4 (123.4 to 251.0) |  | 150.7 (87.8 to 269.4) | 815.8 (567.6 to 1149.2) |
| Lao People's Democratic Republic | 0.6 (0.2 to 1.2) | 99.4 (54.0 to 162.6) |  | 77.0 (37.5 to 134.8) | 1951.1 (971.5 to 3298.0) |  | 9.9 (4.5 to 18.4) | 565.9 (261.6 to 1045.5) |  | 0.1 (0.1 to 0.2) | 6.5 (3.7 to 11.0) |  | 3.6 (1.4 to 8.7) | 21.6 (13.7 to 31.8) |
| Malaysia | 0.6 (0.2 to 1.1) | 114.8 (64.7 to 189.8) |  | 83.4 (32.1 to 160.9) | 2566.6 (1111.3 to 5006.8) |  | 19.3 (7.2 to 37.0) | 841.1 (319.4 to 1643.8) |  | 0.6 (0.3 to 1.0) | 15.0 (8.0 to 26.1) |  | 32.3 (6.9 to 89.0) | 71.2 (20.0 to 140.9) |
| Maldives | 0.0 (0.0 to 0.0) | 3.6 (2.2 to 5.8) |  | 4.4 (2.6 to 6.8) | 86.3 (52.3 to 134.5) |  | 0.9 (0.5 to 1.5) | 33.7 (19.2 to 54.7) |  | 0.0 (0.0 to 0.0) | 0.4 (0.3 to 0.7) |  | 0.2 (0.0 to 0.6) | 0.3 (0.2 to 0.6) |
| Mauritius | 0.0 (0.0 to 0.0) | 3.8 (2.6 to 5.3) |  | 7.3 (5.0 to 9.9) | 132.3 (96.7 to 174.5) |  | 0.9 (0.6 to 1.3) | 39.3 (26.5 to 54.3) |  | 0.0 (0.0 to 0.1) | 0.8 (0.5 to 1.6) |  | 1.3 (0.0 to 5.3) | 1.5 (0.3 to 5.4) |
| Myanmar | 24.8 (8.2 to 51.2) | 3755.8 (1869.5 to 6244.0) |  | 850.4 (364.0 to 1608.5) | 19852.6 (8749.2 to 37520.5) |  | 91.8 (42.8 to 180.5) | 5111.9 (2313.8 to 10269.5) |  | 1.3 (0.7 to 2.6) | 60.4 (33.0 to 124.3) |  | 45.5 (14.9 to 128.7) | 219.6 (118.0 to 433.2) |
| Philippines | 8.8 (5.8 to 11.9) | 1752.3 (1200.7 to 2230.7) |  | 1157.4 (797.7 to 1734.5) | 32816.6 (23881.7 to 46717.8) |  | 150.5 (109.6 to 215.5) | 9174.4 (6619.1 to 13075.5) |  | 2.8 (2.1 to 4.0) | 132.3 (98.8 to 190.6) |  | 167.1 (62.4 to 435.5) | 667.3 (481.2 to 937.9) |
| Seychelles | 0.0 (0.0 to 0.0) | 0.6 (0.3 to 0.9) |  | 0.4 (0.3 to 0.7) | 12.2 (8.0 to 18.5) |  | 0.1 (0.1 to 0.2) | 6.2 (3.9 to 9.3) |  | 0.0 (0.0 to 0.0) | 0.1 (0.0 to 0.2) |  | 0.1 (0.0 to 0.3) | 0.2 (0.1 to 0.4) |
| Sri Lanka | 0.9 (0.4 to 1.6) | 142.5 (85.1 to 234.6) |  | 82.1 (45.3 to 146.5) | 1853.2 (1098.7 to 2917.4) |  | 14.0 (7.7 to 22.0) | 548.1 (313.3 to 823.0) |  | 0.5 (0.3 to 0.7) | 10.6 (7.0 to 14.9) |  | 32.8 (6.5 to 96.8) | 56.7 (17.0 to 105.9) |
| Thailand | 1.2 (0.5 to 2.4) | 200.6 (109.1 to 321.0) |  | 353.6 (160.7 to 540.0) | 4802.2 (2221.1 to 7242.8) |  | 49.4 (20.3 to 79.6) | 1966.2 (842.6 to 3184.5) |  | 1.1 (0.5 to 1.6) | 23.9 (11.4 to 35.2) |  | 99.2 (18.8 to 286.1) | 186.0 (53.0 to 342.6) |
| Timor-Leste | 0.1 (0.0 to 0.2) | 20.4 (8.8 to 32.2) |  | 18.6 (4.4 to 33.5) | 439.9 (113.4 to 782.4) |  | 2.2 (0.6 to 3.9) | 122.5 (34.9 to 220.5) |  | 0.0 (0.0 to 0.0) | 1.3 (0.4 to 2.3) |  | 0.9 (0.2 to 2.4) | 5.2 (1.6 to 8.2) |
| Viet Nam | 4.1 (1.9 to 7.3) | 715.2 (426.3 to 1102.0) |  | 248.8 (133.0 to 439.2) | 9712.0 (5561.7 to 15897.8) |  | 67.0 (38.3 to 114) | 3028.5 (1784.2 to 4987.3) |  | 2.0 (1.3 to 2.9) | 55.5 (38.6 to 79.3) |  | 143.7 (41.3 to 400.0) | 304.1 (179.2 to 540.5) |
| **Sub-Saharan Africa** | 493.7 (261.2 to 819.1) | 60937.4 (38395.1 to 88576.8) |  | 17878.2 (11500.3 to 24581.6) | 597733.3 (404034.2 to 812758.1) |  | 2999.4 (1939.9 to 4630.4) | 174632.0 (113018.8 to 267890.3) |  | 91.1 (37.6 to 268.8) | 4187.5 (1643.3 to 13398.8) |  | 1196.5 (510.7 to 3750.6) | 8722.9 (4344.4 to 23365.1) |
| **Central Sub-Saharan Africa** | 80.1 (27.0 to 171.8) | 9501.3 (4022.7 to 17804.5) |  | 1225.3 (555.9 to 2202.1) | 34551.0 (16398.9 to 63312.6) |  | 199.3 (92.6 to 423.0) | 11855.9 (5452.3 to 25051.5) |  | 6.9 (2.5 to 21.1) | 324.3 (116.3 to 1023.7) |  | 101.0 (39.8 to 306.4) | 809.8 (422.4 to 1806.4) |
| Angola | 19.2 (6.9 to 42.1) | 2346.2 (1025.0 to 4621.9) |  | 415.6 (174.1 to 743.1) | 11670.5 (4961.8 to 20392.5) |  | 69.9 (29.9 to 148.6) | 4138.1 (1781.1 to 8722.2) |  | 2.4 (0.9 to 6.6) | 108.5 (39.4 to 295.1) |  | 37.9 (13.0 to 113.4) | 288.1 (156.6 to 587.4) |
| Central African Republic | 3.2 (1.2 to 6.4) | 375.9 (176.0 to 688.7) |  | 93.7 (24.5 to 219.0) | 2912.4 (812.8 to 6315.3) |  | 13.9 (3.5 to 38.1) | 848.8 (212.3 to 2310.4) |  | 0.5 (0.1 to 1.9) | 25.8 (4.8 to 103.9) |  | 4.3 (0.9 to 20.5) | 40.1 (12.7 to 134.9) |
| Congo | 1.3 (0.5 to 2.8) | 166.9 (74.3 to 314.7) |  | 28.4 (13.2 to 53.4) | 811.1 (393.0 to 1530.6) |  | 5.2 (2.6 to 10.2) | 305.2 (154.5 to 600.5) |  | 0.2 (0.1 to 0.5) | 7.8 (3.3 to 21.2) |  | 3.6 (1.1 to 11.8) | 26.2 (12.3 to 63.6) |
| Democratic Republic of the Congo | 55.6 (15.3 to 133.4) | 6511.2 (2174.8 to 14540.2) |  | 673.1 (283.4 to 1373.1) | 18746.9 (8237.1 to 37402.0) |  | 107.4 (44.3 to 242.7) | 6399.3 (2638.0 to 14408.2) |  | 3.7 (1.2 to 11.9) | 178.2 (59.7 to 587.9) |  | 52.1 (14.5 to 182.3) | 437.6 (186.3 to 1043.2) |
| Equatorial Guinea | 0.3 (0.1 to 0.9) | 46.3 (16.5 to 102.7) |  | 6.0 (2.3 to 12.2) | 166.0 (67.4 to 330.6) |  | 1.1 (0.4 to 2.2) | 64.7 (26.0 to 124.2) |  | 0.0 (0.0 to 0.1) | 1.6 (0.6 to 3.5) |  | 1.3 (0.3 to 3.7) | 7.0 (2.9 to 14.2) |
| Gabon | 0.4 (0.1 to 0.9) | 54.8 (18.9 to 116.9) |  | 8.4 (3.7 to 15.1) | 244.2 (111.1 to 443.6) |  | 1.7 (0.8 to 3.5) | 99.8 (42.8 to 198.7) |  | 0.1 (0.0 to 0.1) | 2.5 (1.1 to 5.6) |  | 1.9 (0.5 to 6.0) | 10.8 (4.7 to 22.6) |
| **Eastern Sub-Saharan Africa** | 232.3 (121.4 to 384.1) | 27181.4 (16533.9 to 40044.4) |  | 12466.5 (6869.5 to 18332.1) | 409051.4 (224471.7 to 591909.8) |  | 1414.1 (999.7 to 2005.9) | 83004.4 (58755.6 to 116935.1) |  | 39.1 (16.5 to 112.5) | 1723.2 (724.5 to 5103.3) |  | 440.0 (203.1 to 1169.1) | 3177.2 (1849.7 to 6286.9) |
| Burundi | 4.8 (1.7 to 10.3) | 533.7 (234.3 to 1022.9) |  | 296.2 (124.6 to 606.5) | 8950.9 (3949.0 to 18861.4) |  | 38.5 (19.9 to 72.1) | 2310.4 (1200.5 to 4268.5) |  | 1.1 (0.3 to 4.2) | 52.9 (13.8 to 205.1) |  | 9.5 (2.1 to 38.3) | 76.8 (26.3 to 240.1) |
| Comoros | 0.2 (0.1 to 0.3) | 18.5 (8.3 to 33.8) |  | 10.6 (5.0 to 21.8) | 313.9 (144.5 to 671.4) |  | 1.4 (0.7 to 2.4) | 83.3 (43.6 to 140.5) |  | 0.0 (0.0 to 0.1) | 1.4 (0.7 to 3.4) |  | 0.4 (0.1 to 1.2) | 3.1 (1.7 to 5.4) |
| Djibouti | 0.4 (0.2 to 1.0) | 53.1 (24.7 to 100.9) |  | 32.6 (15.9 to 57.7) | 935.2 (470.5 to 1736.6) |  | 4.3 (2.5 to 6.8) | 247.3 (142.1 to 395.0) |  | 0.1 (0.0 to 0.3) | 4.8 (2.0 to 12.3) |  | 1.8 (0.5 to 5.6) | 11.3 (5.7 to 24.1) |
| Eritrea | 2.3 (0.8 to 5.0) | 256.1 (111.1 to 510.3) |  | 138.0 (63.5 to 277.8) | 4380.5 (1997.8 to 8734.6) |  | 19.6 (11.6 to 32.7) | 1174.4 (702.8 to 1946.0) |  | 0.6 (0.2 to 1.8) | 30.6 (10.4 to 86.8) |  | 6.3 (2.0 to 19.6) | 52.0 (24.3 to 114.5) |
| Ethiopia | 48.5 (28.0 to 79.7) | 5418.3 (3392.1 to 8543.3) |  | 4408.3 (1805.1 to 7815.4) | 168402.1 (73375.4 to 280340.9) |  | 302.9 (138.5 to 584.4) | 17144.1 (7838.5 to 32825.1) |  | 5.3 (2.8 to 16.4) | 247.9 (126.7 to 778.3) |  | 99.8 (38.7 to 253.7) | 753.0 (435.4 to 1255.9) |
| Kenya | 6.3 (4.0 to 9.2) | 702.7 (494.5 to 1002.4) |  | 525.1 (273.0 to 766.8) | 15418.2 (8272.7 to 23090.9) |  | 89.2 (60.5 to 131.7) | 4944.0 (3388.6 to 7269.4) |  | 1.5 (0.9 to 3.2) | 55.7 (33.2 to 122.8) |  | 24.6 (10.3 to 58.6) | 139.3 (83.8 to 252.6) |
| Madagascar | 7.2 (2.7 to 15.4) | 818.6 (399.4 to 1590.4) |  | 401.4 (223.4 to 679.9) | 12558.6 (7104.6 to 21055.0) |  | 52.7 (32.8 to 80.7) | 3192.4 (1990.0 to 4874.5) |  | 1.5 (0.5 to 4.9) | 69.9 (23.0 to 226.8) |  | 14.9 (5.1 to 49.8) | 122.5 (62.6 to 284.1) |
| Malawi | 19.1 (9.2 to 32.8) | 2368.9 (1384.0 to 3776.4) |  | 253.8 (133.9 to 464.1) | 8265.1 (4337.2 to 15181.5) |  | 48.8 (28.4 to 75.3) | 2896.8 (1704.6 to 4467.1) |  | 3.6 (1.4 to 8.7) | 156.7 (60.3 to 379.4) |  | 17.5 (5.7 to 55.8) | 132.4 (67.8 to 275.4) |
| Mozambique | 27.2 (9.9 to 57.6) | 3260.8 (1473.7 to 6219.3) |  | 1369.5 (622.8 to 2936.7) | 41264.3 (18466.2 to 88169.5) |  | 187.2 (81.3 to 346.6) | 11329.9 (4927.5 to 20975.5) |  | 3.2 (1.2 to 8.2) | 152.5 (58.2 to 384.6) |  | 34.7 (12.5 to 91.1) | 274.8 (153.7 to 472.2) |
| Rwanda | 6.5 (2.2 to 14.1) | 756.7 (296.1 to 1502.1) |  | 273.6 (126.7 to 529.0) | 8190.5 (3885.3 to 15480.2) |  | 39.9 (22.7 to 63.4) | 2332.8 (1341.8 to 3711.9) |  | 1.2 (0.4 to 3.3) | 49.9 (17.1 to 134.8) |  | 14.2 (4.3 to 44.0) | 97.3 (48.5 to 195.6) |
| Somalia | 11.3 (4.3 to 23.5) | 1275.5 (621.9 to 2390.0) |  | 651.9 (323.0 to 1136.4) | 20992.2 (11294.0 to 35752.5) |  | 75.2 (42.1 to 120.0) | 4610.9 (2602.5 to 7281.6) |  | 2.2 (0.6 to 7.7) | 116.4 (30.2 to 415.3) |  | 13.6 (4.0 to 41.6) | 132.5 (53.3 to 285.8) |
| South Sudan | 4.4 (1.7 to 8.9) | 516.3 (267.6 to 939.3) |  | 367.6 (217.3 to 583.9) | 11505.4 (7115.9 to 16900.3) |  | 36.5 (21.0 to 61.2) | 2188.5 (1256.8 to 3654.6) |  | 1.1 (0.3 to 3.4) | 53.0 (14.3 to 165.6) |  | 11.3 (2.9 to 45.2) | 91.6 (43.3 to 244.2) |
| Uganda | 34.7 (14.0 to 72.3) | 4042.9 (1959.1 to 7645.6) |  | 367.6 (217.3 to 583.9) | 11505.4 (7115.9 to 16900.3) |  | 36.5 (21.0 to 61.2) | 2188.5 (1256.8 to 3654.6) |  | 6.1 (1.8 to 16.7) | 261.1 (79.5 to 717.0) |  | 57.0 (17.3 to 204.3) | 400.8 (183.3 to 1000.2) |
| United Republic of Tanzania | 51.1 (18.0 to 112.5) | 6132.8 (2621.5 to 11596.7) |  | 888.7 (452.5 to 1584.8) | 24377.0 (12405.8 to 44083.5) |  | 130.3 (67.5 to 218.1) | 7716.0 (3994.4 to 12911.5) |  | 9.5 (3.3 to 28.6) | 391.3 (138.3 to 1194.7) |  | 106.8 (32.6 to 352.7) | 714.5 (374.0 to 1549.2) |
| Zambia | 8.3 (3.2 to 16.5) | 1004.7 (486.2 to 1779.5) |  | 2381.9 (1259.7 to 4202.1) | 69072.6 (39385.5 to 119905.4) |  | 326.7 (198.2 to 507.0) | 19230.2 (11714.2 to 29772.3) |  | 1.9 (0.8 to 4.8) | 77.6 (33.1 to 199.1) |  | 27.4 (9.0 to 87.7) | 172.9 (90.2 to 367.2) |
| **Southern Sub-Saharan Africa** | 7.5 (3.8 to 14.0) | 919.1 (561.5 to 1489.1) |  | 457.4 (225.2 to 884.6) | 14097.9 (6941.1 to 26907.9) |  | 59.8 (33.6 to 97.6) | 3537.0 (2010.3 to 5768.0) |  | 5.9 (3.4 to 10.8) | 194.0 (106.8 to 357.9) |  | 99.2 (61.1 to 161.8) | 463.1 (334.9 to 628.7) |
| Botswana | 0.0 (0.0 to 0.1) | 5.2 (3.2 to 8.2) |  | 181.1 (127.7 to 252.0) | 4400.2 (3133.2 to 6088.1) |  | 49.1 (32.2 to 69.4) | 2478.3 (1603.4 to 3456.0) |  | 0.2 (0.1 to 0.4) | 5.3 (2.6 to 10.6) |  | 4.0 (1.0 to 11.6) | 24.3 (14.2 to 42.3) |
| Eswatini | 0.1 (0.0 to 0.3) | 17.8 (7.4 to 34.0) |  | 10.1 (4.5 to 19.6) | 251.6 (112.3 to 486.5) |  | 2.4 (1.2 to 4.5) | 126.7 (63.2 to 235.5) |  | 0.1 (0.1 to 0.2) | 4.3 (2.2 to 7.9) |  | 1.5 (0.6 to 3.3) | 10.3 (6.8 to 15.9) |
| Lesotho | 0.2 (0.1 to 0.3) | 20.4 (9.7 to 38.8) |  | 3.8 (2.1 to 6.6) | 111.7 (63.8 to 188.0) |  | 1.0 (0.5 to 1.6) | 56.9 (31.6 to 95.2) |  | 0.1 (0.1 to 0.3) | 6.1 (2.9 to 12.1) |  | 1.7 (0.7 to 3.5) | 14.1 (9.2 to 20.2) |
| Namibia | 0.3 (0.1 to 0.6) | 36.5 (17.9 to 67.2) |  | 5.4 (2.9 to 9.5) | 159.7 (87.7 to 280.3) |  | 1.3 (0.7 to 2.4) | 79.9 (42.7 to 141.7) |  | 0.5 (0.2 to 1.0) | 16.0 (6.5 to 32.5) |  | 3.3 (1.1 to 7.8) | 18.6 (8.9 to 30.2) |
| South Africa | 2.0 (1.3 to 2.8) | 244.7 (177.6 to 334.3) |  | 5.3 (2.7 to 8.9) | 140.0 (72.9 to 235.6) |  | 2.0 (1.1 to 3.4) | 114.0 (58.8 to 191.1) |  | 3.2 (2.0 to 6.3) | 86.7 (52.1 to 176.7) |  | 78.2 (44.7 to 133.4) | 304.1 (212.2 to 427.1) |
| Zimbabwe | 5.0 (1.8 to 10.8) | 594.5 (286.7 to 1093.6) |  | 111.7 (75.7 to 158.2) | 2331.6 (1660 to 3153.6) |  | 31.0 (20.6 to 42.8) | 1421.2 (954.8 to 1970.8) |  | 1.7 (0.7 to 3.6) | 75.6 (28.8 to 156.6) |  | 10.5 (4.3 to 23.0) | 91.8 (52.5 to 144.1) |
| **Western Sub-Saharan Africa** | 173.8 (96.3 to 271.0) | 23335.6 (14660.7 to 32878.7) |  | 44.9 (26.2 to 72.0) | 1405.6 (814.5 to 2251.3) |  | 11.4 (5.9 to 19.1) | 679.7 (348.7 to 1123.7) |  | 39.3 (13.8 to 129.8) | 1946.0 (637.4 to 6840.5) |  | 556.3 (159.3 to 2272.0) | 4272.8 (1578.7 to 15160.3) |
| Benin | 4.3 (1.8 to 8.0) | 588.9 (317.5 to 964.7) |  | 4005.2 (2036.5 to 6341.6) | 149730.7 (80487.4 to 237573.0) |  | 1337.0 (682.0 to 2502.5) | 77293.4 (39010.4 to 145137.3) |  | 1.2 (0.5 to 3.5) | 54.8 (20.8 to 153.7) |  | 21.7 (5.0 to 99.3) | 163.7 (59.0 to 558.3) |
| Burkina Faso | 27.5 (10.8 to 53.1) | 3511.5 (1761.6 to 6121.2) |  | 188.5 (87.2 to 363.1) | 6900.7 (3261.6 to 13381.6) |  | 46.3 (18.8 to 101.3) | 2739.6 (1123.4 to 6029.2) |  | 3.4 (1.0 to 11.0) | 152.9 (41.4 to 505.8) |  | 42.1 (8.4 to 207.3) | 315.9 (105.2 to 1205.5) |
| Cabo Verde | 0.1 (0.0 to 0.1) | 7.8 (3.3 to 14.6) |  | 481.8 (186.3 to 934.8) | 16452.3 (7046.9 to 32853.9) |  | 114.1 (37.3 to 274.5) | 6779.8 (2235.0 to 16404.4) |  | 0.0 (0.0 to 0.1) | 0.7 (0.3 to 1.3) |  | 0.4 (0.1 to 1.1) | 1.1 (0.4 to 2.2) |
| Cameroon | 0.6 (0.3 to 1.1) | 87.1 (50.7 to 136.9) |  | 2.5 (1.1 to 4.6) | 77.0 (34.5 to 144.8) |  | 1.5 (0.4 to 2.7) | 74.5 (20.8 to 132.6) |  | 1.4 (0.7 to 3.9) | 58.5 (27.6 to 158.3) |  | 27.8 (5.8 to 130.5) | 268.2 (76.9 to 1037.3) |
| Chad | 6.1 (2.6 to 11.9) | 775.3 (431.0 to 1299.8) |  | 248.8 (116.9 to 441.0) | 8717.3 (3999.1 to 16200.3) |  | 65.7 (27.2 to 139.5) | 3856.4 (1607 to 8182.6) |  | 1.6 (0.5 to 5.1) | 78.5 (22.6 to 257.9) |  | 20.9 (4.2 to 100.6) | 180.1 (55.8 to 662.5) |
| Côte d'Ivoire | 5.3 (2.2 to 10.0) | 736.1 (369.3 to 1238.3) |  | 239.8 (87.8 to 460.4) | 8775.8 (3393.8 to 17383.2) |  | 54.8 (19.6 to 140.9) | 3307.3 (1192.0 to 8461.2) |  | 1.6 (0.6 to 3.9) | 70.1 (24.9 to 183.1) |  | 29.8 (7.1 to 130.6) | 230.4 (84.2 to 729.3) |
| Gambia | 4.6 (1.6 to 10.1) | 607.2 (276.2 to 1177.5) |  | 207.8 (94.9 to 386.4) | 7822.3 (3623.3 to 14542.8) |  | 59.3 (25.5 to 116.1) | 3474.7 (1497.8 to 6855.6) |  | 0.1 (0.1 to 0.2) | 4.3 (2.5 to 8.1) |  | 2.3 (0.7 to 8.6) | 17.7 (7.8 to 39.5) |
| Ghana | 10.4 (3.9 to 22.4) | 1432.3 (672.1 to 2644.9) |  | 7.2 (3.3 to 13.0) | 311.9 (143.7 to 585.2) |  | 2.9 (1.1 to 5.6) | 172.9 (65.7 to 328.7) |  | 1.4 (0.7 to 2.6) | 51.2 (24.8 to 97.4) |  | 40.1 (8.0 to 175.2) | 247.3 (82.4 to 764.4) |
| Guinea | 31.2 (11.1 to 67.8) | 3849.8 (1761.6 to 7621.5) |  | 110.6 (53.1 to 198.2) | 5002.2 (2423.5 to 8872.4) |  | 66.9 (30.5 to 123.0) | 3867.1 (1780.1 to 7123.8) |  | 3.6 (0.8 to 12.7) | 178.1 (38.9 to 628.8) |  | 20.9 (4.8 to 90.7) | 168.8 (60.9 to 578.0) |
| Guinea-Bissau | 0.4 (0.2 to 0.7) | 51.5 (26.2 to 89.2) |  | 75.4 (40.7 to 134.2) | 3334.8 (1777.4 to 5925.6) |  | 25.6 (12.0 to 47.2) | 1536.4 (719.2 to 2828.0) |  | 0.1 (0.0 to 0.2) | 4.4 (1.5 to 12.2) |  | 1.5 (0.3 to 6.4) | 12.9 (4.4 to 43.7) |
| Liberia | 0.6 (0.2 to 1.3) | 87.8 (44.8 to 157.9) |  | 12.3 (6.2 to 21.9) | 478.5 (242.0 to 855.0) |  | 3.6 (1.7 to 7.7) | 213.7 (99.0 to 458.4) |  | 0.2 (0.1 to 0.4) | 6.7 (2.8 to 18.8) |  | 2.6 (0.6 to 11.3) | 20.3 (7.7 to 61.9) |
| Mali | 20.7 (8.3 to 41.7) | 2537.9 (1332.7 to 4625.8) |  | 21.4 (10.6 to 37.0) | 815.8 (407.3 to 1419.4) |  | 6.4 (2.7 to 12.3) | 385.6 (162.3 to 741.2) |  | 4.1 (1.1 to 12.7) | 187.9 (47.2 to 583.7) |  | 45.9 (8.2 to 254.1) | 345.6 (110.6 to 1359.1) |
| Mauritania | 0.4 (0.1 to 0.8) | 53.8 (24.5 to 101.3) |  | 195.3 (111.8 to 345.2) | 8104.1 (4505.0 to 14535.7) |  | 65.2 (24.1 to 136.3) | 3886.4 (1428.7 to 8061.4) |  | 0.1 (0.1 to 0.2) | 3.9 (1.8 to 9.0) |  | 2.4 (0.6 to 9.0) | 14.9 (6.3 to 37.6) |
| Niger | 0.7 (0.3 to 1.2) | 97.1 (56.0 to 173.6) |  | 11.5 (5.8 to 20.2) | 428.5 (224.4 to 717.2) |  | 3.7 (1.4 to 7.2) | 212.5 (79.9 to 415.9) |  | 2.0 (0.8 to 6.2) | 96.5 (34.3 to 314.7) |  | 28.6 (5.8 to 135.6) | 266.3 (86.0 to 998.9) |
| Nigeria | 54.3 (27.9 to 80.8) | 8008.7 (3926.2 to 12021.0) |  | 422.0 (155.2 to 901.8) | 14026.4 (5736.9 to 30122.9) |  | 84.6 (30.7 to 198.4) | 5067.5 (1842.4 to 11929.8) |  | 16.5 (5.3 to 64.9) | 915.4 (265.3 to 3793.7) |  | 236.2 (45.4 to 1147.4) | 1771.8 (544.0 to 6728.4) |
| Sao Tome and Principe | 0.0 (0.0 to 0.0) | 2.5 (1.0 to 5.1) |  | 1510.2 (650.5 to 2687.3) | 58536.3 (27053.3 to 100440.7) |  | 658.2 (305.8 to 1341.6) | 37081.4 (16793.9 to 76294.6) |  | 0.0 (0.0 to 0.0) | 0.3 (0.2 to 0.5) |  | 0.2 (0.1 to 0.5) | 1.1 (0.4 to 1.9) |
| Senegal | 2.3 (0.9 to 4.6) | 321.6 (152.1 to 579.3) |  | 0.5 (0.2 to 1.1) | 16.8 (5.1 to 36.6) |  | 0.1 (0.0 to 0.2) | 4.9 (1.6 to 8.7) |  | 0.7 (0.3 to 1.4) | 29.6 (14.8 to 63.0) |  | 12.3 (3.7 to 38.0) | 94.4 (41.7 to 204.8) |
| Sierra Leone | 3.1 (1.3 to 6.1) | 415.4 (224.1 to 717.3) |  | 90.5 (47.7 to 151.0) | 3432.7 (1838.3 to 5745.6) |  | 28.2 (11.5 to 54.3) | 1665.4 (685.4 to 3194.5) |  | 0.9 (0.3 to 2.5) | 38.7 (12.7 to 112.1) |  | 13.6 (2.9 to 63.9) | 104.3 (35.0 to 388.0) |
| Togo | 1.2 (0.5 to 2.3) | 162.9 (90.7 to 275.8) |  | 140.6 (60.1 to 287.3) | 5090.1 (2179.4 to 10533.7) |  | 39.1 (13.9 to 97.4) | 2333.7 (826.6 to 5836.3) |  | 0.3 (0.1 to 1.1) | 13.7 (4.9 to 46.6) |  | 6.9 (1.3 to 37.6) | 47.9 (13.9 to 205.2) |

Data in parentheses are 95% uncertainty intervals (UIs) unless otherwise stated.

Abbreviations: Cases = incidence cases; DALYs = disability-adjusted life-years; SDI = Sociodemographic index.

**Supplementary Table 5.** Incidence cases and DALYs of specific cancers among children under 5-year-old globally, and by SDI regions, GBD super-regions, GBD regions, countries and territories in 2019 (continue).

|  | **Kidney cancer** | |  | **Hodgkin lymphoma** | |  | **Non-Hodgkin lymphoma** | |  | **Other malignant neoplasms** | |  | **Other neoplasms** | |
| --- | --- | --- | --- | --- | --- | --- | --- | --- | --- | --- | --- | --- | --- | --- |
|  | **Cases** | **DALYs** |  | **Cases** | **DALYs** |  | **Cases** | **DALYs** |  | **Cases** | **DALYs** |  | **Cases** | **DALYs** |
| **Global** | 7890.4 (6453.0 to 9400.8) | 155753.9 (119112.4 to 197182.9) |  | 558.1 (417.8 to 708.5) | 13156.2 (7363.2 to 19717.5) |  | NA | 165134.3 (130258.6 to 203800.4) |  | 19029.4 (16160.6 to 22410.6) | 1161199.8 (894574.6 to 1484588.5) |  | 8650139.5 (6123797.2 to 11626471.1) | 23301.3 (18919.8 to 28932.3) |
| High SDI | 686.2 (551.0 to 846.7) | 5840.6 (4964.1 to 6685.9) |  | 106.7 (86.3 to 129.2) | 388.0 (340.9 to 434.6) |  | NA | 3989.8 (3396.0 to 4780.3) |  | 2104.1 (1624.8 to 2808.6) | 38957.5 (34680.8 to 43981.8) |  | 1393829.7 (1015587.4 to 1849271.0) | 2656.3 (1930.8 to 3151.6) |
| High-middle SDI | 1540.8 (1303.9 to 1826.2) | 18628.2 (16061.3 to 21526.2) |  | 109.1 (87.7 to 134.0) | 894.0 (729.8 to 1076.1) |  | NA | 14528.8 (12123.8 to 17495) |  | 3295.5 (2734.4 to 4064.3) | 78242.4 (67861.7 to 90358.3) |  | 1888145.1 (1355352.0 to 2530711.5) | 3974.2 (3069.0 to 4801.0) |
| Middle SDI | 2376.8 (1960.8 to 2849.1) | 38711.9 (31812.2 to 46106.8) |  | 143.1 (110.9 to 178.2) | 2404.8 (1826.2 to 3020.8) |  | NA | 34199.8 (27286.2 to 42756) |  | 5673.0 (4482.7 to 6984.0) | 195455.3 (163890.5 to 232332.6) |  | 2464943.3 (1741640.0 to 3302735.1) | 7785.3 (6187.5 to 9755.9) |
| Low-middle SDI | 1280.5 (972.9 to 1613.7) | 30974.0 (22991.5 to 39707.5) |  | 74.5 (48.4 to 118.6) | 2782.9 (1701.2 to 4517.8) |  | NA | 46575.1 (35772.6 to 59102.3) |  | 3616.6 (2796.4 to 4501.4) | 286987.7 (217837.7 to 369979.0) |  | 1427053.3 (988363.3 to 1948901.5) | 5261.5 (3974.0 to 7021.2) |
| Low SDI | 1677.1 (1070.0 to 2370.0) | 61465.4 (36511.6 to 90278.2) |  | 100.4(40.4 to 168.8) | 6680.4 (2361.6 to 11792.5) |  | NA | 65676.3 (40139.0 to 94278.2) |  | 4163.8 (3202.6 to 5458.9) | 560621.1 (385638.3 to 788704.5) |  | 1297786.0 (890007.2 to 1795489.1) | 3595.8 (2333.8 to 6595.5) |
| **Central Europe, Eastern Europe, and Central Asia** | 420.8 (342.0 to 500.5) | 6400.8 (5155.7 to 7696.4) |  | 39.6 (25.5 to 50.7) | 455.5 (260.3 to 602.8) |  | NA | 4641.9 (3816.0 to 5719.4) |  | 834.3 (603.9 to 1124.0) | 29029.4 (24206.5 to 35130.4) |  | 1268430.3 (908408.5 to 1710560.4) | 574.4 (358.7 to 740.8) |
| **Central Asia** | 113.5 (84.8 to 147.1) | 2310.0 (1759.8 to 2975.1) |  | 11.0 (5.5 to 16.7) | 227.9 (111.8 to 350.2) |  | NA | 1376.0 (1000.6 to 1873.2) |  | 142.9 (99.3 to 187.8) | 10342.6 (8163.3 to 13065.3) |  | 364714.4 (255666.2 to 500589.8) | 187.5 (108.1 to 270.3) |
| Armenia | 3.1 (2.0 to4.6) | 55.4 (36.4 to 81.2) |  | 0.1 (0.1 to 0.2) | 1.8 (1.4 to 2.5) |  | NA | 60.6 (38.4 to 88.6) |  | 4.2 (2.8 to 5.8) | 339.2 (233.3 to 462.3) |  | 7749.4 (5381.6 to 10661.9) | 2.4 (1.7 to 4.0) |
| Azerbaijan | 10.3 (5.2 to 19.5) | 216.4 (109.4 to 416.2) |  | 0.8 (0.3 to 2.2) | 17.8 (6.3 to 49.8) |  | NA | 74.0 (31.3 to 172.8) |  | 11.3 (5.6 to 22.0) | 853.3 (481.1 to 1451.5) |  | 28799.4 (19992.2 to 39635.4) | 11.2 (5.6 to 20.6) |
| Georgia | 1.7 (1.0 to 2.5) | 31.2 (19.8 to 46.5) |  | 0.9 (0.3 to 1.7) | 15.4 (5.6 to 28.3) |  | NA | 55.8 (35.2 to 86.0) |  | 4.8 (2.8 to 7.6) | 335.8 (222.8 to 476.0) |  | 8738.3 (6818.1 to 11132.7) | 14.1 (2.9 to 26.6) |
| Kazakhstan | 27.4 (17.9 to 40.6) | 495.7 (329.4 to 738.5) |  | 1.4 (0.9 to 2.3) | 20.5 (13.3 to 31.0) |  | NA | 295.0 (189.8 to 427.8) |  | 42.0 (24.2 to 65.4) | 2626.8 (1838.1 to 3619.6) |  | 69833.9 (48446.0 to 96095.2) | 85.1 (27.7 to 145.6) |
| Kyrgyzstan | 8.2 (5.4 to 11.7) | 170.8 (116.1 to 238.2) |  | 0.3 (0.2 to 0.4) | 6.4 (5.3 to 7.5) |  | NA | 58.1 (39.1 to 81.4) |  | 6.3 (3.8 to 9.3) | 540.2 (421.8 to 769.1) |  | 30844.3 (21493.0 to 42703.2) | 9.1 (7.2 to 11.4) |
| Mongolia | 2.0 (1.2 to 3.4) | 48.2 (29.3 to 77.8) |  | 0.1 (0.0 to 0.1) | 1.7 (1.0 to 3.0) |  | NA | 30.3 (15.3 to 58.2) |  | 3.5 (1.9 to 7.1) | 275.3 (173.0 to 485.4) |  | 14954.8 (10360.1 to 20605.9) | 3.6 (2.6 to 4.8) |
| Tajikistan | 4.5 (2.4 to 7.7) | 114.7 (64.0 to 197.2) |  | 0.2 (0.1 to 0.4) | 6.9 (3.9 to 12.7) |  | NA | 112.8 (62.2 to 200.3) |  | 9.2 (5.8 to 14.0) | 1128.8 (751.6 to 1708.3) |  | 45758.8 (31705.4 to 63076.2) | 14.0 (10.4 to 18.4) |
| Turkmenistan | 10.1 (7.0 to 14.0) | 211.8 (151.3 to 293.2) |  | 1.0 (0.3 to 1.8) | 25.0 (7.5 to 40.3) |  | NA | 56.7 (37.2 to 82.1) |  | 12.9 (9.2 to 17.2) | 948.1 (720.0 to 1222.5) |  | 20893.9 (14484.0 to 28787.4) | 9.1 (5.5 to 17.4) |
| Uzbekistan | 46.3 (30.6 to 66.5) | 965.7 (672.6 to 1348.6) |  | 6.1 (1.9 to 11.1) | 132.4 (42.4 to 239.1) |  | NA | 632.8 (413.2 to 952.5) |  | 48.6 (31.9 to 65.9) | 3295.1 (2538.2 to 4273.2) |  | 137141.8 (95146.0 to 188840.4) | 39.1 (29.7 to 59.4) |
| **Central Europe** | 66.5 (52.0 to 83.9) | 981.7 (775.2 to 1234.6) |  | 8.1 (5.9 to 11.0) | 55.3 (41.1 to 71.8) |  | NA | 860.9 (690.5 to 1075.5) |  | 102.8 (76.7 to 144.8) | 4650.6 (3706.4 to 5977.4) |  | 251207.7 (181742.4 to 335715.7) | 109.9 (73.6 to 144.8) |
| Albania | 3.7 (2.1 to 7.3) | 51.7 (30.5 to 94.3) |  | 0.3 (0.2 to 0.5) | 2.3 (1.4 to 3.6) |  | NA | 39.9 (23.4 to 61.0) |  | 14.0 (9.0 to 20.5) | 508.5 (360.7 to 764.4) |  | 8853.3 (6128.3 to 12074.3) | 4.4 (2.5 to 7.2) |
| Bosnia and Herzegovina | 2.3 (1.5 to 3.4) | 40.0 (26.0 to 58.3) |  | 0.2 (0.0 to 0.5) | 1.4 (0.5 to 4.1) |  | NA | 35.2 (21.7 to 52.7) |  | 1.5 (0.9 to 2.6) | 70.9 (47.3 to 105.4) |  | 7924.0 (5483.3 to 10801.2) | 1.3 (1.0 to 1.7) |
| Bulgaria | 3.8 (2.7 to 5.5) | 66.3 (47.2 to 93.3) |  | 0.6 (0.3 to 1.0) | 7.0 (3.3 to 11.3) |  | NA | 73.9 (49.5 to 105.1) |  | 6.9 (4.6 to 10.0) | 295.7 (224.4 to 380.5) |  | 17009.1 (11766.4 to 23186.7) | 4.7 (3.3 to 6.8) |
| Croatia | 2.8 (1.8 to 4.2) | 27.2 (18.1 to 39.3) |  | 0.3 (0.2 to 0.5) | 1.4 (1.0 to 2.1) |  | NA | 26.3 (17.9 to 37.2) |  | 6.5 (3.7 to 11.6) | 167.2 (112.9 to 226.4) |  | 13457.0 (10103.0 to 17593.5) | 2.1 (1.5 to 3.5) |
| Czechia | 4.6 (3.1 to 6.6) | 50.7 (34.4 to 71.1) |  | 1.1 (0.7 to 1.6) | 4.1 (3.0 to 5.7) |  | NA | 62.6 (42.5 to 88.7) |  | 12.6 (7.3 to 21.3) | 362.0 (250.8 to 486.3) |  | 31905.0 (22302.2 to 43467.9) | 6.1 (4.4 to 9.1) |
| Hungary | 5.3 (3.2 to 7.8) | 70.7 (42.6 to 101.1) |  | 0.5 (0.4 to 0.7) | 3.2 (2.3 to 4.2) |  | NA | 61.8 (39.8 to 89.8) |  | 6.0 (3.3 to 9.6) | 396.5 (261.3 to 540.2) |  | 23831.3 (16488.5 to 32484.2) | 16.9 (8.4 to 25.1) |
| Montenegro | 0.3 (0.2 to 0.6) | 4.7 (2.6 to 8.1) |  | 0.1 (0.0 to 0.1) | 0.4 (0.2 to 0.8) |  | NA | 4.1 (2.3 to 7.1) |  | 0.4 (0.2 to 0.6) | 16.9 (10.8 to 23.9) |  | 1875.9 (1297.5 to 2557.7) | 0.4 (0.3 to 0.5) |
| North Macedonia | 1.4 (0.8 to 2.2) | 20.6 (13.3 to 31.2) |  | 0.1 (0.0 to 0.1) | 0.7 (0.4 to 1.3) |  | NA | 21.6 (14.1 to 32.4) |  | 1.7 (1.1 to 2.5) | 86.1 (61.4 to 119.0) |  | 6177.1 (4272.4 to 8420.9) | 1.3 (1.0 to 1.6) |
| Poland | 16.2 (10.6 to 23.3) | 264.6 (186.9 to 354.1) |  | 2.4 (1.5 to 3.6) | 15.9 (11.3 to 21.7) |  | NA | 229.5 (173.2 to 299.1) |  | 17.6 (10.2 to 31.9) | 1274.3 (929.0 to 1821.3) |  | 24664.5 (18668.2 to 31954.2) | 44.5 (24.4 to 62.1) |
| Romania | 19.1 (13.8 to 26.6) | 293.8 (215.8 to 397.4) |  | 1.5 (0.9 to 2.4) | 13.4 (8.1 to 20.4) |  | NA | 223.0 (153.1 to 322.6) |  | 19.2 (13.4 to 27.4) | 1020.6 (778.7 to 1328.2) |  | 63504.3 (44924.3 to 84780.4) | 15.2 (10.4 to 21.2) |
| Serbia | 1.6 (0.9 to 2.6) | 29.5 (16.4 to 47.0) |  | 0.2 (0.1 to 0.4) | 1.5 (0.8 to 3.0) |  | NA | 26.1 (14.3 to 44.2) |  | 5.2 (3.0 to 8.7) | 188.3 (121.1 to 269.3) |  | 30089.0 (21134.2 to 40602.2) | 8.8 (5.1 to 14.4) |
| Slovakia | 4.2 (2.9 to 6.1) | 52.7 (37.2 to 75.0) |  | 0.7 (0.2 to 2.2) | 3.2 (1.2 to 9.6) |  | NA | 46.2 (31.8 to 66.1) |  | 9.2 (6.1 to 13.7) | 223.0 (164.9 to 295.8) |  | 16726.3 (11616.7 to 22884.3) | 3.0 (2.3 to 4.0) |
| Slovenia | 1.1 (0.6 to 1.7) | 9.2 (4.9 to 14.4) |  | 0.2 (0.1 to 0.3) | 0.7 (0.5 to 0.9) |  | NA | 10.7 (6.6 to 16.0) |  | 2.0 (0.9 to 3.3) | 40.7 (24.3 to 57.7) |  | 5190.9 (3555.0 to 7104.5) | 1.1 (0.8 to 1.5) |
| **Eastern Europe** | 240.7 (193.1 to 291.5) | 3109.1 (2475.4 to 3771.9) |  | 20.5 (13.2 to 26.2) | 172.4 (105.3 to 217.6) |  | NA | 2405.0 (1961.4 to 2896.3) |  | 588.5 (419.6 to 815.5) | 14036.2 (11402.4 to 17041.7) |  | 652508.2 (462159.9 to 885042.7) | 276.9 (170.7 to 360.7) |
| Belarus | 13.9 (8.2 to 23.7) | 162.8 (96.0 to 280.7) |  | 1.0 (0.6 to 1.7) | 6.0 (3.9 to 9.4) |  | NA | 175.2 (114.6 to 259.4) |  | 20.6 (12.0 to 31.9) | 701.5 (471.1 to 980.1) |  | 28431.9 (20139.2 to 38770.2) | 8.9 (5.4 to 13.4) |
| Estonia | 0.4 (0.2 to 0.6) | 4.1 (2.4 to 6.1) |  | 0.1 (0.1 to 0.1) | 0.5 (0.3 to 0.6) |  | NA | 13.7 (8.6 to 20.4) |  | 2.1 (0.9 to 3.7) | 51.5 (32.7 to 70.2) |  | 3518.4 (2491.1 to 4803.3) | 5.4 (1.1 to 9.2) |
| Latvia | 1.9 (1.0 to 2.8) | 20.3 (10.6 to 30.9) |  | 0.1 (0.1 to 0.1) | 0.7 (0.5 to 0.9) |  | NA | 27.2 (16.2 to 41.6) |  | 2.8 (1.1 to 4.8) | 92.8 (55.5 to 130.3) |  | 5012.8 (3426.0 to 6928.7) | 1.1 (0.8 to 1.7) |
| Lithuania | 2.8 (1.6 to4.6) | 35.6 (20.4 to 56.5) |  | 0.2 (0.1 to 0.3) | 1.2 (0.7 to 2.1) |  | NA | 36.5 (21.7 to 58.1) |  | 3.6 (1.8 to 5.5) | 126.6 (80.5 to 182.1) |  | 7574.7 (5272.4 to 10395.1) | 2.5 (1.4 to 4.1) |
| Republic of Moldova | 6.5 (4.2 to 10.2) | 102.3 (66.1 to 155.6) |  | 0.4 (0.1 to 0.7) | 5.1 (1.7 to 8.8) |  | NA | 111.0 (69.6 to 169.7) |  | 5.3 (3.5 to 7.6) | 281.2 (199.8 to 391.1) |  | 8745.4 (6195.3 to 11928.4) | 1.9 (1.4 to 2.6) |
| Russian Federation | 150.5 (121.0 to 183.2) | 1856.1 (1467.0 to 2276.4) |  | 16.9 (10.7 to 21.8) | 128.3 (76.4 to 165.6) |  | NA | 1361.4 (1112.3 to 1665.5) |  | 492.8 (347.5 to 738.8) | 9195.2 (7341.1 to 12164.3) |  | 485390.3 (343821.0 to 657971.6) | 214.3 (128.1 to 286.8) |
| Ukraine | 64.8 (45.3 to 88.7) | 927.9 (662.3 to 1242.5) |  | 1.8 (1.2 to 2.7) | 30.6 (19.6 to 44.4) |  | NA | 679.8 (480.1 to 926.2) |  | 61.3 (38.8 to 81.6) | 3587.6 (2645.9 to 4469.8) |  | 113834.8 (80633.3 to 154334.0) | 42.7 (27.8 to 58.2) |
| **High-income** | 708.7 (570.4 to 882.4) | 6321.2 (5490.7 to 7199.7) |  | 113.9 (91.5 to 137.7) | 434.7 (379.2 to 489.1) |  | NA | 4404.9 (3856.0 to 5015.6) |  | 2180.4 (1667.7 to 2961.1) | 44608.3 (38978.1 to 51158.6) |  | 1469282.8 (1064903.4 to 1966645.6) | 3102.0 (2201.1 to 3724.3) |
| **Australasia** | 23.1 (16.2 to 31.8) | 223.6 (167.9 to 294.9) |  | 4.1 (3.0 to 5.4) | 13.8 (11.6 to 16.1) |  | NA | 131.3 (98.2 to 172.6) |  | 24.1 (16.0 to 33.2) | 1426.0 (1147.1 to 1705.8) |  | 16264.8 (11100.1 to 22665.9) | 34.7 (19.0 to 49.0) |
| Australia | 18.3 (12.0 to 26.5) | 178.9 (129.1 to 245.1) |  | 3.8 (2.8 to 5.0) | 11.8 (9.9 to 13.7) |  | NA | 105.9 (76.3 to 144.1) |  | 17.9 (11.9 to 25.5) | 1162.8 (926.2 to 1388.9) |  | 13846.5 (9290.9 to 19580.6) | 31.8 (16.2 to 45.8) |
| New Zealand | 4.7 (3.3 to 6.4) | 44.7 (32.6 to 57.1) |  | 0.3 (0.2 to 0.4) | 2.0 (1.6 to 2.4) |  | NA | 25.3 (18.9 to 33.8) |  | 6.2 (3.8 to 8.6) | 263.3 (200.0 to 326.0) |  | 2418.4 (1758.5 to 3238.9) | 2.9 (2.3 to 3.8) |
| **High-income Asia Pacific** | 77.3 (61.1 to 95.6) | 792.1 (663.4 to 934.6) |  | 20.6 (16.4 to 25.3) | 55.1 (47.8 to 63.2) |  | NA | 609.4 (497.4 to 752.1) |  | 235.2 (181.5 to 314.0) | 5307.8 (4709.2 to 6057.6) |  | 851002.0 (631692.5 to 1110757.6) | 689.2 (510.5 to 836.4) |
| Brunei Darussalam | 0.6 (0.4 to 0.8) | 10.3 (7.1 to 14.3) |  | 0.0 (0.0 to 0.1) | 0.3 (0.1 to 0.5) |  | NA | 9.7 (6.6 to 14.2) |  | 0.9 (0.7 to 1.2) | 49.2 (36.9 to 64.4) |  | 3326.0 (2398.6 to 4410.7) | 3.7 (2.6 to 5.3) |
| Japan | 45.9 (34.7 to 58.5) | 458.2 (399.9 to 518.0) |  | 15.7 (11.9 to 20.0) | 42.1 (37.1 to 47.8) |  | NA | 343.4 (306.8 to 381.0) |  | 160.3 (109.6 to 236.6) | 3520.1 (3075.9 to 4156.6) |  | 588182.1 (438340.2 to 769281.5) | 541.4 (386.1 to 677.5) |
| Republic of Korea | 24.8 (11.0 to 47.4) | 386.5 (180.7 to 709.9) |  | 4.2 (2.9 to 5.8) | 10.9 (6.9 to 16.3) |  | NA | 253 (114.6 to 536.5) |  | 66.7 (45.7 to 94.9) | 1494.9 (1104.7 to 2121.0) |  | 20312.9 (13375.4 to 28469.6) | 54.0 (29.5 to 93.5) |
| Singapore | 2.0 (1.3 to 2.9) | 21.0 (13.9 to 30.5) |  | 0.7 (0.5 to 0.9) | 1.9 (1.4 to 2.4) |  | NA | 26.9 (17.9 to 38.3) |  | 7.2 (4.2 to 10.6) | 243.6 (145.1 to 339.6) |  | 30524.6 (22020.1 to 40438.3) | 23.8 (12.8 to 35.2) |
| **High-income North America** | 285.9 (218.5 to 372.0) | 2070.4 (1856.2 to 2306.2) |  | 38.9 (28.7 to 52.4) | 160.3 (142.1 to 178.6) |  | NA | 1117.9 (1009.8 to 1241.3) |  | 941.6 (714.3 to 1255.1) | 17796.6 (15789.0 to 19976.4) |  | 63841.5 (45346.9 to 87460.7) | 1334.6 (1008.7 to 1715.7) |
| Canada | 25.5 (18.0 to 35.2) | 179.9 (137.6 to 228.4) |  | 4.9 (3.5 to 6.5) | 16.0 (13.7 to 18.5) |  | NA | 135.8 (100.0 to 181.3) |  | 231.1 (163.0 to 318.0) | 1837.0 (1541.0 to 2173.5) |  | 5658.3 (3853.3 to 8066.1) | 114.2 (85.5 to 155.4) |
| Greenland | 0.0 (0.0 to 0.0) | 0.4 (0.2 to 0.7) |  | 0.0 (0.0 to 0.0) | 0.0 (0.0 to 0.0) |  | NA | 0.2 (0.1 to 0.4) |  | 0.1 (0.0 to 0.1) | 3.1 (1.9 to 5.1) |  | 11.7 (8.0 to 16.7) | 0.1 (0.0 to 0.1) |
| United States of America | 260.4 (195.6 to 345.3) | 1890.1 (1691.2 to 2107.3) |  | 34.0 (24.1 to 47.1) | 144.3 (127.5 to 161.1) |  | NA | 14852.2 (11902.3 to 18307.8) |  | 710.4 (530.0 to 968.6) | 15956.2 (14175.5 to 17914.7) |  | 58170.4 (41508.6 to 79156.4) | 1220.4 (916.9 to 1572.3) |
| **Southern Latin America** | 43.8 (32.3 to 59.5) | 756.5 (586.1 to 968.6) |  | 3.1 (2.2 to 4.2) | 40.5 (32.8 to 52.8) |  | NA | 836.9 (634.0 to 1070.6) |  | 75.5 (53.6 to 137.7) | 4654.1 (3763.9 to 6570.6) |  | 121227.8 (83676.2 to 166614.1) | 392.1 (275.4 to 530.6) |
| Argentina | 29.9 (20.8 to 42.9) | 548.9 (410.7 to 726.6) |  | 1.7 (1.2 to 2.5) | 27.7 (22.5 to 38.5) |  | NA | 575.3 (408.8 to 766.3) |  | 50.6 (35.6 to 95.2) | 3445.9 (2796.6 to 4941.8) |  | 86538.9 (59734.1 to 118937.3) | 232.2 (164.7 to 330.5) |
| Chile | 11.3 (7.6 to 16.2) | 164.8 (113.9 to 229.0) |  | 1.2 (0.8 to 1.9) | 11.2 (7.8 to 16.1) |  | NA | 194.4 (134.8 to 277.8) |  | 20.4 (13.1 to 37.1) | 952.7 (716.7 to 1322.8) |  | 28929.8 (19968.8 to 39761.4) | 137.5 (75.5 to 205.0) |
| Uruguay | 2.5 (1.7 to 3.6) | 42.8 (30.0 to 59.0) |  | 0.1 (0.1 to 0.2) | 1.6 (1.3 to 2.1) |  | NA | 67.1 (44.3 to 96.6) |  | 4.5 (2.9 to 7.0) | 255.3 (183.7 to 341.4) |  | 5752.9 (3972.0 to 7907.1) | 22.3 (14.1 to 32.3) |
| **Western Europe** | 278.6 (216.1 to 353.7) | 2478.7 (2016.5 to 2889.7) |  | 47.2 (37.5 to 57.9) | 165.0 (140.0 to 187.7) |  | NA | 1709.4 (1463.5 to 1986.4) |  | 904.1 (638.1 to 1318.9) | 15423.8 (12549.1 to 17908.8) |  | 416946.7 (287584.8 to 572759.4) | 651.3 (382.3 to 826.5) |
| Andorra | 0.0 (0.0 to 0.1) | 0.3 (0.2 to 0.5) |  | 0.0 (0.0 to 0.0) | 0.0 (0.0 to 0.0) |  | NA | 0.4 (0.3 to 0.7) |  | 0.1 (0.1 to 0.2) | 2.4 (1.5 to 3.4) |  | 48.6 (33.2 to 68.6) | 0.1 (0.0 to 0.1) |
| Austria | 3.7 (2.5 to 5.4) | 35.6 (26.3 to 47.6) |  | 0.7 (0.5 to 1.1) | 3.0 (2.4 to 3.7) |  | NA | 19.7 (13.9 to 26.5) |  | 14.4 (9.0 to 31.0) | 257.3 (183.1 to 385.3) |  | 15394.6 (11159.6 to 20739.3) | 13.2 (5.9 to 20.4) |
| Belgium | 5.6 (3.7 to 8.1) | 54.9 (39.5 to 73.7) |  | 1.2 (0.9 to 1.7) | 4.5 (3.7 to 5.4) |  | NA | 46.6 (31.9 to 65.0) |  | 20.3 (12.9 to 32.4) | 380.6 (305.6 to 492.2) |  | 11474.7 (7810.3 to 16072.3) | 9.8 (6.7 to 13.6) |
| Cyprus | 0.7 (0.4 to 1.0) | 6.6 (4.3 to 9.7) |  | 0.1 (0.1 to 0.1) | 0.3 (0.2 to 0.4) |  | NA | 4.0 (2.6 to 6.2) |  | 2.7 (1.8 to 4.1) | 44.5 (32.0 to 62.5) |  | 1015.3 (682.5 to 1425.4) | 0.7 (0.5 to 1.0) |
| Denmark | 1.5 (1.0 to 2.2) | 18.2 (12.5 to 25.3) |  | 0.6 (0.4 to 0.8) | 2.1 (1.6 to 2.6) |  | NA | 23.9 (15.8 to 33.7) |  | 8.1 (4.9 to 12.5) | 213.4 (155.7 to 272.3) |  | 5534.8 (3757.4 to 7663.1) | 2.6 (2.0 to 3.9) |
| Finland | 2.0 (1.3 to 3.0) | 18.4 (12.4 to 25.9) |  | 0.5 (0.3 to 0.7) | 1.8 (1.4 to 2.2) |  | NA | 24.4 (17.0 to 33.1) |  | 7.9 (4.3 to 12.1) | 185.4 (131.1 to 230.8) |  | 5078.2 (3397.5 to 7049.6) | 2.1 (1.7 to 3.1) |
| France | 48.5 (33.9 to 67.7) | 472.2 (359.0 to 602.6) |  | 8.2 (6.0 to 11.1) | 29.0 (24.6 to 33.7) |  | NA | 355.9 (262.4 to 474.3) |  | 138.8 (89.4 to 207.2) | 2375.4 (1906.1 to 3148.8) |  | 65931.4 (45067.5 to 93180.3) | 120.9 (60.3 to 171.3) |
| Germany | 39.6 (27.0 to 57.3) | 332.9 (246.3 to 435.1) |  | 11.6 (8.5 to 15.2) | 30.5 (25.2 to 36.2) |  | NA | 258.6 (188.3 to 343.4) |  | 293.8 (186.0 to 496.8) | 2579.9 (2029.7 to 3330.2) |  | 81374.9 (55243.9 to 113819.8) | 90.5 (52.8 to 126.8) |
| Greece | 4.8 (3.3 to 6.9) | 49.3 (36.4 to 65.8) |  | 1.4 (0.8 to 2.3) | 5.8 (3.8 to 8.7) |  | NA | 47.3 (33.3 to 66.6) |  | 14.0 (9.1 to 23.6) | 248.9 (195.2 to 354.9) |  | 8165.3 (5581.6 to 11540.2) | 13.7 (5.6 to 20.6) |
| Iceland | 0.2 (0.2 to 0.4) | 2.5 (1.5 to 4.0) |  | 0.0 (0.0 to 0.1) | 0.2 (0.1 to 0.2) |  | NA | 1.5 (0.8 to 2.4) |  | 0.7 (0.4 to 1.0) | 13.3 (8.3 to 20.2) |  | 337.6 (228.7 to 470.5) | 0.3 (0.2 to 0.5) |
| Ireland | 3.4 (2.0 to 5.3) | 12.7 (7.9 to 19.0) |  | 0.8 (0.5 to 1.3) | 2.7 (1.8 to 3.9) |  | NA | 12.5 (8.1 to 18.8) |  | 11.4 (6.7 to 17.6) | 179.3 (129.0 to 237.6) |  | 5733.1 (3919.2 to 8102.6) | 3.2 (2.3 to 4.9) |
| Israel | 6.5 (4.4 to 9.4) | 70.4 (52.0 to 95.2) |  | 1.7 (1.0 to 2.9) | 8.2 (5.9 to 13.9) |  | NA | 88.5 (64.4 to 119.7) |  | 23.9 (16.1 to 35.3) | 605.8 (494.6 to 739.4) |  | 17119.4 (11697.5 to 24208.3) | 16.8 (11.9 to 23.4) |
| Italy | 48.8 (36.1 to 66.6) | 342.2 (268.0 to 405.1) |  | 4.0 (2.8 to 5.4) | 15.8 (13.0 to 18.4) |  | NA | 168.0 (139.3 to 197.0) |  | 112.7 (68.4 to 171.3) | 1957.2 (1395.6 to 2312.5) |  | 56974.3 (41086.5 to 76422.9) | 95.3 (49.4 to 124.8) |
| Luxembourg | 0.2 (0.1 to 0.2) | 1.9 (1.1 to 3.0) |  | 0.1 (0.0 to 0.1) | 0.2 (0.2 to 0.4) |  | NA | 2.4 (1.4 to 4.1) |  | 1.0 (0.6 to 1.7) | 19.9 (12.3 to 30.9) |  | 595.4 (395.5 to 841.0) | 0.5 (0.3 to 0.8) |
| Malta | 0.2 (0.2 to 0.3) | 2.5 (1.6 to 3.8) |  | 0.0 (0.0 to 0.1) | 0.2 (0.1 to 0.2) |  | NA | 2.4 (1.5 to 3.8) |  | 0.7 (0.4 to 1.0) | 21.1 (14.2 to 30.5) |  | 344.9 (235.2 to 484.5) | 0.9 (0.4 to 1.4) |
| Monaco | 0.0 (0.0 to 0.1) | 0.3 (0.1 to 0.5) |  | 0.0 (0.0 to 0.0) | 0.0 (0.0 to 0.0) |  | NA | 0.6 (0.3 to 1.0) |  | 0.1 (0.0 to 0.1) | 0.9 (0.6 to 1.4) |  | 29.3 (20.0 to 41.4) | 0.0 (0.0 to 0.0) |
| Netherlands | 9.1 (5.9 to 13.4) | 84.0 (59.2 to 111.7) |  | 2.1 (1.5 to 2.9) | 6.6 (5.3 to 8.0) |  | NA | 77.4 (54.0 to 106.7) |  | 35.7 (22.0 to 55.0) | 689.4 (529.0 to 875.2) |  | 15907.8 (10871.0 to 22490.4) | 47.1 (22.8 to 66.3) |
| Norway | 3.8 (2.5 to 5.2) | 29.5 (21.0 to 36.8) |  | 0.6 (0.4 to 0.8) | 1.7 (1.4 to 2.0) |  | NA | 19.8 (15.1 to 23.6) |  | 12.3 (7.1 to 17.8) | 147.8 (105.7 to 179.6) |  | 7541.6 (5208.4 to 10258.5) | 3.4 (2.5 to 4.2) |
| Portugal | 6.4 (4.4 to 9.4) | 60.6 (44.3 to 84.5) |  | 0.7 (0.4 to 1.2) | 3.5 (2.4 to 5.1) |  | NA | 60.4 (41.3 to 86.4) |  | 11.5 (6.8 to 18.4) | 272.9 (200.5 to 362.6) |  | 6474.8 (4477.7 to 8904.9) | 28.4 (10.0 to 48.6) |
| San Marino | 0.0 (0.0 to 0.0) | 0.2 (0.1 to 0.3) |  | 0.0 (0.0 to 0.0) | 0.0 (0.0 to 0.0) |  | NA | 0.7 (0.4 to 1.1) |  | 0.1 (0.1 to 0.2) | 2.2 (1.5 to 3.0) |  | 29.4 (20.1 to 41.6) | 0.0 (0.0 to 0.1) |
| Spain | 28.8 (19.6 to 41.8) | 254.8 (191.1 to 333.8) |  | 4.2 (3.0 to 5.9) | 15.5 (12.8 to 19.1) |  | NA | 196.2 (144.3 to 260.2) |  | 61.2 (39.7 to 88.6) | 1552.5 (1175.0 to 1908.4) |  | 36313.3 (24824.0 to 51318.5) | 61.6 (39.5 to 84.4) |
| Sweden | 6.3 (4.3 to 8.6) | 72.9 (51.5 to 96.1) |  | 1.3 (1.0 to 1.8) | 4.4 (3.4 to 5.4) |  | NA | 35.6 (24.5 to 47.2) |  | 15.0 (9.5 to 23.3) | 413.0 (286.6 to 512.0) |  | 18597.7 (12732.3 to 25783.8) | 10.3 (6.0 to 14.9) |
| Switzerland | 3.7 (2.4 to5.3) | 34.9 (24.5 to 47.6) |  | 1.0 (0.7 to 1.3) | 3.2 (2.6 to 3.8) |  | NA | 27.1 (19.1 to 37) |  | 22.5 (14.2 to 33.4) | 289.5 (218.3 to 367.0) |  | 8062.5 (5392.7 to 11243.5) | 4.6 (3.3 to 6.5) |
| United Kingdom | 54.5 (40.3 to 71.4) | 501.1 (411.8 to 579.6) |  | 6.4 (4.7 to 8.5) | 25.4 (21.6 to 29.3) |  | NA | 234.1 (201.2 to 262.2) |  | 94.1 (67.2 to 127.1) | 2957.8 (2362.6 to 3440.2) |  | 48504.2 (34127.5 to 66389.4) | 124.6 (73.0 to 156.3) |
| **Latin America and Caribbean** | 863.5 (679.3 to 1102.3) | 15335.8 (12123.6 to 19377.7) |  | 44.5 (32.7 to 61.2) | 793.1 (585.4 to 1058.8) |  | NA | 12993.4 (10263.8 to 16305.2) |  | 1532.2 (1095.7 to 2076.9) | 69010.8 (52900.4 to 89828.2) |  | 830984.0 (582041.4 to 1129962.4) | 6049.1 (4072.0 to 8180.7) |
| **Andean Latin America** | 57.9 (36.9 to 88.4) | 1211.1 (784.5 to 1853.4) |  | 4.8 (2.7 to 8.2) | 119.1 (67.4 to 202.2) |  | NA | 1638 (970.4 to 2755.5) |  | 259.8 (140.5 to 450.6) | 7056.0 (4991.4 to 9724.5) |  | 71677.2 (51656.2 to 95997.0) | 465.2 (289.5 to 669.5) |
| Bolivia (Plurinational State of) | 17.6 (8.6 to 31.3) | 439.3 (216.6 to 775.4) |  | 1.5 (0.5 to 3.2) | 51.9 (18.0 to 115.4) |  | NA | 618.2 (272.9 to 1137.5) |  | 30.4 (16.4 to 46.3) | 2624.4 (1480.6 to 3622.3) |  | 17558.3 (11909.0 to 24412.4) | 123.3 (71.0 to 186.7) |
| Ecuador | 16.4 (10.3 to 25.5) | 327.3 (206.0 to 493.8) |  | 2.2 (0.9 to 5.1) | 47.8 (20.6 to 105.0) |  | NA | 479.5 (274.4 to 779.0) |  | 23.0 (13.7 to 35.4) | 1519.5 (982.4 to 2175.4) |  | 17999.0 (14245.9 to 22173.1) | 290.5 (152.2 to 458.9) |
| Peru | 23.9 (11.7 to 43.4) | 444.5 (224.3 to 795.2) |  | 1.1 (0.4 to 2.5) | 19.3 (7.0 to 43.1) |  | NA | 540.4 (208.6 to 1233.5) |  | 206.4 (91.1 to 389.8) | 2912.1 (1535.8 to 4577.7) |  | 36119.9 (24531.6 to 50243.4) | 51.4 (28.4 to 85.9) |
| **Caribbean** | 73.0 (46.5 to 105.3) | 1892.1 (1114.0 to 2882.5) |  | 2.3 (1.4 to 4.0) | 68.1 (31.9 to 146.4) |  | NA | 2532.0 (1127.5 to 4393.7) |  | 128.0 (79.6 to 221.0) | 11034.7 (6600.8 to 18648.7) |  | 55532.5 (38152.5 to 77297.7) | 449.0 (280.9 to 732.7) |
| Antigua and Barbuda | 0.1 (0.1 to 0.2) | 1.9 (1.1 to 3.3) |  | 0.0 (0.0 to 0.0) | 0.0 (0.0 to 0.1) |  | NA | 2.3 (1.3 to 3.7) |  | 0.1 (0.1 to 0.2) | 11.4 (6.9 to 17.8) |  | 71.7 (49.3 to 99.6) | 0.3 (0.2 to 0.5) |
| Bahamas | 0.3 (0.2 to 0.4) | 5.8 (3.8 to 8.7) |  | 0.0 (0.0 to 0.0) | 0.2 (0.1 to 0.2) |  | NA | 5.9 (3.6 to 8.9) |  | 0.4 (0.2 to 0.6) | 30.8 (21.0 to 44.0) |  | 306.1 (210.6 to 425.4) | 3.0 (1.8 to 4.6) |
| Barbados | 0.2 (0.1 to 0.3) | 3.9 (2.5 to 5.9) |  | 0.0 (0.0 to 0.0) | 0.1 (0.1 to 0.1) |  | NA | 4.2 (2.7 to 6.4) |  | 0.3 (0.2 to 0.5) | 0.3 (0.1 to 1.0) |  | 205.0 (141.0 to 285.4) | 1.1 (0.7 to 1.7) |
| Belize | 0.5 (0.3 to 0.7) | 11.1 (7.6 to 15.7) |  | 0.0 (0.0 to 0.0) | 0.4 (0.2 to 0.6) |  | NA | 6.4 (4.2 to 9.3) |  | 0.5 (0.3 to 0.7) | 46.7 (35.2 to 62.3) |  | 527.6 (362.8 to 734.7) | 2.9 (1.8 to 4.3) |
| Bermuda | 0.1 (0.0 to 0.1) | 0.9 (0.6 to 1.3) |  | 0.0 (0.0 to 0.0) | 0.0 (0.0 to 0.1) |  | NA | 1.1 (0.7 to 1.7) |  | 0.1 (0.1 to 0.1) | 2.9 (2.1 to 4.1) |  | 36.8 (25.3 to 51.2) | 0.5 (0.3 to 0.7) |
| Cuba | 8.4 (5.8 to 11.6) | 128.7 (89.1 to 177.8) |  | 0.9 (0.4 to 1.4) | 9.2 (4.3 to 14.9) |  | NA | 174.7 (124.0 to 234.9) |  | 13.7 (8.0 to 23.3) | 631.2 (458.5 to 815.6) |  | 7889.4 (5425.8 to 10973.4) | 14.8 (9.9 to 21) |
| Dominica | 0.1 (0.1 to 0.2) | 2.2 (1.3 to 3.6) |  | 0.0 (0.0 to 0.0) | 0.1 (0.0 to 0.1) |  | NA | 3.8 (2.2 to 6.1) |  | 0.1 (0.1 to 0.2) | 18.8 (11.7 to 28.9) |  | 59.6 (41.0 to 82.8) | 0.9 (0.6 to 1.5) |
| Dominican Republic | 26.1 (13.5 to 44.7) | 577.7 (309.9 to 989.2) |  | 0.3 (0.1 to 0.6) | 6.6 (2.9 to 15.9) |  | NA | 309.5 (154.4 to 616.4) |  | 39.7 (19.6 to 68.4) | 2255.8 (1425.8 to 3493.1) |  | 15398.5 (10578.7 to 21436.1) | 177.7 (94.5 to 300.5) |
| Grenada | 0.1 (0.0 to 0.1) | 1.7 (1.0 to 2.5) |  | 0.0 (0.0 to 0.0) | 0.1 (0.0 to 0.1) |  | NA | 4.0 (2.4 to 6.3) |  | 0.1 (0.1 to 0.2) | 9.1 (6.0 to 13.4) |  | 98.6 (67.9 to 137.1) | 1.4 (0.8 to 2.2) |
| Guyana | 0.9 (0.5 to 1.8) | 24.6 (12.1 to 47.7) |  | 0.0 (0.0 to 0.0) | 0.6 (0.4 to 1.0) |  | NA | 14.6 (8.9 to 23.2) |  | 1.1 (0.7 to 1.9) | 95.8 (62.0 to 144.5) |  | 998.2 (685.7 to 1388.0) | 5.3 (3.0 to 8.1) |
| Haiti | 29.3 (10.0 to 54.5) | 979.0 (333.4 to 1822.0) |  | 0.8 (0.2 to 2.3) | 44.4 (12.9 to 118.3) |  | NA | 1820.6 (445.3 to 3625.1) |  | 55.8 (21.0 to 131.6) | 6996.1 (3378.7 to 13781.7) |  | 21433.5 (14716.1 to 29848.3) | 197.4 (83.7 to 494.6) |
| Jamaica | 1.1 (0.7 to 1.7) | 23.6 (14.7 to 35.7) |  | 0.1 (0.0 to 0.1) | 1.7 (1.1 to 2.4) |  | NA | 36.5 (23.0 to 55.6) |  | 1.8 (1.0 to 4.9) | 181.0 (116.1 to 310.0) |  | 2595.5 (1784.3 to 3611.5) | 8.7 (5.1 to 14.9) |
| Puerto Rico | 0.8 (0.6 to 1.2) | 11.2 (7.9 to 15.8) |  | 0.1 (0.1 to 0.2) | 1.0 (0.7 to 1.2) |  | NA | 7.4 (4.9 to 10.7) |  | 3.1 (2.0 to 4.9) | 105.7 (77.6 to 138.5) |  | 1847.7 (1270.1 to 2570.4) | 4.9 (3.2 to 7.8) |
| Saint Kitts and Nevis | 0.1 (0.0 to 0.1) | 1.2 (0.4 to 1.9) |  | 0.0 (0.0 to 0.0) | 0.0 (0.0 to 0.0) |  | NA | 1.2 (0.3 to 2.0) |  | 0.1 (0.0 to 0.2) | 5.8 (2.1 to 9.5) |  | 50.2 (34.5 to 69.9) | 0.2 (0.1 to 0.3) |
| Saint Lucia | 0.1 (0.1 to 0.2) | 2.1 (1.3 to 3.2) |  | 0.0 (0.0 to 0.0) | 0.1 (0.1 to 0.2) |  | NA | 4.4 (2.7 to 7.1) |  | 0.2 (0.1 to 0.3) | 19.2 (12.1 to 29.2) |  | 130.2 (89.5 to 180.9) | 1.3 (0.7 to 2.1) |
| Saint Vincent and the Grenadines | 0.1 (0.0 to 0.1) | 1.7 (1.1 to 2.5) |  | 0.0 (0.0 to 0.0) | 0.1 (0.0 to 0.1) |  | NA | 3.9 (2.4 to 6.1) |  | 0.1 (0.1 to 0.2) | 12.5 (8.2 to 18.6) |  | 108.9 (74.9 to 151.5) | 1.4 (0.8 to 2.2) |
| Suriname | 0.6 (0.4 to 1.0) | 15.4 (9.1 to 25.1) |  | 0.0 (0.0 to 0.0) | 0.5 (0.2 to 1.0) |  | NA | 16.3 (9.5 to 25.2) |  | 1.0 (0.6 to 1.5) | 78.2 (54.5 to 110.7) |  | 642.1 (441.0 to 893.0) | 5.4 (3.3 to 8.6) |
| Trinidad and Tobago | 1.6 (1.0 to 2.3) | 33.8 (22.2 to 48.5) |  | 0.0 (0.0 to 0.0) | 0.7 (0.5 to 1.0) |  | NA | 28.0 (17.2 to 42.4) |  | 5.3 (3.1 to 9.1) | 133.8 (89.7 to 193.1) |  | 1161.8 (799.7 to 1614.9) | 5.8 (3.3 to 9.3) |
| United States Virgin Islands | 0.1 (0.0 to 0.1) | 1.5 (0.7 to 2.6) |  | 0.0 (0.0 to 0.0) | 0.0 (0.0 to 0.0) |  | NA | 1.3 (0.6 to 2.6) |  | 0.1 (0.0 to 0.1) | 4.9 (2.9 to 7.6) |  | 90.0 (61.9 to 125.3) | 0.5 (0.3 to 1.0) |
| **Central Latin America** | 359.2 (265.4 to 485.5) | 6249.4 (4731.7 to 8160.3) |  | 29.0 (20.0 to 41.7) | 472.8 (334.3 to 630.1) |  | NA | 5229.5 (3907.0 to 6836.1) |  | 786.2 (546.1 to 1088.4) | 27534.7 (20000.7 to 36572.0) |  | 627161.7 (443379.6 to 851183.7) | 3634.7 (2226.7 to 5112.9) |
| Colombia | 82.0 (53.5 to 118.5) | 1357.7 (892.7 to 1959.6) |  | 4.9 (2.7 to 8.2) | 67.1 (38.8 to 108.8) |  | NA | 1263.4 (828.1 to 1825.9) |  | 181.0 (107.8 to 278.8) | 5089.9 (3592.8 to 7135.0) |  | 105379.6 (71962.2 to 146825.1) | 824.6 (440.7 to 1318.4) |
| Costa Rica | 2.3 (1.5 to 3.5) | 36.2 (24.5 to 52.0) |  | 0.4 (0.2 to 0.9) | 3.8 (2.0 to 8.5) |  | NA | 59.0 (39.9 to 88.6) |  | 12.0 (7.2 to 25.6) | 273.9 (191.9 to 465.8) |  | 9350.9 (6388.1 to 13018.8) | 27.3 (16.0 to 47.9) |
| El Salvador | 3.5 (1.5 to 6.2) | 66.5 (30.3 to 117.9) |  | 0.4 (0.1 to 1.0) | 7.7 (1.9 to 19.0) |  | NA | 63.2 (17.0 to 132.2) |  | 11.8 (5.7 to 19.9) | 376.3 (187.9 to 619.3) |  | 15704.2 (10725.6 to 21866.9) | 20.7 (9.3 to 38.1) |
| Guatemala | 17.7 (11.6 to 26.2) | 425.5 (279.3 to 622.7) |  | 1.8 (0.9 to 3.1) | 60.4 (31.6 to 99.2) |  | NA | 440.9 (285.7 to 642.6) |  | 34.0 (21.1 to 63.0) | 2030.2 (1355.1 to 2995.0) |  | 54927.7 (37509.8 to 76525.9) | 245.3 (124.4 to 392.2) |
| Honduras | 13.1 (5.8 to 25.6) | 302.2 (137.8 to 590.8) |  | 0.1 (0.1 to 0.3) | 4.3 (1.7 to 9.3) |  | NA | 134.3 (40.4 to 359.0) |  | 97.9 (36.0 to 181.3) | 3554.2 (1599.0 to 6249.6) |  | 31083 (21222.0 to 43311.6) | 114.4 (67.4 to 192.4) |
| Mexico | 186.8 (137.7 to 250.1) | 3063.0 (2369.4 to 3938.1) |  | 17.7 (11.9 to 26.0) | 267.9 (184.5 to 354.3) |  | NA | 2295.8 (1805.1 to 2864.9) |  | 351.8 (253.3 to 475.0) | 12391.8 (9216.2 to 16034.1) |  | 318781.0 (231074.7 to 427835.4) | 1911.6 (1158.6 to 2677.5) |
| Nicaragua | 12.7 (7.9 to 19.4) | 234.7 (148.6 to 359.2) |  | 0.5 (0.3 to 0.9) | 9.6 (5.6 to 15.2) |  | NA | 209.5 (127.9 to 336.9) |  | 16.3 (9.6 to 26.6) | 749.2 (514.5 to 1033.2) |  | 18152.5 (12400.8 to 25279.3) | 88.8 (49.2 to 135.4) |
| Panama | 6.2 (4.2 to 8.9) | 108.7 (75.6 to 150.9) |  | 1.3 (0.3 to 2.3) | 19.3 (4.1 to 33.7) |  | NA | 129.5 (84.2 to 194.6) |  | 13.5 (8.6 to 22.5) | 386.6 (270.9 to 583.8) |  | 10629.6 (7261.2 to 14800.2) | 27.7 (15.6 to 59.1) |
| Venezuela (Bolivarian Republic of) | 34.9 (22.9 to 50.9) | 654.9 (444.2 to 941.1) |  | 1.8 (1.0 to 3.3) | 32.7 (19.0 to 56.2) |  | NA | 633.8 (402.7 to 941.0) |  | 67.9 (41.2 to 122.5) | 2682.7 (1911.9 to 3869.4) |  | 63153.2 (43118.0 to 87997.5) | 374.2 (217.1 to 572.5) |
| **Tropical Latin America** | 373.4 (297.7 to 463.6) | 5983.2 (4729.1 to 7464.1) |  | 8.5 (6.2 to 11.9) | 133.2 (99.5 to 183.3) |  | NA | 3593.8 (2784.7 to 4536.1) |  | 358.1 (269.6 to 468.8) | 23385.4 (18427.7 to 29425.6) |  | 76612.7 (52942.7 to 105116.2) | 1500.2 (1032.5 to 1992.9) |
| Brazil | 370.7 (295.0 to 460.1) | 5929.8 (4693.6 to 7395.5) |  | 8.3 (6.1 to 11.7) | 128.6 (96.3 to 177.0) |  | NA | 3548.2 (2761.4 to 4466.1) |  | 350.0 (263.0 to 459.0) | 22893.4 (18054.9 to 28844.7) |  | 72968.0 (50570.7 to 99905.3) | 1485.1 (1017.0 to 1975.3) |
| Paraguay | 2.6 (1.2 to 5.0) | 53.4 (25.3 to 97.4) |  | 0.2 (0.1 to 0.4) | 4.6 (1.5 to 9.6) |  | NA | 45.7 (15.2 to 100.0) |  | 8.1 (4.3 to 13.6) | 492.1 (273.3 to 865.2) |  | 3644.7 (2445.0 to 5096.8) | 15.1 (7.7 to 28.2) |
| **North Africa and Middle East** | 1008.2 (718.3to1338.9) | 11283.4 (8133.3to15055.4) |  | 29.1 (13.3 to 47.3) | 542.6 (242.0 to 911.7) |  | NA | 9636.4 (6872.7 to 13006.0) |  | 2086.0 (1496.4 to 2716.8) | 68811.6 (54511.3 to 86253.9) |  | 1041664.1 (730141.5 to 1410119.1) | 1156.2 (846.0 to 1517.1) |
| **North Africa and Middle East** | 1008.2 (718.3 to 1338.9) | 11283.4 (8133.3 to 15055.4) |  | 29.1 (13.3 to 47.3) | 542.6 (242.0 to 911.7) |  | NA | 9636.4 (6872.7 to 13006.0) |  | 2086.0 (1496.4 to 2716.8) | 68811.6 (54511.3 to 86253.9) |  | 1041664.1 (730141.5 to 1410119.1 | 1156.2 (846.0 to 1517.1) |
| Afghanistan | 71.4 (37.6 to 129.4) | 1519.8 (807.0 to 2715.6) |  | 1.8 (0.6 to 4.7) | 95.8 (30.7 to 250.9) |  | NA | 1511.8 (671.3 to 2908.2) |  | 280.9 (148.2 to 469.9) | 14961.1 (8422.2 to 24449.5) |  | 114595.9 (79465.7 to 155521.5) | 92.0 (54.6 to 150.2) |
| Algeria | 48.2 (25.7 to 90.0) | 411.5 (242.8 to 694.9) |  | 3.5 (1.2 to 9.6) | 51.7 (18.1 to 136.3) |  | NA | 1417.5 (839.3 to 2362.8) |  | 106.0 (66.5 to 166.3) | 3133.2 (2156.4 to 4261.1) |  | 74159.1 (51371.2 to 100816.9) | 61.9 (44.9 to 88.3) |
| Bahrain | 1.1 (0.6 to 1.7) | 7.4 (4.9 to 10.9) |  | 0.0 (0.0 to 0.1) | 0.3 (0.2 to 0.5) |  | NA | 10.0 (6.3 to 15.3) |  | 2.1 (1.3 to 3.1) | 37.5 (27.8 to 51.4) |  | 1228.4 (851.1 to 1670.7) | 1.1 (0.7 to 1.8) |
| Egypt | 137.1 (60.4 to 278.8) | 1679.0 (834.7 to 3203.2) |  | 1.3 (0.7 to 2.5) | 31.8 (16.2 to 59.3) |  | NA | 1073.7 (406.1 to 2543.8) |  | 351.8 (144.7 to 626.4) | 13006.8 (8475.5 to 19396.9) |  | 187591.9 (129970.7 to 254959.5) | 115.2 (77.6 to 162.6) |
| Iran (Islamic Republic of) | 163.2 (98.6 to 242.9) | 1310.6 (828.2 to 1834) |  | 8.0 (1.9 to 15.4) | 89.6 (21.0 to 174.0) |  | NA | 716.1 (420.6 to 1224.1) |  | 464.1 (239.1 to 719.6) | 7312.7 (3930.2 to 10612.1) |  | 120123.7 (83224.8 to 161851.0) | 88.3 (64.3 to 116.8) |
| Iraq | 83.3 (42.3 to 147.0) | 844.0 (464.5 to 1411.2) |  | 1.6 (0.7 to 3.6) | 29.7 (13.1 to 66.9) |  | NA | 510.6 (275.9 to 911.9) |  | 100.9 (58.1 to 173.3) | 3572.7 (2304.0 to 5545.3) |  | 80656.5 (55843.5 to 109681.3) | 53.1 (37.2 to 74.2) |
| Jordan | 22.3 (13.5 to 34.3) | 177.8 (115.3 to 262.6) |  | 0.3 (0.2 to 0.5) | 4.0 (2.9 to 5.6) |  | NA | 107.0 (70.1 to 162.4) |  | 30.1 (18.6 to 46.4) | 737.0 (499.0 to 1089.0) |  | 17237.8 (12391.4 to 23112.4) | 15.6 (9.5 to 23.0) |
| Kuwait | 15.1 (10.6 to 20.4) | 106.5 (77.0 to 143.5) |  | 0.4 (0.2 to 0.5) | 2.3 (1.8 to 2.9) |  | NA | 72.3 (48.8 to 105.0) |  | 15.2 (9.4 to 23.7) | 190.0 (144.1 to 250.9) |  | 5170.1 (3580.2 to 7031.3) | 3.8 (3.0 to 4.9) |
| Lebanon | 7.3 (3.2 to 14.9) | 58.7 (27.1 to 108.8) |  | 1.0 (0.2 to 2.6) | 6.2 (1.6 to 17.4) |  | NA | 68.5 (27.9 to 123.4) |  | 18.3 (8.0 to 32.8) | 367.8 (157.6 to 688.0) |  | 8763.6 (6074.2 to 11913.0) | 6.7 (4.1 to 10.1) |
| Libya | 5.7 (2.9 to 9.6) | 70.1 (34.8 to 122.0) |  | 0.4 (0.1 to 1.3) | 7.8 (2.2 to 21.3) |  | NA | 75.4 (38.6 to 131.6) |  | 6.6 (3.5 to 11.1) | 385.9 (228.1 to 735.9) |  | 7354.9 (5094.6 to 9994.3) | 5.9 (4.0 to 8.5) |
| Morocco | 32.8 (12.4 to 76.4) | 321.7 (141.7 to 678.0) |  | 1.6 (0.4 to 5.5) | 40.7 (10.0 to 139.4) |  | NA | 488.6 (214.4 to 1021.1) |  | 58.7 (28.9 to 111.2) | 2077.3 (1080.1 to 3483.6) |  | 52862.6 (36607.6 to 71853.9) | 40.1 (23.8 to 63.7) |
| Oman | 8.8 (5.0 to 14.8) | 85.7 (52.6 to 134.5) |  | 0.2 (0.1 to 0.4) | 2.0 (1.4 to 3.4) |  | NA | 88.2 (53.6 to 140.3) |  | 27.0 (16.7 to 42.9) | 383.5 (274.1 to 527.0) |  | 10788.8 (7465.4 to 14670.6) | 6.9 (5.2 to 9.1) |
| Palestine | 8.8 (5.0 to 14.8) | 85.7 (52.6 to 134.5) |  | 0.3 (0.1 to 0.8) | 5.5 (2.4 to 13.6) |  | NA | 88.2 (53.6 to 140.3) |  | 14.6 (8.9 to 22.3) | 419.1 (284.8 to 598.1) |  | 10788.8 (7465.4 to 14670.6) | 6.9 (5.2 to 9.1) |
| Qatar | 1.4 (0.8 to 2.5) | 9.6 (5.8 to 15.6) |  | 0.1 (0.1 to 0.1) | 0.5 (0.4 to 0.8) |  | NA | 22.4 (12.6 to 41.3) |  | 6.4 (3.6 to 10.2) | 74.9 (49.8 to 108.9) |  | 3294.1 (2308.1 to 4448.5) | 1.6 (1.2 to 2.2) |
| Saudi Arabia | 23.3 (9.0 to 47.7) | 190.5 (79.2 to 376.7) |  | 0.9 (0.5 to 1.9) | 10.0 (5.1 to 20.1) |  | NA | 288.8 (115.7 to 637.6) |  | 50.3 (26.8 to 86.0) | 946.6 (612.9 to 1444.0) |  | 39451.2 (27287.6 to 53638.1) | 23.3 (15.3 to 32.4) |
| Sudan | 122.8 (55.7 to 229.1) | 2018.9 (880.4 to 3883.9) |  | 2.1 (0.6 to 5.3) | 75.3 (20.5 to 190.4) |  | NA | 1392.6 (550.5 to 2719.3) |  | 199.9 (91.1 to 355.0) | 8508.5 (4551.8 to 14409.8) |  | 95275.8 (66011.2 to 129509.0) | 82.9 (48.5 to 137.4) |
| Syrian Arab Republic | 9.9 (5.8 to 15.4) | 101.8 (64.4 to 149.6) |  | 0.4 (0.2 to 0.8) | 5.9 (3.7 to 12.3) |  | NA | 94.0 (57.3 to 145.3) |  | 24.4 (14.8 to 37.4) | 713.9 (482.3 to 989.7) |  | 19654.8 (13601.0 to 26733.6) | 20.7 (13.7 to 32.3) |
| Tunisia | 10.3 (5.0 to 20.3) | 87.7 (45.3 to 151.2) |  | 0.7 (0.3 to 2.0) | 7.5 (3.0 to 20.7) |  | NA | 47.1 (27.2 to 80.3) |  | 26.6 (15.0 to 45.2) | 511.4 (333.3 to 729.2) |  | 15036.5 (10428.0 to 20423.5) | 11.9 (8.6 to 16.7) |
| Turkey | 179.8 (98.1 to 296) | 1440.9 (875.4 to 2139.5) |  | 3.3 (1.6 to 8.0) | 33.8 (17.2 to 77.7) |  | NA | 1078.8 (682.1 to 1613.7) |  | 171.7 (112.0 to 269.2) | 5920.1 (3861.1 to 8428.9) |  | 97764.5 (67078.2 to 132216.7) | 463.7 (265.5 to 703.8) |
| United Arab Emirates | 7.4 (3.5 to 14.6) | 81.5 (41.8 to 147.6) |  | 0.1 (0.0 to 0.3) | 2.2 (0.9 to 6.4) |  | NA | 39.3 (19.8 to 77.1) |  | 11.0 (4.3 to 20.9) | 280.7 (141.5 to 459.8) |  | 5804.3 (4023.1 to 7907.8) | 5.1 (3.3 to 7.8) |
| Yemen | 48.9 (22.3 to 96.2) | 697.4 (337.8 to 1263.3) |  | 1.0 (0.3 to 2.4) | 39.4 (12.4 to 96.7) |  | NA | 479.5 (267.1 to 813.5) |  | 117.2 (64.8 to 192.9) | 5201.2 (3027.3 to 8081.6) |  | 77001.1 (53293.3 to 104740.8) | 48.7 (30.4 to 77.4) |
| **South Asia** | 534.3 (352.2 to 730.8) | 12073.2 (8161.7 to 16098.1) |  | 59.8 (34.8 to 100.2) | 2017.6 (1187.6 to 3397.7) |  | NA | 29138.0 (22019.4 to 37569.9) |  | 2735.7 (1899.8 to 3537.4) | 255026.6 (186541.4 to 331620.9) |  | 782620.7 (528493.6 to 1088888.0) | 3354.4 (2171.4 to 5388.5) |
| **South Asia** | 534.3 (352.2 to 730.8) | 12073.2 (8161.7 to 16098.1) |  | 59.8 (34.8 to 100.2) | 2017.6 (1187.6 to 3397.7) |  | NA | 29138.0 (22019.4 to 37569.9) |  | 2735.7 (1899.8 to 3537.4) | 255026.6 (186541.4 to 331620.9) |  | 782620.7 (528493.6 to 1088888.0) | 3354.4 (2171.4 to 5388.5) |
| Bangladesh | 34.2 (19.0 to 57.8) | 858.6 (491.8 to 1444.1) |  | 3.1 (1.6 to 5.5) | 112.8 (57.2 to 200.7) |  | NA | 1832.3 (975.9 to 3342.1) |  | 412.2 (246.2 to 634.7) | 27170.9 (18951.7 to 38737.7) |  | 50046 (33568.5 to 71492.9) | 242.1 (157.0 to 370.9) |
| Bhutan | 0.4 (0.1 to 0.7) | 8.5 (3.6 to 17.0) |  | 0.0 (0.0 to 0.1) | 0.9 (0.4 to 1.6) |  | NA | 19.7 (9.1 to 34.5) |  | 1.0 (0.5 to 1.8) | 112.4 (62.7 to 186.1) |  | 238.1 (159.7 to 339.8) | 2.1 (1.1 to 3.8) |
| India | 294.8 (209.3 to 415.9) | 6575.7 (5004.5 to 8717.2) |  | 23.4 (15.9 to 34.4) | 730.0 (531.0 to 1004.0) |  | NA | 18058.5 (13676.1 to 23290.8) |  | 1109.3 (775.6 to 1509.6) | 100658.6 (77543.8 to 128591.0) |  | 600016.6 (404849.5 to 829014.2) | 2179.6 (1489.4 to 3236.7) |
| Nepal | 5.3 (2.2 to 9.8) | 144.2 (62.0 to 264.0) |  | 0.4 (0.2 to 0.8) | 16.8 (7.5 to 31.7) |  | NA | 963.7 (418.3 to 1954.0) |  | 83.2 (41.9 to 138.0) | 5332.7 (2890.2 to 8398.6) |  | 11534.5 (7912.1 to 16067.6) | 36.9 (23.4 to 59.5) |
| Pakistan | 199.7 (87.6 to 332.3) | 4486.2 (1979.8 to 7521.8) |  | 32.8 (12.4 to 71.6) | 1157.1 (440.0 to 2477.7) |  | NA | 8263.8 (3003.4 to 15014.2) |  | 1130.0 (628.1 to 1863.5) | 121752.0 (74069.6 to 180147.2) |  | 120785.5 (82464.7 to 169612.1) | 893.7 (393.0 to 1883.2) |
| **Southeast Asia, East Asia, and Oceania** | 2373.3 (1970.2 to 2843.1) | 35871.3 (29842.8 to 43262.4) |  | 151.6 (116.8 to 212.8) | 1834.3 (1402.3 to 2622.2) |  | NA | 33415.5 (26797.0 to 42096.4) |  | 5844.2 (4790.8 to 7118.3) | 151709.8 (128133.1 to 179258.9) |  | 1767034.7 (1255342.3 to 2414618.9) | 5964.1 (4736.0 to 8371.9) |
| **East Asia** | 1597.7 (1301.2 to 1908.5) | 20065.4 (16390.7 to 24022.8) |  | 126.5 (94.5 to 186.5) | 1163.3 (865.6 to 1714.4) |  | NA | 16711.2 (13181.3 to 21700.8) |  | 4531.7 (3660.4 to 5632.6) | 76712.8 (63136.8 to 91106.3) |  | 1412162.6 (996413.2 to 1924461.1) | 4561.2 (3484.4 to 6445.1) |
| China | 1547.1 (1255.4 to 1857.4) | 19472.0 (15949.3 to 23357.4) |  | 122.8 (90.9 to 182.6) | 1122.7 (840.2 to 1657.4) |  | NA | 16225.5 (12817.5 to 21100.3) |  | 4416.5 (3548.6 to 5522.7) | 74357.4 (60816.5 to 88575.1) |  | 1383747.1 (974485.1 to 1887110.6) | 4489.6 (3438.3 to 6369.2) |
| Democratic People's Republic of Korea | 24.8 (11.0 to 47.4) | 386.5 (180.7 to 709.9) |  | 1.5 (0.6 to 2.9) | 32.2 (12.4 to 62.1) |  | NA | 253.0 (114.6 to 536.5) |  | 49.6 (29.0 to 78.0) | 1743.4 (1173.4 to 2451.0) |  | 20312.9 (13375.4 to 28469.6) | 54.0 (29.5 to 93.5) |
| Taiwan (Province of China) | 25.8 (17.6 to 37.0) | 207.0 (145.9 to 285.9) |  | 2.2 (1.6 to 3.1) | 8.4 (6.6 to 10.6) |  | NA | 232.7 (158.0 to 328.2) |  | 65.6 (41.1 to 98.9) | 611.9 (454.1 to 788.7) |  | 8102.6 (6641.7 to 9995.8) | 17.5 (12.2 to 25.9) |
| **Oceania** | 17.7 (9.6 to 30.0) | 442.1 (231.0 to 759.4) |  | 0.4 (0.2 to 0.6) | 16.3 (8.4 to 30.0) |  | NA | 534.4 (303.0 to 831.9) |  | 45.7 (29.8 to 68.1) | 2942.7 (1978.4 to 4151.7) |  | 17277.4 (11669.5 to 24258.6) | 179.9 (81.0 to 365.2) |
| American Samoa | 0.0 (0.0 to 0.1) | 0.6 (0.3 to 1.2) |  | 0.0 (0.0 to 0.0) | 0.0 (0.0 to 0.0) |  | NA | 1.3 (0.6 to 2.6) |  | 0.1 (0.1 to 0.2) | 5.7 (3.3 to 9.2) |  | 48.4 (32.7 to 67.8) | 0.3 (0.1 to 0.6) |
| Cook Islands | 0.0 (0.0 to 0.0) | 0.1 (0.0 to 0.2) |  | 0.0 (0.0 to 0.0) | 0.0 (0.0 to 0.0) |  | NA | 0.1 (0.0 to 0.3) |  | 0.0 (0.0 to 0.0) | 0.6 (0.2 to 0.9) |  | 12.6 (8.5 to 17.6) | 0.1 (0.0 to 0.2) |
| Fiji | 1.0 (0.5 to 1.8) | 20.5 (11.3 to 34.9) |  | 0.0 (0.0 to 0.1) | 1.1 (0.5 to 2.4) |  | NA | 41.7 (23.4 to 66.5) |  | 3.1 (1.8 to 5.0) | 158.3 (103.4 to 233.6) |  | 829.9 (561.0 to 1164.4) | 8.5 (4.6 to 14.8) |
| Guam | 0.3 (0.2 to 0.5) | 4.3 (2.6 to 6.8) |  | 0.0 (0.0 to 0.0) | 0.1 (0.0 to 0.1) |  | NA | 6.0 (3.7 to 9.3) |  | 0.5 (0.3 to 0.7) | 11.8 (8.3 to 16.3) |  | 148.3 (100.2 to 208.1) | 1.2 (0.7 to 2.3) |
| Kiribati | 0.2 (0.1 to 0.5) | 6.6 (3.1 to 14.0) |  | 0.0 (0.0 to 0.0) | 0.1 (0.0 to 0.4) |  | NA | 5.3 (2.4 to 11.9) |  | 0.6 (0.3 to 1.1) | 35.5 (18.2 to 61.1) |  | 136.3 (92.1 to 191.3) | 0.6 (0.3 to 1.3) |
| Marshall Islands | 0.0 (0.0 to 0.1) | 0.9 (0.4 to 1.7) |  | 0.0 (0.0 to 0.0) | 0.0 (0.0 to 0.0) |  | NA | 1.3 (0.6 to 2.5) |  | 0.1 (0.1 to 0.2) | 6.8 (4.1 to 10.2) |  | 57.0 (38.5 to 80.0) | 0.4 (0.2 to 0.7) |
| Micronesia (Federated States of) | 0.1 (0.0 to 0.1) | 1.3 (0.3 to 2.7) |  | 0.0 (0.0 to 0.0) | 0.0 (0.0 to 0.1) |  | NA | 1.7 (0.2 to 3.6) |  | 0.2 (0.1 to 0.3) | 9.2 (3.5 to 14.4) |  | 91.1 (61.6 to 127.9) | 0.5 (0.2 to 1.0) |
| Nauru | 0.0 (0.0 to 0.1) | 0.6 (0.3 to 0.9) |  | 0.0 (0.0 to 0.0) | 0.0 (0.0 to 0.0) |  | NA | 0.9 (0.4 to 1.4) |  | 0.1 (0.0 to 0.1) | 2.7 (1.7 to 3.8) |  | 13.1 (8.9 to 18.4) | 0.2 (0.1 to 0.3) |
| Niue | 0.0 (0.0 to 0.0) | 0.1 (0.0 to 0.1) |  | 0.0 (0.0 to 0.0) | 0.0 (0.0 to 0.0) |  | NA | 0.1 (0.0 to 0.1) |  | 0.0 (0.0 to 0.0) | 0.2 (0.1 to 0.3) |  | 1.2 (0.8 to 1.7) | 0.0 (0.0 to 0.0) |
| Northern Mariana Islands | 0.0 (0.0 to 0.1) | 0.6 (0.3 to 1.1) |  | 0.0 (0.0 to 0.0) | 0.0 (0.0 to 0.0) |  | NA | 1 (0.5 to 1.7) |  | 0.1 (0.1 to 0.2) | 2.4 (1.4 to 3.6) |  | 21.4 (14.5 to 30.1) | 0.4 (0.2 to 0.7) |
| Palau | 0.0 (0.0 to 0.0) | 0.1 (0.1 to 0.2) |  | 0.0 (0.0 to 0.0) | 0.0 (0.0 to 0.0) |  | NA | 0.2 (0.1 to 0.3) |  | 0.2 (0.1 to 0.3) | 3.2 (2.0 to 5.0) |  | 9.2 (6.2 to 13.0) | 0.1 (0.0 to 0.1) |
| Papua New Guinea | 14.0 (6.5 to 25.4) | 363.3 (166.5 to 656.8) |  | 0.3 (0.1 to 0.5) | 13.0 (5.7 to 25.3) |  | NA | 278.1 (92.2 to 526.8) |  | 35.0 (20.7 to 55.4) | 2383.2 (1542.3 to 3484.1) |  | 13525.2 (9133.9 to 18998.4) | 150.6 (61.2 to 322.7) |
| Samoa | 0.2 (0.1 to 0.4) | 2.8 (1.1 to 6.6) |  | 0.0 (0.0 to 0.1) | 0.5 (0.1 to 1.5) |  | NA | 2.6 (0.9 to 6.1) |  | 0.4 (0.2 to 0.8) | 19.1 (9.6 to 32.2) |  | 195.7 (132.5 to 274.4) | 1.0 (0.4 to 2.0) |
| Solomon Islands | 0.6 (0.3 to 1.1) | 12.4 (5.8 to 23.6) |  | 0.0 (0.0 to 0.0) | 0.5 (0.3 to 0.8) |  | NA | 154.1 (82.5 to 259.8) |  | 2.1 (1.3 to 3.1) | 109.7 (71.3 to 152.5) |  | 896 (605.2 to 1257.8) | 5.0 (2.4 to 9.8) |
| Tokelau | 0.0 (0.0 to 0.0) | 0.0 (0.0 to 0.0) |  | 0.0 (0.0 to 0.0) | 0.0 (0.0 to 0.0) |  | NA | 0.0 (0.0 to 0.1) |  | 0.0 (0.0 to 0.0) | 0.1 (0.1 to 0.2) |  | 1.6(1.1to2.3) | 0.0 (0.0 to 0.0) |
| Tonga | 0.1 (0.1 to 0.2) | 2.2 (1.1 to 3.9) |  | 0.0 (0.0 to 0.0) | 0.1 (0.0 to 0.1) |  | NA | 7.5 (3.6 to 13.5) |  | 0.4 (0.2 to 0.8) | 16.9 (9.6 to 27.3) |  | 113.1 (76.5 to 158.8) | 0.7 (0.4 to 1.3) |
| Tuvalu | 0.0 (0.0 to 0.0) | 0.1 (0.1 to 0.3) |  | 0.0 (0.0 to 0.0) | 0.0 (0.0 to 0.0) |  | NA | 0.2 (0.1 to 0.4) |  | 0.0 (0.0 to 0.0) | 0.9 (0.6 to 1.4) |  | 10.0 (6.8 to 14.1) | 0.1 (0.0 to 0.1) |
| Vanuatu | 0.2 (0.1 to 0.4) | 4.8 (2.4 to 8.9) |  | 0.0 (0.0 to 0.0) | 0.2 (0.1 to 0.3) |  | NA | 7.1 (3.3 to 11.9) |  | 0.6 (0.4 to 1.0) | 37.5 (22.3 to 56.8) |  | 351.2 (237.3 to 492.8) | 1.9 (1.0 to 3.5) |
| **Southeast Asia** | 757.9 (545.7 to 1072.1) | 15363.8 (11054.7 to 21862.0) |  | 24.7 (15.8 to 40.4) | 654.6 (435.2 to 1052.3) |  | NA | 16169.9 (11835.2 to 22682.8) |  | 1266.8 (983.1 to 1577.3) | 72054.3 (56424.4 to 93172.3) |  | 337594.7 (234083.8 to 459134.1) | 1223.0 (858.1 to 2020.1) |
| Cambodia | 18.9 (10.7 to 31.3) | 376.9 (217.6 to 611.5) |  | 0.5 (0.2 to 1.0) | 16.9 (7.0 to 35.0) |  | NA | 337.6 (176.3 to 540.1) |  | 30.5 (18.1 to 49.1) | 1892.3 (1277.7 to 2685.8) |  | 10764.2 (7358.9 to 14842.1) | 30.1 (17.2 to 52.1) |
| Indonesia | 276.4 (167.7 to 451.3) | 6229.2 (3814.3 to 10415.1) |  | 4.9 (2.8 to 9.2) | 213.1 (119.0 to 427.7) |  | NA | 4987.4 (3387.6 to 7176.2) |  | 485.6 (323.3 to 644.6) | 30085.6 (21731.8 to 38860.8) |  | 129688.9 (91532.9 to 174444.2) | 439.8 (242.7 to 911.9) |
| Lao People's Democratic Republic | 8.7 (4.2 to 16.0) | 202.5 (97.1 to 363.8) |  | 0.2 (0.1 to 0.3) | 6.8 (2.5 to 14.2) |  | NA | 144.9 (63.0 to 265.3) |  | 13.4 (7.3 to 22.1) | 894.3 (514.7 to 1377.5) |  | 4883.3 (3337.7 to 6731.3) | 16.8 (8.0 to 34.2) |
| Malaysia | 13.5 (6.4 to 26.1) | 180.7 (90.0 to 337.7) |  | 1.1 (0.3 to 2.9) | 15.5 (4.4 to 43.1) |  | NA | 188.9 (69.5 to 408.4) |  | 37.7 (19.6 to 66.6) | 1098.7 (605.9 to 1836.7) |  | 15614.2 (10670.0 to 21502.6) | 90.7 (54.1 to 149.2) |
| Maldives | 0.5 (0.3 to 0.9) | 6.3 (3.8 to 10.4) |  | 0.1 (0.0 to 0.2) | 0.7 (0.3 to 1.7) |  | NA | 10.6 (6.0 to18.0) |  | 1.5 (0.9 to 2.5) | 51.7 (33.3 to 81.3) |  | 250.9 (171.6 to 345.7) | 4.0 (2.2 to 6.6) |
| Mauritius | 1.8 (1.2 to 2.6) | 23.4 (16.6 to 32.2) |  | 0.1 (0.0 to 0.1) | 0.8 (0.5 to 1.1) |  | NA | 20.9 (14.1 to 29.7) |  | 3.0 (2.1 to 4.4) | 77.1 (60.3 to 97.3) |  | 393.3 (269.2 to 542.5) | 2.0 (1.3 to 2.8) |
| Myanmar | 95.2 (42.6 to 190.7) | 2006.0 (882.7 to 4001.7) |  | 2.1 (0.7 to 5.0) | 78.4 (24.2 to 204.8) |  | NA | 6081.2 (3196.6 to 11128.7) |  | 111.6 (60.7 to 193.7) | 7940.7 (4602.9 to 13247.6) |  | 30592.4 (20914.5 to 42167.3) | 146.4 (70.8 to 331.1) |
| Philippines | 227.5 (164.8 to 302.8) | 4788.6 (3678.9 to 6016.4) |  | 3.1 (2.0 to 4.8) | 111.7 (76.3 to 183.2) |  | NA | 3175.6 (2431.9 to 4009.1) |  | 259.4 (179.6 to 349.2) | 18218.4 (13574.8 to 25701.6) |  | 73130.1 (50084.2 to 101324.8) | 309.1 (217.9 to 440.6) |
| Seychelles | 0.1 (0.0 to 0.1) | 0.8 (0.5 to 1.1) |  | 0.0 (0.0 to 0.0) | 0.1 (0.0 to 0.1) |  | NA | 1.7 (1.1 to 2.6) |  | 0.2 (0.1 to 0.3) | 5.1 (3.5 to 7.0) |  | 45.4 (31.1 to 62.7) | 0.1 (0.1 to 0.2) |
| Sri Lanka | 16.4 (9.8 to 26.1) | 195.1 (121.8 to 302.8) |  | 0.9 (0.5 to 2.1) | 10.6 (5.2 to 23.4) |  | NA | 172.5 (97.0 to 301.5) |  | 53.5 (31.9 to 83.3) | 1202.2 (807.0 to 1711.2) |  | 9426.6 (6445.2 to 13010.1) | 21.1 (13.2 to 33.9) |
| Thailand | 28.7 (13.9 to 45.1) | 360.3 (179.8 to 535.2) |  | 2.1 (0.7 to 4.0) | 23.7 (8.8 to 46.0) |  | NA | 313.7 (120.3 to 539.4) |  | 78.7 (36.2 to 126.7) | 2352.4 (1172.1 to 3268.1) |  | 19069.4 (13044.1 to 26311) | 40.1 (22.9 to 61.1) |
| Timor-Leste | 1.9 (0.6 to 3.7) | 40.8 (13.9 to 78.6) |  | 0.0 (0.0 to 0.1) | 1.8 (0.3 to 4.3) |  | NA | 36.8 (6.3 to 70.8) |  | 3.1 (1.1 to 5.1) | 203.6 (61.8 to 323.1) |  | 1069.4 (730.3 to 1474.8) | 3.2 (1.3 to 6.4) |
| Viet Nam | 67.5 (34.9 to 121.0) | 933.2 (488.7 to 1611.4) |  | 9.8 (3.2 to 26.0) | 173.8 (54.4 to 444.2) |  | NA | 676.8 (387.0 to 1100.4) |  | 186.9 (106.6 to 309.5) | 7938.0 (4783.6 to 14526.1) |  | 42224.2 (28856.6 to 58217.2) | 117.8 (69.4 to 192.4) |
| **Sub-Saharan Africa** | 1981.5 (1217.3 to 2878.1) | 68468.1 (40672.2 to 101514.6) |  | 119.6 (41.7 to 210.4) | 7078.4 (2339.7 to 12662.3) |  | NA | 70904.2 (43400.0 to 101875.7) |  | 3816.6 (2836.2 to 5203.3) | 543003.2 (368941.6 to 773276.1) |  | 1490122.9 (1022668.6 to 2080553.2) | 3101.2 (1994.5 to 5777.3) |
| **Central Sub-Saharan Africa** | 100.9 (49.4 to 181.1) | 3351.7 (1638.5 to 5982.1) |  | 4.5 (1.6 to 9.4) | 234.8 (82.0 to 486.8) |  | NA | 6632.6 (2419.4 to 11561.4) |  | 306.5 (167.4 to 498.5) | 34748.6 (21684.1 to 50977.8) |  | 204918.0 (140091.9 to 286476.3) | 318.7 (177.0 to 746.8) |
| Angola | 41.0 (16.3 to 80.4) | 1323.5 (524.0 to 2637.4) |  | 1.4 (0.5 to 3.4) | 73.0 (25.7 to 178.3) |  | NA | 2811.4 (791.1 to 5559.9) |  | 79.1 (46.2 to 125.1) | 10091.2 (6309.4 to 15413.5) |  | 50995.8 (34903.2 to 71282.7) | 131.5 (59.4 to 351.6) |
| Central African Republic | 4.7 (1.4 to 9.7) | 185.1 (55.9 to 380.8) |  | 0.3 (0.1 to 1.0) | 19.9 (4.0 to 57.4) |  | NA | 450.8 (88.2 to 978.9) |  | 13.5 (7.3 to 27.1) | 2014.3 (1141.3 to 3285.0) |  | 8322.2 (5686.5 to 11667.4) | 14.8 (6.5 to 42.5) |
| Congo | 3.8 (1.6 to 9.1) | 121.1 (50.7 to 280.9) |  | 0.1 (0.0 to 0.2) | 5.3 (2.0 to 11.7) |  | NA | 194.7 (75.5 to 401) |  | 7.2 (3.8 to 12.3) | 816.6 (469.4 to 1297.8) |  | 6893.0 (4715.7 to 9625.2) | 11.2 (5.5 to 29.8) |
| Democratic Republic of the Congo | 48.4 (21.6 to 94.1) | 1639.5 (742.1 to 3190.1) |  | 2.5 (0.8 to 6.4) | 134.2 (40.2 to 332.7) |  | NA | 3053.3 (1098.2 to 6066.5) |  | 202.7 (93.5 to 376.5) | 21385.9 (11722.8 to 35266.1) |  | 134876.5 (92166.0 to 188550.8) | 152.6 (85.1 to 313.2) |
| Equatorial Guinea | 1.2 (0.4 to 2.9) | 32.5 (10.8 to 78.9) |  | 0.0 (0.0 to 0.1) | 0.9 (0.3 to 2.1) |  | NA | 48.4 (14.6 to 119.3) |  | 1.7 (0.8 to 3.3) | 179.0 (80.0 to 325.1) |  | 1827.8 (1249.7 to 2552.2) | 3.9 (1.4 to 11.7) |
| Gabon | 1.8 (0.6 to 4.1) | 50.0 (16.9 to 112.0) |  | 0.0 (0.0 to 0.1) | 1.6 (0.5 to 3.6) |  | NA | 73.9 (25.2 to 150.4) |  | 2.3 (1.0 to 4.2) | 261.7 (134.7 to 442.4) |  | 2002.6 (1370.7 to 2798.0) | 4.7 (2.0 to 13.1) |
| **Eastern Sub-Saharan Africa** | 564.6 (404.5 to 785.2) | 17847.7 (12716.0 to 24729.2) |  | 30.5 (10.6 to 60.6) | 1594.7 (542.8 to 3123.1) |  | NA | 25547.8 (16718.4 to 37946.9) |  | 1420.7 (1048.9 to 1904.6) | 302643.8 (191870.8 to 461663.1) |  | 384366.9 (261633.6 to 540309.3) | 1279.1 (794.0 to 2554.2) |
| Burundi | 12.3 (5.6 to 23.4) | 417.6 (196.2 to 818.9) |  | 0.8 (0.2 to 2.1) | 41.6 (9.8 to 113.7) |  | NA | 656.0 (270.1 to 1362.2) |  | 39.8 (21.4 to 70.7) | 8009.0 (3946.8 to 16651.3) |  | 12162.7 (8255.6 to 17015.0) | 30.0 (14.8 to 63.2) |
| Comoros | 0.6 (0.3 to 1.1) | 19.6 (10.3 to 35.9) |  | 0.0 (0.0 to 0.0) | 1.0 (0.4 to 2.3) |  | NA | 23.8 (9.4 to 53.9) |  | 1.6 (0.8 to 2.5) | 233.8 (143.3 to 376.8) |  | 457.1 (310.5 to 639.0) | 1.3 (0.8 to 2.3) |
| Djibouti | 2.1 (1.2 to 3.4) | 62.1 (35.5 to 99.5) |  | 0.1 (0.0 to 0.2) | 4.2 (1.3 to 10.6) |  | NA | 96.8 (49.9 to 179.3) |  | 4.3 (2.6 to 6.6) | 860.7 (481.4 to 1429.5) |  | 928.9 (630.5 to 1299.6) | 4.6 (2.3 to 10.5) |
| Eritrea | 7.1 (3.7 to 12.9) | 240.6 (125.8 to 436.2) |  | 0.5 (0.1 to 1.2) | 25.6 (7.4 to 63.8) |  | NA | 445.1 (220.2 to 838.7) |  | 19.8 (11.3 to 31.5) | 4336.5 (2351.4 to 7299.9) |  | 5435.1 (3690.8 to 7601.6) | 19.4 (10.7 to 41.1) |
| Ethiopia | 85.1 (54.7 to 130.4) | 2574.5 (1633.1 to 3952.0) |  | 7.8 (1.8 to 17.9) | 448.1 (99.0 to 1046.2) |  | NA | 1037.0 (654.7 to 1552.4) |  | 324.9 (215.8 to 473.7) | 50838.5 (23256.7 to 84481.1) |  | 104105.4 (71130.9 to 145544.1) | 266.2 (168.8 to 471.4) |
| Kenya | 19.5 (13.5 to 27.7) | 545.0 (386.0 to 772.2) |  | 1.1 (0.6 to 1.8) | 46.5 (22.9 to 76.2) |  | NA | 1732.0 (1089.8 to 2670.3) |  | 70.7 (46.7 to 125.3) | 7175.6 (4053.3 to 14169.9) |  | 40163.3 (27460.4 to 56114.8) | 82.8 (56.7 to 140.4) |
| Madagascar | 20.1 (11.4 to 32.4) | 681.2 (387.6 to 1087.3) |  | 1.0 (0.3 to 2.3) | 51.0 (14.4 to 120.5) |  | NA | 875.3 (460.2 to 1561.7) |  | 61.4 (37.8 to 99.1) | 10737.7 (6371.1 to 18662.9) |  | 23013.4 (15628.0 to 32186.9) | 48.7 (27.6 to 107.7) |
| Malawi | 47.8 (27.2 to 81.6) | 1533.2 (881.6 to 2628.5) |  | 1.0 (0.3 to 2.3) | 51.2 (17.4 to 117.8) |  | NA | 1650.2 (909.2 to 2789.6) |  | 56.4 (34.3 to 88.2) | 38804.3 (22501.3 to 63116.1) |  | 15136.7 (10282.4 to 21160.3) | 47.8 (29.2 to 90.4) |
| Mozambique | 58.8 (30.6 to 109.2) | 1981.9 (1030.0 to 3655.4) |  | 2.2 (0.7 to 5.7) | 117.7 (36.0 to 307.4) |  | NA | 934.2 (321.4 to 2474.3) |  | 163.8 (98.8 to 255.9) | 24692.0 (14671.7 to 38298.9) |  | 29862.2 (20276.1 to 41774.2) | 104.9 (62.4 to 184.4) |
| Rwanda | 16.2 (8.8 to 28.3) | 487.1 (263.1 to 843.0) |  | 0.8 (0.2 to 2.1) | 39.9 (11.2 to 101.1) |  | NA | 803.6 (407.5 to 1466.5) |  | 36.4 (20.3 to 60.0) | 7890.7 (4187.6 to 13041.7) |  | 9658.2 (6561.3 to 13502.9) | 37.9 (20.1 to 75.8) |
| Somalia | 19.0 (7.9 to 36.3) | 729.3 (305.5 to 1376.1) |  | 2.3 (0.4 to 6.8) | 133.2 (25.8 to 393.7) |  | NA | 1156.1 (532.4 to 2143.5) |  | 78.9 (48.2 to 124.8) | 17155.5 (10248.3 to 27793.9) |  | 21780.9 (14775.3 to 30499.2) | 47.9 (27.8 to 95.8) |
| South Sudan | 19.5 (9.4 to 43.0) | 698.3 (342.7 to 1550.6) |  | 1.0 (0.2 to 2.9) | 52.8 (10.6 to 159.1) |  | NA | 1301.1 (470.9 to 2601.4) |  | 39.6 (23.6 to 61.2) | 9136.9 (4863.4 to 14749.9) |  | 8853.1 (6005.6 to 12399.0) | 62.3 (18.5 to 211.7) |
| Uganda | 78.4 (43.1 to 135.1) | 2455.4 (1349.8 to 4169.1) |  | 1.0 (0.2 to 2.9) | 52.8 (10.6 to 159.1) |  | NA | 3396.7 (1724.2 to 5960.8) |  | 139.3 (78.6 to 214.0) | 43001.3 (22293.0 to 73697.2) |  | 41475.4 (28170.7 to 58018.0) | 141.6 (69.9 to 309.4) |
| United Republic of Tanzania | 144.4 (82.7 to 239.4) | 4406.0 (2586.2 to 7343.9) |  | 4.2 (1.0 to 11.7) | 205.7 (46.4 to 584.8) |  | NA | 9940.1 (5303.1 to 17077.9) |  | 321.6 (196.1 to 499.5) | 66081.2 (39548.0 to 104834.1) |  | 54507.6 (37010.2 to 76250.0) | 309.0 (157.7 to 704.1) |
| Zambia | 33.0 (18.3 to 54.9) | 1001.6 (557.0 to 1650.0) |  | 6.4 (1.9 to 15.3) | 310.3 (92.5 to 743.5) |  | NA | 1479.5 (776.2 to 2707.1) |  | 61.2 (38.0 to 95.1) | 13448.4 (7793.9 to 22112.4) |  | 16519.5 (11218.3 to 23096.2) | 73.5 (40.1 to 162.2) |
| **Southern Sub-Saharan Africa** | 54.3 (37.9 to 74.5) | 1236.7 (880.0 to 1685.2) |  | 1.4 (0.4 to 3.4) | 64.6 (20.5 to 164.2) |  | NA | 840.9 (515.9 to 1220.5) |  | 71.1 (52.8 to 94.8) | 5868.8 (4402.9 to 7692.7) |  | 156739.6 (108789.3 to 212930.3) | 165.4 (118.3 to 223.3) |
| Botswana | 2.5 (1.2 to 4.5) | 57.1 (26.4 to 100.4) |  | 1.5 (0.9 to 2.4) | 51.1 (29.9 to 82.4) |  | NA | 38.9 (13.4 to 75.1) |  | 3.4 (1.9 to 5.5) | 191.3 (114.2 to 299.8) |  | 4340.3 (2994.2 to 5954.3) | 5.4 (3.7 to 7.8) |
| Eswatini | 1.3 (0.7 to 2.3) | 39.7 (20.0 to 70.7) |  | 0.1 (0.0 to 0.1) | 1.8 (1.1 to 2.9) |  | NA | 27.3 (11.5 to 49.3) |  | 1.5 (0.9 to 2.3) | 138.3 (89.8 to 199.0) |  | 2576.8 (1779.0 to 3533.3) | 2.8 (2.0 to 3.8) |
| Lesotho | 1.2 (0.7 to 2.0) | 39.9 (22.1 to 65.2) |  | 0.0 (0.0 to 0.1) | 1.2 (0.6 to 2.4) |  | NA | 31.8 (14.0 to 55.8) |  | 2.2 (1.3 to 3.3) | 201.6 (133.3 to 294.6) |  | 4006.9 (2764.7 to 5501.7) | 3.5 (2.5 to 4.9) |
| Namibia | 1.8 (0.9 to 3.2) | 46.8 (22.4 to 79.7) |  | 0.0 (0.0 to 0.1) | 1.8 (0.9 to 3.9) |  | NA | 41.9 (14.6 to 74.3) |  | 3.0 (1.7 to 4.7) | 317.4 (168.0 to 489.0) |  | 5443.1 (3750.5 to 7472.3) | 5.5 (3.4 to 8.1) |
| South Africa | 39.2 (27.0 to 54.0) | 788.5 (560.6 to 1070.6) |  | 0.1 (0.0 to 0.3) | 4.0 (1.1 to 11.0) |  | NA | 571.0 (353.2 to 826.0) |  | 43.5 (31.7 to 58.4) | 3315.9 (2362.7 to 4423.0) |  | 101704.6 (70873.4 to 137495.5) | 79.2 (58.2 to 104.8) |
| Zimbabwe | 8.3 (4.3 to 14.2) | 264.7 (139.0 to 442.2) |  | 0.9 (0.6 to 1.4) | 24.5 (15.2 to 37.5) |  | NA | 130.1 (53.8 to 241.5) |  | 17.6 (10.2 to 27.7) | 1704.4 (1078.2 to 2571.2) |  | 38667.9 (26651.9 to 53064.9) | 68.9 (40.4 to 110.1) |
| **Western Sub-Saharan Africa** | 1261.8 (637.3 to 1965.6) | 46032.0 (22117.3 to 74055.4) |  | 0.4 (0.2 to 0.8) | 17.8 (7.8 to 38.8) |  | NA | 37883.0 (17271.4 to 61344.1) |  | 2018.3 (1422.0 to 2865.0) | 199741.9 (134335.4 to 278606.7) |  | 744098.4 (512734.2 to 1037193.4) | 1338.0 (814.0 to 2408.7) |
| Benin | 70.5 (21.6 to 138.1) | 2307.4 (679.3 to 4614.1) |  | 83.1 (26.7 to 150.2) | 5197.7 (1561.6 to 9632.8) |  | NA | 1602.5 (510.9 to 3022.3) |  | 76.6 (43.5 to 120.2) | 6592.1 (3951.9 to 9672.1) |  | 21997.4 (15017.1 to 30813.2) | 42.7 (25.0 to 76.4) |
| Burkina Faso | 120.5 (39.0 to 243.6) | 3976.5 (1263.2 to 7985.6) |  | 0.9 (0.3 to 2.0) | 44.4 (15.5 to 101.6) |  | NA | 3673.1 (949.0 to 7647.3) |  | 184.6 (112.8 to 284.1) | 15907.4 (10378.9 to 23989.1) |  | 39918.9 (27254.9 to 55971.6) | 90.8 (49.7 to 187.2) |
| Cabo Verde | 1.2 (0.5 to 2.2) | 26.4 (11.1 to 48.8) |  | 2.1 (0.7 to 4.8) | 105.0 (35.1 to 239.7) |  | NA | 14.7 (6.0 to26.9) |  | 1.6 (0.7 to 2.7) | 83.0 (41.6 to 134.4) |  | 525.3 (358.9 to 733.5) | 0.8 (0.4 to 1.3) |
| Cameroon | 45.1 (20.1 to 88.6) | 1392.7 (629.7 to 2736.2) |  | 0.0 (0.0 to 0.0) | 0.3 (0.1 to 0.5) |  | NA | 2706.1 (1349.6 to 4740.9) |  | 107.4 (58.2 to 189.9) | 7229.4 (4143.5 to 11992.2) |  | 41579.1 (28404.9 to 58131.7) | 69.9 (35.6 to 142.2) |
| Chad | 54.4 (15.6 to 112.1) | 1960.1 (561.9 to 4025.9) |  | 1.3 (0.6 to 2.7) | 61.2 (26.0 to 127.0) |  | NA | 2023.6 (543.1 to 4116.3) |  | 91.9 (50.3 to 154.7) | 8102.9 (4690.2 to 12332.0) |  | 32886.4 (22450.6 to 46123.1) | 49.7 (27.3 to 105.7) |
| Côte d'Ivoire | 90.6 (29.4 to 174.4) | 3013.6 (983.1 to 5736.7) |  | 1.2 (0.4 to 2.7) | 65.6 (19.5 to 148.2) |  | NA | 2163.2 (771.8 to 4264.5) |  | 101.1 (56.7 to 159.7) | 8482.4 (4930.4 to 13006.4) |  | 39610.4 (27050.3 to 55443.2) | 62.8 (33.6 to 111.2) |
| Gambia | 3.0 (1.4 to 5.5) | 93.9 (43.0 to 171.8) |  | 1.2 (0.5 to 2.7) | 60.0 (24.0 to 133.5) |  | NA | 29.2 (15.1 to 54.1) |  | 6.6 (3.6 to 10.7) | 602.1 (363.9 to 913.6) |  | 3255.1 (2222.5 to 4554.0) | 5.0 (3.0 to 7.6) |
| Ghana | 64.1 (26.6 to 125.7) | 1857.7 (780.3 to 3664.4) |  | 0.1 (0.1 to 0.3) | 7.1 (2.9 to 15.1) |  | NA | 2872.5 (1305.2 to 5672.7) |  | 82.0 (44.5 to 146.1) | 6196.3 (3479.8 to 9804.2) |  | 39228.8 (26798.7 to 54858.8) | 76.6 (41.1 to 128.8) |
| Guinea | 44.7 (17.0 to 86.8) | 1584.7 (611.3 to 3082.0) |  | 0.8 (0.3 to 1.8) | 32.0 (13.7 to 76.6) |  | NA | 413.8 (211.4 to 693.2) |  | 47.3 (28.7 to 73.2) | 7323.2 (4366.4 to 11404.6) |  | 21161.9 (14449.9 to 29632.9) | 40.3 (22.2 to 75.5) |
| Guinea-Bissau | 3.8 (1.3 to 7.7) | 133.2 (47.1 to 275.9) |  | 4.0 (1.5 to 9.4) | 211.6 (78.4 to 494.9) |  | NA | 105.3 (37.0 to 209.3) |  | 6.7 (3.8 to 10.6) | 542.6 (306.1 to 844.5) |  | 2839.4 (1938.8 to 3971.7) | 3.5 (1.9 to 6.0) |
| Liberia | 7.6 (2.6 to 15.8) | 246.8 (82.7 to 513.5) |  | 0.1 (0.0 to 0.2) | 4.0 (1.3 to 8.8) |  | NA | 150.9 (60.9 to 311.8) |  | 11.3 (6.0 to 19.7) | 880.7 (496.2 to 1475.9) |  | 6252.0 (4267.8 to 8738.2) | 5.8 (3.4 to 9.7) |
| Mali | 117.7 (40.3 to 230.9) | 3939.2 (1336.5 to 7796.8) |  | 0.1 (0.0 to 0.2) | 5.4 (2.2 to 11.4) |  | NA | 864.5 (316.8 to 1632.7) |  | 109.5 (67.7 to 165.7) | 11293.7 (6917.0 to 17086.7) |  | 40105.2 (27380.7 to 56240.2) | 82.7 (47.5 to 154.4) |
| Mauritania | 4.7 (2.1 to 9.2) | 133.5 (59.6 to 259.4) |  | 3.5 (0.9 to 8.7) | 177.6 (45.1 to 453.3) |  | NA | 80.6 (36.4 to 157.7) |  | 8.6 (4.3 to 15.7) | 571.0 (307.9 to 977.6) |  | 5250.0 (3585.6 to 7329.2) | 4.5 (2.6 to 7.7) |
| Niger | 56.2 (20.3 to 104.2) | 1952.7 (663.9 to 3864.7) |  | 0.1 (0.0 to 0.1) | 2.7 (1.1 to 5.4) |  | NA | 1166.2 (399.4 to 2208.1) |  | 141.1 (85.3 to 229.5) | 12880.3 (7173.7 to 21286.9) |  | 47867.7 (32673.4 to 67124.2) | 72.7 (41.1 to 161.6) |
| Nigeria | 498.1 (217.7 to 899.9) | 20838.4 (8646.4 to 38767.9) |  | 2.2 (0.8 to 5.1) | 117.6 (41.5 to 275.7) |  | NA | 18128.5 (6501.9 to 32319.4) |  | 902.6 (575.8 to 1427.3) | 102246.7 (64755.3 to 146971.9) |  | 356986.7 (248016.3 to 489956.6) | 662.3 (367.4 to 1305.7) |
| Sao Tome and Principe | 0.7 (0.2 to 2.1) | 19.3 (5.9 to 60.9) |  | 64.2 (16.7 to 123.3) | 4236.7 (1079.8 to 8113.7) |  | NA | 5.5 (2.6 to 11.4) |  | 5.8 (2.0 to 10.8) | 299.9 (110.1 to 554.6) |  | 230.6 (157.6 to 321.8) | 0.4 (0.2 to 0.6) |
| Senegal | 26.9 (13.1 to 49.8) | 856.1 (415.8 to 1558.8) |  | 0.0 (0.0 to 0.0) | 0.1 (0.1 to 0.2) |  | NA | 622.2 (320.4 to 1056.4) |  | 59.3 (32.7 to 101.2) | 4218.8 (2436.2 to 6716.0) |  | 21230.8 (14497.0 to 29696.6) | 29.4 (17.0 to 44.6) |
| Sierra Leone | 37.6 (12.6 to 75.6) | 1245.2 (404.4 to 2528.6) |  | 0.5 (0.2 to 1.1) | 25.2 (9.9 to 51.4) |  | NA | 938.0 (335.6 to 1867.2) |  | 53.3 (29.4 to 92.5) | 4593.5 (2615.1 to 7290.1) |  | 12149.2 (8295.5 to 17015.7) | 25.9 (14.2 to 47.7) |
| Togo | 14.5 (5.1 to 34.6) | 453.9 (161.3 to 1104.0) |  | 0.6 (0.2 to 1.4) | 29.9 (10.2 to 70.6) |  | NA | 322.0 (126.2 to 668.1) |  | 20.9 (11.5 to 37.6) | 1693.3 (971.6 to 3036.6) |  | 11012.7 (7520.7 to 15393.8) | 12.1 (6.4 to 26.3) |

Data in parentheses are 95% uncertainty intervals (UIs) unless otherwise stated.

Abbreviations: Cases = incidence cases; DALYs = disability-adjusted life-years; SDI = Sociodemographic index; NA = not available.

**Supplementary Table 6.** Incidences and DALYs of total and subtypes of leukemia and other neoplasms among children under 5-year-old globally, and by SDI regions, GBD super-regions, GBD regions, countries and territories in 2019.

|  | **Acute lymphoid leukemia** | |  | **Acute myeloid leukemia** | |  | **Other leukemia** | |  | **Myelodysplastic, myeloproliferative, and other hematopoietic neoplasms** | |  | **Benign and in situ intestinal neoplasms** | |  | **Benign and in situ cervical and uterine neoplasms** | |  | **Other benign and in situ neoplasms** | |
| --- | --- | --- | --- | --- | --- | --- | --- | --- | --- | --- | --- | --- | --- | --- | --- | --- | --- | --- | --- | --- |
|  | **Cases** | **DALYs** |  | **Cases** | **DALYs** |  | **Cases** | **DALYs** |  | **Cases** | **DALYs** |  | **Cases** | **DALYs** |  | **Cases** | **DALYs** |  | **Cases** | **DALYs** |
| **Global** | 14839.6 (11460.4 to 18535.3) | 520731.5 (367311.6 to 678587.9) |  | 6617.9  (5069.7 to 8500.3) | 313264.2 (241310.9 to 398865.5) |  | 35823.9 (28358.0 to 45633.2) | 501951.0 (379444.0 to 658289.8) |  | 19253.9 (12260.8 to 28111.8) | 23301.3 (18919.8 to 28932.3) |  | 14039.8 (7556.7 to 22498.7) | 0.0 (0.0 to 0.0) |  | 4620.8 (2153.0 to 7918.5) | 0.0 (0.0 to 0.0) |  | 8612225 (6096794.4 to 11592099.3) | NA |
| High SDI | 1865.0 (1461.7 to 2330.5) | 13537.1 (11242.8 to 16012.1) |  | 329.9 (256.2 to 410.5) | 14581.8 (11610.5 to 16553.4) |  | 1072.7 (832.8 to 1326.3) | 7838.3 (6373.7 to 9324.8) |  | 1229.5 (876.4 to 1686.6) | 2656.3 (1930.8 to 3151.6) |  | 2721.0 (1703.6 to 4013.9) | 0.0 (0.0 to 0.0) |  | 234.8 (101.3 to 431.4) | 0.0 (0.0 to 0.0) |  | 1389644.3 (1011911.1 to 1845735.4) | NA |
| High-middle SDI | 3536.6 (2588.2 to 4781.6) | 57319.7 (45544.1 to 70758.9) |  | 643.7 (507.0 to 808.1) | 31743.2 (25193.5 to 40089.4) |  | 4913.6 (3448.0 to 6938.4) | 43445.4 (30835.0 to 60869.0) |  | 2213.3 (1497.9 to 3159.6) | 3974.2 (3069.0 to 4801.0) |  | 1587.6 (820.0 to 2596.6) | 0.0 (0.0 to 0.0) |  | 504.5 (209.7 to 895.1) | 0.0 (0.0 to 0.0) |  | 1883839.6 (1351207.3 to 2526013.3) | NA |
| Middle SDI | 3614.0 (2716.5 to 4648.4) | 132043.2 (103703.7 to 162828.2) |  | 1260.9 (980.0 to 1671.6) | 63979.9 (49770.7 to 87194.6) |  | 9854.4 (7412.4 to 13184.0) | 120113.9 (92687.9 to 159071.5) |  | 5986.1 (3931.1 to 8502.5) | 7785.3 (6187.5 to 9755.9) |  | 4066.7 (2212.1 to 6406.0) | 0.0 (0.0 to 0.0) |  | 1704.3 (899.8 to 2746.8) | 0.0 (0.0 to 0.0) |  | 2453186.2 (1730533.9 to 3291301.6) | NA |
| Low-middle SDI | 2003.7 (1428.0 to 2959.8) | 109017.0 (79715.6 to 154013.8) |  | 1668.4 (1205.1 to 2342.3) | 76971.3 (57085.4 to 103986.9) |  | 7252.6 (4908.0 to 10266.0) | 106555.1 (72343.0 to 150821.8) |  | 4937.8 (2969.6 to 7232.3) | 5261.5 (3974.0 to 7021.2) |  | 3387.7 (1723.8 to 5567.5) | 0.0 (0.0 to 0.0) |  | 1265.4 (566.6 to 2185.5) | 0.0 (0.0 to 0.0) |  | 1417462.3 (976916.9 to 1938988) | NA |
| Low SDI | 3247.4 (1806.4 to 5178.2) | 208357.8 (115863.0 to 327609.1) |  | 2349.5 (1320.0 to 3432.5) | 125749.4 (70038.2 to 182227.4) |  | 11314.1 (5870.4 to 16765.6) | 223414.1 (121390.5 to 321574.5) |  | 4141.2 (2419.9 to 6457.2) | 3595.8 (2333.8 to 6595.5) |  | 2052.6 (845.1 to 3746.5) | 0.0 (0.0 to 0.0) |  | 504.5 (209.7 to 895.1) | 0.0 (0.0 to 0.0) |  | 1290683.8 (884087.9 to 1789917.5) | NA |
| **Central Europe, Eastern Europe, and Central Asia** | 581.5 (463.8 to 723.6) | 17089.7 (14006.4 to 20831.9) |  | 199.4 (157.9 to 244.8) | 9974.5 (7785.0 to 12408.5) |  | 612.3 (460.8 to 819.2) | 6788.6 (4963.4 to 9250.4) |  | 313.7 (183.2 to 484.3) | 574.4 (358.7 to 740.8) |  | 960.0 (536.1 to 1472.5) | 0.0 (0.0 to 0.0) |  | 177.5 (69.6 to 315.9) | 0.0 (0.0 to 0.0) |  | 1266979.1 (907307.3to 1709222.9) | NA |
| **Central Asia** | 100.2 (70.7 to 137.7) | 5274.4 (3784.3 to 7155.3) |  | 79.0 (58.7 to 105.9) | 3912.7 (2924.8 to 5182.2) |  | 363.7 (244.4 to 537.6) | 4503.9 (2969.7 to 6584.0) |  | 46.9 (22.5 to 79.5) | 187.5 (108.1 to 270.3) |  | 284.3 (148.0 to 456.2) | 0.0 (0.0 to 0.0) |  | 55.1 (18.8 to 101.8) | 0.0 (0.0 to 0.0) |  | 364328.2 (254904.0 to 500094.9) | NA |
| Armenia | 1.7 (0.7 to 5.5) | 70.0 (28.4 to 214.0) |  | 4.4 (2.1 to 6.8) | 224.3 (106.0 to 347.7) |  | 14.2 (9.5 to 20.9) | 141.4 (95.1 to 206.6) |  | 0.8 (0.4 to 1.5) | 2.4 (1.7 to 4.0) |  | 6.0 (3.1 to 9.6) | 0.0 (0.0 to 0.0) |  | 1.1 (0.4 to 2.1) | 0.0 (0.0 to 0.0) |  | 7741.4 (5374.6 to 10655.9) | NA |
| Azerbaijan | 8.2 (3.1 to 17.3) | 450.4 (170.9 to 934.0) |  | 7.1 (2.9 to 14.9) | 344.6 (136.6 to 727.2) |  | 102.3 (44.3 to 215.4) | 1299.0 (571.2 to 2679.8) |  | 3.8 (1.7 to 6.7) | 11.2 (5.6 to 20.6) |  | 22.2 (11.6 to 35.7) | 0.0 (0.0 to 0.0) |  | 4.2 (1.4 to 7.8) | 0.0 (0.0 to 0.0) |  | 28769.1 (19965.3 to 39609.0) | NA |
| Georgia | 3.0 (2.0 to 4.6) | 154.5 (104.7 to 231.5) |  | 2.0 (1.2 to 3.3) | 99.5 (58.7 to 167.4) |  | 10.9 (6.6 to 18.0) | 129.5 (77.1 to 212.7) |  | 1.2 (0.7 to 2.0) | 14.1 (2.9 to 26.6) |  | 7.9 (4.5 to 12.4) | 0.0 (0.0 to 0.0) |  | 1.5 (0.5 to 2.7) | 0.0 (0.0 to 0.0) |  | 8727.7 (6809.1 to 11122.7) | NA |
| Kazakhstan | 26.8 (17.4 to 40.3) | 1197.4 (804.1 to 1708.6) |  | 18.1 (11.7 to 27.3) | 895.3 (579.5 to 1323.0) |  | 47.3 (29.1 to 76.6) | 504.2 (298.6 to 802.5) |  | 10.7 (5.3 to 18.7) | 85.1 (27.7 to 145.6) |  | 54.5 (28.5 to 87.5) | 0.0 (0.0 to 0.0) |  | 10.7 (3.6 to 19.7) | 0.0 (0.0 to 0.0) |  | 69758 (48352 to 96033.7) | NA |
| Kyrgyzstan | 5.4 (3.5 to 7.5) | 299.4 (199.4 to 413.4) |  | 3.7 (2.4 to 5.3) | 184.9 (124.1 to 266.0) |  | 10.9 (7.7 to 16.5) | 133.5 (95.3 to 198.5) |  | 4.3 (2.1 to 7.2) | 9.1 (7.2 to 11.4) |  | 23.3 (12.0 to 37.9) | 0.0 (0.0 to 0.0) |  | 4.4 (1.5 to 8.1) | 0.0 (0.0 to 0.0) |  | 30812.3 (21465.3 to 42674.6) | NA |
| Mongolia | 1.7 (0.7 to 4.5) | 103.5 (43.4 to 264.9) |  | 2.3 (0.9 to 4.9) | 108.3 (45.7 to 234.3) |  | 2.4 (1.0 to 4.8) | 32.3 (13.8 to 66.0) |  | 1.8 (0.8 to 3.2) | 3.6 (2.6 to 4.8) |  | 11.7 (6.1 to 18.8) | 0.0 (0.0 to 0.0) |  | 2.3 (0.8 to 4.3) | 0.0 (0.0 to 0.0) |  | 14939.0 (10340.5 to 20590.4) | NA |
| Tajikistan | 48.8 (20.7 to 119.6) | 724.2 (303.7 to 1783.2) |  | 8.8 (3.7 to 17.7) | 434.0 (185.4 to 885.7) |  | 48.8 (20.7 to 119.6) | 724.2 (303.7 to 1783.2) |  | 5.7 (2.5 to 9.8) | 14.0 (10.4 to 18.4) |  | 35.7 (18.7 to 57.4) | 0.0 (0.0 to 0.0) |  | 7.0 (2.4 to 12.9) | 0.0 (0.0 to 0.0) |  | 45710.5 (31645.5 to 63028.2) | NA |
| Turkmenistan | 6.3 (3.9 to 9.6) | 353.3 (219.2 to 522.6) |  | 5.4 (3.3 to 8.1) | 271.4 (169.1 to 407.7) |  | 23.5 (14.7 to 35.2) | 287.6 (183.7 to 431.2) |  | 3.0 (1.4 to 5.5) | 9.1 (5.5 to 17.4) |  | 16.3 (8.5 to 26.2) | 0.0 (0.0 to 0.0) |  | 3.2 (1.1 to 5.9) | 0.0 (0.0 to 0.0) |  | 20871.4 (14456.2 to 28768.1) | NA |
| Uzbekistan | 34.6 (22.9 to 50.1) | 1897.7 (1277.2 to 2728.7) |  | 27.3 (17.6 to 42.2) | 1350.4 (868.4 to 2017.3) |  | 103.3 (66.2 to 163.6) | 1252.2 (812.9 to 1980.8) |  | 15.6 (6.7 to 28.6) | 39.1 (29.7 to 59.4) |  | 106.7 (55.9 to 171.4) | 0.0 (0.0 to 0.0) |  | 20.7 (7.1 to 38.4) | 0.0 (0.0 to 0.0) |  | 136998.8 (94968.3 to 188734.8) | NA |
| **Central Europe** | 93.4 (69.3 to 124.7) | 809.3 (621.1 to 1051.1) |  | 32.1 (24.1 to 41.4) | 1620.9 (1226.9 to 2112.4) |  | 93.4 (69.3 to 124.7) | 809.3 (621.1 to 1051.1) |  | 94.6 (59.3 to 141.1) | 109.9 (73.6 to 144.8) |  | 400.2 (245.5 to 591.3) | 0.0 (0.0 to 0.0) |  | 22.8 (7.4 to 46.0) | 0.0 (0.0 to 0.0) |  | 250690.0 (181217.1 to 335263.3) | NA |
| Albania | 10.8 (4.7 to 25.7) | 234.2 (129.0 to 443.5) |  | 2.6 (1.4 to 4.9) | 125.2 (66.1 to 224.4) |  | 19.4 (6.3 to 42.6) | 152.9 (53.9 to 330.6) |  | 3.2 (1.8 to 4.9) | 4.4 (2.5 to 7.2) |  | 11.5 (6.7 to 17.6) | 0.0 (0.0 to 0.0) |  | 0.7 (0.2 to 1.4) | 0.0 (0.0 to 0.0) |  | 8837.9 (6109.9 to 12054.5) | NA |
| Bosnia and Herzegovina | 2.0 (1.0 to 3.5) | 59.6 (31.8 to 92.7) |  | 0.8 (0.4 to 1.3) | 39.9 (19.7 to 65.3) |  | 2.4 (1.0 to 4.3) | 24.1 (9.7 to 43.5) |  | 2.6 (1.6 to 4.2) | 1.3 (1.0 to 1.7) |  | 10.3 (6.0 to 15.8) | 0.0 (0.0 to 0.0) |  | 0.7 (0.2 to 1.3) | 0.0 (0.0 to 0.0) |  | 7910.4 (5472.6 to 10783.5) | NA |
| Bulgaria | 3.0 (1.6 to 6.2) | 99.5 (60.3 to 173.5) |  | 2.7 (1.8 to 3.8) | 131.1 (90.6 to 188.9) |  | 17.8 (10.4 to 26.2) | 167.7 (98.5 to 242.8) |  | 5.6 (3.3 to 8.7) | 4.7 (3.3 to 6.8) |  | 22.1 (12.9 to 33.9) | 0.0 (0.0 to 0.0) |  | 1.4 (0.5 to 2.8) | 0.0 (0.0 to 0.0) |  | 16979.9 (11743.5 to 23160.1) | NA |
| Croatia | 8.6 (3.9 to 15.5) | 69.9 (46.4 to 97.2) |  | 1.0 (0.7 to 1.5) | 52.5 (33.4 to 75.0) |  | 2.0 (1.2 to 3.2) | 14.9 (9.4 to 23.8) |  | 3.6 (2.2 to 5.5) | 2.1 (1.5 to 3.5) |  | 12.6 (7.4 to 19.5) | 0.0 (0.0 to 0.0) |  | 0.9 (0.3 to 1.8) | 0.0 (0.0 to 0.0) |  | 13439.8 (10083.4 to 17576.4) | NA |
| Czechia | 15.3 (6.8 to 29.2) | 129.2 (81.8 to 194.6) |  | 2.8 (1.6 to 4.0) | 138.3 (81.2 to 204.9) |  | 4.6 (2.5 to 7.0) | 35.0 (19.0 to 52.1) |  | 7.4 (4.0 to 11.7) | 6.1 (4.4 to 9.1) |  | 44.2 (25.4 to 66.7) | 0.0 (0.0 to 0.0) |  | 2.8 (0.9 to 5.4) | 0.0 (0.0 to 0.0) |  | 31850.6 (22234.4 to 43418.1) | NA |
| Hungary | 10.5 (5.2 to 19.6) | 156.6 (96.3 to 228.5) |  | 2.6 (1.6 to 3.8) | 131.4 (78.8 to 195.0) |  | 3.1 (1.8 to 4.8) | 25.5 (14.6 to 39.3) |  | 8.6 (5.1 to 13.3) | 16.9 (8.4 to 25.1) |  | 30.9 (18.0 to 47.4) | 0.0 (0.0 to 0.0) |  | 2.0 (0.6 to 4) | 0.0 (0.0 to 0.0) |  | 23789.8 (16455.8 to 32435.8) | NA |
| Montenegro | 0.7 (0.3 to 1.5) | 12.1 (6.4 to 20.7) |  | 0.2 (0.1 to 0.3) | 8.8 (4.6 to 16.0) |  | 0.2 (0.1 to 0.4) | 1.7 (0.7 to 3.5) |  | 0.6 (0.3 to 0.9) | 0.4 (0.3 to 0.5) |  | 2.4 (1.4 to 3.7) | 0.0 (0.0 to 0.0) |  | 0.2 (0.0 to 0.3) | 0.0 (0.0 to 0.0) |  | 1872.7 (1294.9 to 2553.6) | NA |
| North Macedonia | 1.3 (0.6 to 2.4) | 36.5 (19.1 to 60.9) |  | 0.5 (0.3 to 0.9) | 25.0 (14.1 to 42.3) |  | 3.4 (1.8 to 6.1) | 32.9 (17.8 to 58.4) |  | 2.1 (1.2 to 3.2) | 1.3 (1.0 to 1.6) |  | 8.0 (4.7 to 12.3) | 0.0 (0.0 to 0.0) |  | 0.5 (0.2 to 1.0) | 0.0 (0.0 to 0.0) |  | 6166.5 (4264.0 to 8410.0) | NA |
| Poland | 36.2 (16.2 to 72.5) | 574.5 (400.6 to 767.8) |  | 8.7 (5.6 to 12.4) | 459.3 (297.2 to 623.8) |  | 15.1 (9.4 to 25.5) | 132.0 (87.8 to 217.6) |  | 16.1 (10.1 to 23.4) | 44.5 (24.4 to 62.1) |  | 143 (92.7 to 203.7) | 0.0 (0.0 to 0.0) |  | 5.8 (1.5 to 12.7) | 0.0 (0.0 to 0.0) |  | 24499.7 (18500.4 to 31784.1) | NA |
| Romania | 21.7 (12.6 to 38.8) | 539.6 (391.5 to 741.6) |  | 7.4 (4.8 to 10.6) | 366.0 (244.7 to 519.9) |  | 12.9 (8.3 to 18.9) | 114.1 (77.2 to 164.5) |  | 25.7 (16.1 to 39.0) | 15.2 (10.4 to 21.2) |  | 55.0 (31.3 to 84.2) | 0.0 (0.0 to 0.0) |  | 4.0 (1.2 to 7.9) | 0.0 (0.0 to 0.0) |  | 63419.7 (44841.6 to 84701.7) | NA |
| Serbia | 2.4 (0.9 to 5.8) | 54.9 (24.4 to 124.6) |  | 1.3 (0.6 to 2.2) | 66.9 (28.2 to 113.8) |  | 9.0 (3.9 to 15.5) | 80.7 (35.4 to 138.8) |  | 11.9 (7.4 to 18.4) | 8.8 (5.1 to 14.4) |  | 29.2 (16.6 to 44.8) | 0.0 (0.0 to 0.0) |  | 2.0 (0.6 to 4.1) | 0.0 (0.0 to 0.0) |  | 30045.9 (21100.5 to 40549.9) | NA |
| Slovakia | 11.2 (5.1 to 20.8) | 164.5 (99.2 to 245.1) |  | 1.3 (0.7 to 2.4) | 63.2 (32.9 to 119.7) |  | 2.9 (1.6 to 4.8) | 22.7 (13.1 to 36.2) |  | 5.1 (2.8 to 8.0) | 3.0 (2.3 to 4.0) |  | 20.6 (12.0 to 31.5) | 0.0 (0.0 to 0.0) |  | 1.4 (0.4 to 2.8) | 0.0 (0.0 to 0.0) |  | 16699.3 (11597.8 to 22860.4) | NA |
| Slovenia | 4.1 (1.6 to 7.1) | 23.4 (11.1 to 35.5) |  | 0.3 (0.2 to 0.4) | 13.2 (7.5 to 20.6) |  | 0.7 (0.4 to 1.1) | 5.0 (2.6 to 8.1) |  | 2.1 (1.2 to 3.1) | 1.1 (0.8 to 1.5) |  | 10.5 (6.2 to 16.0) | 0.0 (0.0 to 0.0) |  | 0.5 (0.2 to 0.9) | 0.0 (0.0 to 0.0) |  | 5177.9 (3546.1 to 7092.6) | NA |
| **Eastern Europe** | 353.5 (278.9 to 450.1) | 9660.7 (7706.5 to 11939.5) |  | 88.3 (62.5 to 108.3) | 4440.9 (3124.3 to 5559.4) |  | 155.3 (118.7 to 196.7) | 1475.4 (1140.3 to 1863.4) |  | 172.1 (95.8 to 274.2) | 276.9 (170.7 to 360.7) |  | 275.5 (130.5 to 470.5) | 0.0 (0.0 to 0.0) |  | 99.6 (41.2 to 176.2) | 0.0 (0.0 to 0.0) |  | 651961.0 (461580.3 to 884334.5) | NA |
| Belarus | 28.6 (15.0 to 53.3) | 549.5 (348.6 to 823.7) |  | 5.5 (3.2 to 9.8) | 264.8 (157.3 to 478.4) |  | 5.5 (3.1 to 9.3) | 42.9 (25.7 to 74.8) |  | 6.7 (3.5 to 11.0) | 8.9 (5.4 to 13.4) |  | 10.6 (4.7 to 18.5) | 0.0 (0.0 to 0.0) |  | 3.0 (1.0 to 5.8) | 0.0 (0.0 to 0.0) |  | 28411.6 (20119.5 to 38751.7) | NA |
| Estonia | 2.6 (1.2 to 5.2) | 32.6 (19.4 to 47.9) |  | 0.6 (0.4 to 0.9) | 29.8 (17.6 to 45.5) |  | 1.2 (0.8 to 2.0) | 9.5 (5.8 to 15.4) |  | 1.1 (0.6 to 1.6) | 5.4 (1.1 to 9.2) |  | 1.3 (0.6 to 2.3) | 0.0 (0.0 to 0.0) |  | 0.4 (0.1 to 0.7) | 0.0 (0.0 to 0.0) |  | 3515.6 (2488.3 to 4800.7) | NA |
| Latvia | 2.9 (1.4 to 5.5) | 69.7 (37.0 to 104.6) |  | 0.8 (0.5 to 1.2) | 39.8 (23.7 to 60.3) |  | 1.9 (1.1 to 3.3) | 17.0 (9.6 to 29.1) |  | 1.2 (0.6 to 1.9) | 1.1 (0.8 to 1.7) |  | 1.8 (0.8 to 3.1) | 0.0 (0.0 to 0.0) |  | 0.5 (0.2 to 1.0) | 0.0 (0.0 to 0.0) |  | 5009.3 (3421.5 to 6926.6) | NA |
| Lithuania | 2.6 (1.3 to 5.0) | 70.8 (40.3 to 113.0) |  | 1.4 (0.9 to 2.1) | 69.8 (43.0 to 106.0) |  | 2.6 (1.3 to 5.0) | 70.8 (40.3 to 113.0) |  | 1.6 (0.9 to 2.7) | 2.5 (1.4 to 4.1) |  | 2.9 (1.3 to 5.0) | 0.0 (0.0 to 0.0) |  | 0.9 (0.3 to 1.6) | 0.0 (0.0 to 0.0) |  | 7569.3 (5267.3 to 10390.4) | NA |
| Republic of Moldova | 3.0 (1.9 to 4.9) | 136.1 (87.8 to 205.9) |  | 0.8 (0.5 to 1.4) | 41.3 (25.3 to 67.3) |  | 8.2 (5.2 to 13.0) | 87.1 (56.3 to 134.0) |  | 2.3 (1.3 to 3.8) | 1.9 (1.4 to 2.6) |  | 3.2 (1.5 to 5.7) | 0.0 (0.0 to 0.0) |  | 0.9 (0.3 to 1.8) | 0.0 (0.0 to 0.0) |  | 8738.9 (6188.9 to 11922.6) | NA |
| Russian Federation | 235.1 (180.8 to 305.2) | 6115.7 (4792.4 to 7791.1) |  | 53.3 (29.7 to 67.6) | 2726.4 (1505.9 to 3535.4) |  | 82.8 (63.5 to 111.5) | 784.6 (595.7 to 1059.2) |  | 130.7 (72.0 to 208.2) | 214.3 (128.1 to 286.8) |  | 207.1 (98.5 to 353.5) | 0.0 (0.0 to 0.0) |  | 76.1 (31.8 to 134.3) | 0.0 (0.0 to 0.0) |  | 484976.3 (343390.8 to 657575.1) | NA |
| Ukraine | 78.6 (52.2 to 120.0) | 2686.4 (2014.9 to 3490.1) |  | 25.8 (18.3 to 34.8) | 1268.9 (902.0 to 1694.2) |  | 53.9 (36.3 to 73.7) | 519.5 (357.6 to 713.4) |  | 28.5 (15.3 to 45.3) | 42.7 (27.8 to 58.2) |  | 48.6 (23.1 to 82.9) | 0.0 (0.0 to 0.0) |  | 17.8 (7.4 to 31.4) | 0.0 (0.0 to 0.0) |  | 113739.9 (80530.4 to 154211.7) | NA |
| **High-income** | 2198.8 (1731.5 to 2774.7) | 16648.4 (13956.4 to 19744.8) |  | 372.3 (285.6 to 469.2) | 16596.8 (13354.1 to 19084.6) |  | 1071.7 (846.1 to 1325.8) | 7907.1 (6599.5 to 9277.9) |  | 1435.7 (1029.9 to 1938.0) | 3102.0 (2201.1 to 3724.3) |  | 2733.4 (1708.0 to 4072.2) | 0.0 (0.0 to 0.0) |  | 242.9 (105.6 to 451.6) | 0.0 (0.0 to 0.0) |  | 1464870.8 (1061645.7 to 1962025.3) | NA |
| **Australasia** | 15.4 (10.2 to 21.8) | 98.3 (66.1 to 133.7) |  | 8.9 (6.1 to 11.9) | 475.3 (332.8 to 625.2) |  | 15.4 (10.2 to 21.8) | 98.3 (66.1 to 133.7) |  | 8.2 (3.3 to 15.0) | 34.7 (19.0 to 49.0) |  | 34.7 (15.9 to 59.4) | 0.0 (0.0 to 0.0) |  | 8.3 (3.1 to 16.1) | 0.0 (0.0 to 0.0) |  | 16213.7 (11037.9 to 22616.0) | NA |
| Australia | 45.8 (21.1 to 89.5) | 381.0 (273.0 to 528.0) |  | 6.8 (4.6 to 9.5) | 385.2 (259.8 to 517.7) |  | 12.0 (7.5 to 17.6) | 79.1 (50.8 to 111.4) |  | 6.6 (2.5 to 12.3) | 31.8 (16.2 to 45.8) |  | 28.5 (13.0 to 49.1) | 0.0 (0.0 to 0.0) |  | 6.2 (2.1 to 12.6) | 0.0 (0.0 to 0.0) |  | 13805.2 (9239.6 to 19541.0) | NA |
| New Zealand | 16.2 (7.6 to 27.6) | 107.8 (76.3 to 146.0) |  | 2.1 (1.5 to 2.9) | 90.0 (65.2 to 119.5) |  | 3.4 (2.4 to 4.8) | 19.3 (13.7 to 26.8) |  | 1.6 (0.8 to 2.6) | 2.9 (2.3 to 3.8) |  | 6.2 (3.1 to 10.2) | 0.0 (0.0 to 0.0) |  | 2 (0.8 to 3.7) | 0.0 (0.0 to 0.0) |  | 2408.5 (1748.3 to 3229.1) | NA |
| **High-income Asia Pacific** | 372.8 (251.7 to 552.9) | 1934.3 (1534.1 to 2551.9) |  | 47.2 (34.3 to 59.4) | 2347.2 (1697.7 to 2859.7) |  | 194.1 (139.6 to 256.4) | 1292.4 (927.5 to 1658.1) |  | 488.2 (357.3 to 644.9) | 689.2 (510.5 to 836.4) |  | 1051.8 (662.6 to 1559.6) | 0.0 (0.0 to 0.0) |  | 82.9 (38.7 to 140.5) | 0.0 (0.0 to 0.0) |  | 849379.1 (629523.0 to 1109113.0) | NA |
| Brunei Darussalam | 0.4 (0.2 to 0.6) | 15.3 (8.8 to 23.9) |  | 0.5 (0.3 to 0.8) | 26.4 (16.4 to 39.8) |  | 1.7 (1.1 to 2.5) | 17.9 (11.3 to 26.8) |  | 2.2 (1.6 to 3.0) | 3.7 (2.6 to 5.3) |  | 3.8 (2.3 to 5.7) | 0.0 (0.0 to 0.0) |  | 0.3 (0.1 to 0.4) | 0.0 (0.0 to 0.0) |  | 3319.7 (2392.0 to 4405.0) | NA |
| Japan | 246.7 (145.1 to 404.2) | 1173.5 (919.5 to 1822.9) |  | 29.8 (21.2 to 39.2) | 1484.5 (1126.1 to 1696.8) |  | 109.5 (71.9 to 144.8) | 720.6 (488.0 to 868.9) |  | 355.2 (262.1 to 462.1) | 541.4 (386.1 to 677.5) |  | 750.8 (480.7 to 1103.2) | 0.0 (0.0 to 0.0) |  | 62.7 (29.9 to 104.5) | 0.0 (0.0 to 0.0) |  | 587013.4 (768016 to 436922.3) | NA |
| Republic of Korea | 108.6 (50.4 to 199.0) | 641.3 (384.5 to 1033.7) |  | 15.1 (7.9 to 23.0) | 749.3 (384.4 to 1175.7) |  | 80.5 (49.4 to 129.6) | 537.8 (325.4 to 831.8) |  | 115.7 (79.6 to 158.2) | 120.3 (78.5 to 177.1) |  | 262.3 (160.5 to 392.5) | 0.0 (0.0 to 0.0) |  | 17.6 (7.2 to 31.5) | 0.0 (0.0 to 0.0) |  | 587013.4 (436922.3 to 768016.0) | NA |
| Singapore | 17.2 (7.8 to 29.9) | 104.2 (59.7 to 149.7) |  | 1.7 (1.2 to 2.5) | 87.0 (57.6 to 123.5) |  | 2.3 (1.4 to 3.5) | 16.1 (10.2 to 23.9) |  | 15.0 (10.4 to 20.9) | 23.8 (12.8 to 35.2) |  | 34.9 (21.3 to 52.2) | 0.0 (0.0 to 0.0) |  | 2.4 (1.0 to 4.2) | 0.0 (0.0 to 0.0) |  | 30472.3 (21966.2 to 40390.3) | NA |
| **High-income North America** | 290.0 (206.0 to 390.8) | 5101.9 (4394.5 to 6033.7) |  | 139.5 (101.9 to 184.2) | 6470.4 (5359.5 to 7222.0) |  | 413.9 (317.4 to 531.8) | 3000.6 (2593.9 to 3440.9) |  | 1435.7 (1029.9 to 1938.0) | 3102.0 (2201.1  to 3724.3) |  | 1381.4 (883.4 to 2031.9) | 0.0 (0.0 to 0.0) |  | 66.6 (19.5 to 140.7) | 0.0 (0.0 to 0.0) |  | 61966.4 (43562.2 to 85715.2) | NA |
| Canada | 111.5 (61.6 to 169.9) | 503.8 (359.9 to 683.0) |  | 11.3 (7.7 to 15.9) | 553.4 (399.3 to 716.1) |  | 43.8 (29.9 to 62.2) | 279.9 (210.6 to 381.5) |  | 38.6 (22.6 to 60.2) | 114.2 (85.5 to 155.4) |  | 71.1 (38.1 to 111.2) | 0.0 (0.0 to 0.0) |  | 7.1 (2.2 to 14.5) | 0.0 (0.0 to 0.0) |  | 5541.5 (3729.9 to 7960.8) | NA |
| Greenland | 0.0 (0.0 to 0.0) | 0.2 (0.1 to 0.5) |  | 0.0 (0.0 to 0.0) | 0.6 (0.3 to 1.1) |  | 0.0 (0.0 to 0.0) | 0.2 (0.1 to 0.5) |  | 0.1 (0.0 to 0.1) | 0.1 (0.0 to 0.1) |  | 0.1 (0.1 to 0.2) | 0.0 (0.0 to 0.0) |  | 0.0 (0.0 to 0.0) | 0.0 (0.0 to 0.0) |  | 11.5 (7.7 to 16.5) | NA |
| United States of America | 178.6 (123.7 to 250.1) | 4597.2 (3985.5 to 5421.9) |  | 128.2 (93.4 to 171.5) | 5916.4 (4901.9 to 6620.6) |  | 370.1 (282.0 to 481.5) | 2720.5 (2334.7 to 3102.8) |  | 388.3 (273.3 to 538.2) | 1220.4 (916.9 to 1572.3) |  | 1310.2 (841.2 to 1931.8) | 0.0 (0.0 to 0.0) |  | 59.5 (17.1 to 126.7) | 0.0 (0.0 to 0.0) |  | 56412.4 (39754.6 to 77776.3) | NA |
| **Southern Latin America** | 85.2 (60.3 to 116.5) | 3292.5 (2582.1 to 4133.2) |  | 42.3 (29.5 to 59.3) | 2140.7 (1599.8 to 2764.4) |  | 87.4 (60.2 to 120.9) | 928.9 (708.1 to 1214.3) |  | 408.7 (284.3 to 551.4) | 392.1 (275.4 to 530.6) |  | 115.2 (54.3 to 190.0) | 0.0 (0.0 to 0.0) |  | 21.7 (7.0 to 42.8) | 0.0 (0.0 to 0.0) |  | 120682.3 (83117.4 to 166113.2) | NA |
| Argentina | 53.1 (36.2 to 77.8) | 2483.2 (1923.0 to 3166.9) |  | 31.2 (20.9 to 45.2) | 1571.8 (1148.5 to 2101.2) |  | 61.5 (39.9 to 88.6) | 684.8 (493.0 to 915.1) |  | 293.9 (200.8 to 400.0) | 232.2 (164.7 to 330.5) |  | 82.2 (38.7 to 135.6) | 0.0 (0.0 to 0.0) |  | 15.5 (5 to 30.6) | 0.0 (0.0 to 0.0) |  | 86147.3 (59323.4 to 118583.1) | NA |
| Chile | 29.5 (16.5 to 50.7) | 711.3 (503.0 to 970.7) |  | 9.6 (6.4 to 13.9) | 493.5 (338.7 to 702.3) |  | 20.8 (13.5 to 30.5) | 190.6 (132.1 to 266.3) |  | 96.3 (67.9 to 130.0) | 137.5 (75.5 to 205.0) |  | 27.5 (13.0 to 45.4) | 0.0 (0.0 to 0.0) |  | 5.2 (1.7 to 10.2) | 0.0 (0.0 to 0.0) |  | 28800.9 (19846.5 to 39637.6) | NA |
| Uruguay | 2.5 (1.5 to 4.1) | 97.9 (64.4 to 139.8) |  | 1.5 (0.9 to 2.3) | 75.3 (48.6 to 113.8) |  | 5.0 (2.9 to 7.8) | 53.4 (31.5 to 79.2) |  | 18.5 (13.2 to 25.5) | 22.3 (14.1 to 32.3) |  | 5.5 (2.6 to 9.0) | 0.0 (0.0 to 0.0) |  | 1.0 (0.3 to 2.0) | 0.0 (0.0 to 0.0) |  | 5728.0 (3943.3 to 7884.2) | NA |
| **Western Europe** | 1388.8 (1054.4 to 1749.0) | 5830.8 (4563.8 to 7111.0) |  | 134.5 (99.4 to 173.5) | 5163.2 (3980.0 to 6123.1) |  | 361.0 (269.1 to 467.2) | 2586.9 (2002.2 to 3123.7) |  | 103.6 (64.5 to 161.2) | 651.3 (382.3 to 826.5) |  | 150.2 (61.7 to 320.9) | 0.0 (0.0 to 0.0) |  | 63.4 (22.6 to 130.6) | 0.0 (0.0 to 0.0) |  | 416629.4 (287371.9 to 572513.7) | NA |
| Andorra | 0.2 (0.1 to 0.5) | 1.0 (0.5 to 1.8) |  | 0.0 (0.0 to 0.0) | 1.0 (0.5 to 1.6) |  | 0.1 (0.0 to 0.1) | 0.3 (0.2 to 0.6) |  | 0.0 (0.0 to 0.0) | 0.1 (0.0 to 0.1) |  | 0.0 (0.0 to 0.0) | 0.0 (0.0 to 0.0) |  | 0.0 (0.0 to 0.0) | 0.0 (0.0 to 0.0) |  | 48.6 (33.2 to 68.6) | NA |
| Austria | 18.1 (9.3 to 28.0) | 80.5 (56.0 to 113.7) |  | 1.9 (1.3 to 2.6) | 93.0 (67.9 to 118.5) |  | 4.1 (2.8 to 5.8) | 26.8 (19.6 to 36.2) |  | 2.4 (1.1 to 4.1) | 13.2 (5.9 to 20.4) |  | 3.1 (0.7 to 6.7) | 0.0 (0.0 to 0.0) |  | 1.2 (0.2 to 2.7) | 0.0 (0.0 to 0.0) |  | 15388 (11153.6 to 20734.4) | NA |
| Belgium | 27.4 (13.4 to 45.6) | 124.1 (84.1 to 182.2) |  | 3.3 (2.2 to 4.7) | 159.4 (109.8 to 213.6) |  | 11.0 (7.4 to 16.5) | 71.8 (51.3 to 102.4) |  | 0.6 (0.1 to 1.3) | 9.8 (6.7 to 13.6) |  | 2.8 (0.7 to 7.2) | 0.0 (0.0 to 0.0) |  | 1.3 (0.2 to 3) | 0.0 (0.0 to 0.0) |  | 11470.0 (7804.2 to 16069.1) | NA |
| Cyprus | 3.8 (1.8 to 6.8) | 17.6 (10.7 to 29.0) |  | 0.3 (0.1 to 0.4) | 13.8 (7.5 to 22.5) |  | 1.9 (0.9 to 3.0) | 13.1 (5.8 to 21.1) |  | 0.1 (0.0 to 0.2) | 0.7 (0.5 to 1.0) |  | 0.3 (0.1 to 0.9) | 0.0 (0.0 to 0.0) |  | 0.1 (0.0 to 0.3) | 0.0 (0.0 to 0.0) |  | 1014.7 (682.1 to 1424.9) | NA |
| Denmark | 18.1 (9.4 to 27.6) | 77.8 (47.1 to 108.6) |  | 1.3 (0.9 to 1.9) | 76.8 (51.5 to 106.5) |  | 6.9 (4.0 to 9.9) | 37.2 (21.7 to 52.0) |  | 0.3 (0.1 to 0.7) | 2.6 (2.0 to 3.9) |  | 1.4 (0.3 to 3.7) | 0.0 (0.0 to 0.0) |  | 0.6 (0.1 to 1.5) | 0.0 (0.0 to 0.0) |  | 5532.5 (3754.1 to 7660.9) | NA |
| Finland | 15.7 (8.1 to 25.6) | 75.2 (47.1 to 104.0) |  | 1.2 (0.8 to 1.7) | 52.2 (36.1 to 71.2) |  | 3.0 (1.9 to 4.3) | 16.0 (10.2 to 23.2) |  | 0.5 (0.2 to 1.0) | 2.1 (1.7 to 3.1) |  | 1.3 (0.3 to 3.2) | 0.0 (0.0 to 0.0) |  | 0.6 (0.1 to 1.3) | 0.0 (0.0 to 0.0) |  | 5075.9 (3396.1 to 7047.6) | NA |
| France | 218.9 (115.4 to 330.5) | 970.5 (689.6 to 1295.1) |  | 18.5 (12.4 to 25.5) | 896.8 (642.7 to 1174.4) |  | 118.5 (80.0 to 164.7) | 778.9 (566.6 to 1022.4) |  | 9.2 (3.4 to 17.2) | 120.9 (60.3 to 171.3) |  | 17.2 (4.0 to 43.0) | 0.0 (0.0 to 0.0) |  | 7.6 (1.3 to 18.9) | 0.0 (0.0 to 0.0) |  | 65897.4 (45038.4 to 93135.6) | NA |
| Germany | 249.1 (165.2 to 358.1) | 955.7 (701.2 to 1259.9) |  | 43.3 (26.7 to 66.2) | 805.3 (603.4 to 1054.6) |  | 28.4 (19.3 to 40.6) | 313.4 (228.5 to 416.0) |  | 4.2 (1.1 to 9.2) | 90.5 (52.8 to 126.8) |  | 18.8 (4.6 to 47.8) | 0.0 (0.0 to 0.0) |  | 7.8 (1.6 to 19.1) | 0.0 (0.0 to 0.0) |  | 81344.1 (55229.7 to 113803.1) | NA |
| Greece | 31.1 (14.8 to 54.3) | 164.5 (115.0 to 227.6) |  | 2.7 (1.7 to 3.9) | 129.1 (90.3 to 175.2) |  | 13.1 (8.5 to 19.7) | 89.9 (64.4 to 130.8) |  | 0.5 (0.1 to 1.1) | 13.7 (5.6 to 20.6) |  | 2.1 (0.5 to 5.3) | 0.0 (0.0 to 0.0) |  | 0.9 (0.2 to 2.3) | 0.0 (0.0 to 0.0) |  | 8161.7 (5578.3 to 11535.5) | NA |
| Iceland | 0.7 (0.4 to 1.1) | 3.3 (1.9 to 5.4) |  | 0.1 (0.0 to 0.1) | 4.0 (2.3 to 6.2) |  | 0.2 (0.1 to 0.3) | 1.6 (0.8 to 2.6) |  | 0.0 (0.0 to 0.0) | 0.3 (0.2 to 0.5) |  | 0.1 (0.0 to 0.3) | 0.0 (0.0 to 0.0) |  | 0.0 (0.0 to 0.1) | 0.0 (0.0 to 0.0) |  | 337.4 (228.6 to 470.5) | NA |
| Ireland | 14.7 (8.4 to 22.3) | 59.2 (36.7 to 84.4) |  | 1.0 (0.6 to 1.5) | 50.1 (33.0 to 72.8) |  | 2.4 (1.3 to 3.7) | 15.3 (8.3 to 23.3) |  | 0.4 (0.1 to 0.8) | 3.2 (2.3 to 4.9) |  | 1.5 (0.3 to 3.7) | 0.0 (0.0 to 0.0) |  | 0.7 (0.1 to 1.6) | 0.0 (0.0 to 0.0) |  | 5730.6 (3916.7 to 8099.4) | NA |
| Israel | 21.3 (9.4 to 44.4) | 149.1 (97.7 to 236.9) |  | 4.0 (2.6 to 5.9) | 199.8 (139.0 to 278.0) |  | 10.3 (5.4 to 15.1) | 74.0 (39.4 to 103.6) |  | 1.1 (0.4 to 2.5) | 16.8 (11.9 to 23.4) |  | 4.4 (1.0 to 11.1) | 0.0 (0.0 to 0.0) |  | 2.0 (0.3 to 4.9) | 0.0 (0.0 to 0.0) |  | 17111.9 (11690.2 to 24198.9) | NA |
| Italy | 201.8 (136.5 to 277.3) | 830.5 (616.3 to 1031.5) |  | 12.0 (7.6 to 16.2) | 581.5 (370.2 to 722.4) |  | 30.7 (22.1 to 42.8) | 196.5 (158.4 to 276.8) |  | 58.7 (39.4 to 85.6) | 95.3 (49.4 to 124.8) |  | 50.8 (25.5 to 85.6) | 0.0 (0.0 to 0.0) |  | 14.2 (5.7 to 25.9) | 0.0 (0.0 to 0.0) |  | 56850.7 (40982.0 to 76313.0) | NA |
| Luxembourg | 1.0 (0.6 to 1.6) | 4.9 (2.8 to 8.1) |  | 0.1 (0.1 to 0.2) | 6.9 (3.9 to 11.1) |  | 1.0 (0.6 to 1.4) | 7.2 (3.9 to 11.5) |  | 0.0 (0.0 to 0.1) | 0.5 (0.3 to 0.8) |  | 0.2 (0.0 to 0.4) | 0.0 (0.0 to 0.0) |  | 0.1 (0.0 to 0.2) | 0.0 (0.0 to 0.0) |  | 595.2 (395.2 to 840.5) | NA |
| Malta | 6.3 (3.9 to 9.6) | 1.1 (0.5 to 1.9) |  | 6.5 (4.1 to 9.7) | 0.1 (0.1 to 0.2) |  | 0.2 (0.1 to 0.3) | 1.3 (0.8 to 1.9) |  | 0.0 (0.0 to 0.1) | 0.9 (0.4 to 1.4) |  | 0.1 (0.0 to 0.3) | 0.0 (0.0 to 0.0) |  | 0.0 (0.0 to 0.1) | 0.0 (0.0 to 0.0) |  | 344.7 (235.1 to 484.3) | NA |
| Monaco | 0.1 (0.0 to 0.3) | 0.6 (0.1 to 1.1) |  | 0.0 (0.0 to 0.0) | 0.7 (0.3 to 1.2) |  | 0.1 (0.1 to 0.2) | 0.8 (0.4 to 1.4) |  | 0.0 (0.0 to 0.0) | 0.0 (0.0 to 0.0) |  | 0.0 (0.0 to 0.0) | 0.0 (0.0 to 0.0) |  | 0.0 (0.0 to 0.0) | 0.0 (0.0 to 0.0) |  | 29.3 (20.0 to 41.4) | NA |
| Netherlands | 56.1 (35.2 to 81.4) | 223.9 (150.9 to 304.6) |  | 3.9 (2.6 to 5.6) | 192.4 (133.8 to 262.5) |  | 23.3 (14.5 to 34.5) | 147.2 (95.4 to 205.5) |  | 2.1 (0.7 to 4.1) | 47.1 (22.8 to 66.3) |  | 4.1 (1.0 to 10.4) | 0.0 (0.0 to 0.0) |  | 1.8 (0.3 to 4.5) | 0.0 (0.0 to 0.0) |  | 15899.7 (10863.5 to 22479.9) | NA |
| Norway | 11.0 (6.2 to 15.5) | 48.4 (32.4 to 60.4) |  | 1.0 (0.7 to 1.3) | 54.1 (41.0 to 64.2) |  | 2.8 (2.0 to 3.7) | 14.8 (10.5 to 18.4) |  | 2.8 (1.5 to 4.7) | 3.4 (2.5 to 4.2) |  | 4.3 (1.8 to 7.7) | 0.0 (0.0 to 0.0) |  | 4.1 (2.0 to 6.9) | 0.0 (0.0 to 0.0) |  | 7530.4 (5190.1 to 10248.6) | NA |
| Portugal | 18.0 (8.2 to 32.0) | 103.9 (68.2 to 148.6) |  | 2.1 (1.3 to 3.1) | 99.5 (62.7 to 145.8) |  | 7.0 (4.5 to 10.8) | 47.7 (33.2 to 69.9) |  | 1.0 (0.4 to 1.9) | 28.4 (10.0 to 48.6) |  | 2.1 (0.5 to 5.0) | 0.0 (0.0 to 0.0) |  | 0.8 (0.1 to 2.0) | 0.0 (0.0 to 0.0) |  | 6471 (4474.8 to 8899.4) | NA |
| San Marino | 0.4 (0.2 to 0.6) | 1.6 (0.9 to 2.5) |  | 0.0 (0.0 to 0.0) | 0.3 (0.2 to 0.6) |  | 0.1 (0.1 to 0.2) | 0.8 (0.4 to 1.3) |  | 0.0 (0.0 to 0.0) | 0.0 (0.0 to 0.1) |  | 0.0 (0.0 to 0.0) | 0.0 (0.0 to 0.0) |  | 0.0 (0.0 to 0.0) | 0.0 (0.0 to 0.0) |  | 29.4 (20.1 to 41.5) | NA |
| Spain | 171.2 (105.8 to 252.9) | 700.3 (500.3 to 936.1) |  | 10.5 (6.6 to 14.9) | 515.5 (328.8 to 698.0) |  | 49.8 (33.1 to 69.8) | 318.5 (229.2 to 425.5) |  | 49.8 (33.1 to 69.8) | 318.5 (229.2 to 425.5) |  | 9.5 (2.2 to 23.7) | 0.0 (0.0 to 0.0) |  | 4.2 (0.7 to 10.4) | 0.0 (0.0 to 0.0) |  | 36294.7 (24807.8 to 51294.8) | NA |
| Sweden | 40.1 (23.8 to 57.0) | 160.5 (101.0 to 218.0) |  | 3.7 (2.6 to 4.9) | 172.9 (122.8 to 221.7) |  | 11.7 (7.5 to 16.0) | 70.9 (45.3 to 94.7) |  | 11.7 (7.5 to 16.0) | 70.9 (45.3 to 94.7) |  | 5.4 (1.7 to 10.7) | 0.0 (0.0 to 0.0) |  | 2.8 (1.0 to 5.5) | 0.0 (0.0 to 0.0) |  | 18587.1 (12721.4 to 25771.7) | NA |
| Switzerland | 25.1 (15.0 to 37.6) | 103.1 (70.2 to 143.4) |  | 1.8 (1.2 to 2.5) | 87.0 (61.7 to 117.7) |  | 8.6 (5.5 to 12.2) | 55.6 (36.6 to 73.6) |  | 0.6 (0.1 to 1.3) | 4.6 (3.3 to 6.5) |  | 2.1 (0.5 to 5.4) | 0.0 (0.0 to 0.0) |  | 1.1 (0.2 to 2.5) | 0.0 (0.0 to 0.0) |  | 8058.7 (5388.4 to 11240.3) | NA |
| United Kingdom | 242.7 (173.9 to 324.7) | 963.2 (746.1 to 1149.1) |  | 21.6 (15.7 to 28.4) | 960.2 (775.6 to 1115.9) |  | 25.5 (17.9 to 34.1) | 285.1 (212.0 to 336.8) |  | 11.9 (6.1 to 21.4) | 124.6 (73.0 to 156.3) |  | 18.4 (4.9 to 46.0) | 0.0 (0.0 to 0.0) |  | 11.3 (3.6 to 23.7) | 0.0 (0.0 to 0.0) |  | 48462.5 (34087 to 66338.3) | NA |
| **Latin America and Caribbean** | 1247.5 (932.0 to 1627.2) | 58216.0 (44143.8 to 75048.8) |  | 551.4 (403.1 to 716.2) | 24414.7 (18169.6 to 31431.5) |  | 1615.9 (1195.6 to 2114.3) | 20607.0 (14712.7 to 28036.7) |  | 1476.6 (994.6 to 2100.3) | 6049.1 (4072.0 to 8180.7) |  | 2972.2 (1825.2 to 4516.9) | 0.0 (0.0 to 0.0) |  | 1270.8 (750.0 to 1948.2) | 0.0 (0.0 to 0.0) |  | 825264.5 (577603.3to 1124847.6) | NA |
| **Andean Latin America** | 136.5 (74.2 to 221.3) | 7575.1 (4151.2 to 11991.0) |  | 58.1 (29.0 to 96.7) | 2825.0 (1428.2 to 4730.9) |  | 249.8 (127.0 to 425.7) | 3198.5 (1651.0 to 5312.7) |  | 181.3 (115.9 to 268.2) | 465.2 (289.5 to 669.5) |  | 293.3 (171.6 to 440.1) | 0.0 (0.0 to 0.0) |  | 87.9 (45.3 to 141.8) | 0.0 (0.0 to 0.0) |  | 71114.8 (51014.3 to 95537.4) | NA |
| Bolivia (Plurinational State of) | 49.9 (23.3 to 85.5) | 3021.0 (1409.6 to 5147.1) |  | 19.3 (8.9 to 35.5) | 934.5 (430.7 to 1690.3) |  | 92.1 (41.9 to 162.7) | 1366.3 (648.1 to 2283.4) |  | 55.2 (35.8 to 80.6) | 123.3 (71.0 to 186.7) |  | 70.6 (39.8 to 108.5) | 0.0 (0.0 to 0.0) |  | 20.9 (10.7 to 34.0) | 0.0 (0.0 to 0.0) |  | 17411.6 (11762.1 to 24282.7) | NA |
| Ecuador | 34.8 (18.6 to 55.2) | 1885.2 (1007.2 to 2965.2) |  | 13.4 (6.8 to 22.8) | 645.8 (325.8 to 1078.8) |  | 42.2 (20.6 to 77.9) | 498.8 (244.9 to 898.9) |  | 50.0 (34.1 to 72.0) | 290.5 (152.2 to 458.9) |  | 77.5 (49.2 to 115.8) | 0.0 (0.0 to 0.0) |  | 24.0 (12.5 to 39.0) | 0.0 (0.0 to 0.0) |  | 17847.5 (14094.5 to 22048.4) | NA |
| Peru | 51.8 (20.6 to 102.1) | 2668.8 (1082.4 to 5386.1) |  | 25.4 (9.3 to 51.6) | 1244.7 (463.1 to 2491.1) |  | 115.5 (43.6 to 242.1) | 1333.3 (507.7 to 2682.7) |  | 76.0 (43.5 to 118.4) | 51.4 (28.4 to 85.9) |  | 145.2 (81.9 to 223.2) | 0.0 (0.0 to 0.0) |  | 43.0 (22.0 to 70.0) | 0.0 (0.0 to 0.0) |  | 35855.7 (24273.6 to 50010.8) | NA |
| **Caribbean** | 101.2 (49.7 to 192.7) | 5728.1 (2597.5 to 11466.4) |  | 44.5 (22.5 to 85.0) | 2213.9 (1130.6 to 4296.0) |  | 328.4 (109.7 to 661.8) | 5907.3 (1707.5 to 12590.3) |  | 256.9 (179.3 to 349.3) | 449.0 (280.9 to 732.7) |  | 179.1 (99.5 to 284.4) | 0.0 (0.0 to 0.0) |  | 59.2 (30.1 to 95.4) | 0.0 (0.0 to 0.0) |  | 55037.2 (37596.4 to 76790.4) | NA |
| Antigua and Barbuda | 0.1 (0.0 to 0.1) | 3.3 (1.9 to 5.8) |  | 0.1 (0.0 to 0.1) | 3.0 (1.6 to 5.1) |  | 0.3 (0.1 to 0.4) | 2.8 (1.6 to 4.6) |  | 0.4 (0.2 to 0.5) | 0.3 (0.2 to 0.5) |  | 0.2 (0.1 to 0.4) | 0.0 (0.0 to 0.0) |  | 0.1 (0.0 to 0.1) | 0.0 (0.0 to 0.0) |  | 71.0 (48.5 to 99.0) | NA |
| Bahamas | 0.2 (0.1 to 0.3) | 9.2 (5.7 to 14.1) |  | 0.2 (0.1 to 0.3) | 8.8 (5.6 to 13.5) |  | 0.2 (0.2 to 0.4) | 3.0 (2.1 to 4.4) |  | 1.3 (0.9 to 1.8) | 3.0 (1.8 to 4.6) |  | 1.0 (0.6 to 1.6) | 0.0 (0.0 to 0.0) |  | 0.3 (0.2 to 0.5) | 0.0 (0.0 to 0.0) |  | 303.4 (207.6 to 422.5) | NA |
| Barbados | 0.1 (0.1 to 0.2) | 4.6 (2.8 to 7.1) |  | 0.2 (0.1 to 0.2) | 7.7 (4.9 to 11.7) |  | 0.3 (0.2 to 0.5) | 3.8 (2.5 to 5.8) |  | 0.9 (0.6 to 1.2) | 1.1 (0.7 to 1.7) |  | 0.7 (0.4 to 1.0) | 0.0 (0.0 to 0.0) |  | 0.2 (0.1 to 0.4) | 0.0 (0.0 to 0.0) |  | 203.3 (139.0 to 283.5) | NA |
| Belize | 0.4 (0.2 to 0.5) | 21.1 (14.5 to 29.0) |  | 0.1 (0.1 to 0.1) | 4.6 (2.9 to 7.0) |  | 1.3 (0.9 to 1.8) | 17.4 (12.3 to 24.6) |  | 2.6 (1.8 to 3.6) | 2.9 (1.8 to 4.3) |  | 1.7 (0.9 to 2.7) | 0.0 (0.0 to 0.0) |  | 0.6 (0.3 to 0.9) | 0.0 (0.0 to 0.0) |  | 522.8 (357.2 to 729.2) | NA |
| Bermuda | 0.1 (0.0 to 0.2) | 1.1 (0.7 to 1.7) |  | 0.0 (0.0 to 0.0) | 1.5 (1.0 to 2.2) |  | 0.1 (0.1 to 0.1) | 0.6 (0.4 to 0.9) |  | 0.1 (0.1 to 0.2) | 0.5 (0.3 to 0.7) |  | 0.1 (0.1 to 0.2) | 0.0 (0.0 to 0.0) |  | 0.0 (0.0 to 0.1) | 0.0 (0.0 to 0.0) |  | 36.5 (24.9 to 50.9) | NA |
| Cuba | 11.6 (7.1 to 18.4) | 358.2 (246.3 to 495.7) |  | 4.2 (2.9 to 5.9) | 211.3 (143.0 to 296.5) |  | 6.2 (4.1 to 8.7) | 59.4 (41.0 to 82.7) |  | 28.9 (19.7 to 40.4) | 14.8 (9.9 to 21.0) |  | 25.4 (14.2 to 40.3) | 0.0 (0.0 to 0.0) |  | 8.4 (4.3 to 13.5) | 0.0 (0.0 to 0.0) |  | 7826.7 (5354.7 to 10910.6) | NA |
| Dominica | 0.1 (0.1 to 0.2) | 7.4 (4.4 to 11.7) |  | 0.0 (0.0 to 0.1) | 2.2 (1.1 to 3.9) |  | 0.2 (0.1 to 0.4) | 2.9 (1.6 to 4.9) |  | 0.3 (0.2 to 0.4) | 0.9 (0.6 to 1.5) |  | 0.2 (0.1 to 0.3) | 0.0 (0.0 to 0.0) |  | 0.1 (0.0 to 0.1) | 0.0 (0.0 to 0.0) |  | 59 (40.3 to 82.2) | NA |
| Dominican Republic | 14.1 (6.7 to 26.3) | 824.4 (390.2 to 1524.1) |  | 9.1 (4.4 to 16.9) | 452.1 (223.9 to 850.1) |  | 49.5 (22.5 to 95.6) | 644.6 (296.1 to 1174.3) |  | 84.6 (59.8 to 115.8) | 177.7 (94.5 to 300.5) |  | 49.6 (27.5 to 78.8) | 0.0 (0.0 to 0.0) |  | 16.3 (8.3 to 26.3) | 0.0 (0.0 to 0.0) |  | 15247.9 (10412.4 to 21286.5) | NA |
| Grenada | 0.0 (0.0 to 0.1) | 2.6 (1.5 to 4.2) |  | 0.1 (0.0 to 0.1) | 2.5 (1.5 to 4.2) |  | 0.2 (0.1 to 0.4) | 3.1 (1.9 to 4.7) |  | 0.6 (0.4 to 0.7) | 1.4 (0.8 to 2.2) |  | 0.3 (0.2 to 0.5) | 0.0 (0.0 to 0.0) |  | 0.1 (0.1 to 0.2) | 0.0 (0.0 to 0.0) |  | 97.7 (66.7 to 136.2) | NA |
| Guyana | 1.1 (0.6 to 2.0) | 17.2 (9.7 to 32.5) |  | 0.2 (0.1, 0.3) | 7.9 (4.1 to 14.5) |  | 1.1 (0.7 to 2.0) | 17.2 (9.7 to 32.5) |  | 5.2 (3.6 to 7.1) | 5.3 (3.0 to 8.1) |  | 3.2 (1.8 to 5.1) | 0.0 (0.0 to 0.0) |  | 1.1 (0.5 to 1.7) | 0.0 (0.0 to 0.0) |  | 988.7 (675.6 to 1378.9) | NA |
| Haiti | 63.8 (16.9 to 153.2) | 3965.0 (1053.6 to 9465.2) |  | 25.1 (7.1 to 64.4) | 1242.0 (347.6 to 3294.7) |  | 247.6 (37.2 to 584.3) | 4833.1 (758.8 to 11460.7) |  | 97.6 (64.6 to 135.9) | 197.4 (83.7 to 494.6) |  | 69.2 (38.4 to 110.0) | 0.0 (0.0 to 0.0) |  | 22.9 (11.7 to 37.0) | 0.0 (0.0 to 0.0) |  | 21243.7 (14501.8 to 29648.9) | NA |
| Jamaica | 1.9 (1.1 to 2.8) | 109.1 (70.0 to 161.3) |  | 1.4 (0.8 to 2.1) | 74.6 (47.2 to 111.4) |  | 2.9 (1.7 to 4.7) | 38.6 (22.9 to 61.0) |  | 8.7 (6.0 to 11.9) | 8.7 (5.1 to 14.9) |  | 8.4 (4.7 to 13.3) | 0.0 (0.0 to 0.0) |  | 2.8 (1.4 to 4.5) | 0.0 (0.0 to 0.0) |  | 2575.7 (1761 to 3589.9) | NA |
| Puerto Rico | 2.4 (1.2 to 4.5) | 47.0 (32.8 to 64.8) |  | 0.6 (0.4 to 0.8) | 28.1 (18.7 to 41.1) |  | 2.9 (1.7 to 4.2) | 24.9 (15.5 to 34.9) |  | 8.6 (6.0 to 11.8) | 4.9 (3.2 to 7.8) |  | 6.0 (3.3 to 9.4) | 0.0 (0.0 to 0.0) |  | 2.0 (1.0 to 3.2) | 0.0 (0.0 to 0.0) |  | 1831.2 (1252.2 to 2553.6) | NA |
| Saint Kitts and Nevis | 0.0 (0.0 to 0.1) | 2.0 (0.6 to 3.4) |  | 0.0 (0.0 to0.0) | 0.5 (0.2 to 0.8) |  | 0.2 (0.1 to 0.2) | 1.5 (0.6 to 2.4) |  | 0.1 (0.1 to 0.2) | 0.2 (0.1 to 0.3) |  | 0.2 (0.1 to 0.3) | 0.0 (0.0 to 0.0) |  | 0.1 (0.0 to 0.1) | 0.0 (0.0 to 0.0) |  | 49.9 (34.1 to 69.5) | NA |
| Saint Lucia | 0.1 (0.1 to 0.2) | 6.1 (3.8 to 9.4) |  | 0.1 (0.0 to 0.1) | 3.7 (2.2 to 5.8) |  | 0.3 (0.1 to 0.4) | 2.8 (1.7 to 4.3) |  | 0.6 (0.4 to 0.9) | 1.3 (0.7 to 2.1) |  | 0.4 (0.2 to 0.7) | 0.0 (0.0 to 0.0) |  | 0.1 (0.1 to 0.2) | 0.0 (0.0 to 0.0) |  | 129.0 (88.2 to 179.7) | NA |
| Saint Vincent and the Grenadines | 0.1 (0.0 to 0.1) | 4.2 (2.6 to 6.6) |  | 0.1 (0.0 to 0.1) | 2.8 (1.7 to 4.4) |  | 0.2 (0.2 to 0.4) | 3.4 (2.1 to 5.1) |  | 0.5 (0.4 to 0.7) | 1.4 (0.8 to 2.2) |  | 0.4 (0.2 to 0.6) | 0.0 (0.0 to 0.0) |  | 0.1 (0.1 to 0.2) | 0.0 (0.0 to 0.0) |  | 107.9 (73.7 to 150.3) | NA |
| Suriname | 0.4 (0.2 to 0.6) | 22.8 (13.2 to 37.9) |  | 0.4 (0.2 to 0.6) | 18.9 (11.3 to 30.9) |  | 1.8 (1.0 to 2.9) | 25.1 (15.1 to 39.5) |  | 2.6 (1.7 to 3.6) | 5.4 (3.3 to 8.6) |  | 2.1 (1.2 to 3.3) | 0.0 (0.0 to 0.0) |  | 0.7 (0.3 to 1.1) | 0.0 (0.0 to 0.0) |  | 636.7 (435.3 to 887.5) | NA |
| Trinidad and Tobago | 1.5 (0.9 to 2.2) | 83.9 (52.5 to 126.3) |  | 1.3 (0.8 to 2.0) | 64.2 (40.0 to 96.3) |  | 1.8 (1.1 to 2.9) | 21.9 (13.7 to 34) |  | 4.1 (2.8 to 5.7) | 5.8 (3.3 to 9.3) |  | 3.7 (2.1 to 5.9) | 0.0 (0.0 to 0.0) |  | 1.2 (0.6 to 2.0) | 0.0 (0.0 to 0.0) |  | 1152.7 (788.6 to 1605.7) | NA |
| United States Virgin Islands | 0.0 (0.0 to 0.0) | 1.1 (0.5 to 2.3) |  | 0.0 (0.0 to 0.1) | 2.5 (1.3 to 4.9) |  | 0.1 (0.0 to 0.2) | 1.0 (0.4 to 2.1) |  | 0.4 (0.3 to 0.6) | 0.5 (0.3 to 1.0) |  | 0.3 (0.2 to 0.5) | 0.0 (0.0 to 0.0) |  | 0.1 (0.0 to 0.2) | 0.0 (0.0 to 0.0) |  | 89.2 (61.0 to 124.4) | NA |
| **Central Latin America** | 660.2 (469.7 to 905.7) | 29657.7 (21572.1 to 39259.0) |  | 227.0 (157.6 to 312.7) | 10441.5 (7373.6 to 14064.4) |  | 579.7 (404.4 to 811.8) | 6640.1 (4678.1 to 9150.4) |  | 906.8 (617.8 to 1265.6) | 3634.7 (2226.7 to 5112.9) |  | 2027.0 (1260.4 to 3000.4) | 0.0 (0.0 to 0.0) |  | 876.0 (525.5 to 1323.7) | 0.0 (0.0 to 0.0) |  | 623351.8 (439839.8 to 847613.0) | NA |
| Colombia | 131.5 (80.2 to 206.7) | 5420.4 (3621.3 to 7994.7) |  | 42.4 (25.5 to 65.2) | 2106.9 (1267.1 to 3235.6) |  | 108.4 (62.8 to 172.3) | 1119.1 (663.8 to 1749.6) |  | 185.7 (126.9 to 266.3) | 824.6 (440.7 to 1318.4) |  | 349.3 (213.0 to 522.3) | 0.0 (0.0 to 0.0) |  | 120.2 (71.7 to 184.2) | 0.0 (0.0 to 0.0) |  | 104724.4 (71171.9 to 146273.4) | NA |
| Costa Rica | 6.3 (3.7 to 10.8) | 208.9 (137.4 to 311.7) |  | 2.8 (1.7 to 4.1) | 140.9 (87.6 to 206.5) |  | 2.9 (1.7 to 4.5) | 27.0 (16.9 to 41.1) |  | 11.2 (7.2 to 16.5) | 27.3 (16.0 to 47.9) |  | 31.0 (18.9 to 46.4) | 0.0 (0.0 to 0.0) |  | 10.8 (6.4 to 16.6) | 0.0 (0.0 to 0.0) |  | 9297.9 (6323.1 to 12974.4) | NA |
| El Salvador | 4.7 (1.7 to 10.7) | 248.8 (91.5 to 554.1) |  | 2.0 (0.6 to 5.0) | 101.2 (32.2 to 247.7) |  | 22.5 (8.8 to 50.1) | 264.9 (103.5 to 571.8) |  | 15.4 (9.3 to 23.4) | 20.7 (9.3 to 38.1) |  | 52.1 (31.7 to 77.8) | 0.0 (0.0 to 0.0) |  | 18.0 (10.8 to 27.7) | 0.0 (0.0 to 0.0) |  | 15618.7 (10621.8 to 21795.6) | NA |
| Guatemala | 27.9 (18.3 to 40.5) | 1667.2 (1100.6 to 2419.5) |  | 11.5 (6.6 to 17.3) | 559.2 (322.3 to 844.6) |  | 81.7 (53.1 to 120.2) | 1178.9 (786.8 to 1712.9) |  | 64.1 (40.7 to 93.4) | 245.3 (124.4 to 392.2) |  | 182.5 (111.3 to 273.0) | 0.0 (0.0 to 0.0) |  | 63.3 (37.7 to 97.0) | 0.0 (0.0 to 0.0) |  | 54617.8 (37123.3 to 76270.3) | NA |
| Honduras | 30.8 (11.9 to 61.8) | 1867.0 (718.5 to 3684.2) |  | 15.3 (5.8 to 33.6) | 744.6 (288.0 to 1629.7) |  | 59.9 (21.2 to 141.1) | 856.4 (309.5 to 1952.5) |  | 34.9 (21.9 to 52.2) | 114.4 (67.4 to 192.4) |  | 103.1 (62.9 to 154.2) | 0.0 (0.0 to 0.0) |  | 35.6 (21.2 to 54.5) | 0.0 (0.0 to 0.0) |  | 30909.4 (21008.4 to 43166.3) | NA |
| Mexico | 379.1 (264.4 to 526.5) | 16294.2 (11887.2 to 20972.8) |  | 121.3 (84.4 to 172.2) | 5183.5 (3837.0 to 6922.6) |  | 256.5 (180.9 to 388.0) | 2652.6 (1956.9 to 3784.0) |  | 471.7 (326.2 to 654.5) | 1911.6 (1158.6 to 2677.5) |  | 1004.0 (629.1 to 1474.6) | 0.0 (0.0 to 0.0) |  | 522.8 (316.4 to 773.6) | 0.0 (0.0 to 0.0) |  | 316782.5 (229039.9 to 425724.0) | NA |
| Nicaragua | 12.9 (6.9 to 22.9) | 141.5 (78.4 to 237.2) |  | 5.3 (2.8 to 9.1) | 256.5 (140.5 to 445.9) |  | 12.9 (6.9 to 22.9) | 141.5 (78.4 to 237.2) |  | 19.9 (12.7 to 28.6) | 88.8 (49.2 to 135.4) |  | 60.3 (36.7 to 90.1) | 0.0 (0.0 to 0.0) |  | 20.9 (12.5 to 32.1) | 0.0 (0.0 to 0.0) |  | 18051.4 (12274.6 to 25193.8) | NA |
| Panama | 8.7 (5.5 to 13.0) | 379.0 (261.9 to 531.0) |  | 4.7 (3.1 to 7.0) | 232.4 (154.5 to 336.8) |  | 4.5 (2.8 to 7.7) | 47.3 (31.0 to 76.3) |  | 11.7 (7.7 to 17.2) | 27.7 (15.6 to 59.1) |  | 35.2 (21.5 to 52.7) | 0.0 (0.0 to 0.0) |  | 12.2 (7.3 to 18.7) | 0.0 (0.0 to 0.0) |  | 10570.4 (7188.5 to 14751.0) | NA |
| Venezuela (Bolivarian Republic of) | 52.8 (33.7 to 77.4) | 2641.9 (1724.2 to 3859.2) |  | 21.7 (13.0 to 33.0) | 1116.3 (686.2 to 1679.6) |  | 30.4 (18.8 to 46.0) | 352.3 (225.6 to 545.1) |  | 92.3 (62.3 to 132.4) | 374.2 (217.1 to 572.5) |  | 209.4 (127.7 to 313.1) | 0.0 (0.0 to 0.0) |  | 72.1 (43.0 to 110.5) | 0.0 (0.0 to 0.0) |  | 62779.4 (42667.3 to 87682.1) | NA |
| **Tropical Latin America** | 349.6 (268.2 to 451.2) | 15255.1 (11637.1 to 19376.8) |  | 221.8 (161.2 to 284.2) | 8934.3 (6503.3 to 11344.8) |  | 457.9 (340.4 to 593.6) | 4861.2 (3643.4 to 6307.1) |  | 131.5 (73.0 to 216.4) | 1500.2 (1032.5 to 1992.9) |  | 472.8 (251.6 to 765.2) | 0.0 (0.0 to 0.0) |  | 247.7 (120.7 to 402.6) | 0.0 (0.0 to 0.0) |  | 75760.7 (52145.8 to 104311.2) | NA |
| Brazil | 345.2 (263.1 to 442.4) | 15003.5 (11493.1 to 19160.6) |  | 218.9 (160.1 to 279.4) | 8785.1 (6404.0 to 11129.1) |  | 446.6 (335.0 to 572.4) | 4720.2 (3569.2 to 6086.9) |  | 126.9 (70.5 to 208.6) | 1485.1 (1017.0 to 1975.3) |  | 452.1 (241.6 to 732.7) | 0.0 (0.0 to 0.0) |  | 240.2 (117.2 to 390.3) | 0.0 (0.0 to 0.0) |  | 72148.7 (49671.8 to 99246.7) | NA |
| Paraguay | 4.4 (1.9 to 10.9) | 251.6 (108.8 to 610.4) |  | 2.9 (1.3 to 7.6) | 149.2 (65.9 to 396.7) |  | 11.3 (5.3 to 23.3) | 141.0 (67.1 to 291.8) |  | 4.6 (2.3 to 7.8) | 15.1 (7.7 to 28.2) |  | 20.6 (10.7 to 33.5) | 0.0 (0.0 to 0.0) |  | 7.5 (3.4 to 12.6) | 0.0 (0.0 to 0.0) |  | 3612 (2416.8 to 5070.2) | NA |
| **North Africa and Middle East** | 579.2 (316.2 to 829.4) | 27609.2 (14630.6 to 40140.5) |  | 524.5 (309.5 to 767.2) | 25443.6 (15012.2 to 37622.6) |  | 3024.8 (2097.4 to 4197.6) | 41899.3 (27429.9 to 61533.9) |  | 3945.2 (2614.1 to 5472.5) | 1156.2 (846.0 to 1517.1) |  | 1141.3 (529.0 to 1957.3) | 0.0 (0.0 to 0.0) |  | 293.3 (105.9 to 555.2) | 0.0 (0.0 to 0.0) |  | 1036284.3 (725628.1to 1404771.2) | NA |
| **North Africa and Middle East** | 579.2 (316.2 to 829.4) | 27609.2 (14630.6 to 40140.5) |  | 524.5 (309.5 to 767.2) | 25443.6 (15012.2 to 37622.6) |  | 3024.8 (2097.4 to 4197.6) | 41899.3 (27429.9 to 61533.9) |  | 3945.2 (2614.1 to 5472.5) | 1156.2(846to1517.1) |  | 1141.3 (529.0 to 1957.3) | 0.0 (0.0 to 0.0) |  | 293.3 (105.9 to 555.2) | 0.0 (0.0 to 0.0) |  | 1036284.3 (725628.1to 1404771.2) | NA |
| Afghanistan | 98.7 (34.1 to 208.0) | 6108.7 (2113.7 to 12701.1) |  | 88.1 (27.8 to 187.8) | 4288.9 (1378.5 to 8934.0) |  | 636.7 (239.9 to 1259.0) | 12766.5 (4903.3 to 24673.2) |  | 352.0 (218.1 to 523.8) | 92.0 (54.6 to 150.2) |  | 118.9 (52.5 to 208.4) | 0.0 (0.0 to 0.0) |  | 30.2 (9.8 to 58.7) | 0.0 (0.0 to 0.0) |  | 114094.8 (78806.8 to 155107.2) | NA |
| Algeria | 14.2 (6.4 to 27.9) | 653.2 (295.8 to 1242.3) |  | 34.4 (21.0 to 59.0) | 1619.5 (992.8 to 2779.7) |  | 62.3 (31.8 to 112.4) | 649.9 (344.6 to 1194.9) |  | 320.6 (215.2 to 461.0) | 61.9 (44.9 to 88.3) |  | 76.9 (33.8 to 134.7) | 0.0 (0.0 to 0.0) |  | 19.6 (6.3 to 38.0) | 0.0 (0.0 to 0.0) |  | 73742.1 (50872.3 to 100420.2) | NA |
| Bahrain | 0.5 (0.3 to 1.0) | 15.7 (9.2 to 24.7) |  | 0.5 (0.3 to 0.8) | 23.5 (13.9 to 40.0) |  | 0.7 (0.3 to 1.1) | 5.9 (3.2 to 9.9) |  | 3.2 (2.0 to 4.6) | 1.1 (0.7 to 1.8) |  | 1.3 (0.6 to 2.2) | 0.0 (0.0 to 0.0) |  | 0.3 (0.1 to 0.6) | 0.0 (0.0 to 0.0) |  | 1223.6 (845.1 to 1664.9) | NA |
| Egypt | 52.2 (22.7 to 112.6) | 2986.0 (1310.4 to 6387.3) |  | 61.4 (24.6 to 138.0) | 3040.4 (1249.7 to 6889.4) |  | 259.0 (93.5 to 552.2) | 3300.9 (1245.8 to 6956.5) |  | 622.8 (397.8 to 897.5) | 115.2 (77.6 to 162.6) |  | 194.7 (85.5 to 341.3) | 0.0 (0.0 to 0.0) |  | 49.6 (15.9 to 96.0) | 0.0 (0.0 to 0.0) |  | 186724.9 (128858.1to 254066.6) | NA |
| Iran (Islamic Republic of) | 73.9 (34.4 to 135.5) | 2496.1 (1245.6 to 4081.5) |  | 66.3 (27.1 to 99.9) | 3166.1 (1300.6 to 4957.6) |  | 217.2 (73.8 to 403.7) | 2056.8 (674.5 to 3799.3) |  | 504.7 (332.7 to 703.0) | 88.3 (64.3 to 116.8) |  | 140.5 (65.8 to 237.8) | 0.0 (0.0 to 0.0) |  | 41.5 (14.4 to 75.1) | 0.0 (0.0 to 0.0) |  | 119437.0 (82400.8 to 161214.6) | NA |
| Iraq | 21.1 (10.3 to 37.9) | 1110.0 (555.8 to 2003.4) |  | 25.0 (12.6 to 45.6) | 1210.2 (625.9 to 2133.9) |  | 340.2 (186.3 to 541.9) | 3928.7 (2205.0 to 6258.5) |  | 274.2 (179.4 to 402.7) | 53.1 (37.2 to 74.2) |  | 83.7 (36.8 to 146.6) | 0.0 (0.0 to 0.0) |  | 21.3 (6.9 to 41.4) | 0.0 (0.0 to 0.0) |  | 80277.2 (55380.7 to 109313.1) | NA |
| Jordan | 2.1 (1.1 to 3.8) | 80.9 (44.2 to 134.0) |  | 2.6 (1.4 to 4.4) | 125.7 (68.8 to 212.2) |  | 103.0 (64.6 to 164.7) | 996.3 (639.8 to 1577.7) |  | 67.5 (45.2 to 93.9) | 15.6 (9.5 to 23.0) |  | 23.9 (10.8 to 41.4) | 0.0 (0.0 to 0.0) |  | 4.4 (1.1 to 9.1) | 0.0 (0.0 to 0.0) |  | 17142.1 (12298.0 to 23020.1) | NA |
| Kuwait | 5.7 (2.9 to 11.0) | 85.9 (58.7 to 124.9) |  | 1.4 (1.0 to 2.0) | 69.1 (48.2 to 96.8) |  | 13.6 (9.3 to 19.1) | 107.4 (75.9 to 146.7) |  | 20.1 (13.5 to 27.9) | 3.8 (3.0 to 4.9) |  | 5.4 (2.4 to 9.4) | 0.0 (0.0 to 0.0) |  | 1.4 (0.4 to 2.6) | 0.0 (0.0 to 0.0) |  | 5143.2 (3548.9 to 7002.6) | NA |
| Lebanon | 10.7 (4.1 to 23.1) | 179.5 (78.4 to 317.5) |  | 4.0 (1.7 to 7.4) | 195.5 (87.1 to 357.2) |  | 16.8 (7.5 to 32.3) | 136.5 (60.1 to 263.1) |  | 33.0 (22.2 to 46.0) | 6.7 (4.1 to 10.1) |  | 9.1 (4.0 to 15.9) | 0.0 (0.0 to 0.0) |  | 2.3 (0.7 to 4.5) | 0.0 (0.0 to 0.0) |  | 8719.2 (6015.9 to 11874.9) | NA |
| Libya | 3.5 (1.7 to 6.7) | 177.3 (87.5 to 335.8) |  | 4.1 (1.9 to 7.6) | 193.2 (93.1 to 351.2) |  | 17.5 (7.6 to 33.7) | 181.4 (84.2 to 333.5) |  | 29.1 (19.5 to 40.3) | 5.9 (4.0 to 8.5) |  | 7.6 (3.4 to 13.4) | 0.0 (0.0 to 0.0) |  | 1.9 (0.6 to 3.8) | 0.0 (0.0 to 0.0) |  | 7316.2 (5049.6 to 9952.7) | NA |
| Morocco | 7.6 (3.5 to 14.8) | 441.8 (207.1 to 846.9) |  | 9.5 (4.4 to 18.8) | 467.6 (214.9 to 897.9) |  | 29.7 (11.4 to 61.8) | 374.4 (155.3 to 754.0) |  | 209.0 (128.9 to 298.5) | 40.1 (23.8 to 63.7) |  | 54.8 (24.1 to 96.1) | 0.0 (0.0 to 0.0) |  | 14.0 (4.5 to 27.1) | 0.0 (0.0 to 0.0) |  | 52584.8 (36279.1 to 71568.5) | NA |
| Oman | 3.7 (1.5 to 7.6) | 99.4 (42.1 to 167.1) |  | 2.6 (1.3 to 4.2) | 129.7 (63.7 to 203.7) |  | 9.4 (4.9 to 18.2) | 83.2 (43.9 to 162.4) |  | 25.5 (16.9 to 36.6) | 6.5 (4.5 to 9.4) |  | 7.0 (3.1 to 12.3) | 0.0 (0.0 to 0.0) |  | 1.8 (0.6 to 3.5) | 0.0 (0.0 to 0.0) |  | 6755.8 (4659.2 to 9198.1) | NA |
| Palestine | 2.1 (1.2 to 3.5) | 105.0 (60.0 to 175.8) |  | 2.5 (1.3 to 4.1) | 120.2 (66.5 to 198.4) |  | 37.5 (21.0 to 64.1) | 424.2 (243.7 to 719.2) |  | 28.9 (18.3 to 43.5) | 6.9 (5.2 to 9.1) |  | 11.2 (4.9 to 19.6) | 0.0 (0.0 to 0.0) |  | 2.9 (0.9 to 5.5) | 0.0 (0.0 to 0.0) |  | 10745.9 (7414.9 to 14626.5) | NA |
| Qatar | 1.3 (0.5 to 2.7) | 21.8 (10.2 to 41.4) |  | 1.0 (0.5 to 1.9) | 48.4 (25.4 to 93.0) |  | 2.3 (0.7 to 4.8) | 17.6 (6.0 to 35.5) |  | 6.5 (4.0 to 9.6) | 1.6 (1.2 to 2.2) |  | 3.3 (1.7 to 5.4) | 0.0 (0.0 to 0.0) |  | 0.7 (0.2 to 1.3) | 0.0 (0.0 to 0.0) |  | 3283.7 (2296.8 to 4440.3) | NA |
| Saudi Arabia | 16.3 (4.7 to 38.2) | 559.1 (184.8 to 1175.4) |  | 5.3 (1.9 to 10.7) | 297.2 (113.6 to 616.5) |  | 12.4 (3.4 to 38.5) | 127.8 (35.8 to 357.9) |  | 108.1 (68.5 to 160.2) | 23.3 (15.3 to 32.4) |  | 41.0 (18.0 to 71.8) | 0.0 (0.0 to 0.0) |  | 10.4 (3.3 to 20.2) | 0.0 (0.0 to 0.0) |  | 39291.7 (27116.5 to 53476.4) | NA |
| Sudan | 88.0 (28.4 to 185.5) | 5294.6 (1742.4 to 11094.6) |  | 88.1 (22.3 to 182.4) | 4291.6 (1107.4 to 8886.9) |  | 563.6 (218.7 to 1149.6) | 8032.5 (3292.9 to 15459.1) |  | 348.7 (213.6 to 502.5) | 82.9 (48.5 to 137.4) |  | 98.8 (43.5 to 173.2) | 0.0 (0.0 to 0.0) |  | 25.1 (8.1 to 48.8) | 0.0 (0.0 to 0.0) |  | 94803.2 (65421.1 to 129081.9) | NA |
| Syrian Arab Republic | 13.3 (6.5 to 31.2) | 657.7 (337.4 to 1523.1) |  | 12.1 (5.8 to 27.2) | 603.5 (298.1 to 1330.2) |  | 113.7 (58.7 to 234.3) | 1278.0 (674.4 to 2576.4) |  | 54.7 (34.1 to 80.5) | 20.7 (13.7 to 32.3) |  | 20.4 (9.0 to 35.8) | 0.0 (0.0 to 0.0) |  | 5.2 (1.7 to 10.1) | 0.0 (0.0 to 0.0) |  | 19574.5 (13506.1 to 26650.8) | NA |
| Tunisia | 6.5 (3.0 to 12.5) | 213.8 (104.5 to 388.4) |  | 5.1 (2.5 to 8.6) | 248.0 (123.1 to 418.3) |  | 11.6 (5.4 to 22.9) | 109.1 (53.3 to 212.4) |  | 59.3 (38.3 to 83.4) | 11.9 (8.6 to 16.7) |  | 15.6 (6.9 to 27.4) | 0.0 (0.0 to 0.0) |  | 3.9 (1.3 to 7.6) | 0.0 (0.0 to 0.0) |  | 14957.6 (10327.7 to 20344.0) | NA |
| Turkey | 114.5 (53.2 to 203.4) | 3669.8 (1866.5 to 5846.9) |  | 67.2 (39.8 to 103.8) | 3199.3 (1967.0 to 4878.9) |  | 247.6 (132.1 to 395.4) | 2260.8 (1229.1 to 3502.3) |  | 595.7 (416.6 to 794.4) | 463.7 (265.5 to 703.8) |  | 140.3 (71.8 to 231.1) | 0.0 (0.0 to 0.0) |  | 34.4 (14.1 to 62.2) | 0.0 (0.0 to 0.0) |  | 96994 (66275.2 to 131318.8) | NA |
| United Arab Emirates | 0.6 (0.3 to 1.4) | 34.5 (14.6 to 73.6) |  | 1.1 (0.5 to 2.5) | 56.3 (24.2 to 126.9) |  | 1.7 (0.6 to 4.1) | 20.0 (7.2 to 48.4) |  | 20.2 (13.0 to 28.4) | 5.1 (3.3 to 7.8) |  | 6.0 (2.7 to 10.6) | 0.0 (0.0 to 0.0) |  | 1.5 (0.5 to 3.0) | 0.0 (0.0 to 0.0) |  | 5776.6 (3996.9 to 7885.6) | NA |
| Yemen | 42.2 (15.3 to 76.7) | 2590.2 (937.3 to 4680.3) |  | 41.7 (12.7 to 90.6) | 2024.0 (617.1 to 4213) |  | 325.4 (133.8 to 601.2) | 4998.9 (1967.1 to 9049.9) |  | 257.4 (161.9 to 370.5) | 48.7 (30.4 to 77.4) |  | 79.9 (35.1 to 139.9) | 0.0 (0.0 to 0.0) |  | 20.4 (6.6 to 39.6) | 0.0 (0.0 to 0.0) |  | 76643.4 (52866.4 to 104383.0) | NA |
| **South Asia** | 1632.8 (1027.4 to 2714.2) | 80150.8 (52181.6 to 128619.3) |  | 1676.3 (1133.8 to 2458.3) | 66054.5 (46376.8 to 93639.5) |  | 4115.8 (2026.9 to 6661.9) | 55367.5 (28267.6 to 87804.3) |  | 4421.6 (2570.5 to 6706.4) | 3354.4 (2171.4 to 5388.5) |  | 3126.9 (1476.3 to 5279.3) | 0.0 (0.0 to 0.0) |  | 1087.4 (423.0 to 1945.3) | 0.0 (0.0 to 0.0) |  | 773984.8 (520591.9 to 1081220.4) | NA |
| **South Asia** | 1632.8 (1027.4 to 2714.2) | 80150.8 (52181.6 to 128619.3) |  | 1676.3 (1133.8 to 2458.3) | 66054.5 (46376.8 to 93639.5) |  | 4115.8 (2026.9 to 6661.9) | 55367.5 (28267.6 to 87804.3) |  | 4421.6 (2570.5 to 6706.4) | 3354.4 (2171.4 to 5388.5) |  | 3126.9 (1476.3 to 5279.3) | 0.0 (0.0 to 0.0) |  | 1087.4 (423.0 to 1945.3) | 0.0 (0.0 to 0.0) |  | 773984.8 (520591.9 to 1081220.4) | NA |
| Bangladesh | 95.0 (51.7 to 153.6) | 5772.0 (3141.4 to 9348.2) |  | 109.5 (63.0 to 179.8) | 5332.9 (3092.1 to 8618.0) |  | 199.3 (109.1 to 340.2) | 2954.5 (1680.6 to 4908.9) |  | 170.1 (81.5 to 288.3) | 242.1 (157.0 to 370.9) |  | 187.1 (67.9 to 342.8) | 0.0 (0.0 to 0.0) |  | 75.0 (26.2 to 137.6) | 0.0 (0.0 to 0.0) |  | 49613.8 (32988.8 to 71032.6) | NA |
| Bhutan | 0.8 (0.4 to 1.6) | 48.5 (21.8 to 93.9) |  | 1.0 (0.4 to 2.0) | 47.6 (21.6 to 90.1) |  | 2.0 (0.8 to 4.2) | 27.1 (11.7 to 54.4) |  | 1.0 (0.5 to 1.6) | 2.1 (1.1 to 3.8) |  | 0.9 (0.3 to 1.6) | 0.0 (0.0 to 0.0) |  | 0.4 (0.1 to 0.7) | 0.0 (0.0 to 0.0) |  | 235.8 (156.9 to 337.7) | NA |
| India | 910.0 (587.7 to 1454.1) | 45976.9 (31403.4 to 69497.8) |  | 963.2 (636.9 to 1464.7) | 39099.8 (27573.5 to 55845.0) |  | 1964.2 (994.6 to 3249.8) | 27849.4 (14219.0 to 44768.6) |  | 3482.8 (2050.6 to 5244.4) | 2179.6 (1489.4 to 3236.7) |  | 2398.5 (1120.7 to 4038.2) | 0.0 (0.0 to 0.0) |  | 776.8 (302.9 to 1397.7) | 0.0 (0.0 to 0.0) |  | 593358.5 (398822.2 to 824155.1) | NA |
| Nepal | 15.5 (8.4 to 26.0) | 951.9 (515.2 to 1603.2) |  | 16.6 (8.7 to 27.4) | 808.8 (427.0 to 1278.0) |  | 31.8 (15.8 to 54.2) | 516.0 (260.6 to 861.5) |  | 24.9 (9.9 to 46.0) | 36.9 (23.4 to 59.5) |  | 35.6 (14.4 to 63.9) | 0.0 (0.0 to 0.0) |  | 18.8 (7.5 to 34.0) | 0.0 (0.0 to 0.0) |  | 11455.2 (7819.2 to 15994.0) | NA |
| Pakistan | 611.5 (294.3 to 1311.6) | 27401.5 (12875.2 to 57493.1) |  | 585.9 (287.8 to 1070.9) | 20765.4 (10074.2 to 38442.1) |  | 1918.6 (729.8 to 3592.6) | 24020.5 (9342.1 to 44469.7) |  | 742.8 (382.1 to 1135.9) | 893.7 (393.0 to 1883.2) |  | 504.9 (215.4 to 880.6) | 0.0 (0.0 to 0.0) |  | 216.5 (88.4 to 385.4) | 0.0 (0.0 to 0.0) |  | 119321.3 (80676.4 to 168058.4) | NA |
| **Southeast Asia, East Asia, and Oceania** | 5331.0 (3815.8 to 7388.5) | 125550.3 (96497.6 to 158257.9) |  | 902.8 (684.0 to 1384.0) | 49164.8 (37258.7 to 77211.6) |  | 14402.2 (10934.1 to 19885.4) | 162034.8 (127747.3 to 218314.1) |  | 2441.9 (1528.2 to 3665.0) | 5964.1 (4736.0 to 8371.9) |  | 1353.6 (481.8 to 2564.9) | 0.0 (0.0 to 0.0) |  | 710.0 (274.0 to 1311.5) | 0.0 (0.0 to 0.0) |  | 1762529.2 (1251944.6 to 2410470.9) | NA |
| **East Asia** | 4602.5 (3008.1 to 6622.2) | 80020.6 (54246.0 to 105162.2) |  | 466.6 (333.7 to 763.0) | 25505.4 (18308.2 to 41941.4) |  | 10638.4 (7368.5 to 15637.6) | 100544.5 (70523.2 to 148749.1) |  | 1229.9 (796.4 to 1859.7) | 4561.2 (3484.4 to 6445.1) |  | 685.0 (237.1 to 1336.6) | 0.0 (0.0 to 0.0) |  | 416.7 (152.1 to 781.8) | 0.0 (0.0 to 0.0) |  | 1409831.0 (993730.5 to 1921952.7) | NA |
| China | 4543.9 (2966.6 to 6553.2) | 78599.2 (53278.6 to 103893.5) |  | 450.4 (320.3 to 742.9) | 24712.7 (17624.1 to 41099.2) |  | 10395.0 (7195.3 to 15389.5) | 97611.2 (68213.8 to 144987.1) |  | 1192.9 (776.4 to 1804.4) | 4489.6 (3438.3 to 6369.2) |  | 633.3 (214.2 to 1251.1) | 0.0 (0.0 to 0.0) |  | 402.0 (145.7 to 754.7) | 0.0 (0.0 to 0.0) |  | 1381518.8 (972056.5to 1885355.6) | NA |
| Democratic People's Republic of Korea | 17.8 (9.0 to 32.0) | 1039.9 (526.2 to 1837.9) |  | 9.1 (4.1 to 18.0) | 447.2 (202.5 to 882.1) |  | 191.5 (104.7 to 335.1) | 2546.5 (1432.2 to 4312.9) |  | 22.2 (11.9 to 35.7) | 54.0 (29.5 to 93.5) |  | 21.7 (7.3 to 41.0) | 0.0 (0.0 to 0.0) |  | 8.8 (3.0 to 16.5) | 0.0 (0.0 to 0.0) |  | 20260.2 (13315.1 to 28417.8) | NA |
| Taiwan (Province of China) | 40.8 (17.8 to 79.0) | 381.5 (219.7 to 565.0) |  | 7.0 (4.3 to 10.1) | 345.6 (215.0 to 491.4) |  | 51.9 (35.9 to 79.3) | 386.8 (268.2 to 580.7) |  | 14.7 (9.4 to 21.9) | 17.5 (12.2 to 25.9) |  | 30.0 (15.9 to 47.8) | 0.0 (0.0 to 0.0) |  | 5.9 (2.2 to 11.0) | 0.0 (0.0 to 0.0) |  | 8052 (6572.3 to 9947.2) | NA |
| **Oceania** | 19.6 (7.6 to 44.3) | 1208.2 (468.5 to 2740.0) |  | 25.2 (12.0 to 50.0) | 1235.5 (591.4 to 2410.5) |  | 169.4 (51.5 to 355.1) | 3054.0 (940.3 to 6250.6) |  | 53.2 (33.8 to 79.3) | 179.9 (81.0 to 365.2) |  | 18.1 (6.0 to 35.6) | 0.0 (0.0 to 0.0) |  | 7.5 (2.2 to 15.3) | 0.0 (0.0 to 0.0) |  | 17198.6 (11593.1 to 24181.2) | NA |
| American Samoa | 0.0 (0.0 to 0.0) | 1.1 (0.5 to 2.2) |  | 0.0 (0.0 to 0.1) | 1.2 (0.5 to 2.4) |  | 0.1 (0.0 to 0.2) | 1.2 (0.6 to 2.0) |  | 0.2 (0.1 to 0.3) | 0.3 (0.1 to 0.6) |  | 0.1 (0.0 to 0.1) | 0.0 (0.0 to 0.0) |  | 0.0 (0.0 to 0.0) | 0.0 (0.0 to 0.0) |  | 48.1 (32.4 to 67.5) | NA |
| Cook Islands | 0.0 (0.0 to 0.0) | 0.1 (0.0 to 0.2) |  | 0.0 (0.0 to0.0) | 0.2 (0.0 to 0.4) |  | 0.0 (0.0 to0.0) | 0.1 (0.0 to 0.2) |  | 0.0 (0.0 to 0.1) | 0.1 (0.0 to 0.2) |  | 0.0 (0.0 to 0.0) | 0.0 (0.0 to 0.0) |  | 0.0 (0.0 to 0.0) | 0.0 (0.0 to 0.0) |  | 12.5 (8.4 to 17.6) | NA |
| Fiji | 1.1 (0.6 to 1.8) | 65.2 (35.1 to 110.3) |  | 3.1 (1.7 to 5.2) | 150.7 (84.2 to 252.4) |  | 3.1 (1.7 to 5.2) | 45.0 (25.0 to 74.2) |  | 3.8 (2.7 to 5.1) | 8.5 (4.6 to 14.8) |  | 0.9 (0.3 to 1.7) | 0.0 (0.0 to 0.0) |  | 0.4 (0.1 to 0.7) | 0.0 (0.0 to 0.0) |  | 824.8 (556.5 to 1159.7) | NA |
| Guam | 0.7 (0.4 to 1.1) | 7.2 (4.4 to 11.4) |  | 0. 2 (0.1 to 0.2) | 7.6 (4.3 to 12.3) |  | 0.7 (0.4 to 1.1) | 7.2 (4.4 to 11.4) |  | 0.5 (0.3 to 0.7) | 1.2 (0.7 to 2.3) |  | 0.2 (0.1 to 0.3) | 0.0 (0.0 to 0.0) |  | 0.1 (0.0 to 0.1) | 0.0 (0.0 to 0.0) |  | 147.6 (99.5 to 207.4) | NA |
| Kiribati | 0. 1 (0.0 to 0.1) | 3.2 (1.3 to 6.7) |  | 0.1 (0.0 to 0.1) | 2.9 (1.3 to 5.7) |  | 0.7 (0.3 to 1.3) | 13.4 (6.7 to 26.2) |  | 0.4 (0.3 to 0.6) | 0.6 (0.3 to 1.3) |  | 0.1 (0.0 to 0.3) | 0.0 (0.0 to 0.0) |  | 0.1 (0.0 to 0.1) | 0.0 (0.0 to 0.0) |  | 135.7 (91.5 to 190.7) | NA |
| Marshall Islands | 0.0 (0.0 to 0.1) | 1.9 (0.9 to 3.4) |  | 0.0 (0.0 to 0.1) | 2.4 (1.1 to 4.3) |  | 0.2 (0.1 to 0.3) | 2.9 (1.4 to 5.1) |  | 0.2 (0.1 to 0.3) | 0.4 (0.2 to 0.7) |  | 0.1 (0.0 to 0.1) | 0.0 (0.0 to 0.0) |  | 0.0 (0.0 to 0.1) | 0.0 (0.0 to 0.0) |  | 56.7 (38.3 to 79.7) | NA |
| Micronesia (Federated States of) | 0.0 (0.0 to 0.1) | 2.6 (0.6 to 4.9) |  | 0.1 (0.0 to 0.1) | 3.3 (0.7 to 6.8) |  | 0.2 (0.1 to 0.4) | 3.2 (1.1 to 6.3) |  | 0.3 (0.2 to 0.4) | 0.5 (0.2 to 1.0) |  | 0.1 (0.0 to 0.2) | 0.0 (0.0 to 0.0) |  | 0.0 (0.0 to 0.1) | 0.0 (0.0 to 0.0) |  | 90.7 (61.2 to 127.5) | NA |
| Nauru | 0.0 (0.0 to 0.0) | 0.9 (0.4 to 1.7) |  | 0.0 (0.0 to0.0) | 1.2 (0.6 to 2.2) |  | 0.1 (0.1 to 0.2) | 1.5 (0.7 to 2.6) |  | 0.0 (0.0 to 0.1) | 0.2 (0.1 to 0.3) |  | 0.0 (0.0 to 0.0) | 0.0 (0.0 to 0.0) |  | 0.0 (0.0 to 0.0) | 0.0 (0.0 to 0.0) |  | 13.0 (8.8 to 18.3) | NA |
| Niue | 0.0 (0.0 to0.0) | 0.1 (0.0 to 0.2) |  | 0.0 (0.0 to 0.0) | 0.1 (0.0 to 0.2) |  | 0.0 (0.0 to0.0) | 0.1 (0.0 to 0.2) |  | 0.0 (0.0 to 0.0) | 0.0 (0.0 to 0.0) |  | 0.0 (0.0 to 0.0) | 0.0 (0.0 to 0.0) |  | 0.0 (0.0 to 0.0) | 0.0 (0.0 to 0.0) |  | 1.2 (0.8 to 1.7) | NA |
| Northern Mariana Islands | 0.0 (0.0 to 0.0) | 0.6 (0.3 to 1.2) |  | 0.0 (0.0 to 0.0) | 1.3 (0.7 to 2.3) |  | 0.1 (0.0 to 0.2) | 1.0 (0.5 to 1.6) |  | 0.1 (0.0 to 0.1) | 0.4 (0.2 to 0.7) |  | 0.0 (0.0 to 0.0) | 0.0 (0.0 to 0.0) |  | 0.0 (0.0 to 0.0) | 0.0 (0.0 to 0.0) |  | 21.3 (14.4 to 30.0) | NA |
| Palau | 0.0 (0.0 to 0.0) | 0.2 (0.1 to 0.3) |  | 0.0 (0.0 to 0.0) | 0.1 (0.1 to 0.3) |  | 0.0 (0.0 to 0.0) | 0.1 (0.1 to 0.2) |  | 0.0 (0.0 to 0.0) | 0.1 (0.0 to 0.1) |  | 0.0 (0.0 to 0.0) | 0.0 (0.0 to 0.0) |  | 0.0 (0.0 to 0.0) | 0.0 (0.0 to 0.0) |  | 9.2 (6.2 to 12.9) | NA |
| Papua New Guinea | 16.5 (5.3 to 39.9) | 1019.0 (327.7 to 2457.2) |  | 19.4 (7.5 to 42.2) | 949.5 (378.3 to 2033.6) |  | 150.7 (40.0 to 326.6) | 2743.7 (762.1 to 5755.8) |  | 40.4 (24.9 to 61.2) | 150.6 (61.2 to 322.7) |  | 14.2 (4.7 to 27.8) | 0.0 (0.0 to 0.0) |  | 5.9 (1.7 to 11.9) | 0.0 (0.0 to 0.0) |  | 13464.8 (9074.9 to 18934.6) | NA |
| Samoa | 0.1 (0.0 to 0.2) | 3.9 (1.5 to 8.8) |  | 0.1 (0.0 to 0.3) | 4.4 (1.6 to 10.4) |  | 0.4 (0.1 to 0.9) | 4.3 (1.7 to 10.1) |  | 0.7 (0.4 to 0.9) | 1.0 (0.4 to 2.0) |  | 0.2 (0.1 to 0.4) | 0.0 (0.0 to 0.0) |  | 0.1 (0.0 to 0.2) | 0.0 (0.0 to 0.0) |  | 194.8 (131.5 to 273.4) | NA |
| Solomon Islands | 0.5 (0.3 to 1.0) | 33.7 (15.5 to 61.6) |  | 0.7 (0.3 to 1.3) | 34.2 (16.3 to 62.3) |  | 3.8 (1.7 to 6.8) | 62.7 (27.9 to 114.5) |  | 2.6 (1.6 to 3.9) | 5.0 (2.4 to 9.8) |  | 0.9 (0.3 to 1.8) | 0.0 (0.0 to 0.0) |  | 0.4 (0.1 to 0.8) | 0.0 (0.0 to 0.0) |  | 892.1 (601.4 to 1254.1) | NA |
| Tokelau | 0.0 (0.0 to 0.0) | 0.0 (0.0 to 0.1) |  | 0.0 (0.0 to 0.0) | 0.1 (0.0 to 0.1) |  | 0.0 (0.0 to 0.0) | 0.0 (0.0 to 0.1) |  | 0.0 (0.0 to 0.0) | 0.0 (0.0 to 0.0) |  | 0.0 (0.0 to 0.0) | 0.0 (0.0 to 0.0) |  | 0.0 (0.0 to 0.0) | 0.0 (0.0 to 0.0) |  | 1.6 (1.1 to 2.3) | NA |
| Tonga | 0.1 (0.0 to 0.1) | 3.7 (1.7 to 7.1) |  | 0.1 (0.0 to 0.2) | 4.8 (2.2 to 9.6) |  | 0.2 (0.1 to 0.4) | 3.5 (1.7 to 6.7) |  | 0.4 (0.3 to 0.6) | 0.7 (0.4 to 1.3) |  | 0.1 (0.0 to 0.2) | 0.0 (0.0 to 0.0) |  | 0.0 (0.0 to 0.1) | 0.0 (0.0 to 0.0) |  | 112.5 (75.9 to 158.2) | NA |
| Tuvalu | 0.0 (0.0 to 0.0) | 0.2 (0.1 to 0.5) |  | 0.0 (0.0 to 0.0) | 0.3 (0.1 to 0.6) |  | 0.0 (0.0 to 0.0) | 0.3 (0.2 to 0.6) |  | 0.0 (0.0 to 0.1) | 0.1 (0.0 to 0.1) |  | 0.0 (0.0 to 0.0) | 0.0 (0.0 to 0.0) |  | 0.0 (0.0 to 0.0) | 0.0 (0.0 to 0.0) |  | 10 (6.7 to 14.0) | NA |
| Vanuatu | 0.2 (0.1 to 0.3) | 11.5 (5.6 to 20.6) |  | 0.3 (0.1 to 0.5) | 12.9 (5.6 to 25.5) |  | 1.1 (0.5 to 1.8) | 19.5 (9.3 to 32.1) |  | 1.0 (0.6 to 1.5) | 1.9 (1.0 to 3.5) |  | 0.4 (0.1 to 0.7) | 0.0 (0.0 to 0.0) |  | 0.2 (0.0 to 0.3) | 0.0 (0.0 to 0.0) |  | 349.6 (235.9 to 491.3) | NA |
| **Southeast Asia** | 708.9 (522.0 to 974.3) | 44321.5 (32313.6 to 61523.4) |  | 411.0 (278.8 to 737.6) | 22423.8 (14973.9 to 41313.7) |  | 3594.4 (2501.8 to 5370.2) | 58436.4 (40689.8 to 88411.1) |  | 1158.8 (695.0 to 1742.1) | 1223.0 (858.1 to 2020.1) |  | 650.5 (240.3 to 1201.5) | 0.0 (0.0 to 0.0) |  | 285.8 (109.4 to 529.0) | 0.0 (0.0 to 0.0) |  | 335499.6 (232017.7 to 457429.5) | NA |
| Cambodia | 20.9 (9.9 to 37.5) | 1271.6 (608.2 to 2260.2) |  | 11.0 (4.9 to 22.3) | 535.4 (235.6 to 1105.9) |  | 150.9 (76.4 to 248.5) | 2345.2 (1204.5 to 3810.8) |  | 23.9 (12.6 to 40.8) | 30.1 (17.2 to 52.1) |  | 18.2 (6.1 to 35.1) | 0.0 (0.0 to 0.0) |  | 6.9 (2.0 to 14.4) | 0.0 (0.0 to 0.0) |  | 10715.1 (7313.0 to 14806.4) | NA |
| Indonesia | 217.1 (147.3 to 349.9) | 15493.6 (10357.8 to 24400.0) |  | 147.6 (83.6 to 295.2) | 8649.8 (4774.8 to 17692.1) |  | 1294.0 (837.2 to 1999.3) | 23572.6 (15440.2 to 37277.8) |  | 470.1 (282.3 to 701.2) | 439.8 (242.7 to 911.9) |  | 252.4 (94.5 to 458.8) | 0.0 (0.0 to 0.0) |  | 131.1 (51.6 to 231.8) | 0.0 (0.0 to 0.0) |  | 128835.3 (90705.2 to 173703.0) | NA |
| Lao People's Democratic Republic | 9.3 (4.0 to 18.4) | 570.3 (247.4 to 1140.6) |  | 5.2 (2.1 to 10.7) | 252.0 (106.6 to 512.7) |  | 61.9 (27.6 to 113.3) | 1088.8 (509.2 to 1951.4) |  | 14.3 (8.2 to 22.5) | 16.8 (8.0 to 34.2) |  | 8.3 (2.8 to 15.9) | 0.0 (0.0 to 0.0) |  | 3.2 (0.9 to 6.5) | 0.0 (0.0 to 0.0) |  | 4857.6 (3314.8 to 6712.6) | NA |
| Malaysia | 31.1 (12.5 to 66.5) | 1527.2 (635.7 to 3137.1) |  | 11.5 (3.8 to 31.4) | 567.5 (191.5 to 1596.1) |  | 40.4 (11.0 to 86.7) | 448.6 (122.9 to 956.1) |  | 59.1 (37.1 to 88.8) | 90.7 (54.1 to 149.2) |  | 26.4 (8.8 to 50.9) | 0.0 (0.0 to 0.0) |  | 10.0 (2.8 to 20.7) | 0.0 (0.0 to 0.0) |  | 15518.7 (10583.9 to 21431.3) | NA |
| Maldives | 0.4 (0.2 to 0.8) | 16.7 (8.2 to 29.7) |  | 0.8 (0.4 to 1.4) | 41.0 (20.7 to 70.2) |  | 3.1 (1.7 to 5.0) | 27.5 (16.0 to 42.3) |  | 1.0 (0.7 to 1.5) | 4.0 (2.2 to 6.6) |  | 0.4 (0.1 to 0.8) | 0.0 (0.0 to 0.0) |  | 0.2 (0.0 to 0.3) | 0.0 (0.0 to 0.0) |  | 249.3 (170.1 to 344.4) | NA |
| Mauritius | 0.7 (0.5 to 1.0) | 32.8 (22.7 to 45.6) |  | 0.7 (0.4 to 0.9) | 32.3 (21.3 to 46.6) |  | 5.9 (3.9 to 8.4) | 65.9 (45.1 to 89.7) |  | 1.5 (1.0 to 2.2) | 2.0 (1.3 to 2.8) |  | 0.7 (0.2 to 1.3) | 0.0 (0.0 to 0.0) |  | 0.3 (0.1 to 0.5) | 0.0 (0.0 to 0.0) |  | 390.9 (266.9 to 540.4) | NA |
| Myanmar | 94.7 (37.4 to 194.2) | 5779.2 (2303.9 to 11642.6) |  | 54.3 (20.2 to 124.9) | 2646.5 (977.3 to 6451.9) |  | 696.2 (271.9 to 1362.5) | 11110.4 (4299.0 to 22144.2) |  | 90.9 (53.1 to 140.5) | 146.4 (70.8 to 331.1) |  | 51.7 (17.3 to 99.7) | 0.0 (0.0 to 0.0) |  | 19.7 (5.6 to 40.9) | 0.0 (0.0 to 0.0) |  | 30430.1 (20768.7 to 42050.5) | NA |
| Philippines | 157.2 (104.0 to 213.7) | 10925.8 (7136.1 to 15049.0) |  | 117.1 (82.6 to 184.5) | 6599.7 (4698.1 to 10749.5) |  | 875.6 (574.7 to 1389.9) | 14764.1 (10137.5 to 23409.7) |  | 312.3 (189.9 to 458.0) | 309.1 (217.9 to 440.6) |  | 170.0 (62.5 to 312.2) | 0.0 (0.0 to 0.0) |  | 68.2 (25.0 to 125.2) | 0.0 (0.0 to 0.0) |  | 72579.5 (49468.4 to 100900.3) | NA |
| Seychelles | 0.1 (0.1 to 0.2) | 6.1 (3.8 to 9.7) |  | 0.1 (0.0 to 0.1) | 2.7 (1.4 to 4.8) |  | 0.2 (0.1 to 0.4) | 2.4 (1.2 to 4.6) |  | 0.1 (0.1 to 0.2) | 0.1 (0.1 to 0.2) |  | 0.1 (0.0 to 0.1) | 0.0 (0.0 to 0.0) |  | 0.0 (0.0 to 0.1) | 0.0 (0.0 to 0.0) |  | 45.2 (30.9 to 62.5) | NA |
| Sri Lanka | 19.5 (9.9 to 34.6) | 792.1 (419.2 to 1351.3) |  | 10.9 (4.9 to 19.4) | 536.7 (249.0 to 949.6) |  | 51.5 (23.9 to 102.9) | 513.7 (238.5 to 967.0) |  | 18.0 (9.1 to 29.9) | 21.1 (13.2 to 33.9) |  | 16.0 (5.3 to 30.7) | 0.0 (0.0 to 0.0) |  | 6.1 (1.7 to 12.6) | 0.0 (0.0 to 0.0) |  | 9386.6 (6409.8 to 12972.8) | NA |
| Thailand | 19.7 (7.4 to 39.1) | 802.9 (319.3 to 1518.9) |  | 13.6 (5.4 to 24.1) | 659.4 (267.4 to 1197.7) |  | 319.3 (142.8 to 497.3) | 3294.0 (1481.7 to 5057) |  | 55.3 (32.6 to 87.9) | 40.1 (22.9 to 61.1) |  | 32.3 (10.7 to 62.0) | 0.0 (0.0 to 0.0) |  | 12.2 (3.5 to 25.3) | 0.0 (0.0 to 0.0) |  | 18969.7 (12953.9 to 26212.0) | NA |
| Timor-Leste | 2.0 (0.6 to 4.0) | 123.9 (36.0 to 243.0) |  | 1.2 (0.3 to 2.6) | 59.4 (15.0 to 131.9) |  | 15.2 (3.4 to 28.0) | 250.6 (56.4 to 450.5) |  | 2.2 (1.2 to 3.7) | 3.2 (1.3 to 6.4) |  | 1.8 (0.6 to 3.5) | 0.0 (0.0 to 0.0) |  | 0.7 (0.2 to 1.4) | 0.0 (0.0 to 0.0) |  | 1064.7 (726.3 to 1471.1) | NA |
| Viet Nam | 135.1 (79.6 to 219.3) | 6921.2 (4168.6 to 10974.3) |  | 36.6 (14.3 to 81.4) | 1812.0 (709.7 to 4148.2) |  | 75.3 (27.8 to 189.4) | 876.3 (332.1 to 2276.0) |  | 108.5 (57.2 to 170.0) | 117.8 (69.4 to 192.4) |  | 71.5 (23.8 to 137.5) | 0.0 (0.0 to 0.0) |  | 26.9 (7.7 to 55.7) | 0.0 (0.0 to 0.0) |  | 42017.4 (28678.1 to 58034.5) | NA |
| **Sub-Saharan Africa** | 3268.8 (1782.9 to 5041.9) | 195467.1 (104407.2 to 298175.7) |  | 2391.2 (1355.1 to 3508.0) | 121615.4 (68578.8 to 176187.7) |  | 10981.2 (5848.9 to 15896.3) | 207346.6 (114121.5 to 303926.7) |  | 5219.3 (3140.8 to 7788.1) | 3101.2 (1994.5 to 5777.3) |  | 1752.5 (606.0 to 3407.4) | 0.0 (0.0 to 0.0) |  | 838.8 (302.8 to 1578.3) | 0.0 (0.0 to 0.0) |  | 1482312.3 (1013526.0 to 2071111.2) | NA |
| **Central Sub-Saharan Africa** | 128.2 (50.8 to 263.3) | 7895.8 (3142.4 to 16190.7) |  | 87.6 (37.5 to 189.8) | 4328.6 (1825.9 to 9412.3) |  | 968.5 (385.7 to 1838.4) | 19807.8 (7878.8 to 38717.0) |  | 727.4 (441.1 to 1081.0) | 318.7 (177.0 to 746.8) |  | 207.0 (69.4 to 411.3) | 0.0 (0.0 to 0.0) |  | 92.6 (28.4 to 185.4) | 0.0 (0.0 to 0.0) |  | 203891.0 (138892.8 to 285352.2) | NA |
| Angola | 44.2 (16.6 to 97.4) | 2724.9 (1024.3 to 6003.2) |  | 35.3 (13.1 to 95.0) | 1745.3 (661.3 to 4567.2) |  | 320.0 (123.1 to 608.8) | 6202.9 (2384.4 to 11420.4) |  | 222.9 (131.7 to 320.9) | 131.5 (59.4 to 351.6) |  | 51.5 (17.3 to 102.3) | 0.0 (0.0 to 0.0) |  | 23.0 (7.1 to 46.1) | 0.0 (0.0 to 0.0) |  | 50698.4 (34536.3 to 70950.5) | NA |
| Central African Republic | 8.2 (1.8 to 24.4) | 507.9 (108.3 to 1501.0) |  | 4.4 (1.0 to 12.6) | 216.4 (48.0 to 612.9) |  | 76.6 (15.2 to 191.8) | 1905.4 (390.8 to 4714.2) |  | 26.5 (15.4 to 40.9) | 14.8 (6.5 to 42.5) |  | 8.4 (2.8 to 16.7) | 0.0 (0.0 to 0.0) |  | 3.8 (1.1 to 7.6) | 0.0 (0.0 to 0.0) |  | 8283.6 (5642.1 to 11625.2) | NA |
| Congo | 3.6 (1.4 to 7.6) | 219.8 (86.2 to 470.3) |  | 2.9 (1.2 to 7.1) | 145.4 (58.9 to 349.4) |  | 21.1 (8.9 to 40.0) | 394.3 (171.5 to 765.2) |  | 28.9 (17.6 to 42.9) | 11.2 (5.5 to 29.8) |  | 7.0 (2.3 to 13.8) | 0.0 (0.0 to 0.0) |  | 3.1 (1.0 to 6.3) | 0.0 (0.0 to 0.0) |  | 6854.0 (4669.6 to 9584.2) | NA |
| Democratic Republic of the Congo | 70.4 (24.0 to 169.2) | 4327.6 (1469.2 to 10352.9) |  | 43.0 (13.7 to 107.7) | 2122.9 (677.7 to 5299.6) |  | 540.7 (202.7 to 1197.4) | 11134.7 (4194.7 to 24142.9) |  | 431.8 (253.1 to 679.1) | 152.6 (85.1 to 313.2) |  | 136.3 (45.7 to 270.8) | 0.0 (0.0 to 0.0) |  | 60.9 (18.7 to 122.1) | 0.0 (0.0 to 0.0) |  | 134247.4 (91451.2 to 187874.0) | NA |
| Equatorial Guinea | 0.7 (0.3 to 1.5) | 44.9 (17.3 to 93.2) |  | 0.8 (0.3 to 2.2) | 40.5 (13.2 to 107.3) |  | 4.3 (1.6 to 9.2) | 70.9 (26.5 to 148.7) |  | 8.4 (5.3 to 12.4) | 3.9 (1.4 to 11.7) |  | 1.8 (0.6 to 3.7) | 0.0 (0.0 to 0.0) |  | 0.8 (0.2 to 1.6) | 0.0 (0.0 to 0.0) |  | 1816.7 (1237.3 to 2541.8) | NA |
| Gabon | 1.1 (0.5 to 2.3) | 70.7 (29.2 to 142.5) |  | 1.2 (0.4 to 2.8) | 58.1 (20.3 to 136.9) |  | 5.8 (2.4 to 11.1) | 99.5 (41.5 to 188.8) |  | 8.8 (5.7 to 13.1) | 4.7 (2.0 to 13.1) |  | 2.0 (0.7 to 4.0) | 0.0 (0.0 to 0.0) |  | 0.9 (0.3 to 1.8) | 0.0 (0.0 to 0.0) |  | 1990.9 (1356.3 to 2785.6) | NA |
| **Eastern Sub-Saharan Africa** | 2365.9 (1040.8 to 3953.5) | 142056.9 (61955.7 to 233260.4) |  | 1154.0 (483.8 to 2162.8) | 55623.5 (22924.5 to 103831.6) |  | 7860.3 (3721.9 to 12252.9) | 147207.2 (72808.8 to 226927.2) |  | 1446.2 (800.6 to 2305.3) | 1279.1 (794.0 to 2554.2) |  | 580.6 (186.1 to 1175.1) | 0.0 (0.0 to 0.0) |  | 293.3 (96.4 to 584.8) | 0.0 (0.0 to 0.0) |  | 382046.7 (259008.6 to 538219.9) | NA |
| Burundi | 36.3 (12.0 to 95.1) | 2259.1 (747.4 to 5904.8) |  | 21.2 (5.1 to 61.3) | 1061.3 (249.8 to 3003.7) |  | 222.2 (82.2 to 461.2) | 4605.3 (1777.6 to 9718.5) |  | 38.2 (19.3 to 65.0) | 30.0 (14.8 to 63.2) |  | 17.3 (5.4 to 37.2) | 0.0 (0.0 to 0.0) |  | 8.6 (2.2 to 17.6) | 0.0 (0.0 to 0.0) |  | 12098.6 (8194.0 to 16945.7) | NA |
| Comoros | 1.5 (0.5 to 3.8) | 94.0 (31.6 to 232.6) |  | 1.0 (0.3 to 2.5) | 49.7 (16.5 to 125.5) |  | 7.7 (3.3 to 15.7) | 145.2 (63.4 to 297.5) |  | 2.2 (1.2 to 3.3) | 1.3 (0.8 to 2.3) |  | 0.7 (0.2 to 1.4) | 0.0 (0.0 to 0.0) |  | 0.3 (0.1 to 0.7) | 0.0 (0.0 to 0.0) |  | 453.9 (307.6 to 635.4) | NA |
| Djibouti | 4.4 (1.6 to 9.7) | 273.0 (102.7 to 598.7) |  | 3.0 (1.1 to 6.1) | 148.9 (56.7 to 299.4) |  | 23.7 (10.3 to 43.1) | 420.0 (187.7 to 773.8) |  | 4.3 (2.2 to 6.7) | 4.6 (2.3 to 10.5) |  | 1.3 (0.4 to 2.8) | 0.0 (0.0 to 0.0) |  | 0.6 (0.2 to 1.3) | 0.0 (0.0 to 0.0) |  | 922.7 (624.8 to 1292.5) | NA |
| Eritrea | 20.6 (7.0 to 49.5) | 1279.4 (432.3 to 3054.7) |  | 12.7 (4.2 to 31.3) | 629.2 (207.2 to 1570.3) |  | 97.5 (40.6 to 196.1) | 2030.3 (821.2 to 4057.7) |  | 18.8 (10.0 to 30.7) | 19.4 (10.7 to 41.1) |  | 7.7 (2.4 to 16.7) | 0.0 (0.0 to 0.0) |  | 3.8 (1.0 to 7.8) | 0.0 (0.0 to 0.0) |  | 5404.8 (3661.6 to 7566.4) | NA |
| Ethiopia | 1335.6 (529.3 to 2429.3) | 77857.2 (30584.3 to 139627.1) |  | 474.0 (161.8 to 880.0) | 21660.5 (7602.7 to 39460.3) |  | 1939.3 (502.2 to 4086.1) | 31333.8 (8708.2 to 64061.5) |  | 332.9 (174.7 to 557.3) | 266.2 (168.8 to 471.4) |  | 172.6 (55.8 to 340.0) | 0.0 (0.0 to 0.0) |  | 89.8 (29.6 to 169.7) | 0.0 (0.0 to 0.0) |  | 103510.1 (70622.8 to 145025.5) | NA |
| Kenya | 81.8 (39.7 to 141.0) | 4915.7 (2421.5 to 8472.1) |  | 61.7 (24.6 to 119.9) | 2953.7 (1168.8 to 5625.3) |  | 363.3 (182.9 to 541.6) | 6459.6 (3322.4 to 9738.7) |  | 216.9 (127.5 to 326.5) | 82.8 (56.7 to 140.4) |  | 66.5 (21.5 to 131.1) | 0.0 (0.0 to 0.0) |  | 34.8 (11.5 to 65.8) | 0.0 (0.0 to 0.0) |  | 39845.2 (27166.9 to 55792.0) | NA |
| Madagascar | 55.2 (23.2 to 113.9) | 3488.3 (1466.2 to 7113.6) |  | 34.9 (12.2 to 78.3) | 1768.8 (615.5 to 3906.7) |  | 291.5 (151.0 to 500.8) | 6057.4 (3139.7 to 10435.0) |  | 72.2 (36.7 to 121.7) | 48.7 (27.6 to 107.7) |  | 32.8 (10.2 to 70.6) | 0.0 (0.0 to 0.0) |  | 16.1 (4.1 to 32.9) | 0.0 (0.0 to 0.0) |  | 22892.3 (15506.8 to 32054.1) | NA |
| Malawi | 37.0 (14.5 to 78.7) | 2306.6 (906.3 to 4930.6) |  | 40.9 (16.2 to 89.4) | 2057.6 (823.9 to 4539.7) |  | 164.6 (84.3 to 322.0) | 3193.6 (1616.0 to 6186.0) |  | 50.3 (26.7 to 83.8) | 47.8 (29.2 to 90.4) |  | 21.5 (6.7 to 46.4) | 0.0 (0.0 to 0.0) |  | 10.8 (2.8 to 22.0) | 0.0 (0.0 to 0.0) |  | 15054.1 (10201.4 to 21065.6) | NA |
| Mozambique | 176.5 (52.6 to 437.5) | 11109.3 (3311.1 to 27527.6) |  | 104.1 (29.8 to 277.6) | 5240.1 (1492.6 to 14121.2) |  | 1027.0 (448.9 to 2208.3) | 21015.2 (9183.8 to 45003.2) |  | 96.4 (50.3 to 161.5) | 104.9 (62.4 to 184.4) |  | 42.5 (13.2 to 91.5) | 0.0 (0.0 to 0.0) |  | 21.1 (5.4 to 43.1) | 0.0 (0.0 to 0.0) |  | 29702.2 (20120.4 to 41588.1) | NA |
| Rwanda | 38.6 (13.7 to 86.5) | 2397.2 (849.7 to 5367.3) |  | 24.2 (8.6 to 58.7) | 1207.8 (422.3 to 2894.2) |  | 194.1 (82.9 to 373.8) | 3546.8 (1487.8 to 6769.5) |  | 36.9 (20.3 to 57.7) | 37.9 (20.1 to 75.8) |  | 13.7 (4.3 to 29.6) | 0.0 (0.0 to 0.0) |  | 6.8 (1.8 to 13.9) | 0.0 (0.0 to 0.0) |  | 9600.8 (6504.8 to 13438.7) | NA |
| Somalia | 76.1 (25.4 to 163.8) | 4775.7 (1574.1 to 10298.2) |  | 34.3 (8.1 to 96.4) | 1721.2 (411.7 to 5001.3) |  | 502.9 (197.7 to 922.6) | 12083.1 (5025.1 to 22225.2) |  | 60.4 (30.2 to 106.6) | 47.9 (27.8 to 95.8) |  | 31.0 (9.6 to 66.8) | 0.0 (0.0 to 0.0) |  | 15.1 (3.9 to 30.8) | 0.0 (0.0 to 0.0) |  | 21674.4 (14673.4 to 30377.7) | NA |
| South Sudan | 44.0 (13.7 to 102.6) | 2714 (833.9 to 6257.4) |  | 31.3 (10.6 to 75.3) | 1540.9 (525.3 to 3611.0) |  | 268.2 (112.5 to 464.0) | 5768.9 (2415.9 to 9632.8) |  | 28.1 (14.3 to 48.2) | 62.3 (18.5 to 211.7) |  | 12.6 (3.9 to 27.1) | 0.0 (0.0 to 0.0) |  | 6.1 (1.6 to 12.5) | 0.0 (0.0 to 0.0) |  | 8806.3 (5960.1 to 12348.4) | NA |
| Uganda | 75.1 (24.3 to 174.7) | 4709.3 (1528.0 to 10875.5) |  | 63.7 (22.2 to 148.8) | 3247.9 (1113.0 to 7742.4) |  | 697.9 (349.6 to 1256.9) | 13155.7 (6461.8 to 24288.3) |  | 166.4 (91.8 to 262.4) | 141.6 (69.9 to 309.4) |  | 59.0 (18.3 to 127.0) | 0.0 (0.0 to 0.0) |  | 29.1 (7.5 to 59.4) | 0.0 (0.0 to 0.0) |  | 41221.0 (27916.7 to 57739.7) | NA |
| United Republic of Tanzania | 309.1 (118.5 to 657.6) | 19229.9 (7416.2 to 41247.9) |  | 200.1 (75.0 to 459.3) | 9964.1 (3653.5 to 22163.6) |  | 1738.7 (799.9 to 3135.5) | 31548.4 (15226.1 to 57749.6) |  | 243.4 (137.2 to 370.6) | 309.0 (157.7 to 704.1) |  | 77.4 (24.0 to 166.8) | 0.0 (0.0 to 0.0) |  | 38.4 (9.9 to 78.4) | 0.0 (0.0 to 0.0) |  | 54148.4 (36673.7 to 75840.5) | NA |
| Zambia | 72.0 (27.1 to 163.6) | 4534.8 (1699.0 to 10297.1) |  | 46.0 (17.7 to 103.7) | 2327.2 (897.3 to 5131.0) |  | 315.4 (145.0 to 627.9) | 5726.1 (2650.3 to 11254.4) |  | 77.7 (42.6 to 118.4) | 73.5 (40.1 to 162.2) |  | 23.5 (7.3 to 50.5) | 0.0 (0.0 to 0.0) |  | 11.7 (3.0 to 23.8) | 0.0 (0.0 to 0.0) |  | 16406.6 (11114.2 to 22971.3) | NA |
| **Southern Sub-Saharan Africa** | 27.7 (16.7 to 40.6) | 1502.5 (919.2 to 2213.8) |  | 19.3 (12.3 to 29.7) | 847.4 (530.7 to 1288.1) |  | 131.6 (90.5 to 187.5) | 1909.1 (1335.5 to 2675.3) |  | 892.6 (588.6 to 1211.6) | 165.4 (118.3 to 223.3) |  | 151.7 (70.3 to 260.6) | 0.0 (0.0 to 0.0) |  | 76.7 (34.9 to 128.9) | 0.0 (0.0 to 0.0) |  | 155618.6 (107679.2 to 211987.6) | NA |
| Botswana | 1.5 (0.6 to 3.1) | 88.5 (35.2 to 183.2) |  | 1.1 (0.4 to 2.4) | 52.4 (19.2 to 114.6) |  | 7.3 (3.0 to 15.0) | 99.1 (42.3 to 206.2) |  | 24.0 (15.3 to 34.1) | 5.4 (3.7 to 7.8) |  | 3.9 (1.6 to 6.9) | 0.0 (0.0 to 0.0) |  | 1.8 (0.8 to 3.2) | 0.0 (0.0 to 0.0) |  | 4310.5 (2966.8 to 5919.3) | NA |
| Eswatini | 0.6 (0.3 to 1.1) | 36.5 (17.5 to 67.9) |  | 0.5 (0.2 to 0.9) | 22.3 (10.3 to 42.9) |  | 2.7 (1.4 to 4.8) | 49.2 (26.7 to 84.6) |  | 14.3 (9.1 to 19.6) | 2.8 (2.0 to 3.8) |  | 2.3 (1.0 to 4.1) | 0.0 (0.0 to 0.0) |  | 1.1 (0.5 to 1.9) | 0.0 (0.0 to 0.0) |  | 2559.0 (1761.5 to 3513.7) | NA |
| Lesotho | 0.8 (0.4 to 1.6) | 49.5 (22.1 to 100.4) |  | 0.5 (0.2 to 1.1) | 26.1 (11.0 to 52.4) |  | 4.0 (1.9 to 7.0) | 77.8 (38.0 to 138.0) |  | 21.9 (13.4 to 30.9) | 3.5 (2.5 to 4.9) |  | 3.6 (1.5 to 6.4) | 0.0 (0.0 to 0.0) |  | 1.7 (0.7 to 3.0) | 0.0 (0.0 to 0.0) |  | 3979.6 (2737.6 to 5468.2) | NA |
| Namibia | 0.8 (0.3 to 1.4) | 46.9 (20.9 to 85.0) |  | 0.6 (0.3 to 1.3) | 31.1 (13.3 to 63.3) |  | 3.8 (1.7 to 6.6) | 57.2 (27.3 to 97.1) |  | 27.8 (18.7 to 38.8) | 5.5 (3.4 to 8.1) |  | 4.9 (2.0 to 8.6) | 0.0 (0.0 to 0.0) |  | 2.3 (1.0 to 4.1) | 0.0 (0.0 to 0.0) |  | 5408.1 (3719.6 to 7432.8) | NA |
| South Africa | 16.6 (9.4 to 24.0) | 821.1 (468.6 to 1188.8) |  | 9.8 (6.3 to 14.6) | 390.1 (250.7 to 571.6) |  | 84.3 (55.2 to 124.5) | 1070.1 (729.2 to 1587.9) |  | 631.2 (413.8 to 853.3) | 79.2 (58.2 to 104.8) |  | 102.1 (47.1 to 173.2) | 0.0 (0.0 to 0.0) |  | 53.1 (24.0 to 86.9) | 0.0 (0.0 to 0.0) |  | 100918.2 (70103.9 to 136654.0) | NA |
| Zimbabwe | 7.5 (3.5 to 13.6) | 459.9 (218.8 to 834.9) |  | 6.8 (3.2 to 12.9) | 325.3 (157.0 to 597.6) |  | 29.6 (16.4 to 50.7) | 555.7 (314.2 to 922.7) |  | 173.3 (113.2 to 246.2) | 68.9 (40.4 to 110.1) |  | 34.8 (14.2 to 61.5) | 0.0 (0.0 to 0.0) |  | 16.6 (7.1 to 28.9) | 0.0 (0.0 to 0.0) |  | 38443.1 (26445.1 to 52823.1) | NA |
| **Western Sub-Saharan Africa** | 747.0 (430.6 to 1175.0) | 44011.9 (25591.5 to 69186.6) |  | 1130.3 (583.7 to 1896.9) | 60815.9 (31020.4 to 104319.9) |  | 2020.7 (896.2 to 3270.7) | 38422.5 (16611.5 to 63267.6) |  | 2153.0 (1282.5 to 3251.4) | 1338.0 (814.0 to 2408.7) |  | 813.1 (275.0 to 1558.3) | 0.0 (0.0 to 0.0) |  | 376.2 (137.1 to 709.6) | 0.0 (0.0 to 0.0) |  | 740756.1 (508590.5 to 1034225.4) | NA |
| Benin | 34.3 (14.7 to 71.7) | 2133.6 (920.3 to 4438.8) |  | 54.6 (21.9 to 127.1) | 2754.0 (1115.2 to 6469.5) |  | 96.4 (38.2 to 189.3) | 1814.4 (714.4 to 3576) |  | 43.5 (22.3 to 71.2) | 42.7 (25.0 to 76.4) |  | 22.3 (7.5 to 44.3) | 0.0 (0.0 to 0.0) |  | 9.9 (3.0 to 19.8) | 0.0 (0.0 to 0.0) |  | 21921.7 (30736.6 to 14931) | NA |
| Burkina Faso | 77.6 (32.8 to 164.2) | 4867.7 (2058.8 to 10275.3) |  | 108.4 (36.5 to 252.1) | 5513.3 (1854.7 to 12939.4) |  | 284.8 (88.7 to 561.0) | 5398.3 (1675.2 to 10541.7) |  | 77.0 (39.6 to 127.8) | 90.8 (49.7 to 187.2) |  | 40.4 (13.6 to 80.4) | 0.0 (0.0 to 0.0) |  | 17.9 (5.5 to 36.0) | 0.0 (0.0 to 0.0) |  | 39783.6 (27095.2 to 55835.5) | NA |
| Cabo Verde | 0.5 (0.2 to 0.9) | 27.1 (10.3 to 50.7) |  | 0.6 (0.2 to 1.3) | 29.8 (10.7 to 66.6) |  | 1.4 (0.6 to 2.9) | 17.1 (7.2 to 35.1) |  | 1. 1 (0.6 to 1.7) | 0.8 (0.4 to 1.3) |  | 0.5 (0.2 to 1.1) | 0.0 (0.0 to 0.0) |  | 0.2 (0.1 to 0.5) | 0.0 (0.0 to 0.0) |  | 523.5 (356.6 to 731.8) | NA |
| Cameroon | 34.4 (14.7 to 68.3) | 2154.3 (922.3 to 4285.0) |  | 78.7 (30.3 to 167.2) | 3997.2 (1552.4 to 8551.6) |  | 131.7 (55.5 to 242.9) | 2318.5 (995.0 to 4190.6) |  | 100.0 (56.1 to 157.1) | 69.9 (35.6 to 142.2) |  | 42.1 (14.1 to 83.6) | 0.0 (0.0 to 0.0) |  | 18.7 (5.7 to 37.5) | 0.0 (0.0 to 0.0) |  | 41418.3 (28213.5 to 57960.9) | NA |
| Chad | 38.7 (15.4 to 82.6) | 2420.8 (962.0 to 5162.3) |  | 63.9 (19.4 to 160.7) | 3239.1 (1000.0 to 7838.2) |  | 132.5 (41.3 to 258.9) | 2823.4 (869.1 to 5629.6) |  | 58.2 (28.7 to 100.4) | 49.7 (27.3 to 105.7) |  | 33.3 (11.2 to 66.2) | 0.0 (0.0 to 0.0) |  | 14.8 (4.5 to 29.8) | 0.0 (0.0 to 0.0) |  | 32780.2 (22325.1 to 46035.7) | NA |
| Côte d'Ivoire | 33.7 (15.1 to 68.0) | 2068.8 (935.5 to 4163.0) |  | 71.9 (28.6 to 149.9) | 3653.6 (1433.3 to 7762.8) |  | 98.4 (39.9 to 191.0) | 1868.3 (794.9 to 3588.8) |  | 91.8 (51.3 to 146.3) | 62.8 (33.6 to 111.2) |  | 40.1 (13.5 to 79.7) | 0.0 (0.0 to 0.0) |  | 17.7 (5.4 to 35.5) | 0.0 (0.0 to 0.0) |  | 39460.8 (26876.8 to 55284.4) | NA |
| Gambia | 1.7 (0.7 to 3.6) | 108.9 (45.2 to 229.2) |  | 2.9 (1.3 to 5.8) | 146.1 (65.6 to 298.0) |  | 2.3 (0.9 to 5.0) | 42.5 (16.8 to 91.0) |  | 5.8 (3.0 to 9.6) | 5.0 (3.0 to 7.6) |  | 3.3 (1.1 to 6.5) | 0.0 (0.0 to 0.0) |  | 1.5 (0.4 to 2.9) | 0.0 (0.0 to 0.0) |  | 3244.5 (2210.1 to 4543.0) | NA |
| Ghana | 22.7 (8.7 to 47.3) | 1423.2 (552.3 to 2968.3) |  | 60.3 (28.0 to 113.8) | 3048.5 (1405.7 to 5655.3) |  | 26.4 (10.6 to 55.1) | 454.7 (184.0 to 966.1) |  | 97.8 (54.2 to 152.5) | 76.6 (41.1 to 128.8) |  | 39.7 (13.3 to 78.9) | 0.0 (0.0 to 0.0) |  | 17.6 (5.4 to 35.2) | 0.0 (0.0 to 0.0) |  | 39073.7 (26615 to 54694.1) | NA |
| Guinea | 19.6 (10.0 to 34.0) | 1218.9 (626.2 to 2114.2) |  | 28.5 (12.6 to 56.7) | 1448.7 (643.6 to 2864.5) |  | 24.6 (12.2 to 46.0) | 498.0 (251.5 to 931.7) |  | 39.1 (20.0 to 65.9) | 40.3 (22.2 to 75.5) |  | 21.4 (7.2 to 42.6) | 0.0 (0.0 to 0.0) |  | 9.5 (2.9 to 19.1) | 0.0 (0.0 to 0.0) |  | 21091.9 (14366.2 to 29556) | NA |
| Guinea-Bissau | 2.3 (1.2 to 4.2) | 145.6 (74.5 to 258.8) |  | 3.9 (1.5 to 8.4) | 195.9 (75.6 to 423.7) |  | 5.9 (2.8 to 11.0) | 124.3 (56.6 to 229.0) |  | 5.5 (2.9 to 9.4) | 3.5 (1.9 to 6.0) |  | 2.9 (1.0 to 5.7) | 0.0 (0.0 to 0.0) |  | 1.3 (0.4 to 2.6) | 0.0 (0.0 to 0.0) |  | 2829.8 (1927.5 to 3961.4) | NA |
| Liberia | 4.6 (2.1 to 8.4) | 295.1 (132.7 to 534.1) |  | 5.9 (2.4 to 11.9) | 298.5 (123.9 to 602.1) |  | 10.5 (4.6 to 19.3) | 200.0 (88.9 to 358.5) |  | 11.3 (5.7 to 19.4) | 5.8 (3.4 to 9.7) |  | 6.3 (2.1 to 12.6) | 0.0 (0.0 to 0.0) |  | 2.8 (0.9 to 5.6) | 0.0 (0.0 to 0.0) |  | 6231.6 (4244.7 to 8719.3) | NA |
| Mali | 41.5 (23.0 to 71.7) | 2601.0 (1438.4 to 4482.6) |  | 71.0 (31.4 to 148.3) | 3623.4 (1570.5 to 7543.8) |  | 75.8 (41.2 to 139.4) | 1440.6 (787.2 to 2648.4) |  | 76.0 (39.1 to 129.4) | 82.7 (47.5 to 154.4) |  | 40.6 (13.6 to 80.7) | 0.0 (0.0 to 0.0) |  | 18.0 (5.5 to 36.2) | 0.0 (0.0 to 0.0) |  | 39970.6 (27221.7 to 56136.3) | NA |
| Mauritania | 2.5 (1.2 to 4.6) | 155.9 (72.6 to 287.6) |  | 3.4 (1.6 to 6.4) | 172.3 (79.1 to 311.9) |  | 5.3 (2.4 to 10.4) | 87.6 (40.2 to 167.6) |  | 11.5 (6.2 to 18.3) | 4.5 (2.6 to 7.7) |  | 5.3 (1.8 to 10.6) | 0.0 (0.0 to 0.0) |  | 2.4 (0.7 to 4.7) | 0.0 (0.0 to 0.0) |  | 5230.9 (3563.4 to 7312.4) | NA |
| Niger | 44.7 (18.8 to 95.0) | 2783.5 (1195.5 to 5883.9) |  | 106.9 (35.1 to 269.1) | 5470.5 (1692.7 to 14086.6) |  | 263.5 (74.3 to 592.1) | 5343.7 (1531.0 to 11954.3) |  | 85.9 (41.6 to 150.7) | 72.7 (41.1 to 161.6) |  | 48.4 (16.3 to 96.3) | 0.0 (0.0 to 0.0) |  | 21.4 (6.5 to 43.2) | 0.0 (0.0 to 0.0) |  | 47712.0 (32492.9 to 67010.0) | NA |
| Nigeria | 334.5 (163.5 to 570.1) | 18250.5 (8870.2 to 31239.8) |  | 397.5 (183.6 to 766.8) | 23600.5 (10983.9 to 45038.2) |  | 721.7 (254.8 to 1428.1) | 13346.8 (4468.6 to 27004.6) |  | 1364.6 (830.6 to 1957.2) | 662.3 (367.4 to 1305.7) |  | 421.4 (144.2 to 797.9) | 0.0 (0.0 to 0.0) |  | 202.5 (70.2 to 366.8) | 0.0 (0.0 to 0.0) |  | 354998.2 (245902.8 to 488287.6) | NA |
| Sao Tome and Principe | 0.1 (0.0 to 0.2) | 5.1 (1.4 to 11.5) |  | 0.1 (0.0 to 0.4) | 7.0 (1.8 to 18.0) |  | 0.3 (0.1 to 0.8) | 4.0 (1.3 to 10.8) |  | 0.5 (0.3 to 0.8) | 0.4 (0.2 to 0.6) |  | 0.2 (0.1 to 0.5) | 0.0 (0.0 to 0.0) |  | 0.1 (0.0 to 0.2) | 0.0 (0.0 to 0.0) |  | 229.8 (156.5 to 321.1) | NA |
| Senegal | 19.2 (9.0 to 37.9) | 1198.6 (576.8 to 2337.3) |  | 26.2 (12.3 to 49.4) | 1321.5 (618.0 to 2463.5) |  | 43.5 (21.4 to 77.3) | 815.0 (397.9 to 1426.0) |  | 38.1 (19.5 to 63.9) | 29.4 (17.0 to 44.6) |  | 21.5 (7.2 to 42.7) | 0.0 (0.0 to 0.0) |  | 9.5 (2.9 to 19.1) | 0.0 (0.0 to 0.0) |  | 21161.7 (14414.2 to 29624.5) | NA |
| Sierra Leone | 27.5 (9.8 to 62.2) | 1722.1 (613.3 to 3903.2) |  | 33.6 (12.5 to 80.3) | 1706.9 (613.4 to 3999.7) |  | 76.5 (26.3 to 158.8) | 1478.1 (504.3 to 3056.1) |  | 23.1 (12.0 to 38.7) | 25.9 (14.2 to 47.7) |  | 12.3 (4.1 to 24.4) | 0.0 (0.0 to 0.0) |  | 5.5 (1.7 to 11.0) | 0.0 (0.0 to 0.0) |  | 12108.3 (8247.7 to 16974.4) | NA |
| Togo | 6.9 (3.6 to 12.3) | 430.5 (224.4 to 767.6) |  | 11.7 (4.3 to 24.0) | 588.4 (227.3 to 1219.0) |  | 19.2 (9.5 to 35.3) | 346.6 (175.4 to 634.5) |  | 22.1 (11.2 to 35.9) | 12.1 (6.4 to 26.3) |  | 11.2 (3.7 to 22.2) | 0.0 (0.0 to 0.0) |  | 4.9 (1.5 to 9.9) | 0.0 (0.0 to 0.0) |  | 10974.5 (7475.7 to 15355.3) | NA |

Data in parentheses are 95% uncertainty intervals (UIs) unless otherwise stated.

Abbreviations: Cases = incidence cases; DALYs= disability-adjusted life-years; SDI = Sociodemographic index; NA= not available.


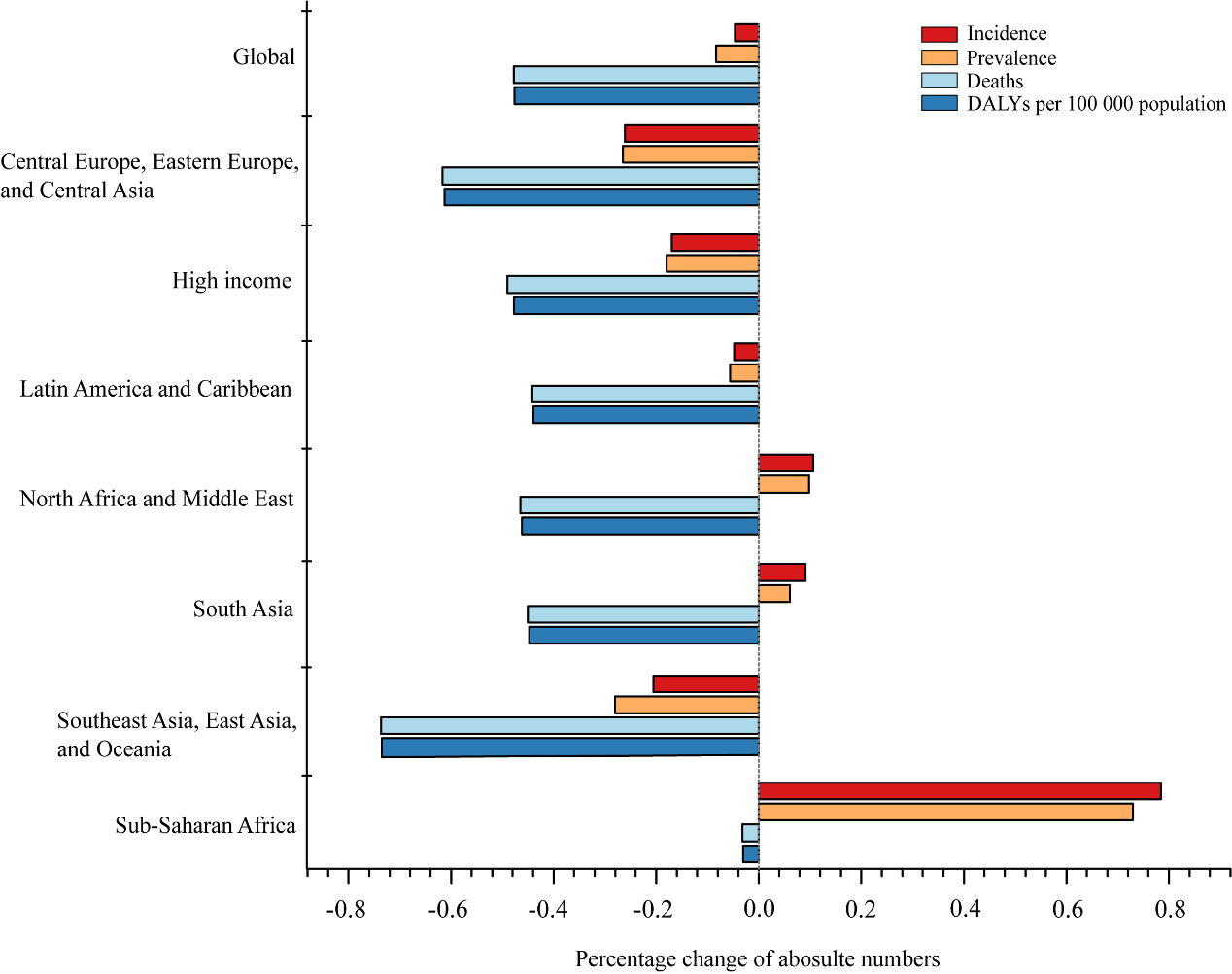


**Supplementary Figure 1.** Percentage changes in absolute numbers of incidence, prevalence, deaths and DALYs of total childhood cancers globally and for 7 GBD super regions between 1990 and 2019.

DALY=disability-adjusted life-years. GBD=Global Burden of Diseases, Injuries and Risk Factors Study.


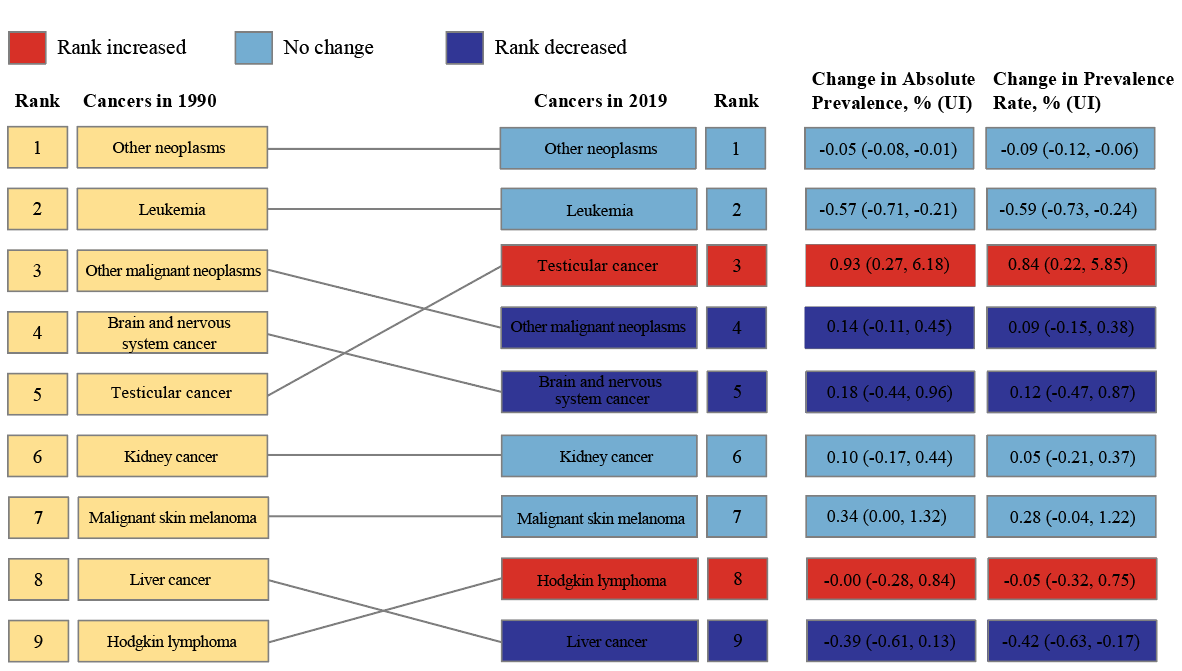


**Supplementary Figure 2.** Leading 9 childhood cancers of global prevalence for 1990 and 2019, with changes of absolute number and rates for both sexes combined. Cancers are ranked by number of prevalence in 1990 and 2019.

The “Non-Hodgkin lymphoma” group is not included in these data because there is no data in this age group available in the result tool of GBD. GBD=Global Burden of Diseases, Injuries, and Risk Factors Study.


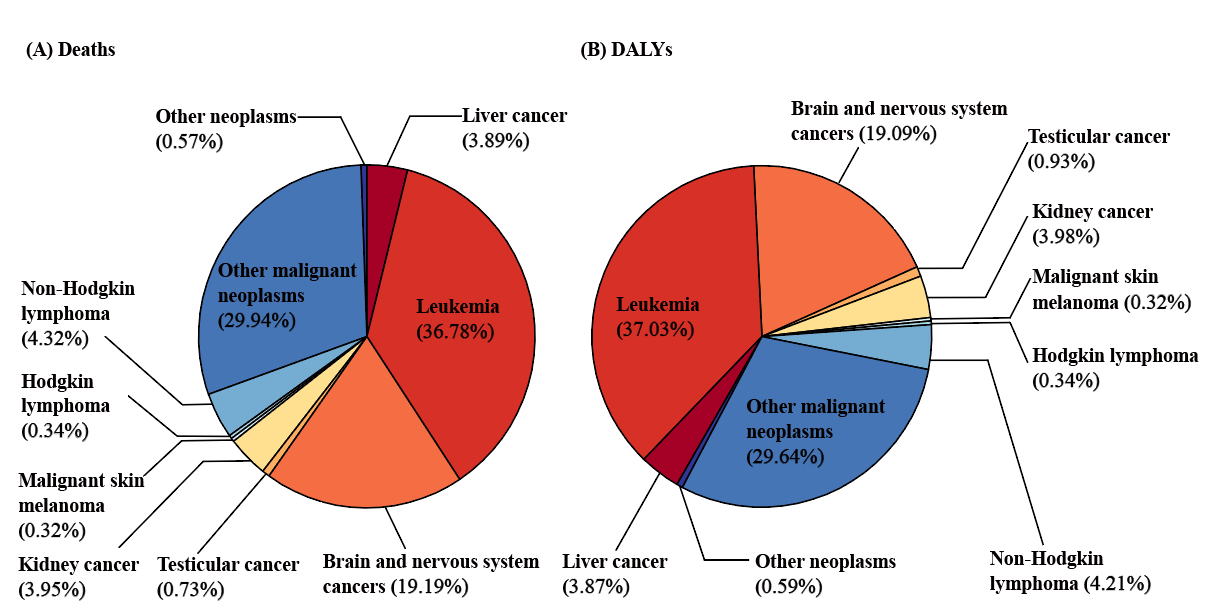


**Supplementary Figure 3.** Relative proportion of deaths (A) and DALYs (B) of each childhood cancers genotype.

Size of pie charts is proportional to the number of deaths and DALY of total childhood cancers.


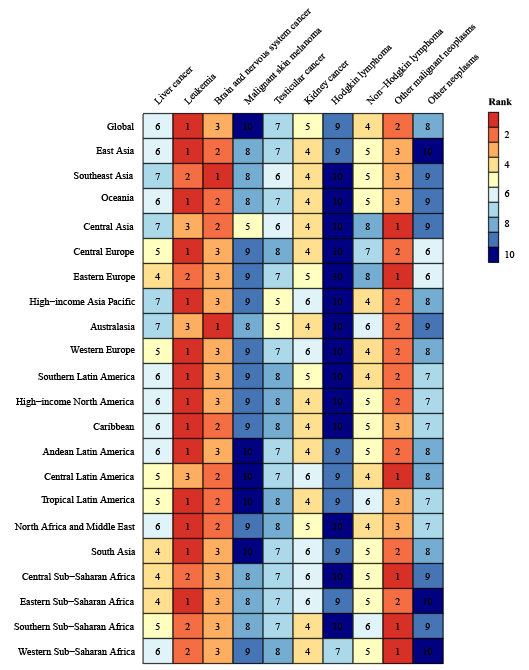


**Supplementary Figure 4.** Childhood cancers ranked by number of DALYs in both sexes, global and by 21 GBD regions in 2019.

Causes in the figure are ordered according to ranks for absolute number of DALYs. Ranks are also color shaded to indicate rank.

DALY=disability-adjusted life-years. GBD=Global Burden of Diseases, Injuries, and Risk Factors Study.


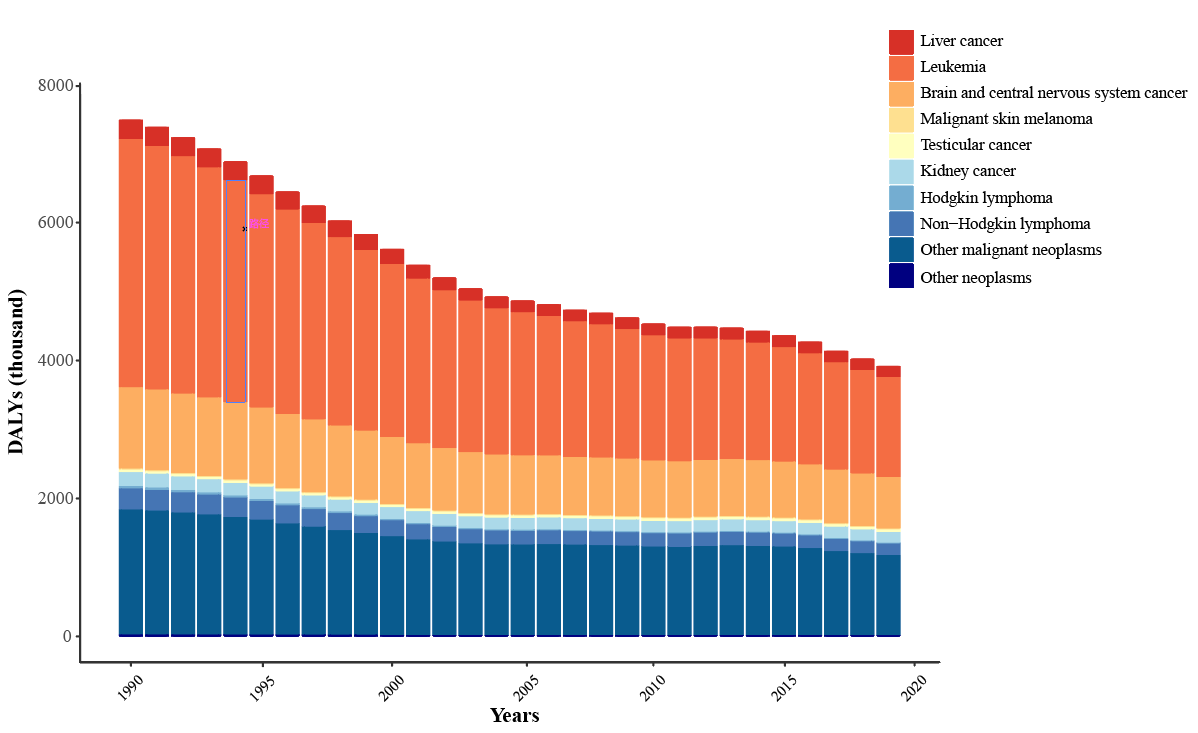


**Supplementary Figure 5.** Global DALYs for 10 childhood cancers between 1990 and 2019. DALY=disability-adjusted life-years.


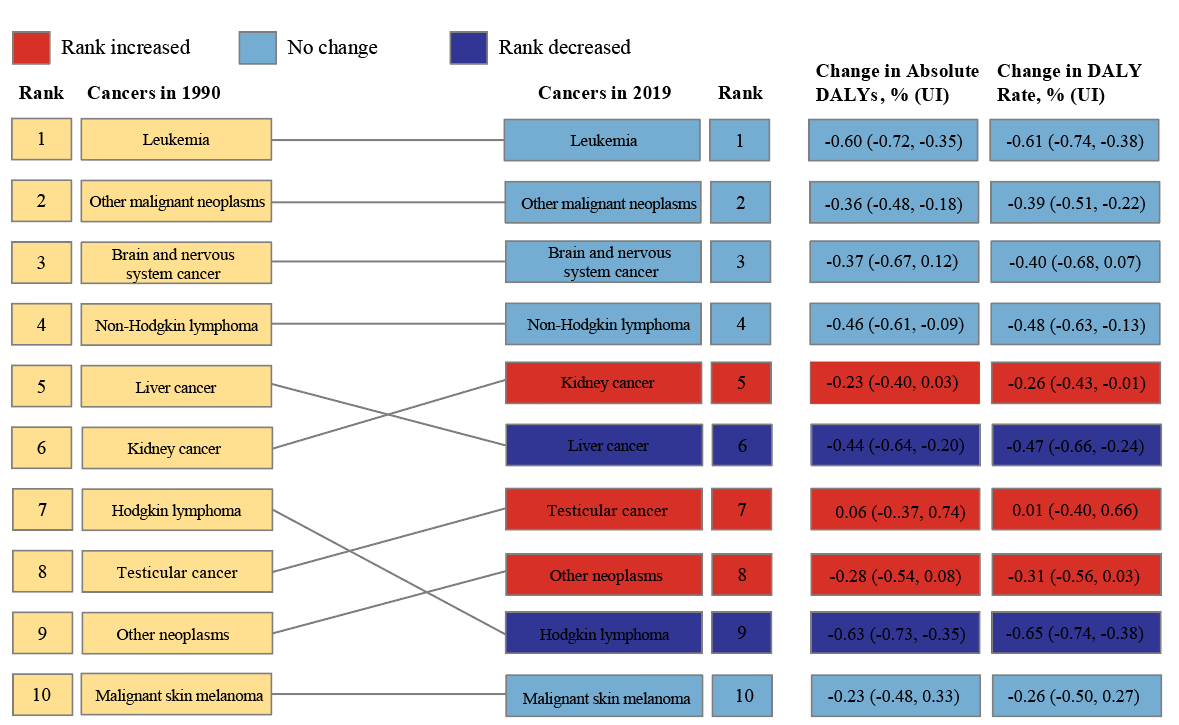


**Supplementary Figure 6.** Childhood cancers ranked globally and for both sexes by absolute DALYs, with changes of absolute number and rates for both sexes combined between 1990 and 2019.

Cancers are ranked by number of DALYs in 1990 and 2019.

DALY=disability-adjusted life-years.
